# Chemoselective Activation of Diethyl Phosphonates: Modular Synthesis of Biologically Relevant Phosphonylated Scaffolds

**DOI:** 10.1002/anie.201806343

**Published:** 2018-09-11

**Authors:** Pauline Adler, Amandine Pons, Jing Li, Jörg Heider, Bogdan R. Brutiu, Nuno Maulide

**Affiliations:** ^1^ Institute of Organic Chemistry, University of Vienna Währinger Strasse 38 1090 Vienna Austria

**Keywords:** phosphinates, phosphonamidates, phosphonates, phosphonothioates, triflic anhydride

## Abstract

Phosphonates have garnered considerable attention for years owing to both their singular biological properties and their synthetic potential. State‐of‐the‐art methods for the preparation of mixed phosphonates, phosphonamidates, phosphonothioates, and phosphinates rely on harsh and poorly selective reaction conditions. We report herein a mild method for the modular preparation of phosphonylated derivatives, several of which exhibit interesting biological activities, that is based on chemoselective activation with triflic anhydride. This procedure enables flexible and even iterative substitution with a broad range of O, S, N, and C nucleophiles.

The phosphonate functional group remains a cornerstone of modern organic chemistry. Indeed, phosphonic acids and derivatives thereof can be found in the scaffolds of a range of bioactive products (Scheme 1).^1^ Among them, aminophosphonates are commonly used as analogues of amino acids.^2^ As phosphonates present enhanced resistance towards hydrolysis, the phosphonate moiety has proven very useful in the development of potential drugs and agrochemicals.^3^

**Scheme 1:**
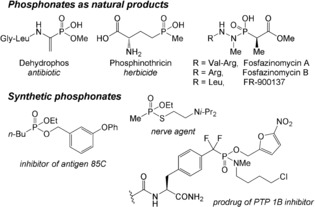
Selected examples of phosphonate‐containing biologically active compounds.

The synthesis of phosphonates classically relies mainly on two different strategies, namely on either the action of a trialkyl phosphite on an alkyl halide (Michaelis–Arbuzov reaction)^4^ or a metal‐mediated coupling with dialkyl phosphite.^5^ Although these methods are efficient, they only lead to symmetric phosphonates (i.e., phosphonates of the form RP(O)(OR′)_2_). To access mixed phosphonates, a general method consists of preforming either a dichloro‐ or a monochlorophosphonyl derivative from a readily available symmetric phosphonate with a strong chlorinating agent, or from a phosphonic acid ester with classical acid activation. These intermediates can then be substituted by different nucleophiles as shown in Scheme 2 a.1e, 6 Depending on the chlorinating agent used, some lack of selectivity between mono‐ and disubstitution was observed when phosphorus pentachloride was used.^7^ Moreover, these are somewhat harsh reagents with low functional group tolerance. However, milder chlorinating agents, such as oxalyl chloride, can be used to efficiently yield the monochlorinated product.^8^ An elegant approach employing copper catalysis and diaryliodonium reagents has been developed to substitute phosphonates, but it is limited to aryloxy modifications.^9^ Selective reductions^10^, ^11^ or arylations^12^ of aryl phosphine oxides or phosphonates have been achieved with different activating agents. The Atherton–Todd reaction is also an elegant approach for the synthesis of phosphonamidates and phosphoramidates with global inversion of configuration.^13^

**Scheme 2:**
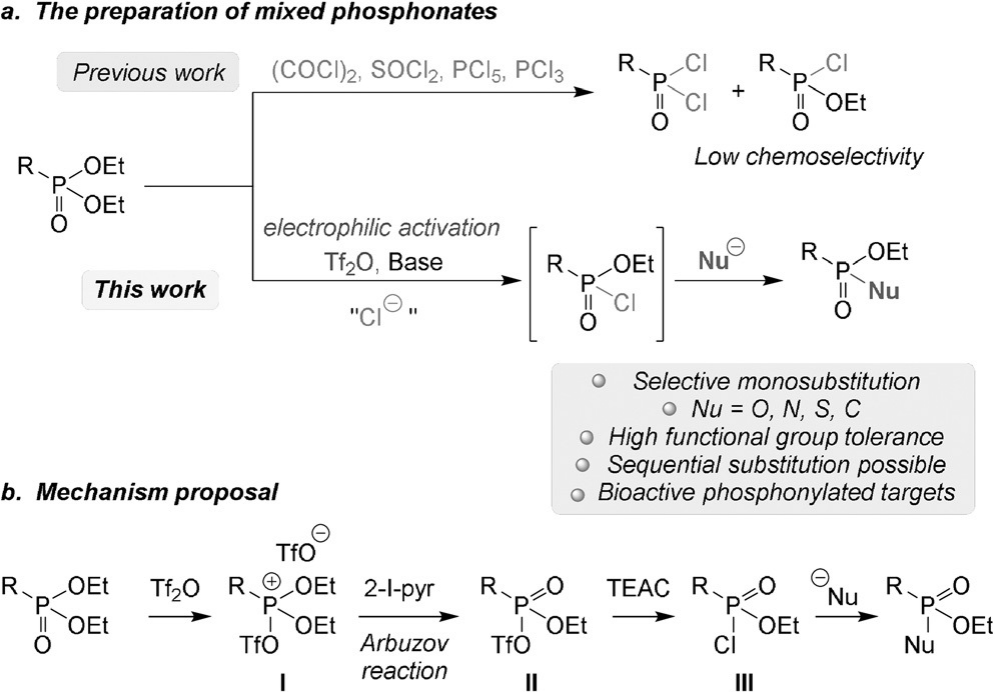
Substitutions of phosphonates and mechanistic proposal. TEAC=tetraethylammonium chloride.

Herein, we present an approach to the substitution of phosphonates. This strategy relies on electrophilic activation with triflic anhydride followed by the addition of a chloride source to selectively and transiently yield a monochlorophosphonyl species. In situ attack by a nucleophile then provides a simple and versatile approach for the synthesis of a range of not only phosphonates, but also phosphonamidates, phosphonothioates, and phosphinates (Scheme 2 a).^14^

Phosphonates can be activated with triflic anhydride to give phosphonium ion **I**. Then, an Arbuzov reaction can occur promoted by triflate and 2‐iodopyridine (see the Supporting Information for details) to yield the mixed phosphonate **II**. Simple substitution with a chloride forms **III**, which ultimately generates the expected product after addition of the deprotonated nucleophile (Scheme 2 b). In contrast to a report by Kang and co‐workers,^14^ the replacement of the triflate by the pyridine on intermediate **II** was not observed.

Our investigations started with phosphonate **2 a** as the substrate and sodium isopropoxide as the nucleophile (Table 1; for full optimization details and mechanistic investigations by ^31^P NMR spectroscopy, see the Supporting Information). We found that reproducibly high yields of product were obtained when 2‐iodopyridine was employed as the base (entry 1) instead of pyridine (entry 2). Using tetraethylammonium chloride as the chloride source (entry 3) avoided the formation of unidentified side products. The use of trifluoromethanesulfonyl chloride did not lead to any conversion (entry 4). Ultimately, the optimized conditions allowed full conversion into the desired product (entry 5), which was isolated in 60 % yield.

**Table 1:** Optimization of the reaction conditiosn. 
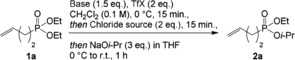

Entry	TfX	Base	Chloride source	**2 a** ^[a]^ [%]	**1 a** ^[a]^ [%]	Other^[a]^ [%]
1	Tf_2_O	2‐I‐pyr	–	71	22	8
2	Tf_2_O	pyridine	–	51	40	9
3	Tf_2_O	2‐I‐pyr	TEAC^[b]^	89	11	0
4	TfCl	2‐I‐pyr	–	nd^[c]^	100	0
5^[d]^	Tf_2_O	2‐I‐pyr	TEAC^[b]^	100	nd^[c]^	0

[a] Yields determined by ^31^P NMR analysis of the crude residue. [b] Tetraethylammonium chloride. [c] Not detected. [d] With fully optimized conditions: 2‐Iodopyridine (1.5 equiv) and triflic anhydride (2 equiv) were added to a solution of the phosphonate (0.2 mmol) in CH_2_Cl_2_ (4 mL) at 0 °C. After 30 min, TEAC (2.5 equiv.) was added at 0 °C. After another 15 min, a solution of the deprotonated nucleophile (4 equiv) in THF (2 mL) was added, and the reaction mixture was stirred at room temperature for 18 h. 2‐I‐pyr=2‐iodopyridine.

First, we delineated the scope and limitations of this reaction with alcohols as nucleophiles (Scheme 3). A broad range of aliphatic alcohols efficiently delivered mixed phosphonates, including isopropyl **2 a**, propargyl (**2 b**, **2 c**), allyl (**2 h**), and electron‐poor alkoxides such as trifluoroethyl (**2 d**) and hexafluoroisopropyl (**2 e**). Phenol could also be used (**2 f**). Interestingly, a protected furanose core was also incorporated (**2 g**).

**Scheme 3:**
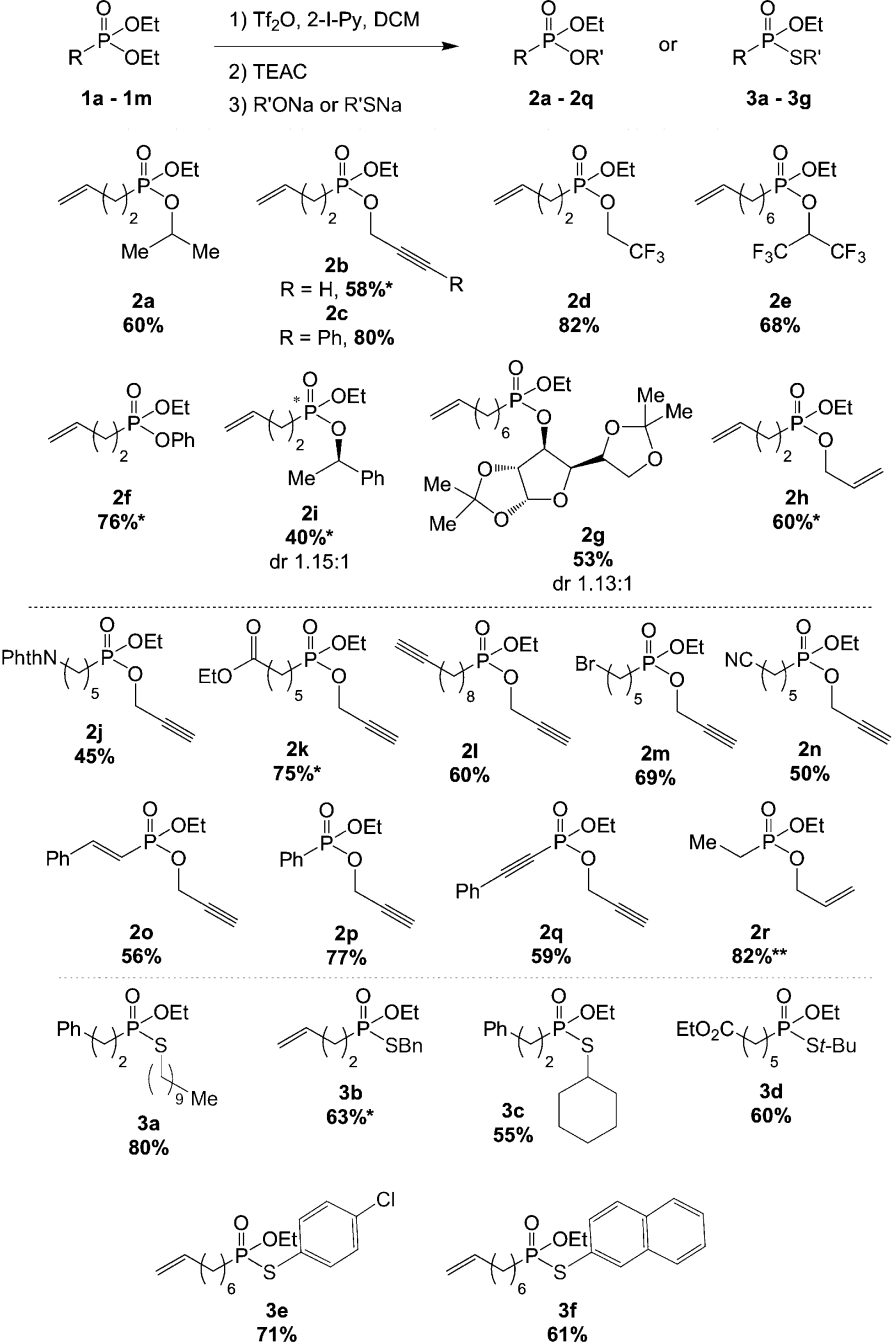
Scope with alcohol and thiol nucleophiles. Yields refer to isolated, pure compounds. 2‐Iodopyridine (1.5 equiv) and triflic anhydride (2 equiv) were added to a solution of the phosphonate (0.2 mmol) in CH_2_Cl_2_ (4 mL) at 0 °C. After 30 min, TEAC (2.5 equiv) was added at 0 °C. After another 15 min, a solution of deprotonated nucleophile (4 equiv) in THF (2 mL) was added, and the reaction mixture was stirred at room temperature for 18 h. * Tetrabutylammonium chloride was used instead of TEAC. ** 15 mmol scale. Phth=phthalimido.

The reaction displays excellent functional group tolerance. Indeed, reactive functional groups such as a phthalimide‐protected amine (**2 j**), an ester (**2 k**), or a nitrile (**2 n**) were all well‐tolerated. This unique chemoselectivity is all the more noteworthy as even a primary alkyl bromide (**2 m**) is tolerated without competing S_N_2 substitution taking place. The use of vinyl, phenyl, or alkynyl phosphonates was also possible (**2 o**–**2 q**). Finally, this method was applied on 15 mmol scale to prepare 2.2 g of the phosphonate **2 r** in a very good 82 % yield.

We then turned our attention to the extension of this method to the formation of phosphonothioates. Gratifyingly, a range of thiols, including decanethiol (**3 a**), benzyl mercaptan (**3 b**), and the bulkier cyclohexanethiol (**3 c**) and *tert*‐butylthiol (**3 d**), were all competent nucleophiles for this process. Finally, substitution with aryl thiols allowed us to prepare **3 e** and **3 f**.

Eager to access phosphonamidates by a similar process, we decided to study the addition of nitrogen nucleophiles (Scheme 4). We found that upon deprotonation with NaH, sulfonamides are competent partners in this reaction. Thus the nosyl phosphonamidate **4 a** and the tosyl phosphonamidates **4 b** and **4 c** were readily accessed, the latter carrying a protected glycine moiety. Valuable amines, such as morpholine, piperazine, and difluoropyrrolidine, were added as their lithium amides to prepare **4 d**, **4 e**, and **4 f**, respectively, in very good yields. The acyclic substrates methylallylamine and dimethylamine were also viable nucleophiles, delivering the corresponding phosphonamidates **4 g** and **4 i** in lower yields whilst benzylamine afforded **4 h** in good yield.

**Scheme 4:**
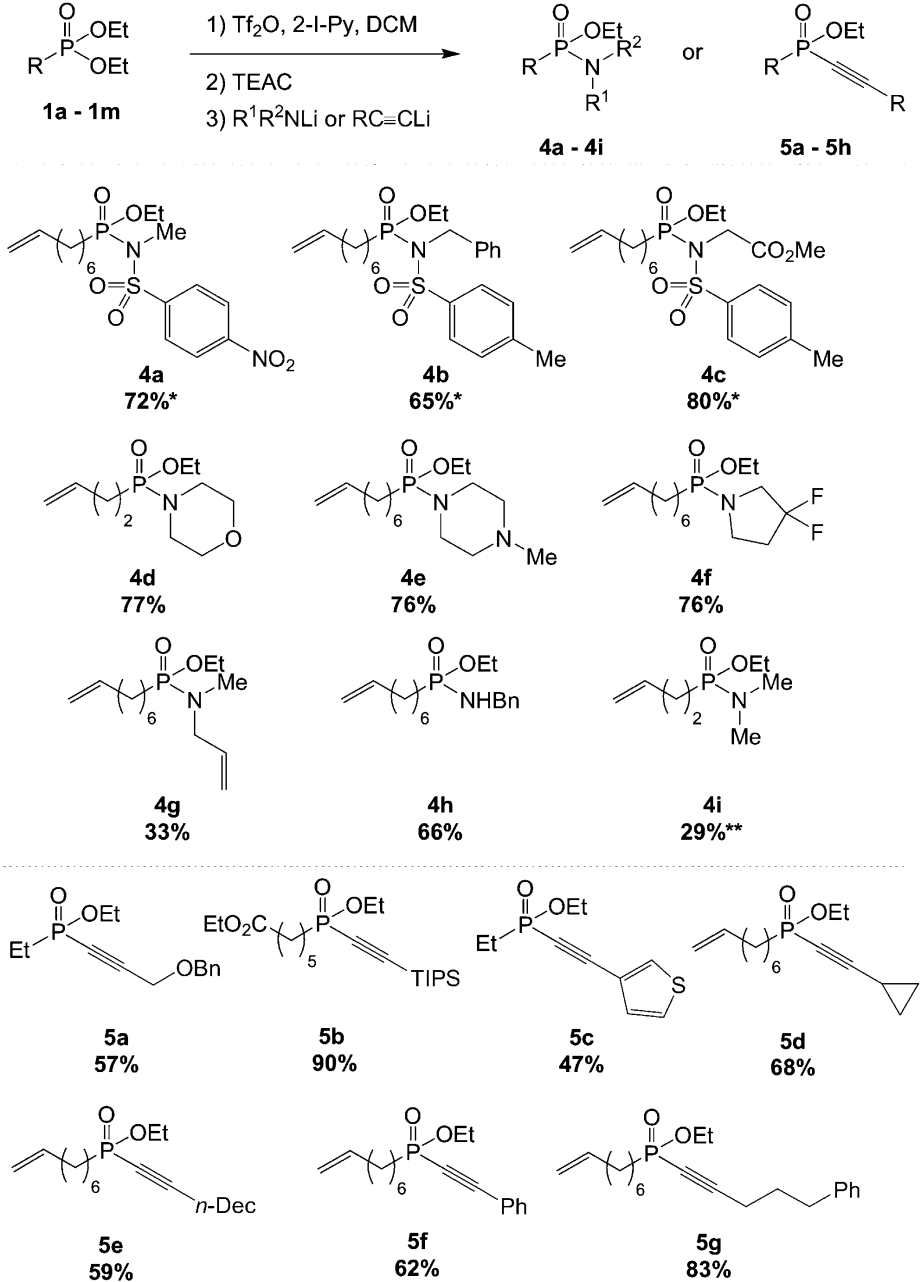
Scope with amine and alkyne nucleophiles. Yields refer to isolated, pure compounds. See the Supporting Information for the reaction conditions. 2‐Iodopyridine (1.5 equiv) and triflic anhydride (2 equiv) were added to a solution of the phosphonate (0.2 mmol) in CH_2_Cl_2_ (4 mL) at 0 °C. After 30 min, TEAC (2.5 equiv) was added at 0 °C. After another 15 min, a solution of deprotonated nucleophile (4 equiv) in THF (2 mL) was added, and the reaction mixture was stirred at room temperature for 18 h. * NaH used for the deprotonation. ** Tetrabutylammonium chloride was used instead of TEAC. Bn=benzyl, TIPS=triisopropylsilyl.

At this point, we envisioned that alkynes could be attractive nucleophiles for the preparation of phosphinates. The terminal alkynes were deprotonated with *n*‐butyllithium prior to addition (Scheme 4). The use of a benzyl‐protected propargyl alcohol led to the formation of **5 a** in good yield. Triisopropylsilylacetylene could also be used to generate phosphinate **5 b** in an excellent yield of 90 %, and a thiophene ring was also tolerated (**5 c**). Phosphinates bearing alkyl chains such as cyclopropyl (**5 d**), decyl (**5 e**), or phenylpropyl (**5 g**) were efficiently prepared. Phenylacetylene could also be used to form **5 f**.

Having demonstrated that a broad range of nucleophiles, including heteroatom nucleophiles, perform competently in this substitution reaction, we were intrigued by the possibility of achieving sequential double substitution. Indeed, as the products still carry one OEt moiety, we hypothesized that renewed activation and substitution would lead to an iterative procedure for decorating a phosphorus center in a flexible manner. Indeed, starting from phosphonate **1 l**, a first substitution with phenol yielded the mixed phosphonate **2 s** in high yield. Renewed activation of **2 s** enabled displacement with morpholine to form the modularly assembled phosphonamidate **6 a** in moderate yield (Scheme 5 a). An iterative substitution was also possible on phosphinate **5 b** with propargyl alcohol, affording product **6 b** in moderate yield.

**Scheme 5:**
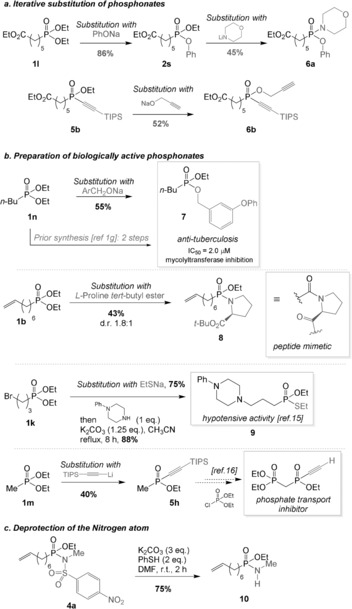
a) Iterative substitution of phosphonates. b) Preparation of biologically active phosphonylated targets. c) Deprotection of the nosyl phosphonamidate.

As mentioned in the introduction, phosphonylated compounds exhibit a wide range of biological activities. We therefore envisaged the preparation of various bioactive targets using this method (Scheme 5 b). For instance, the mixed phosphonate **7** presents antituberculosis properties.1f,1g This compound was readily prepared using the novel method reported herein in a single step from commercially available diethyl butylphosphonate (**1 n**) and the commercially available benzylic alcohol derivative in 55 % yield. The incorporation of phosphonamidates into peptide chains is particularly interesting. In particular, phosphonamide surrogates of glycine‐proline are appealing for their singular stability and reactivity, which are due to the slightly pyramidal nitrogen atom.^15^ In this context, we decided to investigate the use of proline as a nucleophile. Phosphonamidate **8** was indeed accessed in moderate yield. After preparation of phosphonothioate **3 g** from phosphonate **1 k**, displacement of the bromide with *N*‐phenylpiperazine yielded **9**, a compound exhibiting hypotensive activity.^16^ Furthermore, we prepared phosphinate **5 h** as a patented precursor to a phosphate transport inhibitor.^17^ Finally, the nosyl group on phosphonamidate **4 a** could be efficiently cleaved under classical conditions to yield the deprotected compound **10** (Scheme 5 c).

In conclusion, we have developed a mild electrophilic activation method that enables the chemoselective substitution of phosphonates in the presence of a range of functional groups such as esters, nitriles, or halides. By following this procedure, a plethora of O, N, S, and C nucleophiles can be added to efficiently prepare mixed phosphonates, phosphonamidates, phosphonothioates, and phosphinates, respectively (several of them are bioactive substances). We believe that the mild conditions and high functional group tolerance of this procedure are well suited for late‐stage functionalization and the modular decoration of phosphorus centers in medicinal and agricultural chemistry.

## Conflict of interest

The authors declare no conflict of interest.

## Supporting information

As a service to our authors and readers, this journal provides supporting information supplied by the authors. Such materials are peer reviewed and may be re‐organized for online delivery, but are not copy‐edited or typeset. Technical support issues arising from supporting information (other than missing files) should be addressed to the authors.

Supplementary

## References

[1a] G. P. Horsman , D. L. Zechel , Chem. Rev. 2017, 117, 5704–5783; 27787975 10.1021/acs.chemrev.6b00536

[1b] A. J. Wiemer , D. F. Wiemer in Phosphorus Chem. I (Ed.: J.-L. Montchamp), Springer International Publishing, Cham, 2014, pp. 115–160;

[1c] W. W. Metcalf , W. A. van der Donk , Annu. Rev. Biochem. 2009, 78, 65–94; 19489722 10.1146/annurev.biochem.78.091707.100215 PMC2729427

[1d] M.-H. Chen , Z. Chen , B.-A. Song , P. S. Bhadury , S. Yang , X.-J. Cai , D.-Y. Hu , W. Xue , S. Zeng , J. Agric. Food Chem. 2009, 57, 1383–1388; 19199594 10.1021/jf803215t

[1e] I. G. Boutselis , X. Yu , Z.-Y. Zhang , R. F. Borch , J. Med. Chem. 2007, 50, 856–864; 17249650 10.1021/jm061146x

[1f] S. Gobec , I. Plantan , J. Mravljak , R. A. Wilson , G. S. Besra , D. Kikelj , Bioorg. Med. Chem. Lett. 2004, 14, 3559–3562; 15177473 10.1016/j.bmcl.2004.04.052

[1g] S. Gobec , I. Plantan , J. Mravljak , U. Švajger , R. A. Wilson , G. S. Besra , S. L. Soares , R. Appelberg , D. Kikelj , Eur. J. Med. Chem. 2007, 42, 54–63. 17010479 10.1016/j.ejmech.2006.08.007

[2a] V. P. Kukhar , H. R. Hudson , Aminophosphonic and Aminophosphinic Acids: Chemistry and Biological Activity, Wiley, Chichester, New York, 2000;

[2b] A. Woschek , W. Lindner , F. Hammerschmidt , Adv. Synth. Catal. 2003, 345, 1287–1298.

[3] F. Orsini , G. Sello , M. Sisti , Curr. Med. Chem. 2010, 17, 264–289. 20214568 10.2174/092986710790149729

[4a] A. Michaelis , R. Kaehne , Ber. Dtsch. Chem. Ges. 1898, 31, 1048–1055;

[4b] A. E. Arbuzov , J. Russ. Phys. Chem. Soc. 1906, 38, 687;

[4c] A. K. Bhattacharya , G. Thyagarajan , Chem. Rev. 1981, 81, 415–430.

[5a] K. M. Kem , N. V. Nguyen , D. J. Cross , J. Org. Chem. 1981, 46, 5188–5192;

[5b] W. J. Ruan , A. Hassner , Eur. J. Org. Chem. 2001, 1259–1266;

[5c] D. Gelman , L. Jiang , S. L. Buchwald , Org. Lett. 2003, 5, 2315–2318; 12816437 10.1021/ol0346640

[5d] M. Kalek , A. Ziadi , J. Stawinski , Org. Lett. 2008, 10, 4637–4640; 18808138 10.1021/ol801935r

[5e] J. Xu , P. Zhang , Y. Gao , Y. Chen , G. Tang , Y. Zhao , J. Org. Chem. 2013, 78, 8176–8183. 23865378 10.1021/jo4012199

[6] From symmetric phosphonates:

[6a] X. Morise , P. Savignac , J.-M. Denis , J. Chem. Soc. Perkin Trans. 1 1996, 2179;

[6b] M. de F. Fernandez , C. P. Vlaar , H. Fan , Y.-H. Liu , F. R. Fronczek , R. P. Hammer , J. Org. Chem. 1995, 60, 7390–7391;

[6c] P. Fourgeaud , C. Midrier , J.-P. Vors , J.-N. Volle , J.-L. Pirat , D. Virieux , Tetrahedron 2010, 66, 758–764;

[6d] J. Motoyoshiya , T. Kusaura , K. Kokin , S. Yokoya , Y. Takaguchi , S. Narita , H. Aoyama , Tetrahedron 2001, 57, 1715–1721; for selected examples from hydrogen phosphonates:

[6e] K. A. Fredriksen , M. Amedjkouh , Eur. J. Org. Chem. 2016, 474–482;

[6f] M. Quintiliani , J. Balzarini , C. McGuigan , Tetrahedron 2013, 69, 9111–9119;

[6g] M. Van Overtveldt , T. S. A. Heugebaert , I. Verstraeten , D. Geelen , C. V. Stevens , Org. Biomol. Chem. 2015, 13, 5260–5264. 25811608 10.1039/c5ob00137d

[7a] L. Maier , Phosphorus Sulfur Silicon Relat. Elem. 1990, 47, 465–470;

[7b] B. Iorga , D. Carmichael , P. Savignac , C. R. Acad. Sci. Ser. IIc: Chim. 2000, 3, 821–829.

[8] For selected recent reports, see:

[8a] B. J. Foust , M. M. Poe , N. A. Lentini , C.-H. C. Hsiao , A. J. Wiemer , D. F. Wiemer , ACS Med. Chem. Lett. 2017, 8, 914–918; 28947936 10.1021/acsmedchemlett.7b00245 PMC5601366

[8b] S. Bag , R. Jayarajan , R. Mondal , D. Maiti , Angew. Chem. Int. Ed. 2017, 56, 3182–3186; 10.1002/anie.201611360 28206690

[8c] K. Seth , M. Bera , M. Brochetta , S. Agasti , A. Das , A. Gandini , A. Porta , G. Zanoni , D. Maiti , ACS Catal. 2017, 7, 7732–7736.

[9] M. Fañanás-Mastral , B. L. Feringa , J. Am. Chem. Soc. 2014, 136, 9894–9897. 24959810 10.1021/ja505281v

[10] N. P. Kenny , K. V. Rajendran , D. G. Gilheany , Chem. Commun. 2015, 51, 16561–16564. 10.1039/c5cc06389b 26413588

[11] M. J. Petersson , W. A. Loughlin , I. D. Jenkins , Chem. Commun. 2008, 4493–4494. 10.1039/b807695b 18802601

[12] T. Yuan , S. Huang , C. Cai , G. Lu , Org. Biomol. Chem. 2018, 16, 30–33. 10.1039/c7ob02620j 29199738

[13] S. S. Le Corre , M. Berchel , H. Couthon-Gourvès , J.-P. Haelters , P.-A. Jaffrès , Beilstein J. Org. Chem. 2014, 10, 1166–1196. 24991268 10.3762/bjoc.10.117 PMC4077366

[14] During the preparation of this manuscript, Kang and co-workers reported a similar approach for the formation of mixed phosphonates ( H. Huang , J. Denne , C.-H. Yang , H. Wang , J. Y. Kang , Angew. Chem. Int. Ed. 2018, 57, 6624–6628; 10.1002/anie.201802082 29660223

[15] L. Demange , M. Moutiez , C. Dugave , J. Med. Chem. 2002, 45, 3928–3933. 12190314 10.1021/jm020865i

[16] V. S. Reznik , V. D. Akamsin , I. V. Galyametdinova , Russ. Chem. Bull. 2001, 50, 125–129.

[17] C. Huval , A. Dios , *Unsaturated Phosphinyl-Phosphonate Phosphate Transport Inhibitors*, 2004, WO2004085448 (A2).

