# Supplementary material for: Chemoselective Activation of Diethyl Phosphonates: Modular Synthesis of Biologically Relevant Phosphonylated Scaffolds
Source: Angew Chem Int Ed Engl. 2018 Sep 11;57(40):13330–4. doi: 10.1002/anie.201806343 (PMC6175129; doi:10.1002/anie.201806343)
Supplement: Supplementary file 1 — Supplementary [file ANIE-57-13330-s001.pdf]

Angewandte Chemie
Eine Zeitschrift der Gesellschaft Deutscher Chemiker

Supporting Information

Chemoselective Activation of Diethyl Phosphonates: Modular Synthesis of Biologically Relevant Phosphonylated Scaffolds

*Pauline Adler, Amandine Pons, Jing Li, Jörg Heider, Bogdan R. Brutiu, and Nuno Maulide**

anie_201806343_sm_miscellaneous_information.pdf

Table of Contents

1. General Information S3

2. Optimization S4

2.1. Screening of bases S4

2.2 Screening of chloride sources S5

2.3 Screening of activating agent S5

3 NMR studies and proposed mechanism S6

3.1. ^{31}P NMR study S6

3.2. ^{1}H NMR study S7

4 Preparation of the starting materials S10

4.1 Preparation of phosphonates S10

4.2 Characterizations S10

5 Product of substitution S14

5.1 General procedure C S14

5.2. Alcohols as nucleophiles: preparation of mixed phosphonates S14

5.3. Thiols as nucleophiles: preparation of phosphonothioates S24

5.4. Amines and amides as nucleophiles: preparation of phosphonamidates S28

5.5. Alkynes as nucleophiles: preparation of phosphinates S33

6 References S37

7 NMR spectra S38

1. General Information

Unless otherwise stated, all glassware was flame-dried before use and all reactions were performed under an atmosphere of argon. All solvents were distilled from appropriate drying agents prior to use. Triflic anhydride was distilled over P_4O_{10} prior to use. All other reagents were used as received from commercial suppliers unless otherwise stated. Reaction progress was monitored by thin layer chromatography (TLC) performed on aluminium plates coated with silica gel F_{254} with 0.2 mm thickness. Chromatograms were visualized by fluorescence quenching with UV light at 254 nm or by staining using potassium permanganate. Flash column chromatography was performed using silica gel 60 (230-400 mesh, Merck and co.). Neat infra-red spectra were recorded using a Perkin-Elmer Spectrum 100 FT-IR spectrometer. Wavenumbers ($\mathbf{v}_{\mathbf{max}}$) are reported in cm^{-1}. Mass spectra were obtained using a Finnigan MAT 8200 or (70 eV) or an Agilent 5973 (70 eV) spectrometer, using electrospray ionization (ESI). All ^{1}H NMR, ^{13}C NMR, ^{18}F NMR and ^{31}P NMR spectra were recorded using a Bruker AV-400, AV-600 and AV-700 spectrometer at 300K. Chemical shifts were given in parts per million (ppm, δ), referenced to the solvent peak of $CDCl_3$, defined at δ = 7.26 ppm (^{1}H NMR) and δ = 77.16 (^{13}C NMR). Coupling constants are quoted in Hz (*J*). ^{1}H NMR splitting patterns were designated as singlet (s), doublet (d), triplet (t), quartet (q), pentet (p). Splitting patterns that could not be interpreted or easily visualized were designated as multiplet (m) or broad (br). Selected ^{13}C NMR spectra were recorded using the attached proton test (APT) to facilitate the confirmation and assignment of the structure.

2. Optimization

2.1. Screening of bases

Table S1 - Screening of bases

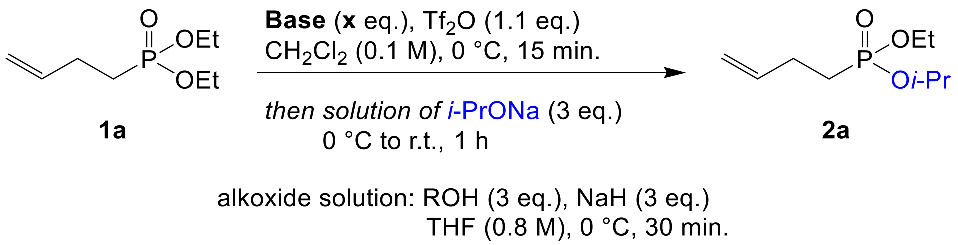

<table>
<tr><th colspan="2">Base (1.5 eq.)</th><th>% desired product</th><th>%starting material</th><th>%unkown</th></tr>
<tr><td colspan="2">2-I-pyr</td><td>71</td><td>22</td><td>8</td></tr>
<tr><td colspan="2">2-F-pyr</td><td>62</td><td>30</td><td>8</td></tr>
<tr><td colspan="2">Triethylamine</td><td>13</td><td>87</td><td>0</td></tr>
<tr><td colspan="2">Proton Sponge®</td><td>60</td><td>34</td><td>6</td></tr>
<tr><td colspan="2">DBU</td><td>12</td><td>60</td><td>27</td></tr>
<tr><td colspan="2">2,6-di-t-Bu-4-Me-pyr</td><td>52</td><td>31</td><td>14</td></tr>
<tr><td colspan="2">2,6-Lutidine</td><td>35</td><td>43</td><td>25</td></tr>
<tr><td colspan="2">Pyridine</td><td>51</td><td>40</td><td>9</td></tr>
<tr><td colspan="2">Pyridine (with TEAC)*</td><td>49</td><td>40</td><td>11</td></tr>
<tr><td colspan="2">2-Cl-pyr</td><td>40</td><td>54</td><td>7</td></tr>
<tr><td colspan="2">2-MeO-pyr</td><td>41</td><td>52</td><td>7</td></tr>
<tr><td colspan="2">4-I-pyr</td><td>13</td><td>83</td><td>4</td></tr>
<tr><td rowspan="4">2-I-pyr</td><td>0.2 eq.</td><td>Not observed</td><td></td><td></td></tr>
<tr><td>1 eq.</td><td>72</td><td></td><td></td></tr>
<tr><td>1.5 eq.</td><td>71</td><td></td><td></td></tr>
<tr><td>2.2 eq.</td><td>75</td><td></td><td></td></tr>
</table>

The percentages have been determined by ^{31}P NMR of the crude residue. DBU = 1,8-Diazabicyclo[5.4.0]undec-7-ene. *modification with TEAC (tetraethylammonium chloride) addition like in fully optimized conditions.

2.2 Screening of chloride sources

Table S2 - Screening of chloride sources

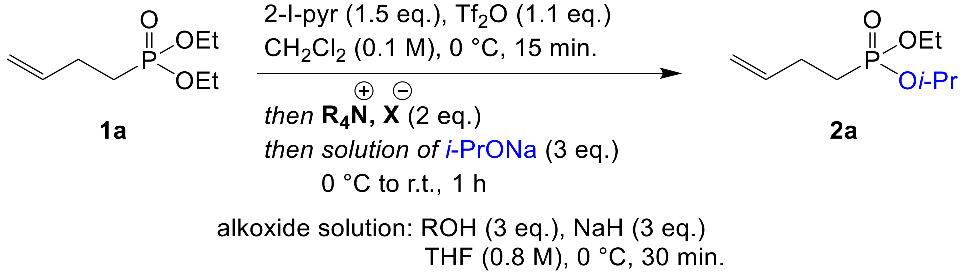

R_4NX	% desired product	%starting material	%unkown
None	71	22	8
TBAC (1 eq.)	32	9	58
TBAC (2 eq.)	84	16	0
TBAB (2 eq.)	36	0	64
TBAI (2 eq.)	77	16	5
TEAC (2 eq.)	***89***	***11***	***0***

The percentages have been determined by ^{31}P NMR of the crude residue. TBAC = Tetrabutylammonium chloride; TBAB = Tetrabutylammonium bromide; TBAI = Tetrabutylammonium iodide; TEAC = Tetraethylammonium chloride.

2.3 Screening of activating agent

Table S3 - Screening activating agent

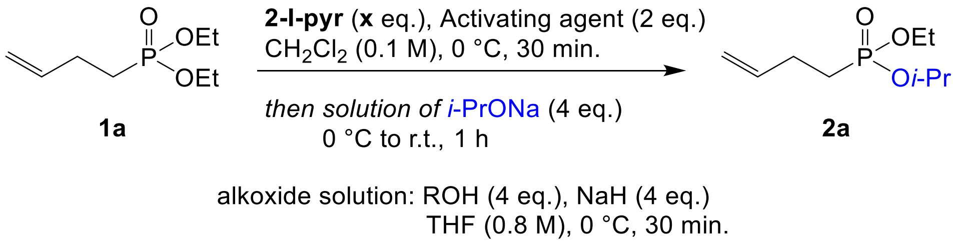

Activating agent	2-I-pyr (x eq.)	% desired product	%starting material	%unkown
Tf_2O (with TEAC)*	***1.5 eq.***	***89***	***11***	***0***
TfCl	1.5 eq.	0	100	0
TfCl	none	0	100	0

The percentages have been determined by ^{31}P NMR of the crude residue. *modification with TEAC (tetraethylammonium chloride) addition like in fully optimized conditions.

3 NMR studies and proposed mechanism

3.1. ^{31}P NMR study

We decided to follow the reaction with this starting with this benzyl diethylphosphonate because Kang *et al*. reported their NMR study on the exact same compound.[1]

The reaction was followed by ^{31}P NMR, (CD_2Cl_2, 243 MHz).

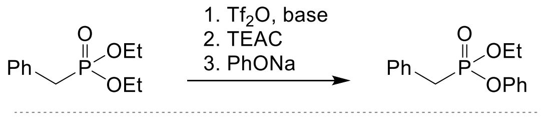

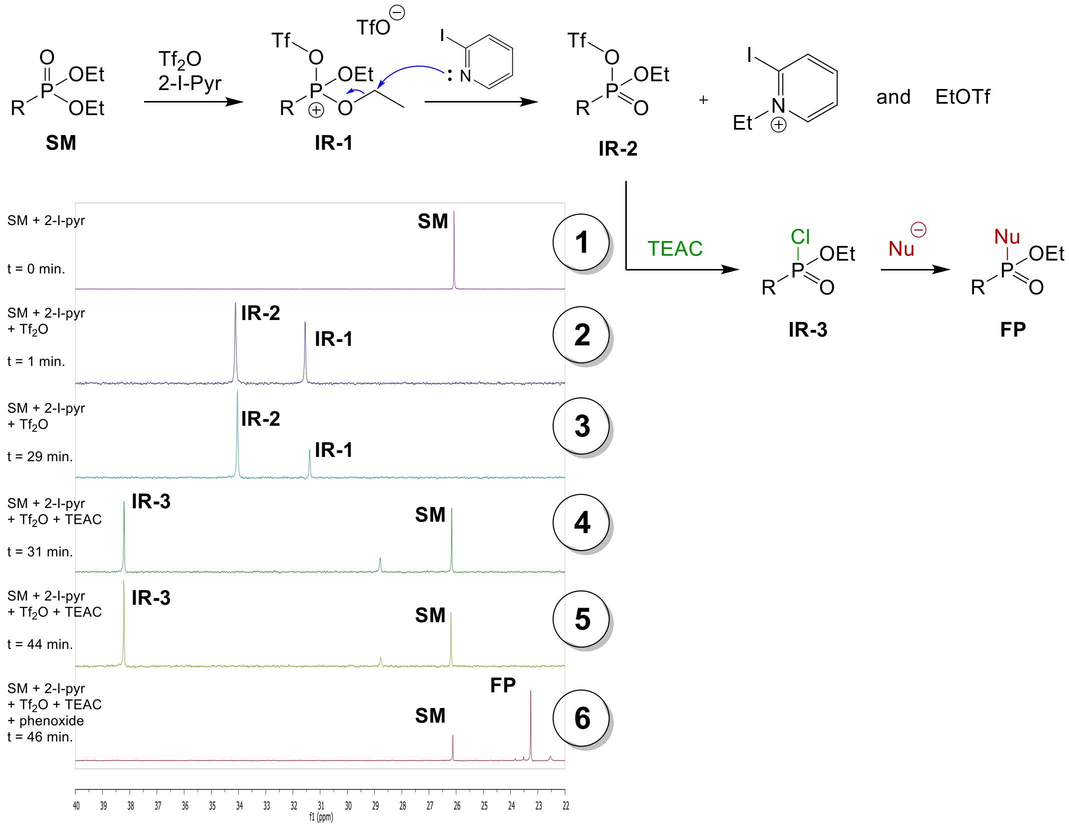

In total, 6 spectra have been measured.

Spectrum 1: starting material (SM) in solution with 2-I-pyridine.

Spectrum 2: Triflic anhydride had just been added. The activation of the phosphonate is total because the peak of SM disappeared. Two intermediates appeared: IR-1 and IR-2.

Spectrum 3: After 15 more minutes, the IR-1 disappear in favour of IR-2 suggesting the Arbuzov reaction noted above.

Spectrum 4: The tetraethylammonium chloride was just added. According to integration, all IR-1 become SM again, while all IR-2 because the phosphoryl chloride IR-3.

Spectrum 5: No changes after 10 minutes.

Spectrum 6: The nucleophile was just added. The peak of the final product (FP) appeared while IR-3 disappeared.

3.2. ^{1}H NMR study

The reaction was followed by ^{1}H NMR, (CD_2Cl_2, 400 MHz).

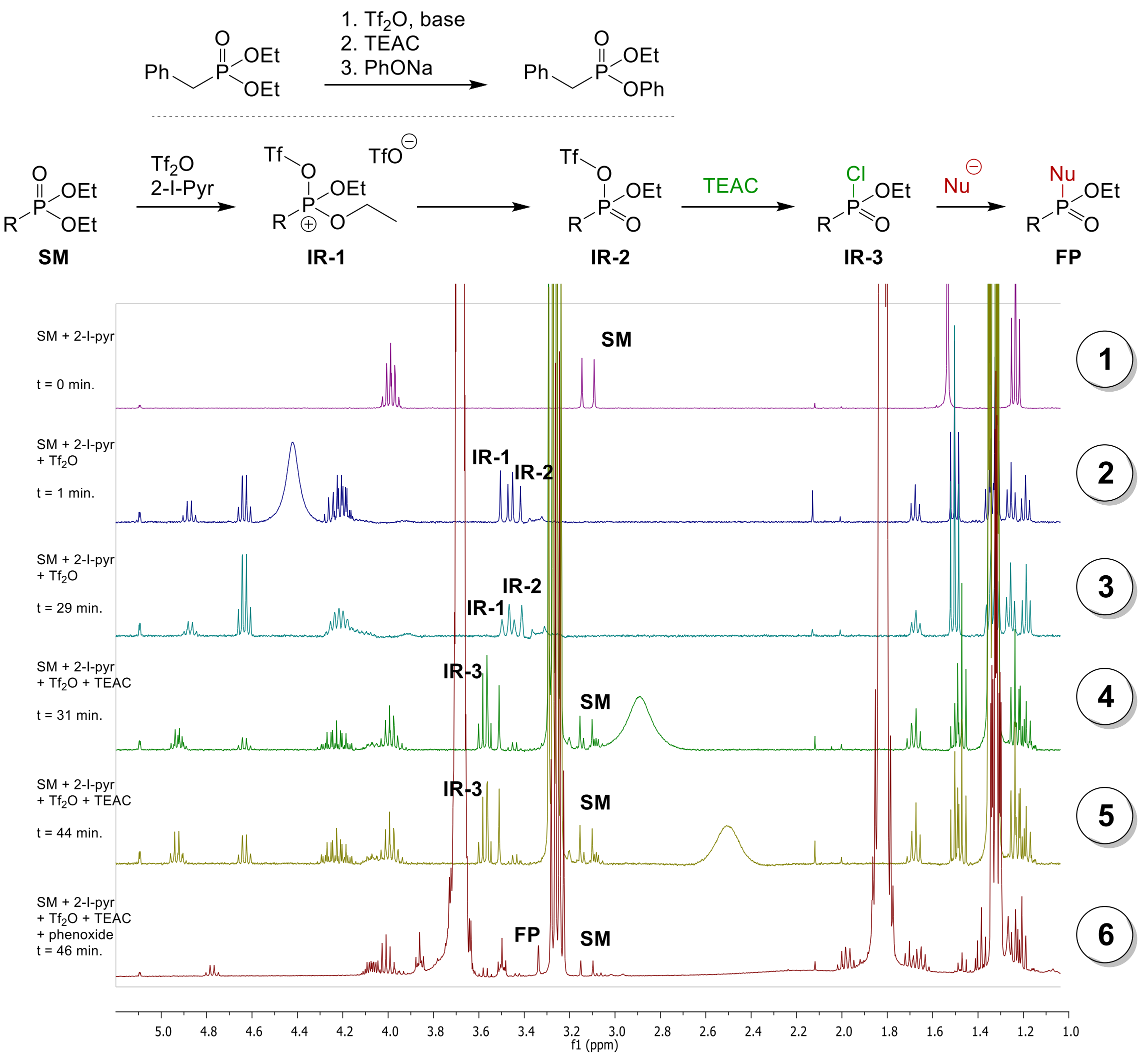

The comments are the same than previously.
On this study, we got particularly interested in the signal of the benzylic CH_2. In particular for the spectra 1, 2 and 3. Indeed, in Kang's paper,[1] the chemical shifts are the following, depending on their intermediate.

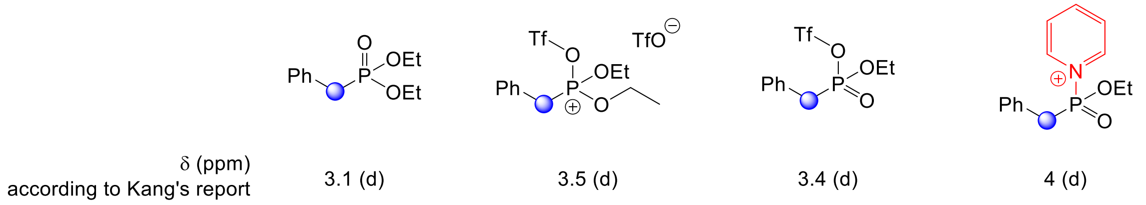

In our case, on the spectrum 1, we clearly observed the doublet of the starting material.

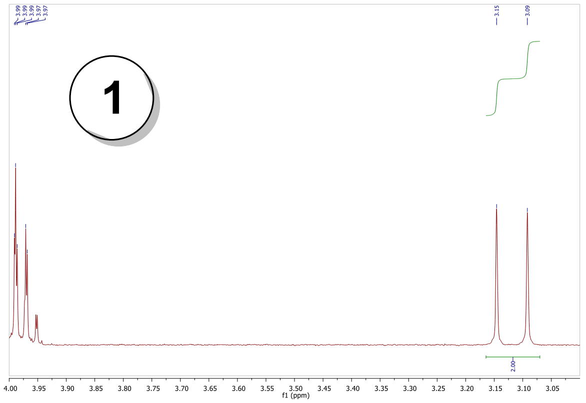

As soon as triflic anhydride was added, this doublet disappeared and two new doublets appeared around 3.4 and 3.5 ppm.

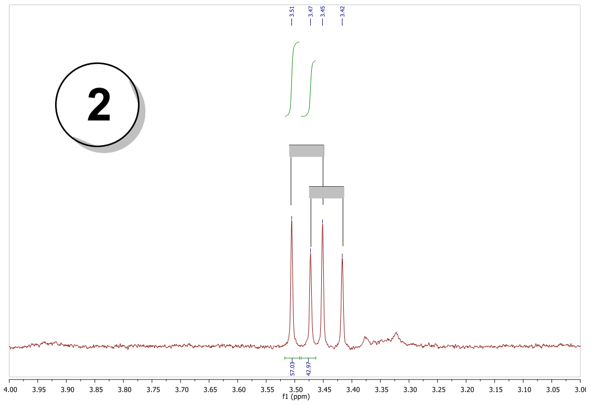

The integration here is 57% for the signal at 3.5 ppm and 43% for the signal at 3.4 ppm. On the phosphorus spectrum 2, we had the same integration: IR-2 (60%) IR-1 (40%).

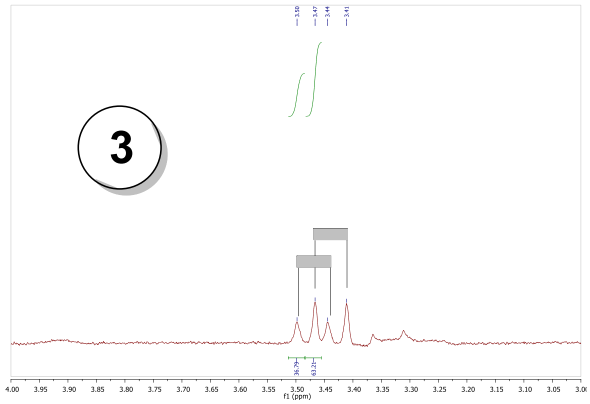

After 30 minutes, the doublet at 3.5 ppm disappeared slowly (integration 36%) and the doublet at 3.4 ppm increased (integration 64%). (Same assessment for phosphorus spectrum)

At this stage, the doublet disappearing seems to be the phosphonium **IR-1** and the doublet appearing the triflyl phosphonate **IR-2**.

It has to be noted that no signal appeared around 4 ppm as Kang and co-workers observed in their work, corresponding to the pyridinium species.

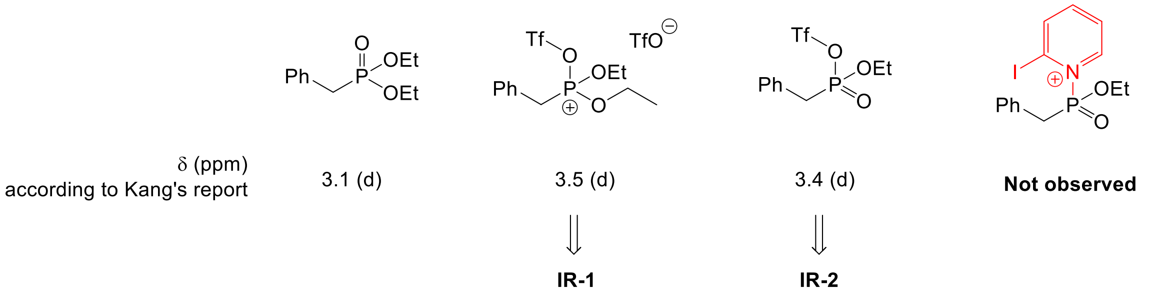

This allows us to conclude that we don’t form the pyridinium species under our conditions. And this supports the proposed mechanism written above.

4 Preparation of the starting materials

4.1 Preparation of phosphonates

General Procedure A:

Trialkylphosphite (1 eq.) and alkyl bromide (1 eq.) were stirred for 3 hours at 240 °C and 20 bar. The resulting crude material was purified by flash column chromatography on silica gel (heptane/ethyl acetate) to afford the desired compound.

General Procedure B:

Diethyl phosphite (6.6 mmol, 1.3 eq.) was added to a suspension of sodium hydride (1.3 eq.) in THF (10 mL) at 0 °C. The mixture was stirred at reflux for 2 hours, then bromoalkane (5 mmol, 1 eq.) was added at 0 °C. The mixture was stirred overnight at room temperature and quenched with water. The aqueous layer was extracted 3 times with dichloromethane, the combined organic layers were dried over $MgSO_4$ and concentrated under reduced pressure. The resulting crude material was purified by flash column chromatography on silica gel (heptane/ethyl acetate) to afford the desired compound.

4.2 Characterizations

Diethyl but-3-en-1-ylphosphonate (1a)

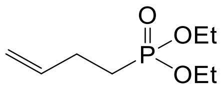

General Procedure A; (85%). All analytical data were in good accordance with data reported in the literature.[2]

Diethyl oct-7-en-1-ylphosphonate (1b)

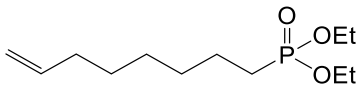

General Procedure B; (94%). All analytical data were in good accordance with data reported in the literature.[3]

Diethyl (5-bromopentyl)phosphonate (1c)

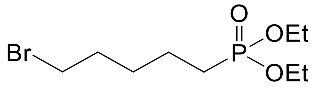

General Procedure B; (14%). All analytical data were in good accordance with data reported in the literature.[4]

Diethyl (5-(1,3-dioxoisoindolin-2-yl)pentyl)phosphonate (1d)

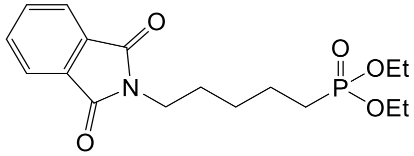

To a solution of diethyl (5-bromopentyl)phosphonate (0.35 mmol, 1 eq.) in DMF (3 mL) was added potassium phthalimide (1.5 eq.) at room temperature. The mixture was stirred at room temperature for 16 hours, then diluted with water. The aqueous layer was extracted 3 times with ethyl acetate, the combined organic layers were dried over $MgSO_4$ and concentrated under reduced pressure. The crude material was purified by flash column chromatography on silica gel (heptane/ethyl acetate) to afford the desired compound in 61% yield. **^{1}H NMR (400 MHz, $CDCl_3$)**: δ 7.85 – 7.78 (m, 2H), 7.72 – 7.67 (m, 2H), 4.12 – 3.98 (m, 4H), 3.66 (t, *J* = 7.2 Hz, 2H), 1.74 – 1.55 (m, 6H), 1.42 (dd, *J* = 15.0, 7.9 Hz, 2H), 1.28 (t, *J* = 7.1 Hz, 6H); **^{13}C NMR (100 MHz, $CDCl_3$):** δ 168.5 (2C), 134.0 (2C), 132.2 (2C), 123.3 (2C), 61.5 (d, *J* = 6.5 Hz, 2C), 37.8, 28.2, 27.9 (d, *J* = 17.0 Hz), 25.7 (d, *J* = 140.9 Hz), 22.2 (d, *J* = 5.2 Hz), 16.6 (d, *J* = 6.0 Hz, 2C); **^{31}P NMR (162 MHz, $CDCl_3$)**: δ 31.9; **IR (neat) ν_{max}:** 2932, 1770, 1706, 1439, 1396, 1368, 1229, 1021, 958, 720; **HRMS (ESI+):** exact mass calculated for $[M+Na]^+$ ($C_{17}H_{24}NNaO_5P$) requires *m/z* 376.1284, found *m/z* 376.1282.

Diethyl dec-9-yn-1-ylphosphonate (1e)

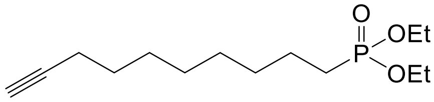

General Procedure A from the iodoalkane; (60%). **^{1}H NMR (600 MHz, $CDCl_3$):** δ 3.98-4.13 (m, 4H), 2.16-2.19 (m, 2H), 1.93 (t, *J* = 6.0 Hz, 1H), 1.68-1.72 (m, 3H), 1.49-1.61 (m, 4H), 1.28-1.41 (m, 13H); **^{13}C NMR (150 MHz, $CDCl_3$)** δ 68.1, 65.4 (d, *J* = 4 Hz), 61.4 (d, *J* = 4.0 Hz), 30.6, 30.5, 28.9, 28.8, 28.6, 28.4, 26.1, 25.2, 22.4 (d, J = 4.0 Hz), 18.4, 16.5 (d, J = 4.0 Hz); **^{31}P NMR (162 MHz, $CDCl_3$):** δ 32.6; **IR (neat) ν_{max}:** 2981, 1230, 1052, 1024, 956, 768, 700, 626, 489; **HRMS (ESI+):** exact mass calculated for $[M+Na]^+$ ($C_{14}H_{27}NaO_3P$) requires *m/z* 297.1590, found *m/z* 297.1590.

Diethyl (5-cyanopentyl)phosphonate (1f)

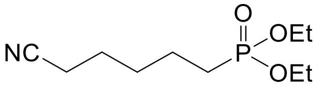

Prepared according to general Procedure B; (62%). **^{1}H NMR (400 MHz, CDCl$_3$):** δ 4.14 – 3.99 (m, 4H), 2.33 (t, *J* = 7.0 Hz, 2H), 1.77 – 1.49 (m, 8H), 1.30 (t, *J* = 7.1 Hz, 6H); **^{13}C NMR (100 MHz, CDCl$_3$)** δ 119.5, 61.6 (d, *J* = 6.5 Hz, 2C), 29.5 (d, *J* = 16.4 Hz), 25.5 (d, *J* = 140.0 Hz), 25.1 (d, *J* = 0.9 Hz), 21.9 (d, *J* = 5.1 Hz), 17.1, 16.55 (d, *J* = 6.0 Hz, 2C); **^{31}P NMR (162 MHz, CDCl$_3$):** δ 31.5; **IR (neat) ν_{max}:** 2934, 2245, 1641, 1446, 1392, 1218, 1163, 1097, 1020, 957, 785; **HRMS (ESI+):** exact mass calculated for $[M+Na]^+$ ($C_{10}H_{20}NNaO_3P$) requires *m/z* 256.1073, found *m/z* 256.1068.

Diethyl (*E*)-styrylphosphonate (1g)

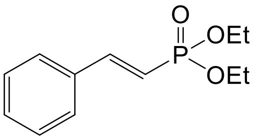

21% yield

(*E*)-β-Nitrostyrene (4 mmol, 1 eq.), diethyl phosphite (1.5 eq.) and silver nitrate (0.2 eq.) in acetonitrile (40 mL) were added in a round bottom flask. The mixture was heated at 90 °C for 2 hours. After stirring for 2 h, the reaction mixture was cooled down to room temperature and diluted with H_2O. The aqueous phase was extracted three times with EtOAc. The organic layers were combined and dried over anhydrous sodium sulfate, filtered and concentrated under reduced pressure. The resulting crude material was purified by flash column chromatography on silica gel (heptane/ethyl acetate) to afford the desired compound in 21% yield.

All analytical data were in good accordance with data reported in the literature.[5]

Diethyl phenylphosphonate (1h)

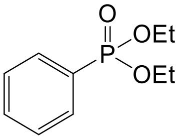

64% yield

To a solution of dichloro(phenyl)phosphine (5.0 mmol, 1 eq.) in diethyl ether (15 mL) were added ethanol (2.1 eq.) and Et_3N (2.1 eq.) at 0 °C. The reaction mixture was allowed to warm up to room temperature. After stirring for 3 hours, the reaction mixture was filtered through a pad of Celite®. The filtrate was concentrated under reduced pressure. The residue was dissolved in THF (5.0 mL) followed by addition at 0 °C of 30% H_2O_2 (1.5 eq.). After stirring for 1 hour at room temperature, the reaction mixture was

quenched with saturated $Na_2S_2O_3$ solution (10 mL). The aqueous phase was extracted with diethyl ether (3×10 mL). The washed solution was dried over anhydrous sodium sulfate, filtered and concentrated under reduced pressure. The resulting crude material was purified by flash column chromatography on silica gel (heptane/ethyl acetate) to afford the desired compound in 64% yield.
All analytical data were in good accordance with data reported in the literature.[6]

Diethyl (phenylethynyl)phosphonate (1i)

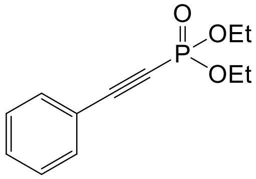

83% yield

To a solution of phenylacetylene (2 mmol, 1 eq.) in THF (7 mL) was added *n*-butyl lithium (1.2 eq., 2.5 M in hexane) at -78 °C. The mixture was stirred at -78 °C for 2 hours, then a solution of diethylchlorophosphate (1.2 eq.) in THF (7mL) was added at -78 °C. The solution was stirred for 1 hour at -78°C, warmed to room temperature and stirred for 3 hours. The mixture was evaporated under reduced pressure. The resulting crude material was purified by flash column chromatography on silica gel (heptane/ethyl acetate) to afford the desired compound in 83% yield.
All analytical data were in good accordance with data reported in the literature.[7]

Diethyl phenethylphosphonate (1j)

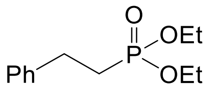

General Procedure A; (70%). All analytical data were in good accordance with data reported in the literature.[8]

Diethyl (5-bromopentyl)phosphonate (1k)

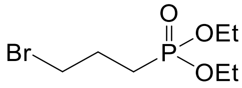

General Procedure B; (50%). All analytical data were in good accordance with data reported in the literature.[9]

5 Product of substitution

5.1 General procedure C

To a solution of phosphonate (0.2 mmol, 1 eq.) and 2-iodopyridine (1.5 eq.) in dichloromethane (4 mL) was added triflic anhydride (2 eq.) at 0 °C. After 30 minutes stirring, the tetraethylammonium chloride salt (2,5 eq.) was added. The solution was stirred 15 minutes at 0 °C followed by the addition of the solution of the deprotonated nucleophile in THF (4 eq. of nucleophile + 4 eq. of Base in 1 mL of THF). The reaction mixture was stirred at room temperature during 16 hours and quenched with a saturated solution of ammonium chloride. After separation of the phases, the aqueous phase was extracted three times with ethyl acetate. The organic phases were combined and dried on magnesium sulfate. After filtration and evaporation, the crude was then purified by flash column chromatography on silica gel.

5.2. Alcohols as nucleophiles: preparation of mixed phosphonates

Ethyl isopropyl but-3-en-1-ylphosphonate (2a)

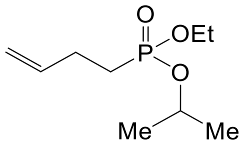

Prepared according to general procedure C; 60% yield; **^{1}H NMR (400 MHz, CDCl$_3$)**: δ 5.84 (ddt, *J* = 16.7, 10.2, 6.4 Hz, 1H), 5.11 – 4.94 (m, 2H), 4.76 – 4.63 (m, 1H), 4.16 – 3.99 (m, 2H), 2.41 – 2.26 (m, 2H), 1.86 – 1.71 (m, 2H), 1.36 – 1.27 (m, 9H); **^{13}C NMR (100 MHz, CDCl$_3$):** δ 137.5 (d, *J* = 17.8 Hz), 115.2, 70.3 (d, *J* = 6.6 Hz), 61.4 (d, *J* = 6.3 Hz), 26.8 (d, *J* = 4.2 Hz), 25.8 (d, *J* = 141.6 Hz), 24.2, 16.6 (d, *J* = 6.0 Hz); **^{31}P NMR (162 MHz, CDCl$_3$):** δ 30.4; **IR (neat) ν_{max}:** 2980, 2929, 1642, 1442, 1386, 1283, 1240, 1216, 1045, 998, 962, 914, 816, 781; **HRMS (ESI+):** exact mass calculated for $[M+Na]^+$ ($C_9H_{19}NaO_3P$) requires *m/z* 229.0970, found *m/z* 229.0964.

Ethyl prop-2-yn-1-yl but-3-en-1-ylphosphonate (2b)

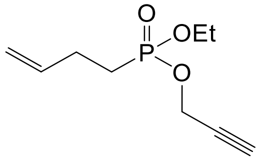

Prepared according to general procedure C (tetrabutylammonium chloride was used instead of tetraethylammonium; extraction with dichloromethane); 58% yield; **^{1}H NMR (400 MHz, CDCl$_3$)**: δ 5.84 (ddt, *J* = 16.7, 10.2, 6.4 Hz, 1H), 5.06 (ddd, *J* = 17.1, 3.2, 1.6 Hz, 1H), 5.01 (ddd, *J* = 10.2, 2.7, 1.3 Hz, 1H), 4.66 (ddd, *J* = 10.6, 2.5, 0.8 Hz, 2H), 4.22 – 4.03 (m, 2H), 2.53 (t, *J* = 2.5 Hz, 1H), 2.43 – 2.31 (m, 2H), 1.96 – 1.82 (m, 2H), 1.33 (t, *J* = 7.1 Hz, 3H); **^{13}C NMR (100 MHz, CDCl$_3$):** δ 137.0 (d, *J* = 17.8 Hz), 115.2, 75.4 (d, *J* = 3.0 Hz), 61.8 (d, *J* = 6.8 Hz), 53.0 (d, *J* = 5.6 Hz), 26.3 (d, *J* = 4.6 Hz), 25.3 (d, *J* = 140.5 Hz), 16.3 (d, *J* = 6.1 Hz); **^{31}P NMR (162 MHz, CDCl$_3$):** δ 33.0; **IR (neat) ν_{max}:** 2982, 2930, 2122, 1642, 1445, 1394, 1371, 1331, 1241, 1217, 1051,1020, 995, 962, 916, 823, 751, 716; **HRMS (ESI+):** exact mass calculated for $[M+Na]^+$ ($C_9H_{15}NaO_3P$) requires *m/z* 225.0657, found *m/z* 225.0650.

Ethyl (3-phenylprop-2-yn-1-yl) but-3-en-1-ylphosphonate (2c)

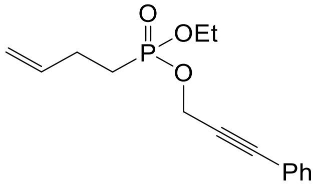

Prepared according to general procedure C; 80% yield; **^{1}H NMR (600 MHz, CDCl$_3$)**: δ 7.47 – 7.40 (m, 2H), 7.37 – 7.29 (m, 3H), 5.85 (ddt, *J* = 16.7, 10.2, 6.4 Hz, 1H), 5.08 – 4.98 (m, 2H), 4.90 (dd, *J* = 10.6, 1.7 Hz, 2H), 4.21 – 4.11 (m, 2H), 2.44 – 2.37 (m, 2H), 1.96 – 1.88 (m, 2H), 1.34 (t, *J* = 7.1 Hz, 3H); **^{13}C NMR (150 MHz, CDCl$_3$):** δ 137.2 (d, *J* = 17.9 Hz), 132.0, 129.0, 128.5, 122.1, 115.4, 87.2, 83.8 (d, *J* = 5.8 Hz), 61.9 (d, *J* = 6.8 Hz), 54.1 (d, *J* = 5.6 Hz), 26.6 (d, *J* = 4.4 Hz), 25.5 (d, *J* = 140.2 Hz), 16.5 (d, *J* = 6.2 Hz); **^{31}P NMR (243 MHz, CDCl$_3$):** δ 33.0; **IR (neat) ν_{max}:** 3079, 2981, 2928, 1641, 1599, 1491, 1443, 1242, 1216, 1048, 1016, 992, 962, 915, 823, 756, 691; **HRMS (ESI+):** exact mass calculated for $[M+Na]^+$ ($C_{15}H_{19}NaO_3P$) requires *m/z* 301.0964, found *m/z* 301.0961.

Ethyl (2,2,2-trifluoroethyl) but-3-en-1-ylphosphonate (2d)

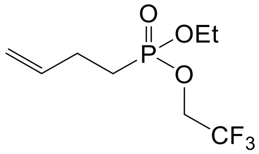

Prepared according to general procedure C; 82% yield; **^{1}H NMR (400 MHz, $CDCl_3$)**: δ 5.83 (ddt, *J* = 16.7, 10.2, 6.4 Hz, 1H), 5.07 (dd, *J* = 17.1, 1.5 Hz, 1H), 5.02 (dd, *J* = 10.2, 1.2 Hz, 1H), 4.35 (m, *J* = 8.2 Hz, 2H), 4.23 – 4.03 (m, 2H), 2.41 – 2.29 (m, 2H), 1.97 – 1.83 (m, 2H), 1.33 (t, *J* = 7.1 Hz, 3H); **^{13}C NMR (150 MHz, $CDCl_3$):** δ 136.6 (d, *J* = 17.6 Hz), 122.9 (qd, *J* = 277.6, 7.7 Hz), 115.6, 62. (d, *J* = 7.0 Hz), 61.9 (qd, *J* = 37.5, 7.4 Hz), 26.2 (d, *J* = 4.8 Hz), 25.0 (d, *J* = 141.7 Hz), 16.3 (d, *J* = 6.2 Hz); **^{31}P NMR (162 MHz, $CDCl_3$):** δ 33.0; **^{19}F NMR (565 MHz, $CDCl_3$):** δ -75.3 (t, *J* = 8.3 Hz); **IR (neat) ν_{max}:** 2987, 2918, 1643, 1447, 1418, 1288, 1248, 1200, 1163, 1092, 1032, 963, 917, 839, 758, 652; **HRMS (ESI+):** exact mass calculated for $[M+Na]^+$ ($C_8H_{14}NaO_3PF_3$) requires *m/z* 269.0530, found *m/z* 269.0527.

Ethyl (1,1,1,3,3,3-hexafluoropropan-2-yl) oct-7-en-1-ylphosphonate (2e)

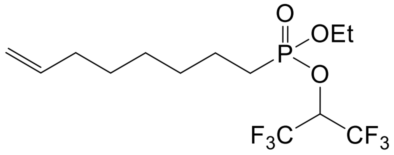

Prepared according to general procedure C; 68% yield; **^{1}H NMR (700 MHz, $CDCl_3$)**: δ 5.79 (ddt, *J* = 16.9, 10.2, 6.7 Hz, 1H), 5.27 – 5.21 (m, 1H), 5.02 – 4.91 (m, 2H), 4.25 – 4.16 (m, 1H), 4.12 – 4.04 (m, 1H), 2.04 (dd, *J* = 14.4, 7.0 Hz, 2H), 1.88 – 1.81 (m, 2H), 1.66 – 1.60 (m, 2H), 1.43 – 1.36 (m, 4H), 1.35 – 1.29 (m, 5H); **^{13}C NMR (176 MHz, $CDCl_3$):** δ 139.0, 120.8 (q, *J* = 282.9 Hz), 114.6, 70.2 (dtd, *J* = 69.3, 34.5, 5.0 Hz), 62.4 (d, *J* = 7.6 Hz), 33.8, 30.3 (d, *J* = 17.6 Hz), 28.7, 28.5, 26.2 (d, *J* = 141.1 Hz), 22.1 (d, *J* = 5.7 Hz), 16.2 (d, *J* = 6.6 Hz); **^{31}P NMR (162 MHz, $CDCl_3$):** δ 35.0; **^{19}F NMR (659 MHz, $CDCl_3$):** δ -74.1 (t, *J* = 6.4 Hz), -74.2 (t, *J* = 6.5 Hz); **IR (neat) ν_{max}:** 2934, 2860, 1642, 1384, 1295, 1251, 1222, 1196, 1107, 1031, 995, 970, 901, 874, 687; **HRMS (ESI+):** exact mass calculated for $[M+Na]^+$ ($C_{13}H_{21}NaO_3PF_6$) requires *m/z* 393.1025, found *m/z* 393.1025.

Ethyl phenyl but-3-en-1-ylphosphonate (2f)

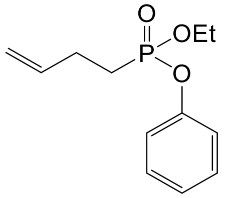

Prepared according to general procedure C; (tetrabutylammonium chloride was used instead of tetraethylammonium; extraction with dichloromethane); 76% yield; **^{1}H NMR (400 MHz, $CDCl_3$)**: δ 7.40 – 7.28 (m, 2H), 7.22 – 7.18 (m, 2H), 7.17 – 7.12 (m, 1H), 5.85 (ddt, *J* = 16.7, 10.2, 6.4 Hz, 1H), 5.07 (ddd, *J* = 17.1, 3.1, 1.5 Hz, 1H), 5.01 (dd, *J* = 10.2, 1.3 Hz, 1H), 4.26 – 4.07 (m, 2H), 2.52 – 2.36 (m, 2H), 2.04 – 1.90 (m, 2H), 1.29 (t, *J* = 7.1 Hz, 3H); **^{13}C NMR (100 MHz, $CDCl_3$):** δ 150.6 (d, *J* = 8.4 Hz), 136.8 (d, *J* = 17.8 Hz), 129.7 (2C), 124.8 (d, *J* = 0.9 Hz), 120.4 (d, *J* = 4.2 Hz,, 2C), 115.4, 62.4 (d, *J* = 6.9 Hz), 26.4 (d, *J* = 4.7 Hz), 25.1 (d, *J* = 141.2 Hz), 16.3 (d, *J* = 5.9 Hz); **^{31}P NMR (162 MHz, $CDCl_3$):** δ 28.4; **IR (neat) ν_{max}:** 2982 ,2912 ,1642, 1592, 1489, 1445, 1394, 1254, 1204, 1163, 1097, 1035, 962, 916, 811, 752, 691; **HRMS (ESI+):** exact mass calculated for $[M+Na]^+$ ($C_{12}H_{17}NaO_3P$) requires *m/z* 263.0813, found *m/z* 263.0812.

(3a*R*,5*R*,6*S*,6a*R*)-5-((*R*)-2,2-Dimethyl-1,3-dioxolan-4-yl)-2,2-dimethyltetrahydrofuro[2,3-d][1,3]dioxol-6-yl ethyl oct-7-en-1-ylphosphonate (2g)

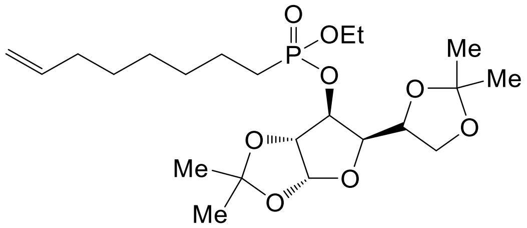

Prepared according to general procedure C; 53% yield, d.r. 1.13:1; **^{1}H NMR (400 MHz, $CDCl_3$)**: δ 5.88 (t, *J* = 3.3 Hz, 1H), 5.79 (ddt, *J* = 16.9, 10.2, 6.7 Hz, 1H), 5.02 – 4.95 (m, 1H), 4.93 (ddd, *J* = 10.2, 2.1, 1.0 Hz, 1H), 4.83 (dd, *J* = 8.2, 2.4 Hz, 1H), 4.72 (dd, *J* = 18.3, 3.6 Hz, 1H), 4.25 – 4.05 (m, 5H), 4.00 (ddd, *J* = 8.6, 5.2, 2.8 Hz, 1H), 2.03 (q, *J* = 6.6 Hz, 2H), 1.83 – 1.69 (m, 2H), 1.65 – 1.55 (m, 2H), 1.49 (s, 3H), 1.43 – 1.28 (m, 18H); **^{13}C NMR (100 MHz, $CDCl_3$):** δ 139, 114.5, 112.5 (dia1), 112.5 (dia2), 109.5 (dia1), 109.4 (dia2), 105.2 (dia1), 105.2 (dia2), 84.4 (dia2), 84.1 (dia1), 80.8 (d, *J* = 7.2 Hz, dia1), 80.7 (d, *J* = 7.0 Hz, dia2), 78.0 (d, *J* = 5.7 Hz, dia1), 77.8 (d, *J* = 7.1 Hz, dia2), 72.4 (dia1), 72.3 (dia2), 67.6 (dia1), 67.5 (dia2), 62.3 (d, *J* = 6.5 Hz, dia2), 61.6 (d, *J* = 6.7 Hz, dia1), 33.8 (dia1), 33.8 (dia2), 30.7 (d, *J* = 18.1 Hz, dia1), 30.5 (d, *J* = 17.3 Hz, dia2), 28.8 (dia1), 28.8 (dia2), 28.7 (dia1), 28.7 (dia2), 27.0, 27.0 (dia1), 26.9 (dia2), 26.4 (dia1), 26.4 (dia2), 26.3 (d, *J* = 141.0 Hz, dia1), 25.9 (d, *J* = 138.0 Hz, dia2), 25.5 (dia2), 25.4 (dia1), 22.4 (d, *J* = 1.8 Hz, dia1), 22.4 (d, *J* =

1.9 Hz, dia2), 16.6 (d, *J* = 6.3 Hz, dia2), 16.5 (d, *J* = 5.9 Hz, dia1); **^{31}P NMR (162 MHz, CDCl$_3$):** δ 33.9, 32.6; **IR (neat) ν_{max}:** 2932, 1640, 1374, 1250, 1215, 1164, 1072, 1019, 957, 842, 636; **HRMS (ESI+):** exact mass calculated for $[M+Na]^+$ ($C_{22}H_{39}NaO_8P$) requires *m/z* 485.2275, found *m/z* 485.2274.

Allyl ethyl but-3-en-1-ylphosphonate (2h)

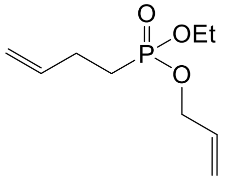

Prepared according to general procedure C; (tetrabutylammonium chloride was used instead of tetraethylammonium; extraction with dichloromethane); 60% yield; **^{1}H NMR (600 MHz, CDCl$_3$)**: δ 5.94 (ddd, *J* = 22.5, 10.7, 5.5 Hz, 1H), 5.85 (ddt, *J* = 16.7, 10.2, 6.3 Hz, 1H), 5.36 (dd, *J* = 17.1, 1.3 Hz, 1H), 5.24 (dd, *J* = 10.4, 0.9 Hz, 1H), 5.07 (dd, *J* = 17.1, 1.4 Hz, 1H), 5.01 (dd, *J* = 10.2, 1.0 Hz, 1H), 4.55 – 4.51 (m, 2H), 4.17 – 4.05 (m, 2H), 2.40 – 2.32 (m, 2H), 1.93 – 1.78 (m, 2H), 1.32 (t, *J* = 7.1 Hz,3H); **^{13}C NMR (150 MHz, CDCl$_3$):** δ 137.1 (d, *J* = 18.0 Hz), 133.1 (d, *J* = 6.1 Hz), 117.8, 115.2, 65.9 (d, *J* = 6.1 Hz), 61.6 (d, *J* = 6.6 Hz), 26.5 (d, *J* = 4.5 Hz), 25.2 (d, *J* = 140.8 Hz), 16.5 (d, *J* = 6.1 Hz); **^{31}P NMR (162 MHz, CDCl$_3$):** δ 31.9; **IR (neat) ν_{max}:** 2982. 2928, 1644, 1445, 1245, 1217, 1100, 1048, 1020, 963, 922, 846, 824, 752; **HRMS (ESI+):** exact mass calculated for $[M+Na]^+$ ($C_9H_{17}NaO_3P$) requires *m/z* 227.0813, found *m/z* 227.0808.

Ethyl ((*R*)-1-phenylethyl) but-3-en-1-ylphosphonate (2i)

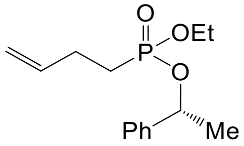

Prepared according to general procedure C; (tetrabutylammonium chloride was used instead of tetraethylammonium; extraction with dichloromethane); 40% yield, d.r. 1.15:1; **^{1}H NMR (600 MHz, CDCl$_3$)**: δ 7.41 – 7.33 (m, 4H), 7.33 – 7.27 (m, 1H), 5.84 (ddt, *J* = 16.7, 10.2, 6.3 Hz, 1H, dia1), 5.73 – 5.65 (m, 1H, dia2), 5.60 – 5.50 (m, 1H), 5.05 (dd, *J* = 17.1, 1.5 Hz, 1H, dia1), 4.99 (dd, *J* = 10.2, 1.3 Hz, 1H, dia2), 4.93 – 4.88 (m, 2H), 4.18 – 4.10 (m, 1H, dia2), 4.10 – 4.02 (m, 1H, dia1), 3.90 – 3.83 (m, 1H, dia2), 3.71 – 3.63 (m, 1H, dia1), 2.39 – 2.31 (m, 2H, dia1), 2.20 – 2.13 (m, 2H, dia2), 1.89 – 1.76 (m, 2H), 1.63-1.59 (4H), 1.60 (d, *J* = 6.5 Hz, 3H, dia1), 1.32 (t, *J* = 7.1 Hz, 3H, dia1), 1.08 (t, J = 7.1 Hz, 3H, dia2); **^{13}C NMR (150 MHz, CDCl$_3$):** δ 142.2 (d, *J* = 3.5 Hz), 142.0 (d, *J* = 4.2 Hz), 137.3 (d, *J* = 7.8 Hz), 137.2 (d, *J* = 8.2 Hz), 128.5 (s, 2C), 128.5 (s, 2C), 128.1, 128.0, 125.9, 115.1, 114.9, 74.8 (d, *J* = 5.9 Hz), 74.3 (d, *J* = 6.7 Hz), 61.3 (d, *J* = 6.7 Hz), 61.1

(d, J = 6.7 Hz), 26.5 (d, J = 4.5 Hz), 26.4 (d, J = 4.5 Hz), 25.6 (d, J = 142.3 Hz), 25.5 (d, J = 139.9 Hz), 24.7 (d, J = 6.0 Hz), 24.6 (d, J = 4.8 Hz), 16.4 (d, J = 6.4 Hz), 16.2 (d, J = 6.3 Hz); **^{31}P NMR (162 MHz, $CDCl_3$):** δ 31.1, 30.6; **IR (neat) ν_{max}:** 2980, 2930, 1642, 1449, 1374, 1283, 1243, 1212, 1173, 1041, 1008, 995, 960, 914, 824, 755, 699; **HRMS (ESI+):** exact mass calculated for $[M+Na]^+$ ($C_{14}H_{21}NaO_3P$) requires m/z 291.1126, found m/z 291.1123.

Ethyl prop-2-yn-1-yl (5-(1,3-dioxoisoindolin-2-yl)pentyl)phosphonate (2j)

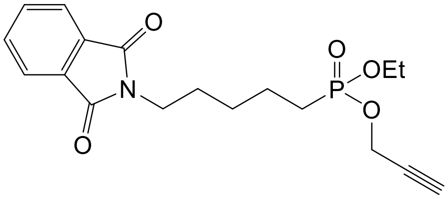

Prepared according to general procedure C; 45% yield; **^{1}H NMR (400 MHz, $CDCl_3$):** δ 7.85 – 7.78 (m, 2H), 7.72 – 7.65 (m, 2H), 4.63 (dd, J = 10.5, 2.4 Hz, 2H), 4.16 – 4.01 (m, 2H), 3.66 (t, J = 7.2 Hz, 2H), 2.53 (t, J = 2.4 Hz, 1H), 1.82 – 1.71 (m, 2H), 1.71 – 1.58 (m, 4H), 1.48 – 1.37 (m, 2H), 1.29 (t, J = 7.1 Hz, 3H); **^{13}C NMR (100 MHz, $CDCl_3$):** δ 168.5 (2C), 134.0 (2C), 132.2 (2C), 123.3 (2C), 75.5 (d, J = 2.0 Hz), 61.8 (d, J = 6.8 Hz), 53.1 (d, J = 5.7 Hz), 37.8, 28.2, 27.8 (d, J = 17.2 Hz), 25.9 (d, J = 140.3 Hz), 22.0 (d, J = 5.2 Hz), 16.4 (d, J = 6.2 Hz); **^{31}P NMR (162 MHz, $CDCl_3$):** δ 33.4; **IR (neat) ν_{max}:** 2939, 1770, 1705, 1439, 1396, 1368, 1232, 1019, 995, 961, 718; **HRMS (ESI+):** exact mass calculated for $[M+Na]^+$ ($C_{18}H_{22}NNaO_5P$) requires m/z 386.1128, found m/z 386.1127.

Ethyl 6-(ethoxy(prop-2-yn-1-yloxy)phosphoryl)hexanoate (2k)

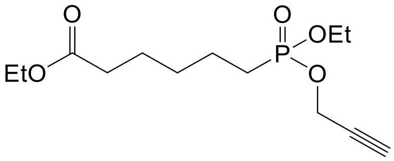

Prepared according to general procedure C (tetrabutylammonium chloride was used instead of tetraethylammonium; extraction with dichloromethane); 75% yield; **^{1}H NMR (600 MHz, $CDCl_3$)**: δ 4.65 (ddd, J = 10.6, 2.4, 0.8 Hz, 2H, H11), 4.18 – 4.04 (m, 4H, H7,9), 2.54 (t, J = 2.4 Hz, 1H, H13), 2.29 (t, J = 7.5 Hz, 2H, H5), 1.82 – 1.74 (m, 2H, H1), 1.67 – 1.58 (m, 4H, H2,4), 1.45 – 1.36 (m, 2H, H3), 1.32 (t, J = 7.1 Hz, 3H, H10), 1.24 (t, J = 7.1 Hz, 3H, H8); **^{13}C NMR (150 MHz, $CDCl_3$):** δ 173.5, 75.4, 61.7 (d, J = 6.9 Hz), 60.3, 53.0 (d, J = 5.7 Hz), 34.0, 29.9 (d, J = 17.5 Hz), 25.7 (d, J = 140.2 Hz), 24.3, 22.0 (d, J = 5.2 Hz), 16.3 (d, J = 6.3 Hz), 14.2; **^{31}P NMR (243 MHz, $CDCl_3$):** δ 33.7; **IR (neat) ν_{max}:** 2982, 2937, 1730, 1450, 1373, 1328, 1122,

1097, 1052, 996, 962, 829, 749, 704; **HRMS (ESI+):** exact mass calculated for $[M+Na]^+$ ($C_{13}H_{23}NaO_5P$) requires *m/z* 313.1181, found *m/z* 313.1177.

Ethyl prop-2-yn-1-yl dec-9-yn-1-ylphosphonate (2l)

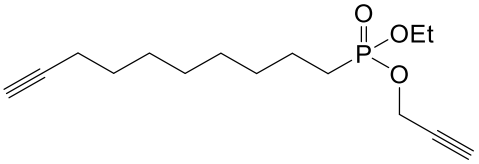

Prepared according to general procedure C; 60% yield; **^{1}H NMR (700 MHz, $CDCl_3$)**: δ 4.64-4.71 (m, 2H), 4.07-4.18 (m, 2H), 2.55 (t, *J* = 2.1 Hz, 1H), 2.17-2.20 (m, 2H), 1.95 (t, *J* = 2.1 Hz, 1H), 1.76-1.83 (m, 2H), 1.62-1.64 (m, 2H), 1.51-1.54 (m, 2H), 1.37-1.42 (m, 4H), 1.34 (t, *J* = 7.0 Hz, 3H), 1.28-1.32 (m, 4H); **^{13}C NMR (175 MHz, $CDCl_3$)**: δ 75.3, 68.1, 61.7 (d, *J* = 7 Hz), 52.9 (d, *J* = 7 Hz), 30. 5, 30.4, 28.9, 28.8, 28.6, 28.4, 26.3, 25.5, 22.2 (d, *J* = 5.3 Hz), 18.4, 16.4 (d, *J* = 2.1 Hz); **^{31}P NMR (243 MHz, $CDCl_3$):** δ 34.0; **IR (neat) ν_{max}:** 1459, 1371, 1235, 1164, 1097, 1053, 1024, 995, 963, 836, 705, 636; **HRMS (ESI+):** exact mass calculated for $[M+Na]^+$ ($C_{15}H_{25}NaO_3P$) requires *m/z* 307.1434, found *m/z* 307.1427.

Ethyl prop-2-yn-1-yl (5-cyanopentyl)phosphonate (2m)

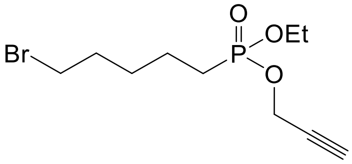

Prepared according to general procedure C; 69% yield; **^{1}H NMR (400 MHz, $CDCl_3$):** δ 4.66 (dd, *J* = 10.5, 2.4 Hz, 2H), 4.19 – 4.05 (m, 2H), 3.40 (t, *J* = 6.7 Hz, 2H), 2.54 (t, *J* = 2.4 Hz, 1H), 1.91 – 1.75 (m, 4H), 1.73 – 1.59 (m, 2H), 1.58 – 1.50 (m, 2H), 1.33 (t, *J* = 7.1 Hz, 3H); **^{13}C NMR (100 MHz, $CDCl_3$):** δ 75.5 (d, *J* = 2.1 Hz), 61.9 (d, *J* = 6.8 Hz), 53.2 (d, *J* = 5.7 Hz), 33.4, 32.3 (d, *J* = 1.1 Hz), 29.1 (d, *J* = 17.1 Hz), 25.9 (d, *J* = 140.5 Hz), 21.7 (d, *J* = 5.2 Hz), 16.5 (d, *J* = 6.2 Hz); **^{31}P NMR (162 MHz, $CDCl_3$):** δ 33.3; **IR (neat) ν_{max}:** 2938, 1639, 1446, 1371, 1241, 1224, 1050, 1017, 994, 960, 839, 815, 704, 639; **HRMS (ESI+):** exact mass calculated for $[M+Na]^+$ ($C_{10}H_{18}BrNaO_3P$) requires *m/z* 319.0069, found *m/z* 319.0068.

Ethyl prop-2-yn-1-yl (5-cyanopentyl)phosphonate (2n)

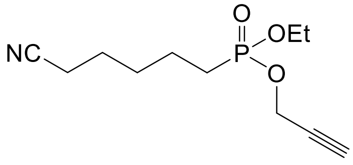

Prepared according to general procedure C; 49% yield; **^{1}H NMR (400 MHz, $CDCl_3$):** δ 4.65 (dd, *J* = 10.6, 2.5 Hz, 2H), 4.18 – 4.03 (m, 2H), 2.54 (t, *J* = 2.4 Hz, 1H), 2.34 (t, *J* = 7.0 Hz, 2H), 1.85 – 1.73 (m, 2H), 1.72 – 1.59 (m, 4H), 1.58 – 1.51 (m, 2H), 1.32 (t, *J* = 7.1 Hz, 3H); **^{13}C NMR (100 MHz, $CDCl_3$):** δ 119.5, 75.6 (d, *J* = 1.9 Hz), 61. 9 (d, *J* = 6.9 Hz), 53.2 (d, *J* = 5.8 Hz), 29.4 (d, *J* = 16.7 Hz), 25.7 (d, *J* = 143.0 Hz), 25.0 (d, *J* = 2.2 Hz), 21.7 (d, *J* = 5.2 Hz), 17.1, 16.4 (d, *J* = 6.2 Hz); **^{31}P NMR (162 MHz, $CDCl_3$):** δ 33.0; **IR (neat) ν_{max}:** 2939, 2246, 1657, 1450, 1220, 1018, 962, 826; **HRMS (ESI+):** exact mass calculated for $[M+Na]^+$ ($C_{11}H_{18}NNaO_3P$) requires *m/z* 266.0917, found *m/z* 266.0913.

Ethyl prop-2-yn-1-yl (*E*)-styrylphosphonate (2o)

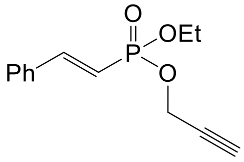

Prepared according to general procedure C; 56% yield; **^{1}H NMR (400 MHz, $CDCl_3$)**: δ 7.55 (d, *J* = 17.5 Hz, 1H), 7.52 – 7.46 (m, 2H), 7.41 – 7.33 (m, 3H), 6.26 (dd, *J* = 18.3, 17.6 Hz, 1H), 4.75 – 4.61 (m, 2H), 4.22 – 4.12 (m, 2H), 2.53 (t, *J* = 2.5 Hz, 1H), 1.36 (t, *J* = 7.1 Hz, 3H); **^{13}C NMR (100 MHz, $CDCl_3$):** δ 149.4 (d, *J* = 6.8 Hz), 134.8 (d, *J* = 23.6 Hz), 130.5, 129.0, 127.9, 113.4 (d, *J* = 193.1 Hz), 75.7, 62.4 (d, *J* = 5.5 Hz), 53.3 (d, *J* = 4.2 Hz), 16.4 (d, *J* = 6.5 Hz); **^{31}P NMR (162 MHz, $CDCl_3$):** δ 20.7; **IR (neat) ν_{max}:** 3291, 3207, 3027, 2982, 1615, 1576, 1522, 1449, 1392, 1371, 1341, 1240, 1046, 1016, 992, 951, 860, 829, 802, 741, 688; **HRMS (ESI+):** exact mass calculated for $[M+Na]^+$ ($C_{13}H_{15}NaO_3P$) requires *m/z* 273.0651, found *m/z* 273.0647.

Ethyl prop-2-yn-1-yl phenylphosphonate (2p)

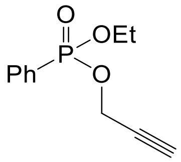

Prepared according to general procedure C; 77% yield; **^{1}H NMR (400 MHz, $CDCl_3$)**: δ 7.78 – 7.65 (m, 2H), 7.50 – 7.40 (m, 1H), 7.39 – 7.31 (m, 2H), 4.58 (dddd, *J* = 43.5, 15.5, 9.5, 2.5 Hz, 2H), 4.13 – 3.99 (m, 2H), 2.38 (t, *J* = 2.5 Hz, 1H), 1.30 – 1.19 (m, 3H); **^{13}C NMR (100 MHz, $CDCl_3$):** δ 132.8 (d, *J* = 3.1 Hz), 131.9 (d, *J* =

10.1 Hz), 128.6 (d, J = 15.3 Hz), 126.8, 75.7 (d, J = 2.2 Hz), 62.6 (d, J = 5.8 Hz), 53.5 (d, J = 4.4 Hz), 16.4 (d, J = 6.5 Hz); **^{31}P NMR (162 MHz, $CDCl_3$):** δ 19.8; **IR (neat) ν_{max}:** 3294, 3213, 2983, 1652, 1594, 1440, 1238, 1131, 1047, 1015, 992, 949, 849, 818, 775, 748, 693; **HRMS (ESI+):** exact mass calculated for $[M+Na]^+$ ($C_{11}H_{13}NaO_3P$) requires *m/z* 247.0495, found *m/z* 247.0496.

Ethyl prop-2-yn-1-yl (phenylethynyl)phosphonate (2q)

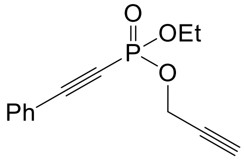

Prepared according to general procedure C; 59% yield; **^{1}H NMR (400 MHz, $CDCl_3$)**: 7.62 – 7.53 (m, 2H), 7.51 – 7.42 (m, 1H), 7.42 – 7.33 (m, 2H), 4.80 – 4.70 (m, 2H), 4.26 (dq, J = 8.7, 7.1 Hz, 2H), 2.58 (t, J = 2.5 Hz, 1H), 1.42 (td, J = 7.1, 0.7 Hz, 3H); **^{13}C NMR (100 MHz, $CDCl_3$):** δ 132.7 (d, J = 1.8 Hz), 130.9, 128.6, 119.3 (d, J = 5.7 Hz), 99.9 (d, J = 54.5 Hz), 79.2, 76.0, 63.7 (d, J = 5.4 Hz), 54.2 (d, J = 3.8 Hz), 16.1 (d, J = 6.9 Hz); **^{31}P NMR (162 MHz, $CDCl_3$):** δ -5.6; **IR (neat) ν_{max}:** 3294, 3216, 2985, 2932, 2185, 2126, 1490, 1444, 1259, 1163, 1016, 990, 860, 757, 687; **HRMS (ESI+):** exact mass calculated for $[M+Na]^+$ ($C_{13}H_{13}NaO_3P$) requires *m/z* 271.0495, found *m/z* 271.0492.

Allyl ethyl ethylphosphonate (2r)

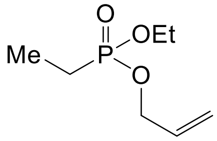

Prepared according to general procedure C on 15 mmol scale; 82% yield; **^{1}H NMR (600 MHz, $CDCl_3$):** δ 5.98 – 5.90 (m, 1H), 5.36 (ddd, J = 17.1, 3.1, 1.5 Hz, 1H), 5.24 (ddd, J = 10.4, 2.5, 1.2 Hz, 1H), 4.57 – 4.50 (m, 2H), 4.17 – 4.05 (m, 2H), 1.83 – 1.73 (m, 2H), 1.32 (t, J = 7.1 Hz, 3H), 1.17 (dt, J = 20.2, 7.7 Hz, 3H); **^{13}C NMR (150 MHz, $CDCl_3$):** δ 133.3 (d, J = 6.2 Hz), 117.9, 66.2 (d, J = 6.0 Hz), 61.9 (d, J = 6.3 Hz), 19.0 (d, J = 142.8 Hz), 16.6 (d, J = 6.1 Hz), 6.7 (d, J = 6.9 Hz); **^{31}P NMR (243 MHz, $CDCl_3$):** δ 33.9; **IR (neat) ν_{max}:** 2984, 2945, 1651, 1460, 1410, 1247, 1220, 1161, 1099, 1008, 962, 922, 851, 794, 732, 638; **HRMS (ESI+):** exact mass calculated for $[M+Na]^+$ ($C_7H_{15}NaO_3P$) requires *m/z* 201.0651, found *m/z* 201.0652.

Ethyl 6-(ethoxy(phenoxy)phosphoryl)hexanoate (2s)

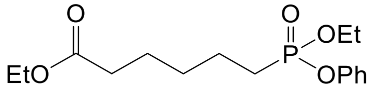

Prepared according to general procedure C on 2 mmol scale; 86% yield; **^{1}H NMR (400 MHz, $CDCl_3$):** δ 7.31 (t, *J* = 7.8 Hz, 2H), 7.21 – 7.16 (m, 2H), 7.14 (t, *J* = 7.4 Hz, 1H), 4.23 – 4.15 (m, 1H), 4.15 – 4.07 (m, 3H), 2.28 (t, *J* = 7.4 Hz, 2H), 1.94 – 1.82 (m, 2H), 1.76 – 1.59 (m, 4H), 1.48 – 1.38 (m, 2H), 1.28 (t, *J* = 7.1 Hz, 3H), 1.23 (td, *J* = 7.1, 0.8 Hz, 3H); **^{13}C NMR (100 MHz, $CDCl_3$):** δ 173.6, 150.8 (d, *J* = 8.4 Hz), 129.8 (2C), 124.9, 120.6 (d, *J* = 4.3 Hz, 2C), 62.4 (d, *J* = 7.0 Hz), 60.4, 34.1, 30.0 (d, *J* = 17.1 Hz), 25.7 (d, *J* = 141.0 Hz), 24.5, 22.2 (d, *J* = 5.3 Hz), 16.5 (d, *J* = 5.9 Hz), 14.3; **^{31}P NMR (162 MHz, $CDCl_3$):** δ 29.1; **IR (neat) ν_{max}:** 2935, 1730, 1592, 1490, 1251, 1204, 1162, 1025, 918, 762, 729, 690; **HRMS (ESI+):** exact mass calculated for $[M+Na]^+$ ($C_{16}H_{25}NaO_5P$) requires *m/z* 351.1332, found *m/z* 351.1325.

Ethyl 6-((prop-2-yn-1-yloxy)((triisopropylsilyl)ethynyl)phosphoryl)hexanoate (6b)

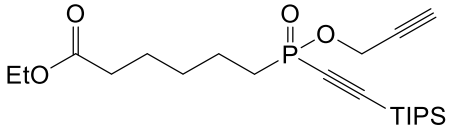

Prepared according to general procedure C; 52% yield; **^{1}H NMR (400 MHz, $CDCl_3$)**: δ 4.70 (dd, *J* = 9.8, 2.5 Hz, 2H), 4.11 (q, *J* = 7.1 Hz, 2H), 2.53 (t, *J* = 2.5 Hz, 1H), 2.28 (t, *J* = 7.5 Hz, 2H), 1.95 – 1.85 (m, 2H), 1.76 – 1.58 (m, 4H), 1.49 – 1.40 (m, 2H), 1.24 (t, *J* = 7.1 Hz, 3H), 1.14 – 1.07 (m, 21H); **^{13}C NMR (100 MHz, $CDCl_3$)**: δ 173.6, 109.1 (d, *J* = 24.1 Hz), 98.7 (d, *J* = 175.3 Hz), 75.9 (d, *J* = 2.4 Hz), 60.4, 52.9 (d, *J* = 6.3 Hz), 34.1, 30.9 (d, *J* = 116.5 Hz), 29.9 (d, *J* = 17.3 Hz), 24.6, 21.6 (d, *J* = 4.1 Hz), 18.6 (6C), 14.4, 11.0 (3C); **^{31}P NMR (162 MHz, $CDCl_3$):** δ 23.0; **IR (neat) ν_{max}:** 3212, 2943, 2866, 2124, 1732, 1462, 1371, 1235, 1192, 1027, 993, 794, 764, 713; **HRMS (ESI+):** exact mass calculated for $[M+Na]^+$ ($C_{22}H_{39}NaO_4PSi$) requires *m/z* 447.2247, found *m/z* 447.2244.

Ethyl (3-phenoxybenzyl) but-3-en-1-ylphosphonate (7)

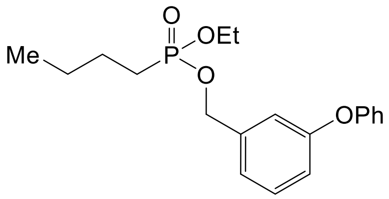

Prepared according to general procedure C; 55% yield; **^{1}H NMR (400 MHz, CDCl$_3$)**: δ 7.38 – 7.28 (m, 3H), 7.15 – 7.07 (m, 2H), 7.04 – 6.93 (m, 4H), 5.02 (d, *J* = 8.2 Hz, 2H), 4.14 – 3.95 (m, 2H), 1.78 – 1.67 (m, 2H), 1.61 – 1.49 (m, 2H), 1.42 – 1.32 (m, 2H), 1.27 (t, *J* = 7.1 Hz, 3H), 0.88 (t, *J* = 7.3 Hz, 3H); **^{31}P NMR (162 MHz, CDCl$_3$):** δ 33.3; All analytical data were in good accordance with data reported in the literature.[11]

5.3. Thiols as nucleophiles: preparation of phosphonothioates

***O*-Ethyl *S*-octyl phenethylphosphonothioate (3a)**

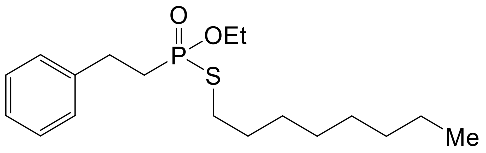

Prepared according to general procedure C; 80% yield; **^{1}H NMR (600 MHz, CDCl$_3$)**: δ 7.28-7.31 (m, 2H), 7.20-7.22 (m, 3H), 4.17-4.24 (m, 1H), 4.08-4.15 (m, 1H), 2.95-3.01 (m, 2H), 2.81-2.90 (m, 2H), 2.24-2.31 (m, 2H), 1.65-1.70 (m, 2H), 1.35-1.42 (m, 2H), 1.23-1.32 (m, 9H), 1.34 (t, *J* = 6.0 Hz, 3H), 0.87 (t, *J* = 6.0, 3H); **^{13}C NMR (150 MHz, CDCl$_3$):** δ 140.7 (d, *J* = 2.0 Hz), 128.6, 128, 126.4, 61.2 (d, *J* = 5.0 Hz), 35.7, 35.0, 31.8, 31.4 (d, *J* = 3.0 Hz), 29.2, 29.0, 28.7, 28.4 (d, *J* = 3.0 Hz), 22.6, 16.3 (d, *J* = 5.0 Hz), 14.1; **^{31}P NMR (243 MHz, CDCl$_3$):** δ 56.1; . **IR (neat) ν_{max}:** 1455, 1231, 1033, 953, 761, 743, 698; **HRMS (ESI+):** exact mass calculated for $[M+Na]^+$ ($C_{18}H_{31}NaO_2PS$) requires *m*/*z* 365.1675, found *m*/*z* 365.1675.

***S*-Benzyl *O*-ethyl but-3-en-1-ylphosphonothioate (3b)**

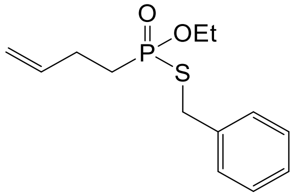

Prepared according to general procedure C; (tetrabutylammonium chloride was used instead of tetraethylammonium; extraction with dichloromethane); 63% yield; **^{1}H NMR (400 MHz, $CDCl_3$)**: δ 7.33 – 7.28 (m, 2H), 7.28 – 7.22 (m, 2H), 7.22 – 7.16 (m, 1H), 5.69 (ddt, *J* = 16.7, 10.2, 6.4 Hz, 1H), 4.96 – 4.91 (m, 1H), 4.91 – 4.87 (m, 1H), 4.09 (ddq, *J* = 14.1, 10.0, 7.1 Hz, 1H), 3.99 (dd, *J* = 12.2, 2.8 Hz, 2H), 3.97 – 3.89 (m, 1H), 2.31 – 2.18 (m, 2H), 1.87 – 1.74 (m, 2H), 1.23 (t, *J* = 7.1 Hz, 3H); **^{13}C NMR (100 MHz, $CDCl_3$):** δ 138.1 (d, *J* = 3.7 Hz), 136.8 (d, *J* = 18.8 Hz), 128.9 (2C), 128.7 (2C), 127.5, 115.3, 61.4 (d, *J* = 7.4 Hz, 34.5 (d, *J* = 2.8 Hz), 32.8 (d, *J* = 106.0 Hz), 26.2 (d, *J* = 3.9 Hz), 16.2 (d, *J* = 6.8 Hz); **^{31}P NMR (162 MHz, $CDCl_3$):** δ 55.8; **IR (neat) $ν_{max}$:** 2979, 2926, 1641, 1495, 1391, 1227, 1160, 1907, 1080, 1012, 952, 914, 793, 761, 699; **HRMS (ESI+):** exact mass calculated for $[M+Na]^+$ ($C_{13}H_{19}NaO_2PS$) requires *m/z* 293.0741, found *m/z* 293.0740.

S-Cyclohexyl O-ethyl phenethylphosphonothioate (3c)

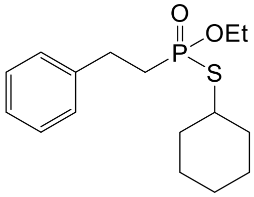

Prepared according to general procedure C; 55% yield; **^{1}H NMR (400 MHz, $CDCl_3$)**: δ 7.28-7.34 (m, 2H), 7.21-7.24 (m, 3H), 4.08-4.27 (m, 2H), 3.31-3.41 (m, 1H), 2.97-3.03 (m, 2H), 2.24-2.32 (m, 2H), 2.06-2.13 (m, 2H), 1.75-1.78 (m, 1H), 1.52-1.62 (m, 3H), 1.26-1.47 (m, 2H), 1.35 (t, *J* = 8.0 Hz, 3H); **^{13}C NMR (100 MHz, $CDCl_3$):** δ 140.9 (d, *J* = 19 Hz), 128.6, 128.1, 126.4, 61.2 (d, *J* = 7 Hz), 45.2 (d, *J* = 3 Hz), 36.7, 36.1 (d, *J* = 4.0 Hz), 35.8 (d, *J* = 4.0 Hz), 35.7, 28.5 (d, *J* = 4.0 Hz), 26.0, 25.3, 16.2 (d, *J* = 7.0 Hz); **^{31}P NMR (243 MHz, $CDCl_3$):** δ 55.2; **IR (neat) $ν_{max}$:** 1450, 1265, 1231, 1033, 998, 954, 763, 748, 699, 599; **HRMS (ESI+):** exact mass calculated for $[M+Na]^+$ ($C_{16}H_{25}NaO_2PS$) requires *m/z* 335.1205, found *m/z* 335.1198.

Ethyl 6-((*tert*-butylthio)(ethoxy)phosphoryl)hexanoate (3d)

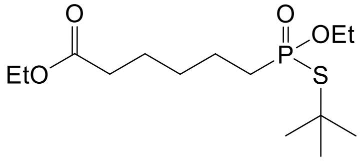

Prepared according to general procedure C; 61% yield; **^{1}H NMR (600 MHz, $CDCl_3$)**: δ 4.22 – 4.04 (m, 4H), 2.30 (t, *J* = 7.5 Hz, 2H), 1.98 – 1.87 (m, 2H), 1.72 – 1.60 (m, 4H), 1.56 (s, 9H), 1.47 – 1.38 (m, 2H), 1.31 (t, *J* = 7.1 Hz, 3H), 1.25 (t, *J* = 7.1 Hz, 3H); **^{13}C NMR (150 MHz, $CDCl_3$):** δ 173.7, 61.0 (d, *J* = 7.3 Hz), 60.4, 50.5, 35.5 (d, *J* = 105.8 Hz), 34.2, 33.2 (d, *J* = 4.5 Hz), 30.0 (d, *J* = 17.6 Hz), 24.6, 22.3 (d, *J* = 5.2 Hz), 16.4 (d, *J* =

6.9 Hz), 14.4; **^{31}P NMR (243 MHz, $CDCl_3$):** δ 55.2; **IR (neat) ν_{max}:** 2934, 2868, 1734, 1460, 1367, 1232, 1195, 1162, 1029, 952; **HRMS (ESI+):** exact mass calculated for $[M+Na]^+$ ($C_{14}H_{29}NaO_4PS$) requires *m/z* 347.1416, found *m/z* 347.1409.

***S*-(4-Chlorophenyl) *O*-ethyl oct-7-en-1-ylphosphonothioate (3e)**

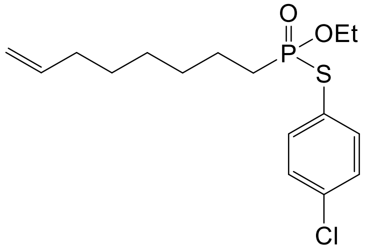

Prepared according to general procedure C; 71% yield; **^{1}H NMR (600 MHz, $CDCl_3$)**: δ 7.49-7.52 (m, 2H), 7.34-7.36 (m, 2H), 5.77-5.84 (m,. 1H), 4.94-5.03 (m, 2H), 4.26-4.35 (m, 1H), 4.13-4.22 (m, 1H), 2.03-2.07 (m, 2H), 1.86-1.91 (m, 2H), 1.63-1.71 (m, 1H), 1.34-1.45 (m, 8H), 1.27-1.31 (m, 2H); **^{13}C NMR (150 MHz, $CDCl_3$):** δ 138.9, 136.2 (d, *J* = 2.5Hz, 2C), 135.5 (d, *J* = 2.5 Hz, 1C), 129.6 (d, *J* = 1.3 Hz, 2C), 126 (d, *J* = 5.0 Hz, 1C), 114.4, 61.8 (d, *J* = 6.3 Hz, 1C), 33.6, 32.0, 31.3, 30.2 (d, *J* = 13.8 Hz, 1C), 28.5 (d, *J* = 6.3 Hz, 1C), 22.1 (d, *J* = 3.8 Hz, 1C), 16.3 (d, *J* = 5.0 Hz, 1C); **^{31}P NMR (162 MHz, $CDCl_3$):** δ 55.1; **IR (neat) ν_{max}:** 1640, 1475, 1391, 1236, 1162, 1092, 1028, 1013, 956, 911, 821, 745; **HRMS (ESI+):** exact mass calculated for $[M+H]^+$ ($C_{16}H_{25}ClO_2PS$) requires *m/z* 347.0996, found *m/z* 347.0989.

***O*-Ethyl *S*-(naphthalen-2-yl) phenethylphosphonothioate (3f)**

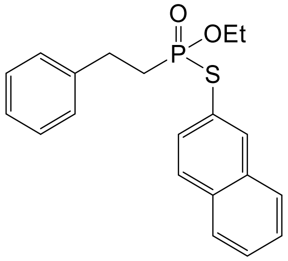

Prepared according to general procedure C; 61% yield; **^{1}H NMR (400 MHz, $CDCl_3$)**: δ 8.10 (s, 1H), 7.81-7.88 (m, 3H), 7.53-7.64 (m, 3H), 7.13-7.30 (m, 5H), 4.26-4.45 (m, 2H), 2.93-3.09 (m, 2H), 2.19-2.28 (m, 2H), 1.42 (t, *J* = 7.2 Hz, 3H); **^{13}C NMR (150 MHz, $CDCl_3$):** δ **^{13}C NMR (100 MHz, $CDCl_3$):** δ 140.5 (d , *J* = 9.0 Hz), 135.0 (d, *J* = 5.0 Hz), 133.7 (d, *J* = 2.0 Hz), 133.1 (d, *J* = 1 Hz), 131.4 (d, *J* = 3.0 Hz), 129.2 (d, *J* = 1.0 Hz), 128.6, 128.1, 127.8 (d, *J* = 1.0 Hz), 127.7 (d, *J* = 1Hz), 127.2 (d, *J* = 1.0 Hz), 126.9, 126.4, 124.3 (d, *J* = 6.0 Hz), 61.9 (d, *J* = 8.0 Hz), 33.8, 32.8, 28.3 (d, *J* = 4.0 Hz), 16.3, 16.2; **^{31}P NMR (162 MHz, $CDCl_3$):** δ 54.1; **IR (neat) ν_{max}:**

1498, 1454, 1267, 1231, 1030, 958, 941, 860, 958, 941, 699; **HRMS (ESI+):** exact mass calculated for $[M+H]^+$ ($C_{20}H_{22}O_2PS$) requires *m/z* 357.1073, found *m/z* 357.1066.

***O,S*-Diethyl (3-bromopropyl)phosphonothioate (3g)**

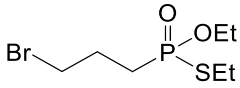

Prepared according to general procedure C; 75% yield; **^{1}H NMR (400 MHz, $CDCl_3$)**: δ 4.27 – 4.04 (m, 2H), 3.50 (t, *J* = 6.3 Hz, 2H), 2.96 – 2.81 (m, 2H), 2.29 – 2.18 (m, 2H), 2.17 – 2.07 (m, 2H), 1.38 (t, *J* = 6.9 Hz, 2H), 1.34 (t, J = 6.5 Hz, 2H); **^{13}C NMR (100 MHz, $CDCl_3$):** δ 61.5 (d, *J* = 7.2 Hz), 33.1 (d, *J* = 55.8 Hz), 32.7 (d, *J* = 183.4 Hz), 25.9 (d, *J* = 3.5 Hz), 25.2 (d, *J* = 3.2 Hz), 17.0 (d, *J* = 4.7 Hz), 16.4 (d, *J* = 6.9 Hz); **^{31}P NMR (243 MHz, $CDCl_3$):** δ 55.6; **IR (neat) ν_{max}:** 2955, 2923, 2852, 1659, 1633, 1467, 1264, 1213, 1160, 1030, 959, 751, 639, 590; **HRMS (ESI+):** exact mass calculated for $[M+Na]^+$ ($C_7H_{16}NaO_2PSBr$) requires *m/z* 298.9669, found *m/z* 298.9661.

***O,S*-Diethyl (3-(4-phenylpiperazin-1-yl)propyl)phosphonothioate (9)**

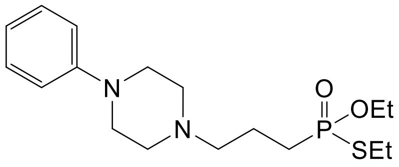

Prepared according the method reported by Lewkowski *et al.*;[10] 88% yield; **^{1}H NMR (400 MHz, $CDCl_3$)**: δ 7.30 – 7.21 (m, 3H), 6.93 (dd, J = 8.8, 1.0 Hz, 2H), 6.90 – 6.81 (m, 1H), 4.29 – 4.03 (m, 2H), 3.29 – 3.12 (m, 4H), 2.96 – 2.79 (m, 2H), 2.61 (dd, J = 5.8, 4.2 Hz, 4H), 2.48 (t, J = 7.1 Hz, 2H), 2.10 – 1.99 (m, 2H), 1.96 – 1.81 (m, 2H), 1.61 (d, J = 6.7 Hz, 2H), 1.43 – 1.29 (m, 6H); **^{13}C NMR (100 MHz, $CDCl_3$):** δ 151.5, 129.3, 119.8, 116.2, 61.3 (d, *J* = 7.3 Hz), 58.4 (d, *J* = 18.4 Hz), 53.2, 49.3, 31.4 (d, *J* = 107.9 Hz), 25.1 (d, *J* = 3.1 Hz), 19.9 (d, *J* = 4.0 Hz), 17.1 (d, *J* = 5 Hz), 16.4 (d, *J* = 7 Hz); **^{31}P NMR (243 MHz, $CDCl_3$):** δ 57.4; **IR (neat) ν_{max}:** 2930, 2818, 2358, 1718, 1599, 1578, 1498, 1453, 1379, 1353, 1306, 1265, 1233, 1158, 1139, 1101, 1025, 954, 758, 693, 647, 582, 527, 462, 435, 416; **HRMS (ESI+):** exact mass calculated for $[M+Na]^+$ ($C_{17}H_{29}NaN_2O_2PS$) requires *m/z* 379.1585, found *m/z* 379.1565.

5.4. Amines and amides as nucleophiles: preparation of phosphonamidates

Ethyl *N*-methyl-*N*-((4-nitrophenyl)sulfonyl)-*P*-(oct-7-en-1-yl)phosphonamidate (4a)

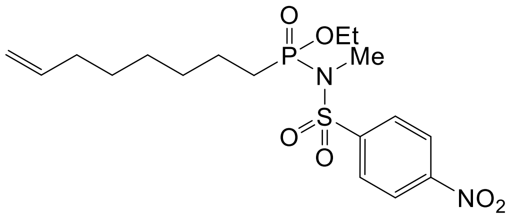

Prepared according to general procedure C; 72% yield; **^{1}H NMR (600 MHz, $CDCl_3$)**: δ 8.38 (d, *J* = 8.9 Hz, 2H), 8.19 (d, *J* = 8.9 Hz, 2H), 5.80 (ddt, *J* = 16.9, 10.2, 6.7 Hz, 1H), 5.03 – 4.91 (m, 2H), 4.15 – 4.05 (m, 1H), 3.85 – 3.76 (m, 1H), 3.08 (d, *J* = 6.8 Hz, 3H), 2.19 – 2.11 (m, 2H), 2.07 – 2.01 (m, 2H), 1.69 – 1.58 (m, 2H), 1.46 – 1.37 (m, 4H), 1.35 – 1.31 (m, 2H), 1.29 (t, *J* = 7.1 Hz, 3H); **^{13}C NMR (150 MHz, $CDCl_3$):** δ 150.6, 144.8, 139.0, 129.3, 124.4, 114.6, 62.3 (d, *J* = 7.0 Hz), 33.9, 33.8, 30.4 (d, *J* = 18.4 Hz), 29.3 (d, *J* = 128.7 Hz), 28.7, 28.7, 22.0 (d, *J* = 4.7 Hz), 16.3 (d, *J* = 6.3 Hz); **^{31}P NMR (243 MHz, $CDCl_3$):** δ 33.4; **IR (neat) ν_{max}:** 3106, 3072, 2978, 2929, 2856, 1640, 1607, 1530, 1462, 1350, 1311, 1245, 1168, 1090, 1025, 964, 868, 849, 784, 686, 647; **HRMS (ESI+):** exact mass calculated for $[M+Na]^+$ ($C_{17}H_{27}N_2NaO_6PS$) requires *m/z* 441.1220, found *m/z* 441.1219.

Ethyl *N*-methyl-*P*-(oct-7-en-1-yl)phosphonamidate (10)

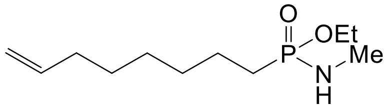

To a solution of the nosyl protected phosphonamidate **4a** (0.03 mmol) in DMF (0.3 mL) was added dry potassium carbonate (3 eq.) and the thiophenol (2 eq.). After stirring 2 hours, the mixture was diluted with a saturated solution of ammonium chloride and ethyl acetate. After separation of the phases, the aqueous phase was extracted three times with ethyl acetate. The organic phases were combined and dried on magnesium sulfate. After filtration and evaporation, the crude was then purified by flash column chromatography on silica gel (dichloromethane/methanol); 75% yield; **^{1}H NMR (600 MHz, $CDCl_3$)**: δ 5.79 (ddt, *J* = 16.9, 10.2, 6.7 Hz, 1H), 5.03 – 4.88 (m, 2H), 4.11 – 4.01 (m, 1H), 4.01 – 3.91 (m, 1H), 2.59 (dd, *J* = 11.0, 4.6 Hz, 3H), 2.33 (br. s, 1H), 2.08 – 1.98 (m, 2H), 1.74 – 1.63 (m, 2H), 1.62 – 1.53 (m, 2H), 1.43 – 1.34 (m, 4H), 1.34 – 1.26 (m, 5H); **^{13}C NMR (150 MHz, $CDCl_3$):** δ 139.1, 114.4, 59.4 (d, *J* = 6.6 Hz), 33.8, 30.8, 30.7, 28.8, 27.4, 26.2 (d, *J* = 129.8 Hz), 22.4 (d, *J* = 4.1 Hz), 16.6 (d, *J* = 6.4 Hz); **^{31}P NMR (243 MHz, $CDCl_3$):** δ 36.8; **IR (neat) ν_{max}:** 3335, 3236, 3077, 2977, 2928, 2856, 1641, 1441, 1276, 1205, 1114, 1042, 952, 910,

861, 804, 765, 750; **HRMS (ESI+):** exact mass calculated for $[M+Na]^+$ ($C_{11}H_{24}NNaO_2P$) requires *m/z* 256.1437, found *m/z* 256.1433.

Ethyl *N*-benzyl-*P*-(oct-7-en-1-yl)-*N*-tosylphosphonamidate (4b)

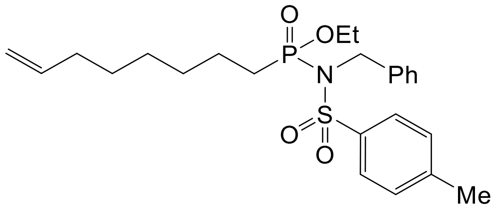

Prepared according to general procedure C; 65% yield; **^{1}H NMR (400 MHz, $CDCl_3$)**: δ 7.62 (d, *J* = 8.3 Hz, 2H), 7.45 – 7.37 (m, 2H), 7.26 – 7.21 (m, 3H), 7.18 (d, *J* = 8.1 Hz, 2H), 5.79 (ddt, *J* = 16.9, 10.2, 6.7 Hz, 1H), 5.02 – 4.91 (m, 2H), 4.72 (d, *J* = 9.3 Hz, 2H), 4.10 – 3.99 (m, 1H), 3.56 – 3.44 (m, 1H), 2.38 (s, 3H), 2.15 – 1.97 (m, 4H), 1.56 – 1.43 (m, 2H), 1.38 – 1.23 (m, 6H), 1.20 (t, *J* = 7.1 Hz, 3H); **^{13}C NMR (100 MHz, $CDCl_3$):** δ 144.1, 139.1, 137.7, 137.0, 129.4, 129.3, 128.3, 127.9, 127.8, 114.4, 61.9 (d, *J* = 7.0 Hz), 51.1, 33.8, 30.4 (d, *J* = 18.7 Hz), 30.1 (d, *J* = 128.5 Hz), 28.7, 28.6, 21.9 (d, *J* = 4.6 Hz), 21.6, 16.3 (d, *J* = 6.5 Hz); **^{31}P NMR (162 MHz, $CDCl_3$):** δ 34.3; **IR (neat) ν_{max}:** 2968, 2855, 1599, 1496, 1456, 1348, 1245, 1164, 1091, 1023, 998, 833; **HRMS (ESI+):** exact mass calculated for $[M+Na]^+$ ($C_{24}H_{34}NNaO_4PS$) requires *m/z* 486.1838, found *m/z* 486.1842.

Methyl *N*-(ethoxy(oct-7-en-1-yl)phosphoryl)-*N*-tosylglycinate (4c)

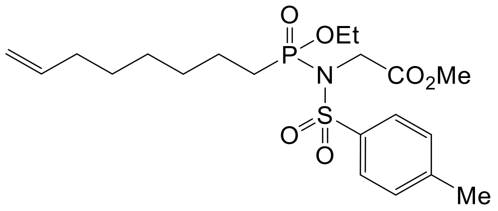

Prepared according to general procedure C; 80% yield; **^{1}H NMR (400 MHz, $CDCl_3$)**: δ 7.80 (d, *J* = 8.3 Hz, 2H), 7.32 (d, *J* = 8.0 Hz, 2H), 5.81 (ddt, *J* = 16.9, 10.2, 6.7 Hz, 1H), 5.03 – 4.90 (m, 2H), 4.40 – 4.08 (m, 3H), 3.82 – 3.72 (m, 1H), 3.62 (s, 3H), 2.44 (s, 3H), 2.33 – 2.22 (m, 2H), 2.09 – 1.99 (m, 2H), 1.73 – 1.59 (m, 2H), 1.48 – 1.31 (m, 6H), 1.28 (t, *J* = 7.1 Hz, 3H); **^{13}C NMR (100 MHz, $CDCl_3$):** δ 169.5, 144.8, 139.1, 136.5, 129.7, 128.2, 114.5, 62.3 (d, *J* = 7.5 Hz), 52.4, 47.9, 33.8, 30.5 (d, *J* = 14.1 Hz), 29.8 (d, *J* = 125.8 Hz), 28.8, 28.7, 22.0 (d, *J* = 4.8 Hz), 21.8, 16.3 (d, *J* = 6.9 Hz); **^{31}P NMR (162 MHz, $CDCl_3$):** δ 33.7; **IR (neat) ν_{max}:** 2980, 2929, 2856, 1758, 1640, 1598, 1439, 1350, 1214, 1163, 1119, 1091, 1026, 965, 842, 816; **HRMS (ESI+):** exact mass calculated for $[M+Na]^+$ ($C_{20}H_{32}NNaO_6PS$) requires *m/z* 468.1580, found *m/z* 468.1586.

Ethyl but-3-en-1-yl(morpholino)phosphinate (4d)

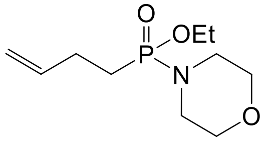

Prepared according to general procedure C; 77% yield; **^{1}H NMR (400 MHz, $CDCl_3$)**: δ 5.84 (ddt, *J* = 16.7, 10.2, 6.4 Hz, 1H), 5.11 – 4.91 (m, 2H), 4.15 – 3.99 (m, 1H), 3.99 – 3.84 (m, 1H), 3.63 (t, *J* = 4.6 Hz, 4H), 3.20 – 3.03 (m, 4H), 2.42 – 2.25 (m, 2H), 1.87 – 1.62 (m, 2H), 1.29 (t, *J* = 7.1 Hz, 3H); **^{13}C NMR (100 MHz, $CDCl_3$):** δ 137.6 (d, *J* = 17.3 Hz), 115.2, 67.5 (d, *J* = 4.7 Hz), 59.7 (d, *J* = 6.7 Hz), 44.2, 26.5 (d, *J* = 3.2 Hz), 25.2 (d, *J* = 130.2 Hz), 16.5 (d, *J* = 6.5 Hz); **^{31}P NMR (162 MHz, $CDCl_3$):** δ 33.1; **IR (neat) ν_{max}:** 2978, 2914, 2853, 1642, 1449, 1392, 1257, 1225, 1210, 1136, 1032, 967, 914, 805, 774, 638; **HRMS (ESI+):** exact mass calculated for $[M+Na]^+$ ($C_{10}H_{20}NNaO_3P$) requires *m/z* 256.1073, found *m/z* 256.1064.

Ethyl (4-methylpiperazin-1-yl)(oct-7-en-1-yl)phosphinate (4e)

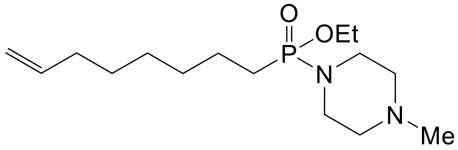

Prepared according to general procedure C; 76% yield; **^{1}H NMR (400 MHz, $CDCl_3$):** δ 5.78 (ddt, *J* = 16.9, 10.2, 6.7 Hz, 1H), 5.01 – 4.89 (m, 2H), 4.10 – 3.99 (m, 1H), 3.96 – 3.85 (m, 1H), 3.26 – 3.10 (m, 4H), 2.47 (bs, 4H), 2.37 (s, 3H), 2.07 – 1.98 (m, 2H), 1.74 – 1.59 (m, 2H), 1.59 – 1.46 (m, 2H), 1.39 – 1.24 (m, 9H); **^{13}C NMR (100 MHz, $CDCl_3$):** δ 139.0, 114.5, 60.0, 55.3 (d, *J* = 4.2 Hz, 2C), 46.2, 43.5 (2C), 33.8, 30.7 (d, *J* = 17.5 Hz), 28.8, 28.7, 26.0 (d, *J* = 131.0 Hz), 22.2 (d, *J* = 4.2 Hz), 16.3 (d, *J* = 6.8 Hz); **^{31}P NMR (162 MHz, $CDCl_3$):** δ 34.5; **IR (neat) ν_{max}:** 2978, 2855, 1641, 1454, 1286, 1224, 1159, 1031, 972, 910, 729, 637; **HRMS (ESI+):** exact mass calculated for $[M+H]^+$ ($C_{15}H_{32}NO_2P$) requires *m/z* 303.2196, found *m/z* 303.2189.

Ethyl (3,3-difluoropyrrolidin-1-yl)(oct-7-en-1-yl)phosphinate (4f)

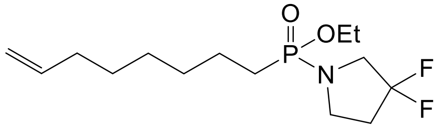

Prepared according to general procedure C; 76% yield; **^{1}H NMR (400 MHz, $CDCl_3$):** δ 5.79 (ddt, *J* = 16.9, 10.2, 6.7 Hz, 1H), 5.01 – 4.95 (m, 1H), 4.95 – 4.90 (m, 1H), 4.13 – 4.04 (m, 1H), 3.98 – 3.87 (m, 1H), 3.52 – 3.34 (m, 4H), 2.37 – 2.23 (m, 2H), 2.06 – 1.99 (m, 2H), 1.76 – 1.63 (m, 2H), 1.63 – 1.49 (m, 2H), 1.41 – 1.27 (m, 9H); **^{13}C NMR (100 MHz, $CDCl_3$):** δ 139.0, 129.2 (td, *J* = 249.1, 10.4 Hz), 114.5, 60.1 (d, *J* = 6.9 Hz), 53.3

(td, J = 31.9, 4.6 Hz), 44.2 (d, J = 3.4 Hz), 35.2 (td, J = 24.4, 5.1 Hz), 33.8, 30.7 (d, J = 17.0 Hz), 28.8, 28.7, 25.7 (d, J = 131.1 Hz), 22.0 (d, J = 4.5 Hz), 16.4 (d, J = 6.5 Hz); **^{31}P NMR (162 MHz, CDCl$_3$):** δ 33.1; **^{19}F NMR (565 MHz, CDCl$_3$):** δ -100.1 (dp, J = 230.3, 13.3 Hz), -100.6 (dp, J = 230.4, 13.0 Hz); **IR (neat) ν_{max}:** 2929, 1641, 1441, 1393, 1223, 1160, 1121, 1030, 957, 9817, 638; **HRMS (ESI+):** exact mass calculated for $[M+Na]^+$ ($C_{14}H_{26}F_2NNaO_2P$) requires *m/z* 332.1561, found *m/z* 332.1559.

Ethyl *N*-allyl-*N*-methyl-*P*-(oct-7-en-1-yl)phosphonamidate (4g)

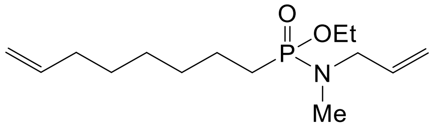

Prepared according to general procedure C; 33% yield; **^{1}H NMR (400 MHz, CDCl$_3$):** δ 5.84 – 5.67 (m, 2H), 5.23 – 5.13 (m, 2H), 5.02 – 4.94 (m, 1H), 4.94 – 4.89 (m, 1H), 4.08 – 3.97 (m, 1H), 3.90 – 3.80 (m, 1H), 3.66 – 3.52 (m, 2H), 2.58 (d, J = 8.9 Hz, 3H), 2.06 – 1.98 (m, 2H), 1.75 – 1.51 (m, 4H), 1.39 – 1.25 (m, 9H); **^{13}C NMR (100 MHz, CDCl$_3$):** δ 139.1, 135.0 (d, J = 2.9 Hz), 117.5, 114.4, 59.2 (d, J = 6.8 Hz), 51.2 (d, J = 4.2 Hz), 33.8, 32.8, 30.7 (d, J = 16.9 Hz), 28.8, 28.8 (d, J = 0.9 Hz), 26.1 (d, J = 130.8 Hz), 22.3 (d, J = 4.3 Hz), 16.4 (d, J = 6.6 Hz); **^{31}P NMR (162 MHz, CDCl$_3$):** δ 36.5; **IR (neat) ν_{max}:** 2928, 2854, 1641, 1444, 1217, 1164, 1034, 948, 933, 811, 782; **HRMS (ESI+):** exact mass calculated for $[M+Na]^+$ ($C_{14}H_{28}NNaO_2P$) requires *m/z* 296.1750, found *m/z* 196.1738.

Ethyl *N*-benzyl-*P*-(oct-7-en-1-yl)phosphonamidate (4h)

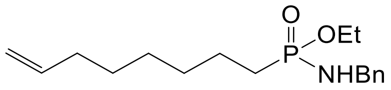

Prepared according to general procedure C; 66% yield; **^{1}H NMR (400 MHz, CDCl$_3$):** δ 7.39 – 7.28 (m, 5H), 5.79 (ddt, J = 16.9, 10.2, 6.7 Hz, 1H), 5.02 – 4.95 (m, 1H), 4.95 – 4.91 (m, 1H), 4.08 – 3.96 (m, 3H), 3.92 – 3.85 (m, 1H), 3.68 (bs, 1H), 2.05 – 1.96 (m, 2H), 1.67 – 1.55 (m, 2H), 1.54 – 1.41 (m, 2H), 1.35 – 1.18 (m, 9H); **^{13}C NMR (100 MHz, CDCl$_3$):** δ 139.7 (d, J = 4.8 Hz), 139.1, 128.7 (2C), 127.5, 127.3 (2C), 114.4, 60.1 (d, J = 6.5 Hz), 44.7 (d, J = 16.7 Hz), 33.8, 30.7 (d, J = 17.9 Hz), 28.8, 28.7, 26.6 (d, J = 131.0 Hz), 22.1 (d, J = 4.0 Hz), 16.3 (d, J = 6.7 Hz); **^{31}P NMR (162 MHz, CDCl$_3$):** δ 36.9; **IR (neat) ν_{max}:** 2978, 1640, 1454, 1244, 1166, 1029, 960, 698, 638; **HRMS (ESI+):** exact mass calculated for $[M+Na]^+$ ($C_{17}H_{28}NNaO_2P$) requires *m/z* 332.1750, found *m/z* 332.1739.

Ethyl *P*-(but-3-en-1-yl)-*N*,*N*-dimethylphosphonamidate (4i)

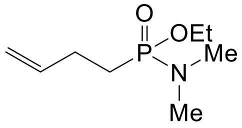

Prepared according to general procedure C (tetrabutylammonium chloride was used instead of tetraethylammonium; extraction with dichloromethane); 29% yield; **^{1}H NMR (400 MHz, $CDCl_3$):** δ 5.84 (ddt, *J* = 16.7, 10.2, 6.4 Hz, 1H), 5.05 (ddd, *J* = 17.1, 3.3, 1.6 Hz, 1H), 4.98 (ddd, *J* = 10.1, 2.7, 1.3 Hz, 1H), 4.04 (ddq, *J* = 14.3, 10.2, 7.1 Hz, 1H), 3.88 (ddq, *J* = 14.2, 10.2, 7.1 Hz, 1H), 2.68 (d, *J* = 8.9 Hz, 6H), 2.43 – 2.23 (m, 2H), 1.82 – 1.68 (m, 2H), 1.29 (t, *J* = 7.1 Hz, 3H); **^{13}C NMR (100 MHz, $CDCl_3$):** δ 137.8 (d, *J* = 16.9 Hz), 114.8, 59.2 (d, *J* = 6.8 Hz), 36.1 (2C), 26.3 (d, *J* = 3.4 Hz), 24.7 (d, *J* = 130.4 Hz), 16.3(d, *J* = 6.6 Hz); **^{31}P NMR (162 MHz, $CDCl_3$):** δ 36.0; **IR (neat) ν_{max}:** 2981, 2905, 1642, 1447, 1300, 1230, 1211, 1040, 990, 952, 809, 765, 750; **HRMS (ESI+):** exact mass calculated for $[M+Na]^+$ ($C_8H_{18}NNaO_2P$) requires *m/z* 214.0973, found *m/z* 214.0968.

Ethyl 6-(morpholino(phenoxy)phosphoryl)hexanoate (6)

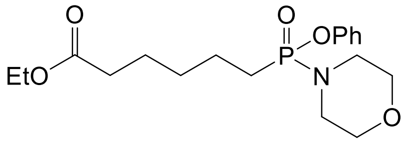

Prepared according to general procedure C; 45% yield; **^{1}H NMR (400 MHz, $CDCl_3$):** δ 7.30 (t, *J* = 7.9 Hz, 2H), 7.19 (d, *J* = 7.9 Hz, 2H), 7.12 (t, *J* = 7.3 Hz, 1H), 4.11 (q, *J* = 7.1 Hz, 2H), 3.55 – 3.41 (m, 4H), 3.19 – 3.07 (m, 4H), 2.30 (t, *J* = 7.4 Hz, 2H), 1.96 – 1.77 (m, 2H), 1.77 – 1.60 (m, 4H), 1.51 – 1.41 (m, 2H), 1.23 (t, *J* = 7.1 Hz, 3H); **^{13}C NMR (100 MHz, $CDCl_3$):** δ 173.6, 151.0 (d, *J* = 9.3 Hz), 129.8 (2C), 124.6, 120.4 (d, *J* = 5.0 Hz, 2C), 67.1 (d, *J* = 4.8 Hz, 2C), 60.4, 44.1 (2C), 34.1, 30.2 (d, *J* = 16.9 Hz), 26.0 (d, *J* = 132.8 Hz), 24.6, 22.0 (d, *J* = 4.4 Hz), 14.3; **^{31}P NMR (162 MHz, $CDCl_3$):** δ 32.1; **IR (neat) ν_{max}:** 2935, 2855, 1728, 1592, 1489, 1201, 1112, 970, 894, 764, 732, 692; **HRMS (ESI+):** exact mass calculated for $[M+Na]^+$ ($C_{18}H_{28}NNaO_5P$) requires *m/z* 392.1597, found *m/z* 392.1592.

tert-Butyl (ethoxy(oct-7-en-1-yl)phosphoryl)-L-prolinate (8)

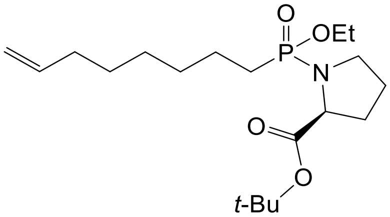

Prepared according to general procedure C; 43% yield, d.r. 1:1.8; **^{1}H NMR (600 MHz, $CDCl_3$)**: δ 5.85 – 5.77 (m, 1H), 5.04 – 4.87 (m, 2H), 4.26 – 4.19 (m, 1H, dia2), 4.18 – 4.12 (m, 1H, dia1), 4.10 – 4.03 (m, 1H), 3.92 – 3.81 (m, 1H), 3.31 – 3.20 (m, 2H), 2.17 – 2.11 (m, 1H), 2.08 – 2.02 (m, 2H), 1.99 – 1.93 (m, 2H), 1.89 – 1.85 (m, 1H), 1.84 – 1.76 (m, 3H), 1.69 – 1.58 (m, 3H), 1.48 (s, 9H, dia1), 1.47 (s, 9H, dia2), 1.43 – 1.36 (m, 5H), 1.33 – 1.26 (m, 3H); **^{13}C NMR (150 MHz, $CDCl_3$):** δ 173.9 (dia1), 173.6 (dia2), 139.2 (dia2), 139.2 (dia1), 114.4 (dia2), 114.4 (dia1), 81.1 (dia2), 80.9 (dia1), 60.4 (d, *J* = 5.0 Hz), 59.8 (d, *J* = 6.5 Hz, dia1), 59.2 (d, *J* = 6.4 Hz, dia2), 46.8 (d, *J* = 3.7 Hz, dia2), 46.7 (d, *J* = 4.4 Hz, dia1), 33.9, 31.6 (d, *J* = 6.6 Hz, dia1), 31.4 (d, *J* = 6.3 Hz, dia2), 30.9 (d, *J* = 17.3 Hz, dia2), 30.8 (d, *J* = 16.7 Hz, dia1), 28.9, 28.8, 28.2 (dia2, 3C), 28.1 (dia1, 3C), 26.7 (d, *J* = 130.9 Hz, dia2), 26.4 (d, *J* = 131.4 Hz, dia1), 25.4 – 25.2 (m), 22.4 – 22.1 (m), 16.6 – 16.4 (m); **^{31}P NMR (243 MHz, $CDCl_3$):** δ 33.6, 33.1; **IR (neat) ν_{max}:** 2973, 2929, 2859, 1736, 1653, 1453, 1223, 1147, 1036, 952, 845, 812; **HRMS (ESI+):** exact mass calculated for $[M+Na]^+$ ($C_{10}H_{20}NNaO_3P$) requires *m/z* 396.2274, found *m/z* 396.2267.

5.5. Alkynes as nucleophiles: preparation of phosphinates

Ethyl (3-(benzyloxy)prop-1-yn-1-yl)(ethyl)phosphinate (5a)

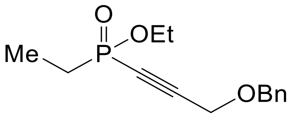

Prepared according to general procedure C; 57% yield; **^{1}H NMR (600 MHz, $CDCl_3$)**: δ 7.40 – 7.28 (m, 5H), 4.60 (s, 2H), 4.27 (d, *J* = 2.9 Hz, 2H), 4.26 – 4.18 (m, 1H), 4.17 – 4.09 (m, 1H), 1.94 – 1.80 (m, 2H), 1.37 (t, *J* = 7.1 Hz, 3H), 1.21 (dt, *J* = 21.6, 7.7 Hz, 3H); **^{13}C NMR (150 MHz, $CDCl_3$):** δ 136.8, 128.7, 128.3, 128.2, 97.6 (d, *J* = 32.1 Hz), 79.1 (d, *J* = 186.4 Hz), 72.3, 62.1 (d, *J* = 7.4 Hz), 57.2 (d, *J* = 2.3 Hz), 24.0 (d, *J* = 120.6 Hz), 16.4 (d, *J* = 6.8 Hz), 6.0 (d, *J* = 5.6 Hz); **^{31}P NMR (243 MHz, $CDCl_3$):** δ 24.4; **IR (neat) ν_{max}:** 3032, 2980, 2940, 2905, 2196, 1643, 1496, 1391, 1353, 1282, 1212, 1076, 1022, 1003, 957, 787, 751, 699; **HRMS (ESI+):** exact mass calculated for $[M+Na]^+$ ($C_{14}H_{19}NaO_3P$) requires *m/z* 289.0964, found *m/z* 289.0962.

Ethyl 6-(ethoxy((triisopropylsilyl)ethynyl)phosphoryl)hexanoate (5b)

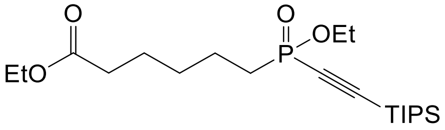

Prepared according to general procedure C; 90% yield; **^{1}H NMR (600 MHz, $CDCl_3$)**: δ 4.23 – 4.15 (m, 2H), 4.12 (q, *J* = 7.1 Hz, 2H), 2.29 (t, *J* = 7.5 Hz, 2H), 1.90 – 1.80 (m, 2H), 1.73 – 1.66 (m, 2H), 1.63 (dd, *J* = 15.3, 7.7 Hz, 2H), 1.50 – 1.42 (m, 2H), 1.36 (t, *J* = 7.1 Hz, 3H), 1.25 (t, *J* = 7.1 Hz, 3H), 1.13 – 1.06 (m, 21H); **^{13}C NMR (150 MHz, $CDCl_3$):** δ 173.7, 107.4 (d, *J* = 23.3 Hz), 99.7 (d, *J* = 172.3 Hz), 62.0 (d, *J* = 7.5 Hz), 60.4, 34.1, 30.8 (d, *J* = 118.3 Hz), 30.0 (d, *J* = 17.2 Hz), 24.6, 21.8 (d, *J* = 4.0 Hz), 18.6, 16.4 (d, *J* = 6.6 Hz), 14.4, 11.0; **^{31}P NMR (243 MHz, $CDCl_3$):** δ 21.5; **IR (neat) ν_{max}:** 2943, 2867, 2123, 1735, 1463, 1371, 1240, 1194, 1120, 1097, 1032, 998, 957, 883, 790, 708, 679, 587; **HRMS (ESI+):** exact mass calculated for $[M+Na]^+$ ($C_{21}H_{41}NaO_4PSi$) requires *m/z* 439.2404, found *m/z* 439.2399.

Ethyl ethyl(thiophen-3-ylethynyl)phosphinate (5c)

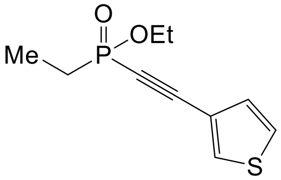

Prepared according to general procedure C; 47% yield; **^{1}H NMR (400 MHz, $CDCl_3$)**: 7.70 (dd, *J* = 3.0, 0.6 Hz, 1H), 7.32 (dd, *J* = 5.0, 3.0 Hz, 1H), 7.20 (dd, *J* = 5.0, 1.1 Hz, 1H), 4.32 – 4.12 (m, 2H), 1.92 (dq, *J* = 17.1, 7.7 Hz, 2H), 1.39 (t, *J* = 7.1 Hz, 3H), 1.25 (dt, *J* = 21.5, 7.7 Hz, 3H); **^{13}C NMR (150 MHz, $CDCl_3$):** δ 133.2 (d, *J* = 2.0 Hz), 130.1, 126.3, 119.3 (d, *J* = 4.4 Hz), 95.7 (d, *J* = 35.6 Hz), 81.0 (d, *J* = 192.4 Hz), 62.0 (d, *J* = 7.4 Hz), 24.2 (d, *J* = 121.1 Hz), 16.4 (d, *J* = 6.9 Hz), 6.1 (d, *J* = 5.6 Hz); **^{31}P NMR (243 MHz, $CDCl_3$):** δ 25.3; **IR (neat) ν_{max}:** 2978, 2924, 2856, 2169, 1656, 1458, 1395, 1360, 1259, 1208, 1160, 1030, 952, 874, 787, 772, 697, 668, 638, 604; **HRMS (ESI+):** exact mass calculated for $[M+Na]^+$ ($C_{10}H_{13}NaO_2PS$) requires *m/z* 251.0272, found *m/z* 251.0265.

Ethyl (cyclopropylethynyl)(oct-7-en-1-yl)phosphinate (5d)

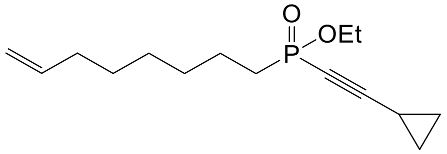

Prepared according to general procedure C; 68% yield; **^{1}H NMR (600 MHz, CDCl$_3$)**: δ 5.79 (ddt, *J* = 16.9, 10.2, 6.7 Hz, 1H), 5.05 – 4.85 (m, 2H), 4.20 – 4.11 (m, 1H), 4.12 – 3.99 (m, 1H), 2.08 – 1.99 (m, 2H), 1.83 – 1.73 (m, 2H), 1.66 – 1.57 (m, 2H), 1.43 – 1.29 (m, 10H), 0.95 – 0.90 (m, 2H), 0.89 – 0.84 (m, 2H); **^{13}C NMR (150 MHz, CDCl$_3$):** δ 139.1, 114.5 (d, *J* = 4.6 Hz), 107.0 (d, *J* = 36.9 Hz), 68.4 (d, *J* = 198.9 Hz), 61.6 (d, *J* = 7.3 Hz), 33.8, 31.0 (d, *J* = 119.2 Hz), 30.4 (d, *J* = 17.4 Hz), 28.8, 28.7, 21.9 (d, *J* = 4.3 Hz), 16.4 (d, *J* = 6.9 Hz), 9.3 (dd, *J* = 2.9, 1.1 Hz), -0.0 (d, *J* = 4.1 Hz); **^{31}P NMR (243 MHz, CDCl$_3$):** δ 23.2; **IR (neat) ν_{max}:** 2979, 2929, 2856, 2191, 1640, 1456, 1229, 1031, 957, 836; **HRMS (ESI+):** exact mass calculated for $[M+Na]^+$ ($C_{15}H_{25}NaO_2P$) requires *m/z* 291.1484, found *m/z* 291.1479.

Ethyl dodec-1-yn-1-yl(oct-7-en-1-yl)phosphinate (5e)

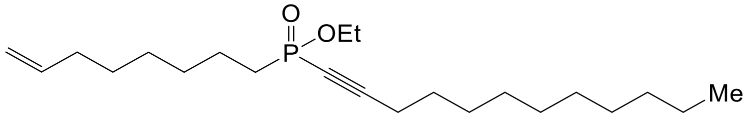

Prepared according to general procedure C; 59% yield; **^{1}H NMR (400 MHz, CDCl$_3$)**: δ 5.77 (ddt, *J* = 16.9, 10.2, 6.7 Hz, 1H), 5.03 – 4.84 (m, 2H), 4.22 – 4.00 (m, 2H), 2.36 – 2.23 (m, 2H), 2.06 – 1.98 (m, 2H), 1.83 – 1.74 (m, 2H), 1.67 – 1.50 (m, 4H), 1.42 – 1.21 (m, 23H), 0.86 (t, *J* = 6.9 Hz, 3H); **^{13}C NMR (100 MHz, CDCl$_3$):** δ 139.0, 114.4, 104.3 (d, *J* = 35.5 Hz), 73.6 (d, *J* = 197.1 Hz), 61.6 (d, *J* = 7.2 Hz), 33.8, 32.0, 31.0 (d, *J* = 111.4 Hz), 30.4, 30.3, 29.6, 29.6, 29.4, 29.1, 28.81 (d, *J* = 18.6 Hz), 28.7, 27.7, 22.8, 21.9 (d, *J* = 4.0 Hz), 19.4 (d, *J* = 2.6 Hz), 16.4 (d, *J* = 6.8 Hz), 14.2; **^{31}P NMR (162 MHz, CDCl$_3$):** δ 23.1; **IR (neat) ν_{max}:** 2924, 2854, 2195, 1640, 1463, 1240, 1035, 956, 909, 717; **HRMS (ESI+):** exact mass calculated for $[M+Na]^+$ ($C_{22}H_{41}NaO_2P$) requires *m/z* 391.2736, found *m/z* 391.2734.

Ethyl oct-7-en-1-yl(phenylethynyl)phosphinate (5f)

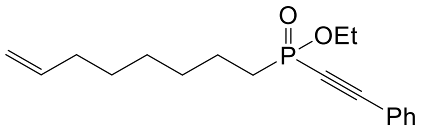

Prepared according to general procedure C; 62% yield; **^{1}H NMR (600 MHz, CDCl$_3$)**: δ 7.56 – 7.51 (m, 2H), 7.47 – 7.41 (m, 1H), 7.39 – 7.34 (m, 2H), 5.78 (ddt, *J* = 16.9, 10.2, 6.7 Hz, 1H), 5.00 – 4.89 (m, 2H), 4.30 –

4.14 (m, 2H), 2.07 – 2.00 (m, 2H), 1.95 – 1.88 (m, 2H), 1.74 – 1.67 (m, 2H), 1.47 – 1.31 (m, 9H); **^{13}C NMR (150 MHz, CDCl$_3$):** δ 139.0, 132.7 (d, *J* = 1.8 Hz), 130.6, 128.7, 120.0 (d, *J* = 3.8 Hz), 114.5, 100.3 (d, *J* = 35.0 Hz), 81.5 (d, *J* = 191.7 Hz), 62.0 (d, *J* = 7.3 Hz), 33.8, 31.0 (d, *J* = 119.2 Hz), 30.4 (d, *J* = 17.3 Hz), 28.7, 28.7, 21.9 (d, *J* = 4.0 Hz), 16.4 (d, *J* = 6.8 Hz); **^{31}P NMR (243 MHz, CDCl$_3$):** δ 23.6; **IR (neat) ν_{max}:** 2978, 2929, 2856, 2179, 1640, 1490, 1461, 1443, 1393, 1240, 1033, 959, 848, 759; **HRMS (ESI+):** exact mass calculated for [M+Na]$^+$ ($C_{18}H_{25}NaO_2P$) requires *m/z* 327.1484, found *m/z* 327.1479.

Ethyl oct-7-en-1-yl(5-phenylpent-1-yn-1-yl)phosphinate (5g)

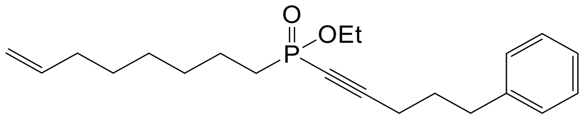

Prepared according to general procedure C; 83% yield; **^{1}H NMR (400 MHz, CDCl$_3$)**: δ 7.32 – 7.27 (m, 2H), 7.24 – 7.14 (m, 3H), 5.88 – 5.71 (m, 1H), 5.04 – 4.86 (m, 2H), 4.28 – 4.02 (m, 2H), 2.73 (t, *J* = 7.5 Hz, 2H), 2.34 (td, *J* = 7.1, 3.4 Hz, 2H), 2.03 (q, *J* = 6.9 Hz, 2H), 1.95 – 1.77 (m, 4H), 1.73 – 1.59 (m, 2H), 1.46 – 1.28 (m, 9H); **^{13}C NMR (100 MHz, CDCl$_3$):** δ 140.9, 139.1, 128.6 (d, *J* = 5.3 Hz), 126.4, 114.5, 103.5 (d, J = 35.5 Hz), 74.2 (d, *J* = 195.8 Hz), 61.7 (d, *J* = 7.4 Hz), 34.8, 33.8, 31.0 (d, *J* = 130.8 Hz), 30.5, 29.3, 28.7 (d, *J* = 4.6 Hz), 21.9 (d, *J* = 4.3 Hz), 18.8, 16.4 (d, *J* = 6.9 Hz); **^{31}P NMR (162 MHz, CDCl$_3$):** δ 23.0; **IR (neat) ν_{max}:** 452, 3064, 3027, 2978, 2928, 2856, 2195, 1640, 1603, 1496, 1455, 1393, 1350, 1236, 1162, 1098, 1034, 957, 910, 850, 805, 748, 701, 618; **HRMS (ESI+):** exact mass calculated for [M+Na]$^+$ ($C_{21}H_{31}NaO_2P$) requires *m/z* 369.1959, found *m/z* 369.1950.

Ethyl methyl((triisopropylsilyl)ethynyl)phosphinate (5h)

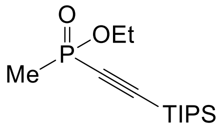

Prepared according to general procedure C on 1 mmol scale; 40% yield; **^{1}H NMR (400 MHz, CDCl$_3$):** δ 4.25 – 4.06 (m, 2H), 1.64 (d, *J* = 16.6 Hz, 3H), 1.35 (t, *J* = 7.1 Hz, 3H), 1.14 – 1.04 (m, 21H); **^{13}C NMR (100 MHz, CDCl$_3$):** δ 106.8 (d, *J* = 25.5 Hz), 100.5 (d, *J* = 178.7 Hz), 61.9 (d, *J* = 7.0 Hz), 18.5 (6C), 17.9 (d, *J* = 121.8 Hz), 16.3 (d, *J* = 6.9 Hz), 11.0 (3C); **^{31}P NMR (162 MHz, CDCl$_3$):** δ 16.8; **IR (neat) ν_{max}:** 2945, 1867, 1464, 1305, 1239, 1037, 962, 884, 797, 776; **HRMS (ESI+):** exact mass calculated for [M+Na]$^+$ ($C_{14}H_{29}NaO_2PSi$) requires *m/z* 311.1572, found *m/z* 311.1561.

6 References

[1] H. Huang, J. Denne, C.-H. Yang, H. Wang, J. Y. Kang, *Angew. Chem. Int. Ed.* **2018**, DOI 10.1002/anie.201802082.

[2] K. N. Bauer, H. T. Tee, I. Lieberwirth, F. R. Wurm, *Macromolecules* **2016**, *49*, 3761.

[3] T. M. Putvinski, M. L. Schilling, H. E. Katz, C. E. D. Chidsey, A. M. Mujsce, A. B. Emerson, *Langmuir* **1990**, *6*, 1567–1571.

[4] M. Cherevatskaya, M. Neumann, S. Füldner, C. Harlander, S. Kümmel, S. Dankesreiter, A. Pfitzner, K. Zeitler, B. König, *Angew. Chem. Int. Ed*. **2012**, *51*, 4062–4066.

[5] J.-W. Yuan, L.-R. Yang, P. Mao, L.-B. Qu, *RSC Adv.* **2016**, *6*, 87058–87065.

[6] H. Rao, Y. Jin, H. Fu, Y. Jiang, Y. Zhao, *Chem. - Eur. J.* **2006**, *12*, 3636–3646.

[7] D. Lecerclé, M. Sawicki, F. Taran, *Org. Lett.* **2006**, *8*, 4283–4285.

[8] M. I. Antczak, J.-L. Montchamp, *Org. Lett.* **2008**, *10*, 977–980.

[9] B. Basnar, M. Litschauer, S. Abermann, E. Bertagnolli, G. Strasser, M.-A. Neouze, *Chem. Commun.* **2011**, *47*, 361–363.

[10] J. Lewkowski, A. Jóźwiak, P. Tokarz, P. M. Zagórski, R. Hamera, D. Cal, G. Satała, A. J. Bojarski, *Heteroat. Chem.* **2015**, *26*, 290–298.

[11] S. Gobec, I. Plantan, J. Mravljak, U. Švajger, R. A. Wilson, G. S. Besra, S. L. Soares, R. Appelberg, D. Kikelj, *Eur. J. Med. Chem.* **2007**, *42*, 54–63.

7 NMR spectra

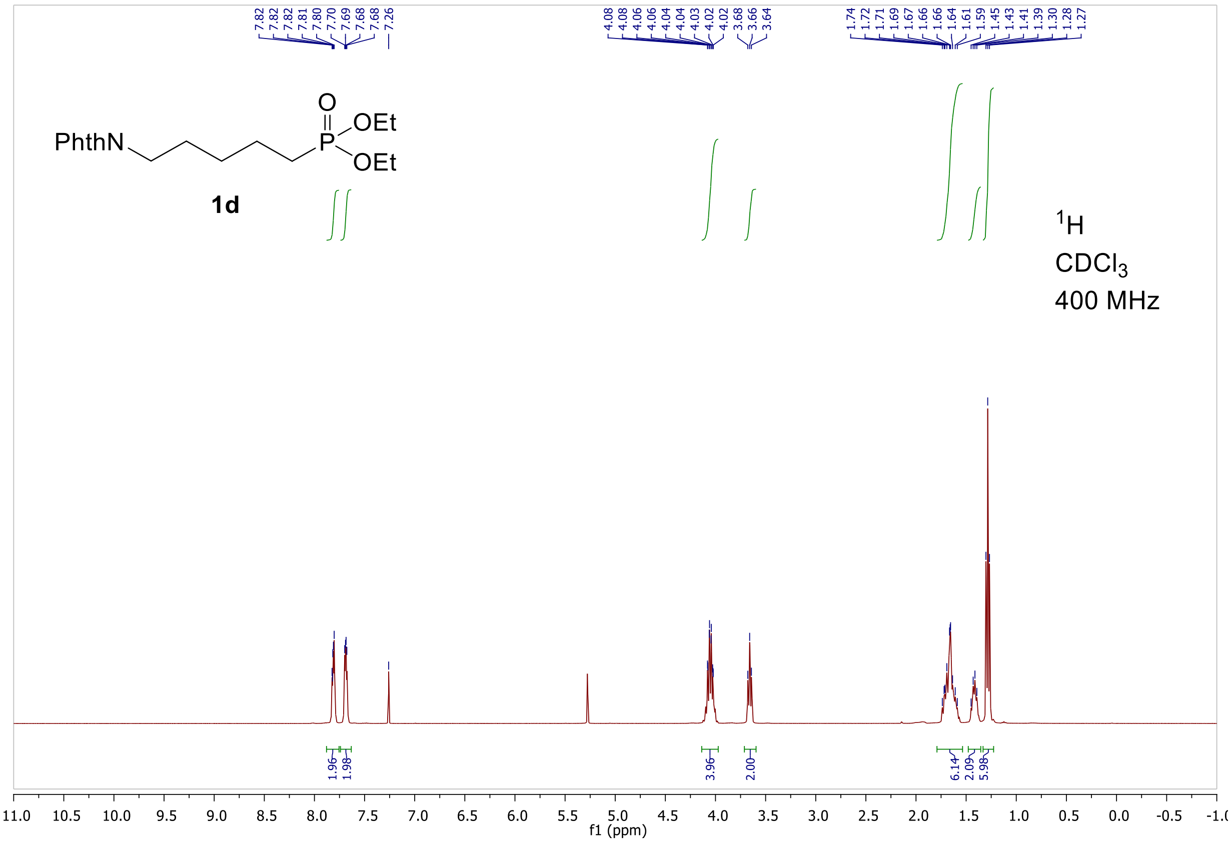

7.82 7.82 7.82 7.81 7.80 7.70 7.69 7.68 7.68 7.26
4.08 4.08 4.06 4.06 4.04 4.04 4.03 4.02 4.02 3.68 3.66 3.64
1.74 1.72 1.71 1.69 1.67 1.66 1.66 1.64 1.61 1.59 1.45 1.43 1.41 1.39 1.30 1.28 1.27
PhthN
O
OEt
P
OEt
1d
1H
CDCl3
400 MHz
1.96 1.98 3.96 2.00 6.14 2.09 5.98
f1 (ppm)

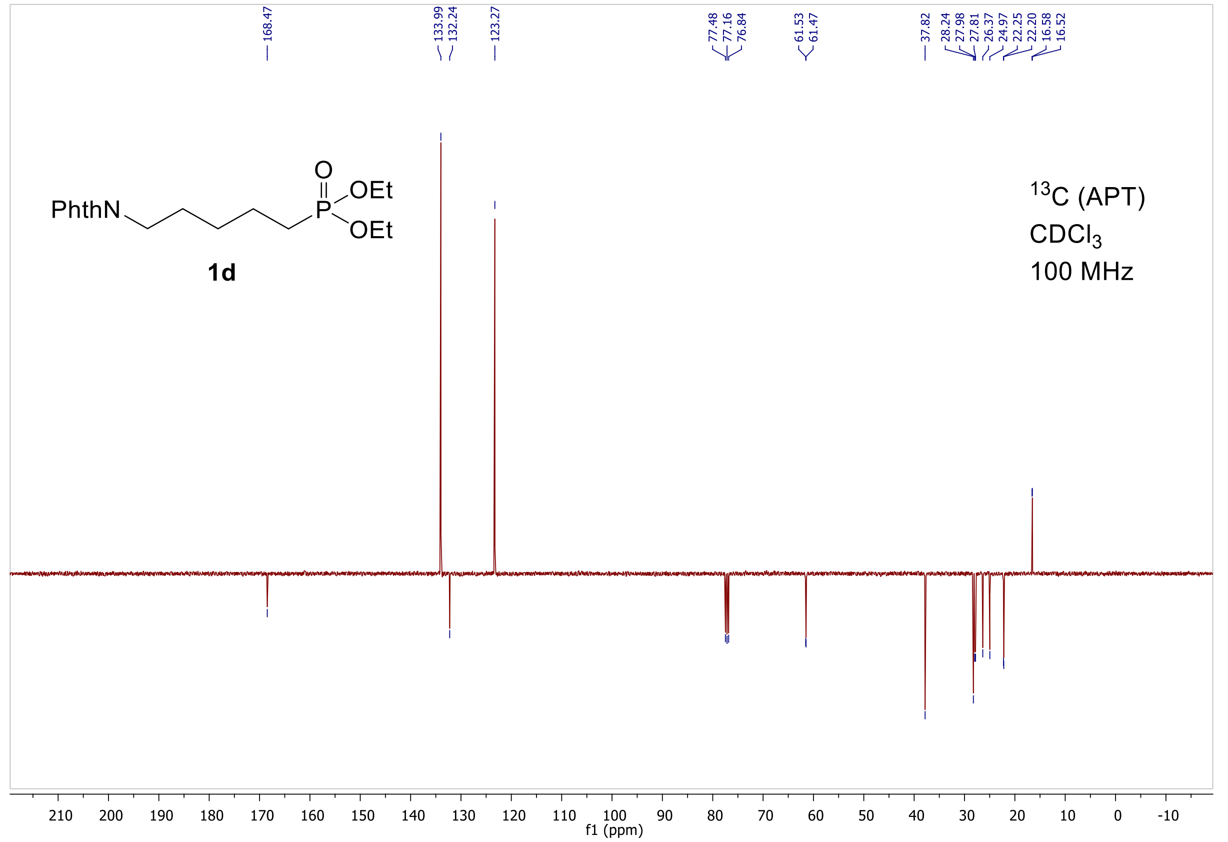

168.47
133.99 132.24
123.27
77.48 77.16 76.84
61.53 61.47
37.82 28.24 27.98 27.81 26.37 24.97 22.25 22.20 16.58 16.52
PhthN
O
OEt
P
OEt
1d
13C (APT)
CDCl3
100 MHz
f1 (ppm)

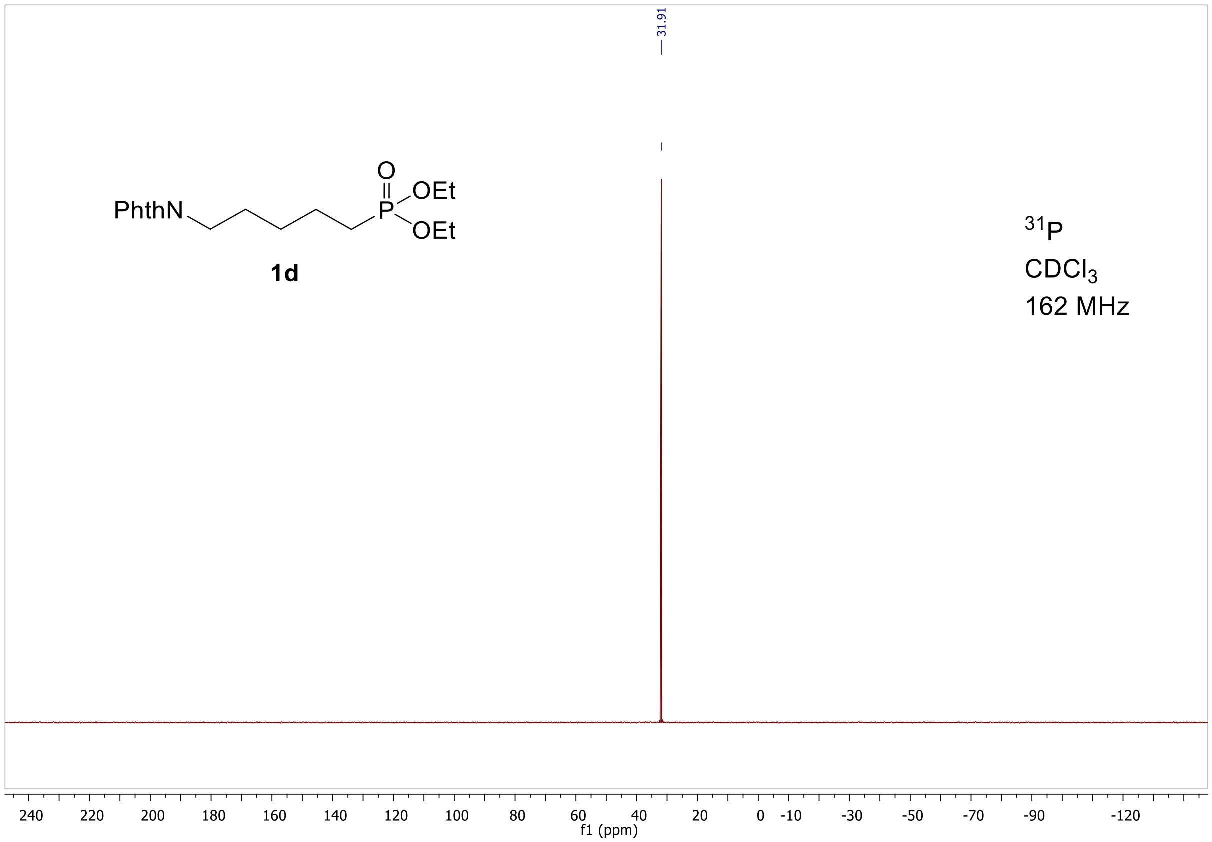
31.91
PhthN
OEt
OEt
O
P
1d
31P
CDCl3
162 MHz
240 220 200 180 160 140 120 100 80 60 40 20 0 -10 -30 -50 -70 -90 -120
f1 (ppm)

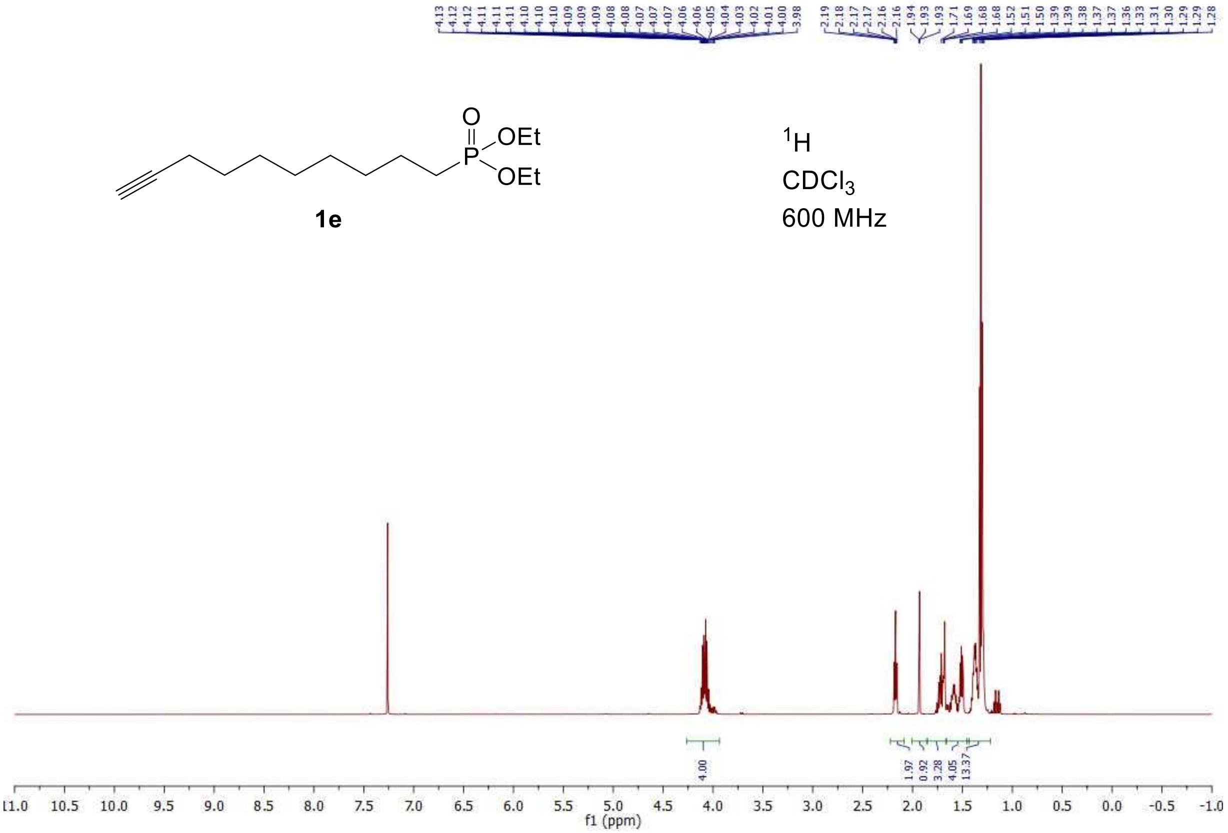

O
OEt
P
OEt
1e
1H
CDCl3
600 MHz
f1 (ppm)

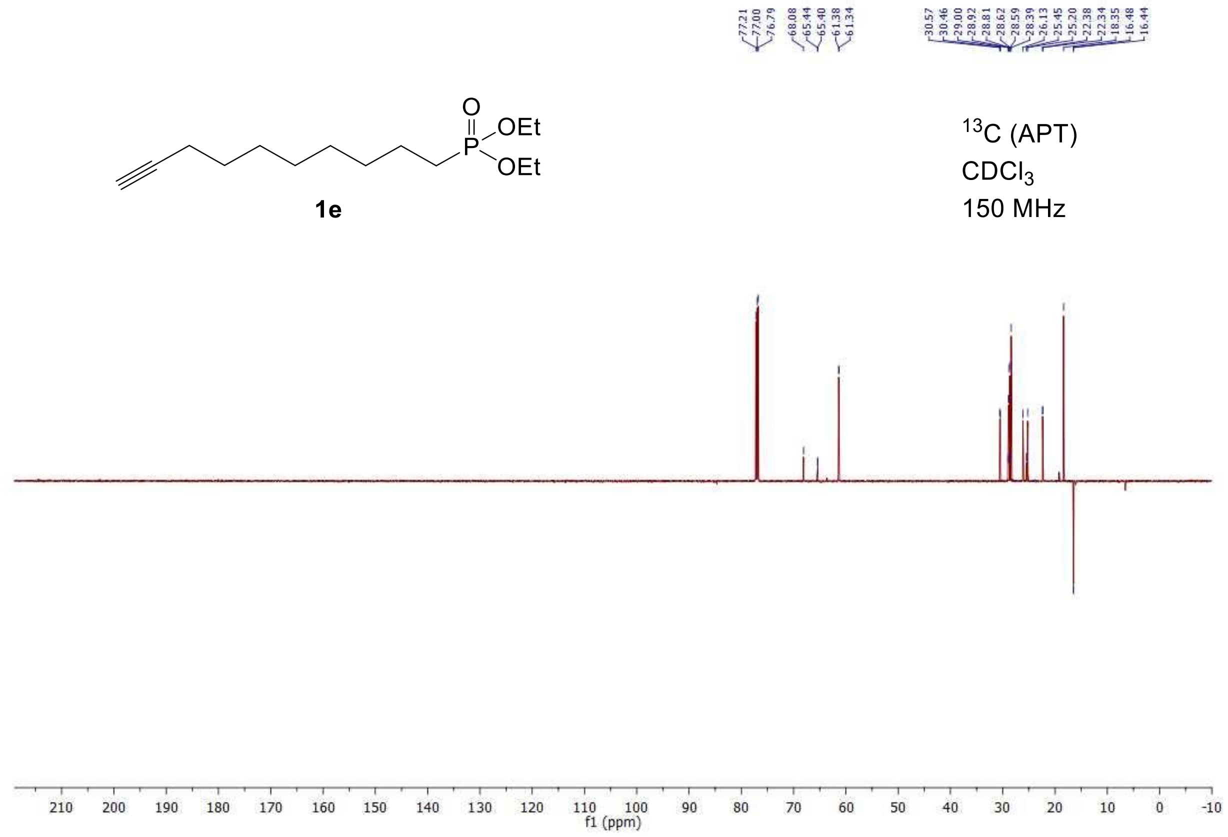

O
OEt
P
OEt
1e
13C (APT)
CDCl3
150 MHz
f1 (ppm)

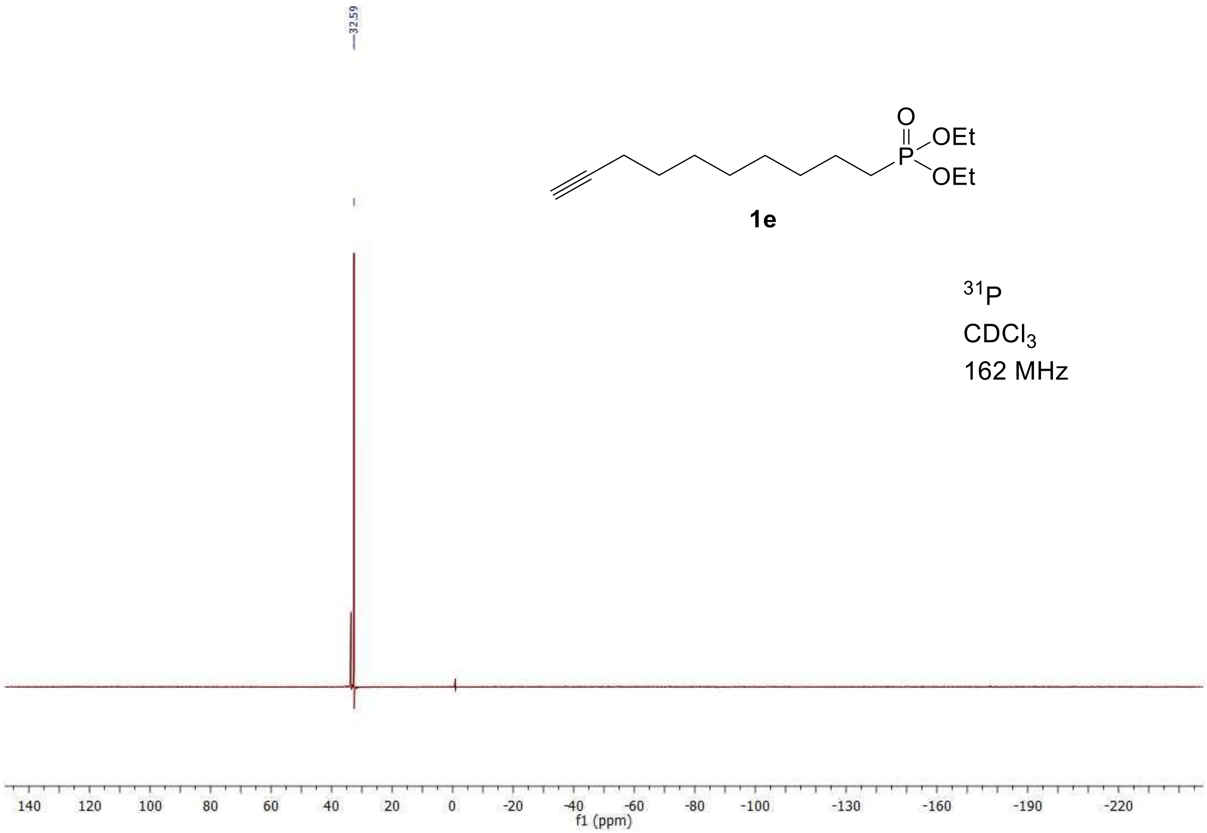
32.59
O
OEt
P
OEt
1e
31P
CDCl3
162 MHz
140 120 100 80 60 40 20 0 -20 -40 -60 -80 -100 -130 -160 -190 -220
f1 (ppm)

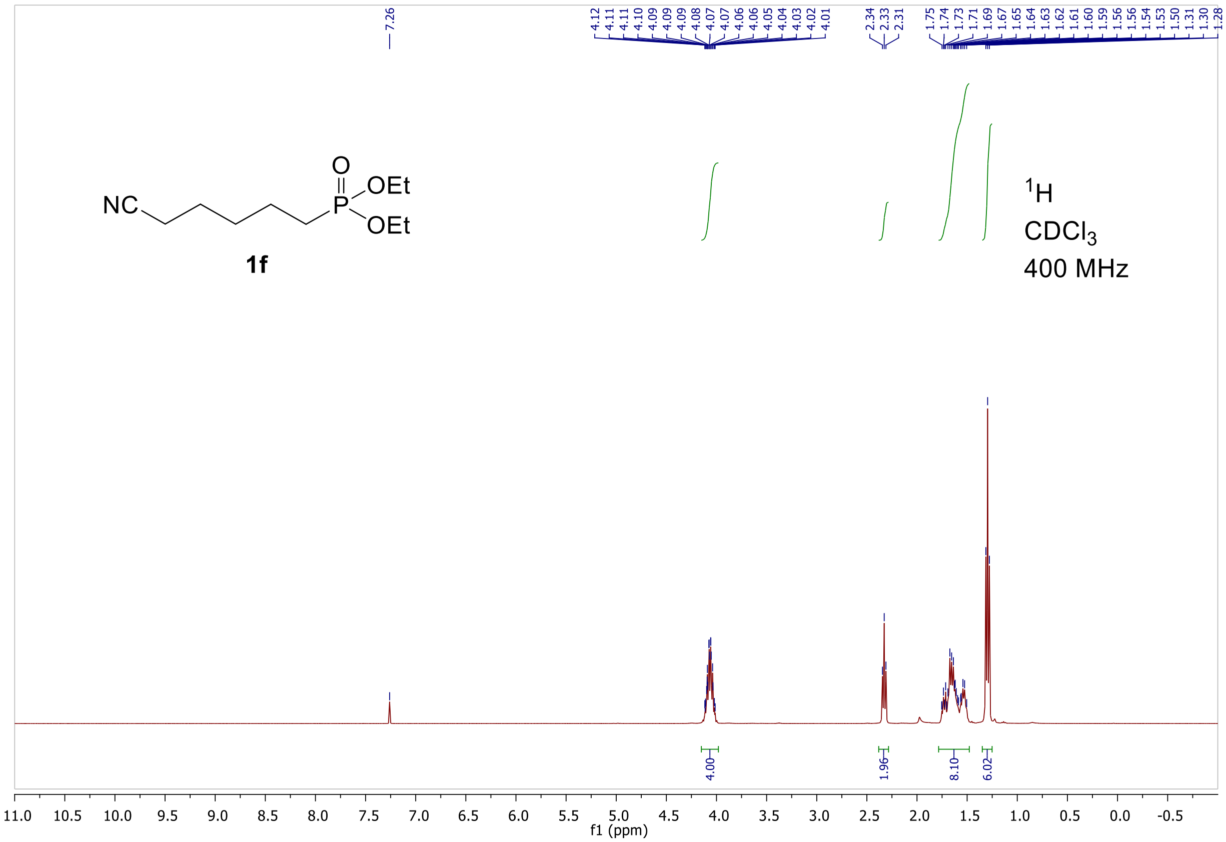
7.26
4.12 4.11 4.11 4.10 4.09 4.09 4.09 4.08 4.07 4.07 4.06 4.06 4.05 4.04 4.03 4.02 4.01
2.34 2.33 2.31
1.75 1.74 1.73 1.71 1.69 1.67 1.65 1.65 1.64 1.63 1.62 1.61 1.60 1.59 1.56 1.56 1.54 1.53 1.50 1.31 1.30 1.28
NC
O
P
OEt
OEt
1f
1H
CDCl3
400 MHz
4.00
1.96
8.10
6.02
11.0 10.5 10.0 9.5 9.0 8.5 8.0 7.5 7.0 6.5 6.0 5.5 5.0 4.5 4.0 3.5 3.0 2.5 2.0 1.5 1.0 0.5 0.0 -0.5
f1 (ppm)

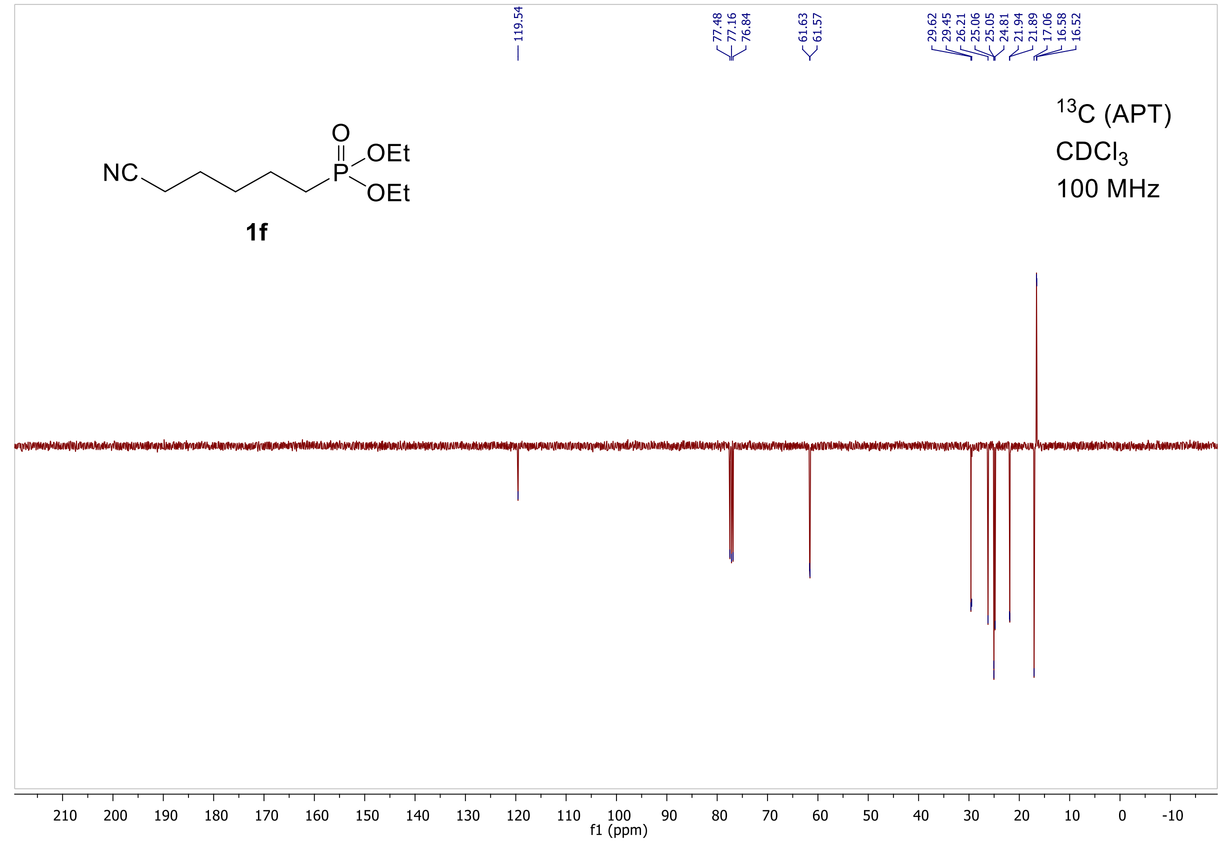
119.54
77.48 77.16 76.84
61.63 61.57
29.62 29.45 26.21 25.06 25.05 24.81 21.94 21.89 17.06 16.58 16.52
NC
O
P
OEt
OEt
1f
13C (APT)
CDCl3
100 MHz
210 200 190 180 170 160 150 140 130 120 110 100 90 80 70 60 50 40 30 20 10 0 -10
f1 (ppm)

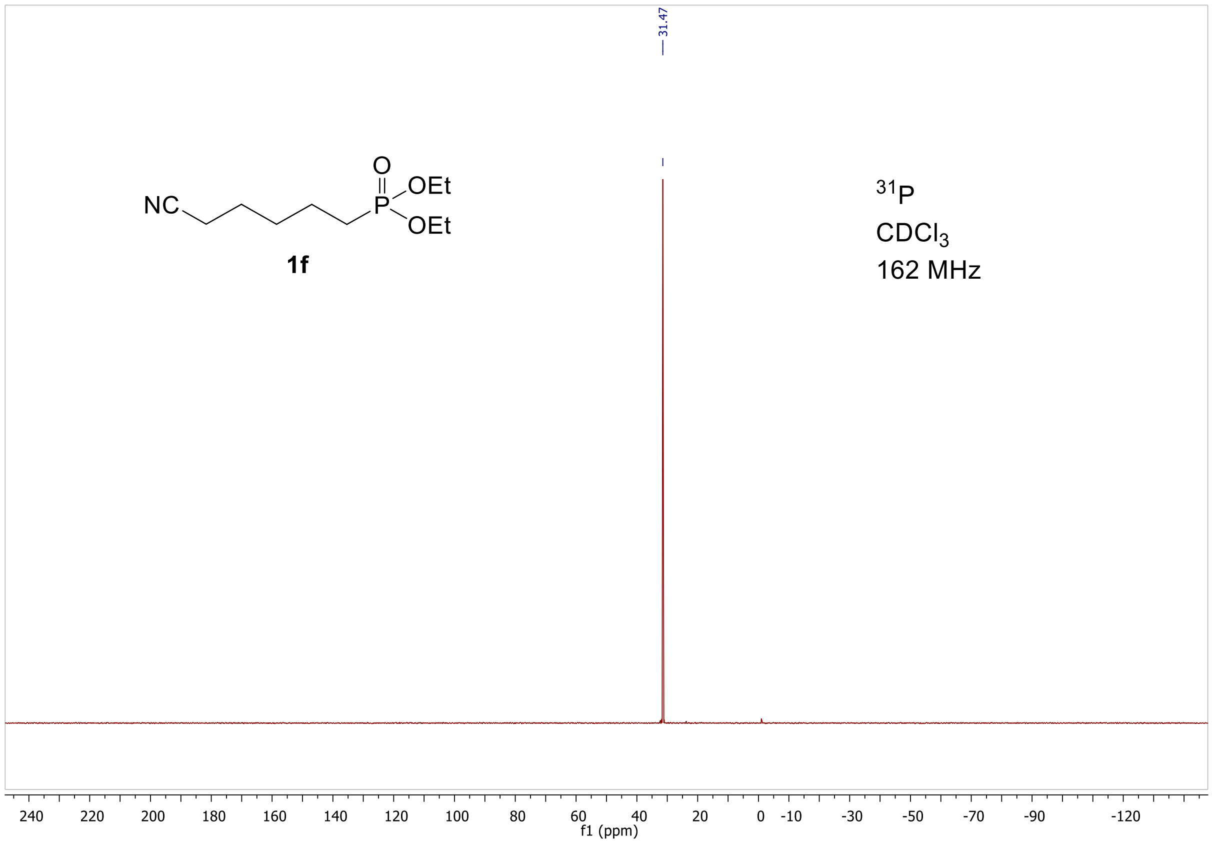
31.47
O
OEt
NC
P
OEt
1f
^{31}P
$CDCl_3$
162 MHz
240 220 200 180 160 140 120 100 80 60 40 20 0 -10 -30 -50 -70 -90 -120
f1 (ppm)

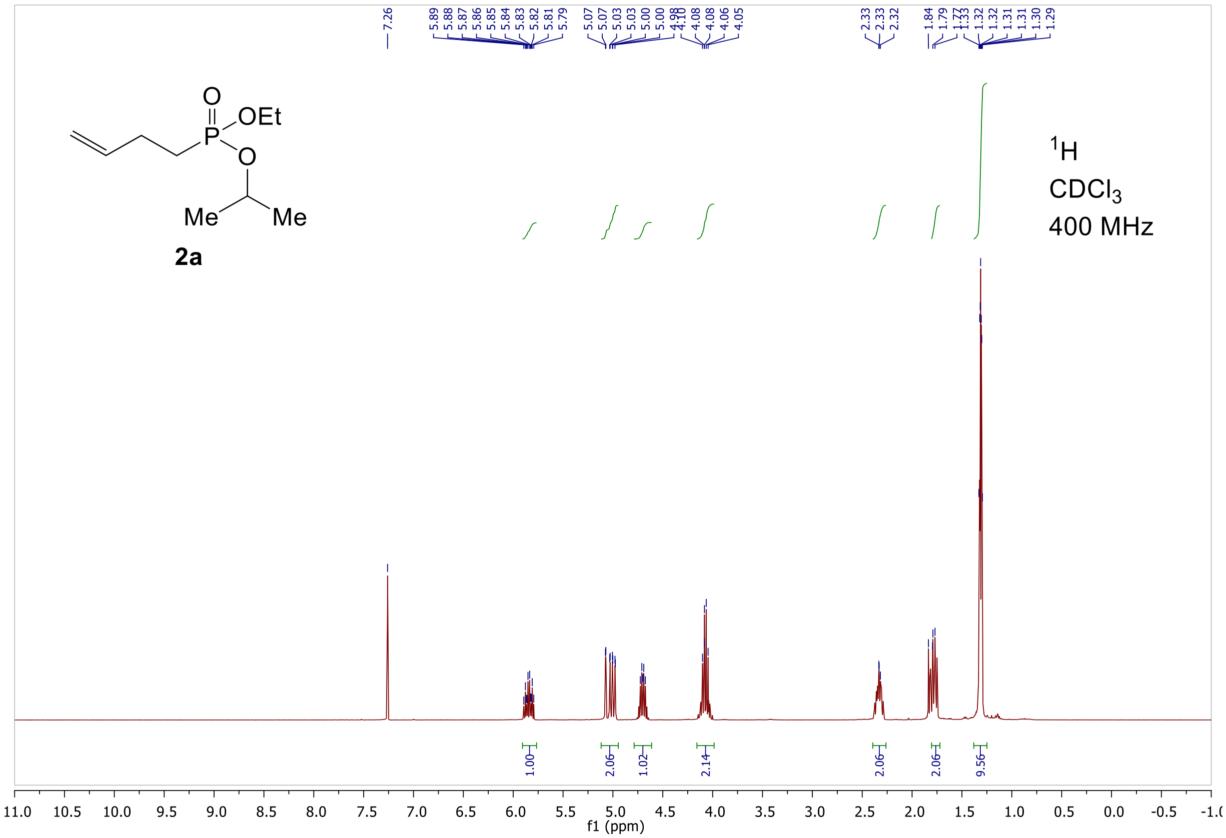

7.26
5.89
5.88
5.87
5.86
5.85
5.84
5.83
5.82
5.81
5.79
5.07
5.07
5.03
5.03
5.00
5.00
4.98
4.10
4.08
4.08
4.06
4.05
2.33
2.33
2.32
1.84
1.79
1.77
1.33
1.32
1.32
1.31
1.31
1.30
1.29
O
OEt
P
O
Me
Me
2a
1H
CDCl3
400 MHz
1.00
2.06
1.02
2.14
2.06
2.06
9.56
11.0 10.5 10.0 9.5 9.0 8.5 8.0 7.5 7.0 6.5 6.0 5.5 5.0 4.5 4.0 3.5 3.0 2.5 2.0 1.5 1.0 0.5 0.0 -0.5
f1 (ppm)

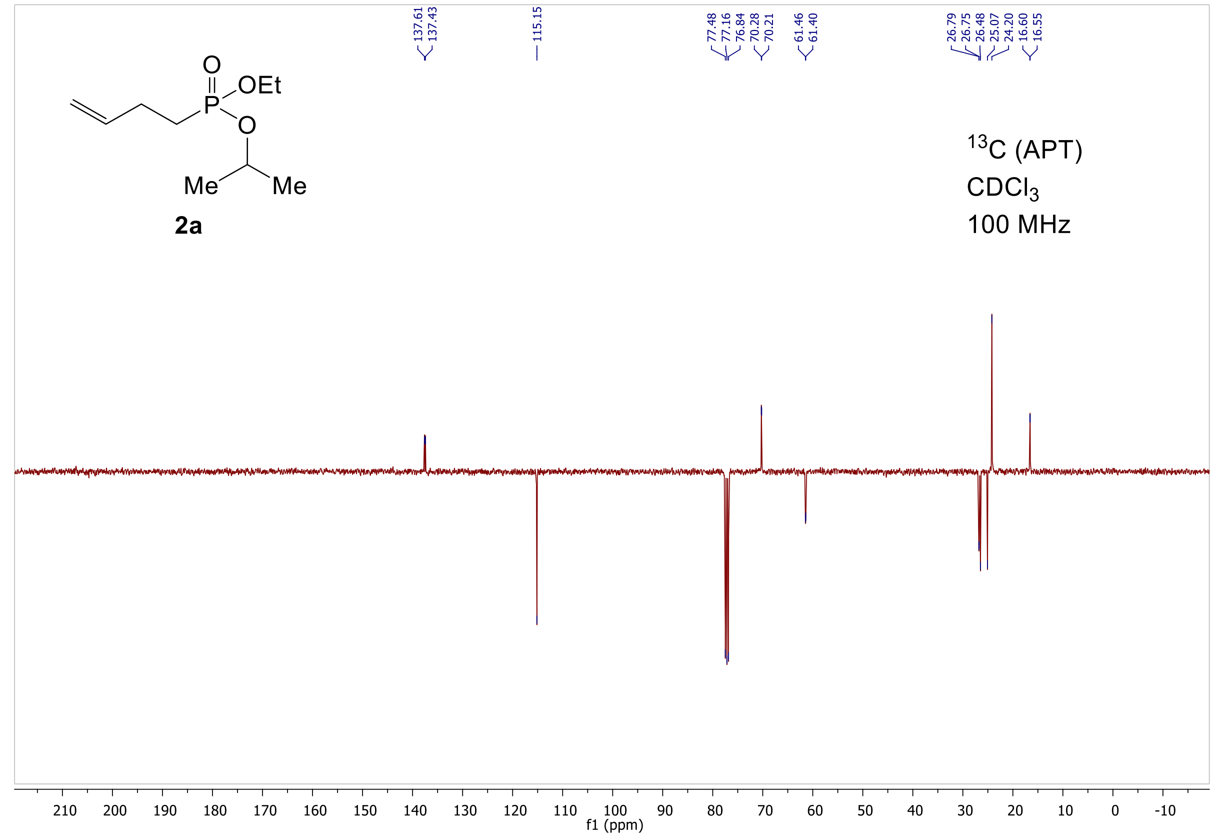

137.61
137.43
115.15
77.48
77.16
76.84
70.28
70.21
61.46
61.40
26.79
26.75
26.48
25.07
24.20
16.60
16.55
O
OEt
P
O
Me
Me
2a
13C (APT)
CDCl3
100 MHz
210 200 190 180 170 160 150 140 130 120 110 100 90 80 70 60 50 40 30 20 10 0 -10
f1 (ppm)

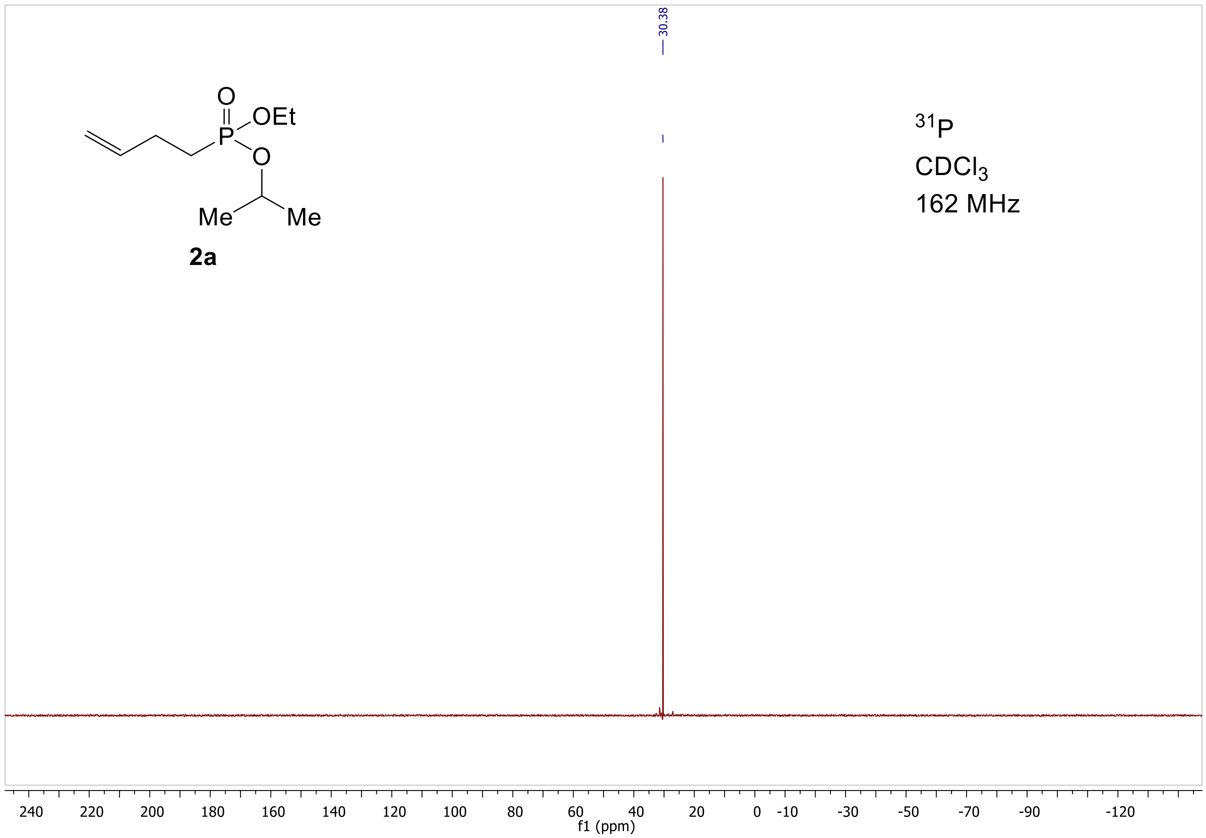
30.38
O
OEt
P
O
Me
Me
2a
31P
CDCl3
162 MHz
240 220 200 180 160 140 120 100 80 60 40 20 0 -10 -30 -50 -70 -90 -120
f1 (ppm)

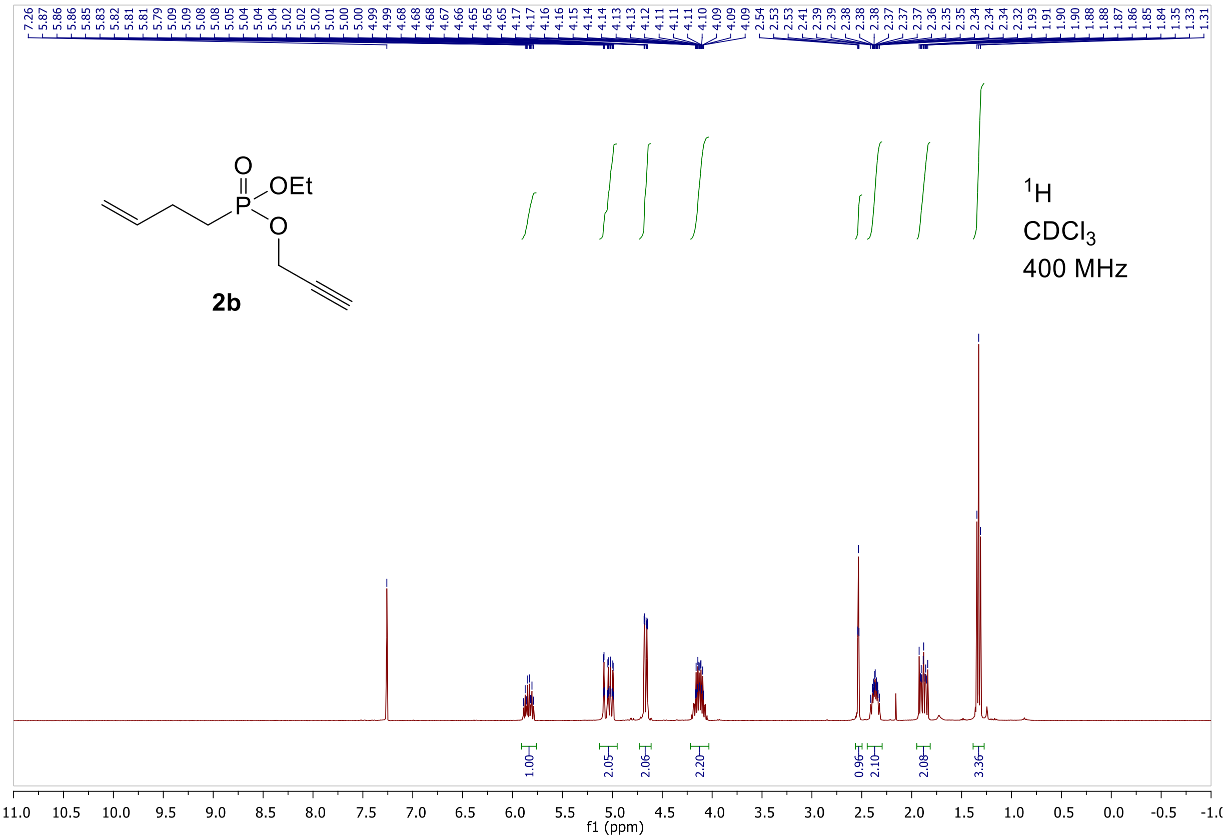

OEt
O
P
O
2b
1H
CDCl3
400 MHz
1.00
2.05
2.06
2.20
0.96
2.10
2.08
3.36
f1 (ppm)

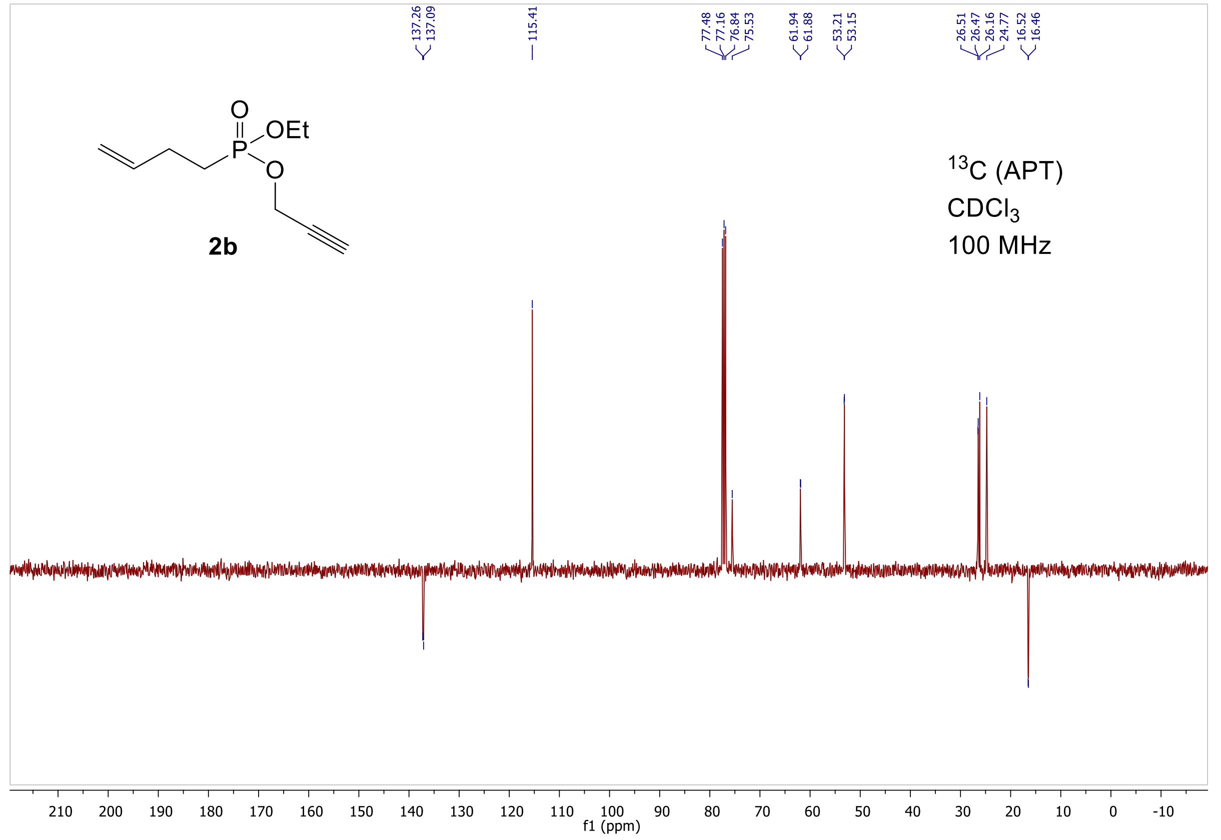

137.26
137.09
115.41
77.48
77.16
76.84
75.53
61.94
61.88
53.21
53.15
26.51
26.47
26.16
24.77
16.52
16.46
OEt
O
P
O
2b
13C (APT)
CDCl3
100 MHz
f1 (ppm)

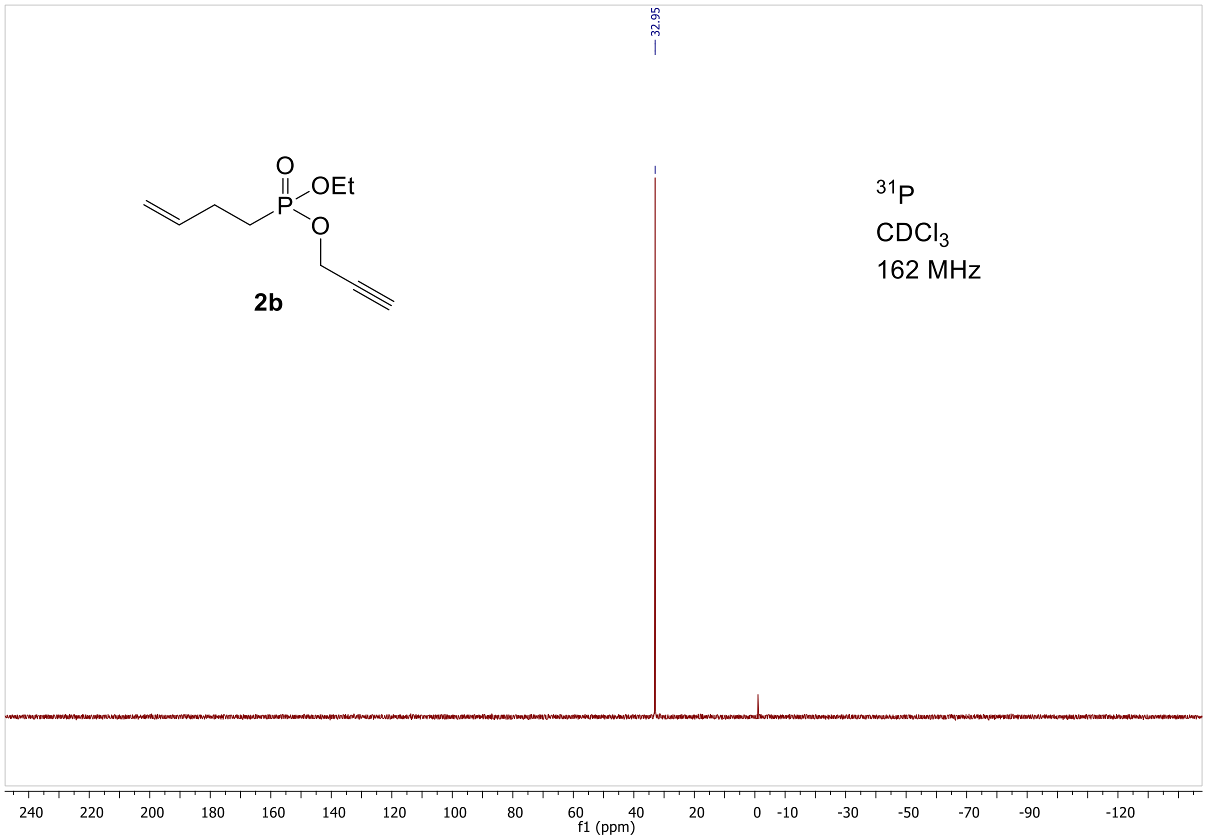

32.95
OEt
O
O
P
2b
^{31}P
$CDCl_3$
162 MHz
240
220
200
180
160
140
120
100
80
60
40
20
0
-10
-30
-50
-70
-90
-120
f1 (ppm)

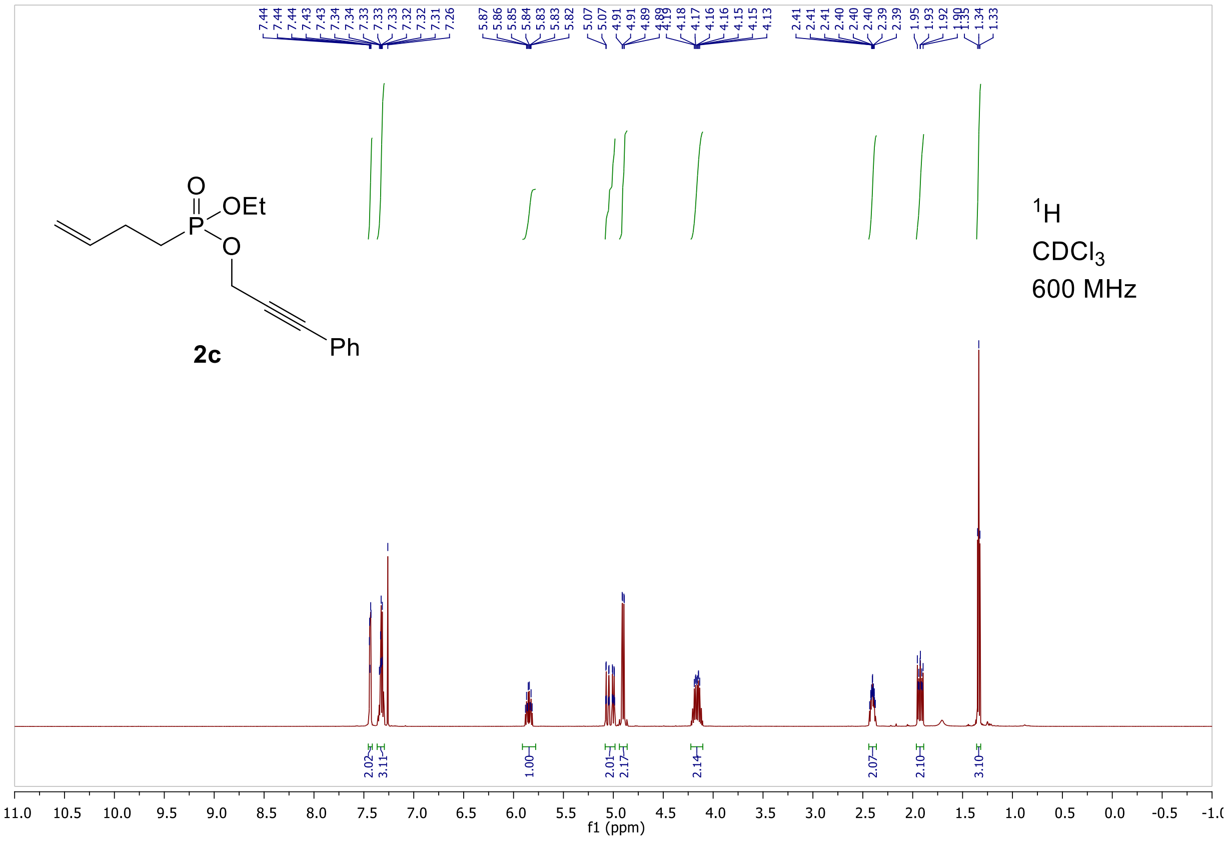
OEt
O
P
O
Ph
2c
1H
CDCl3
600 MHz
f1 (ppm)

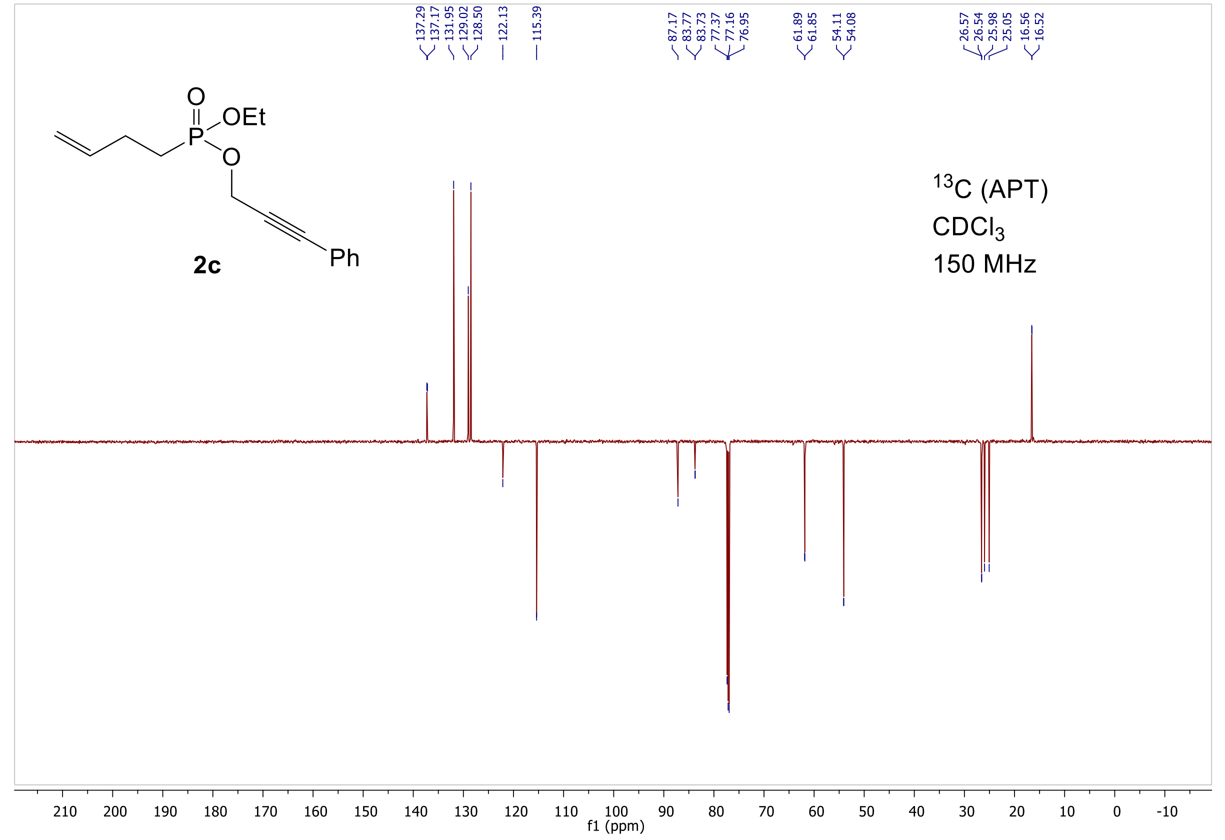
OEt
O
P
O
Ph
2c
13C (APT)
CDCl3
150 MHz
f1 (ppm)

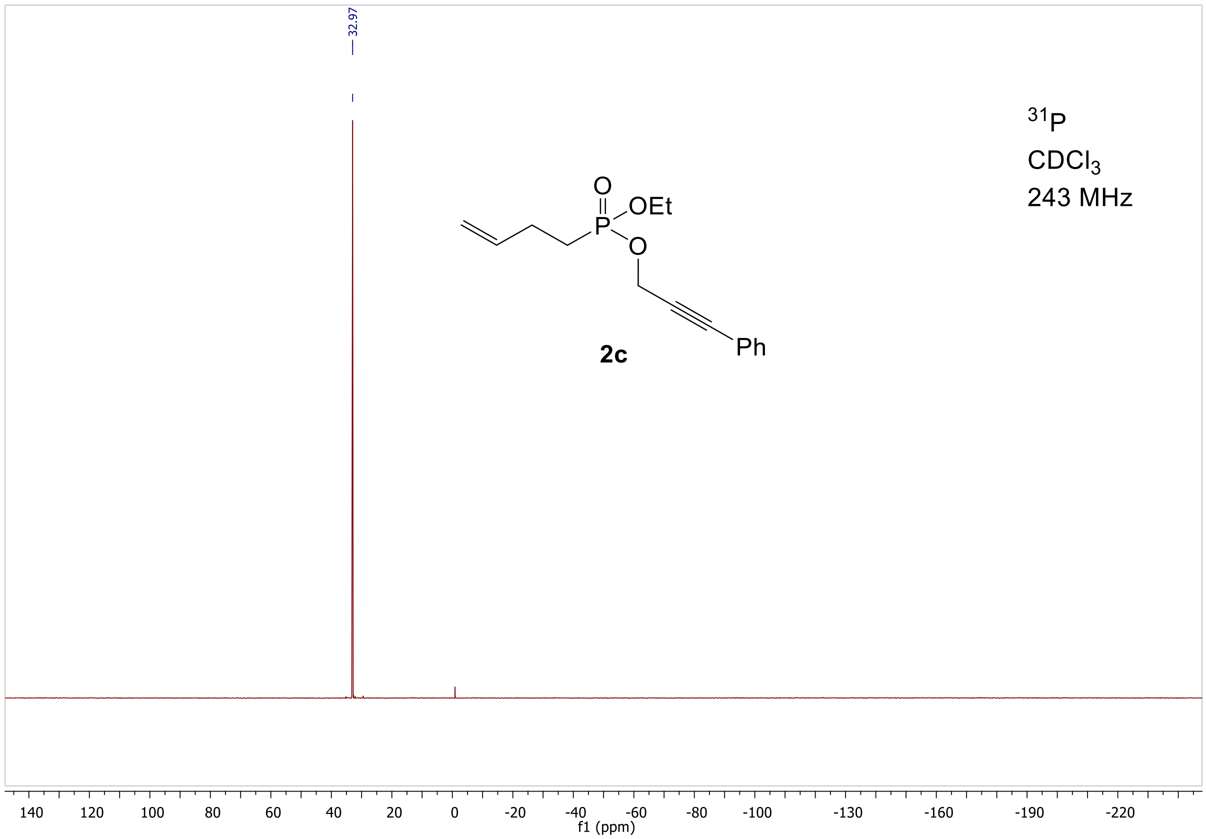
32.97
^{31}P
CDCl$_3$
243 MHz
O
OEt
P
O
Ph
2c
140
120
100
80
60
40
20
0
-20
-40
-60
-80
-100
-130
-160
-190
-220
f1 (ppm)

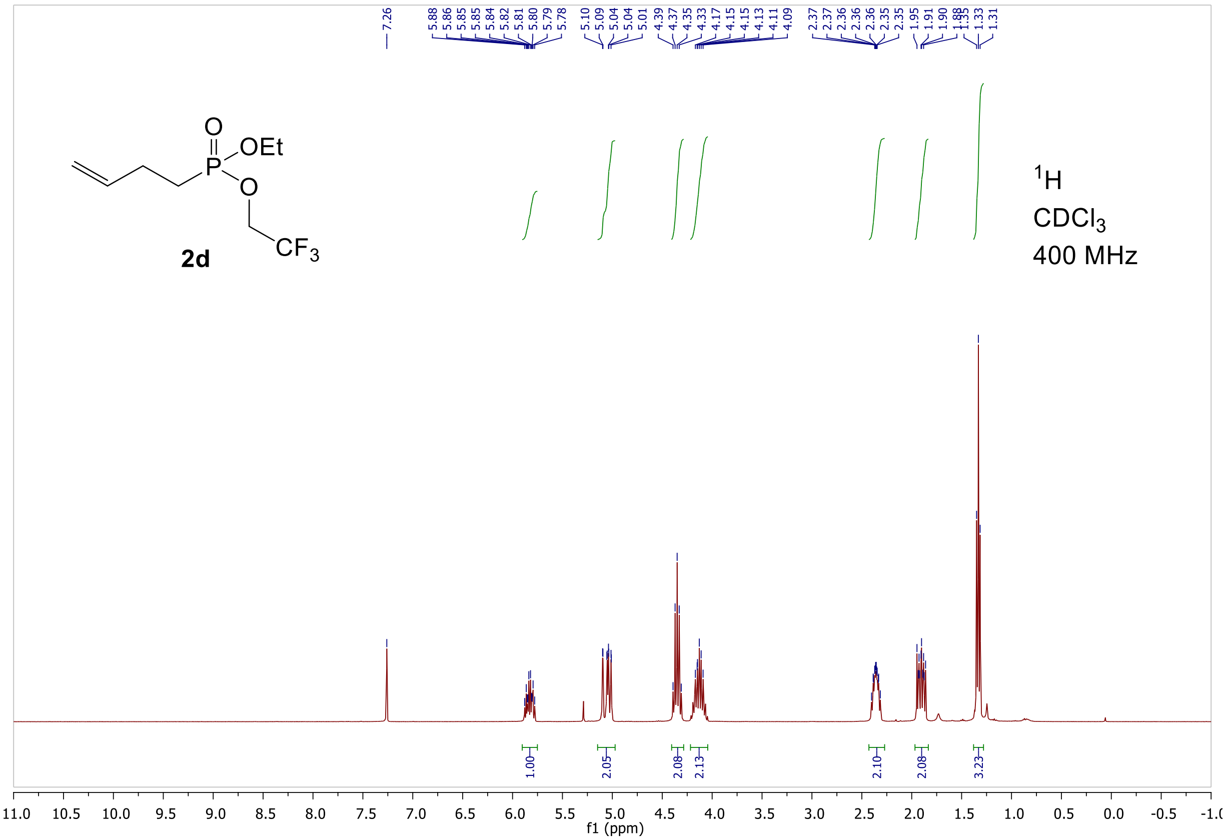
7.26
5.88
5.86
5.85
5.85
5.84
5.82
5.81
5.80
5.79
5.78
5.10
5.09
5.04
5.04
5.01
4.39
4.37
4.35
4.33
4.17
4.15
4.15
4.13
4.11
4.09
2.37
2.37
2.36
2.36
2.35
2.35
1.95
1.91
1.90
1.88
1.35
1.33
1.31
OEt
O
P
O
CF3
2d
1H
CDCl3
400 MHz
1.00
2.05
2.08
2.13
2.10
2.08
3.23
11.0
10.5
10.0
9.5
9.0
8.5
8.0
7.5
7.0
6.5
6.0
5.5
5.0
4.5
4.0
3.5
3.0
2.5
2.0
1.5
1.0
0.5
0.0
-0.5
f1 (ppm)

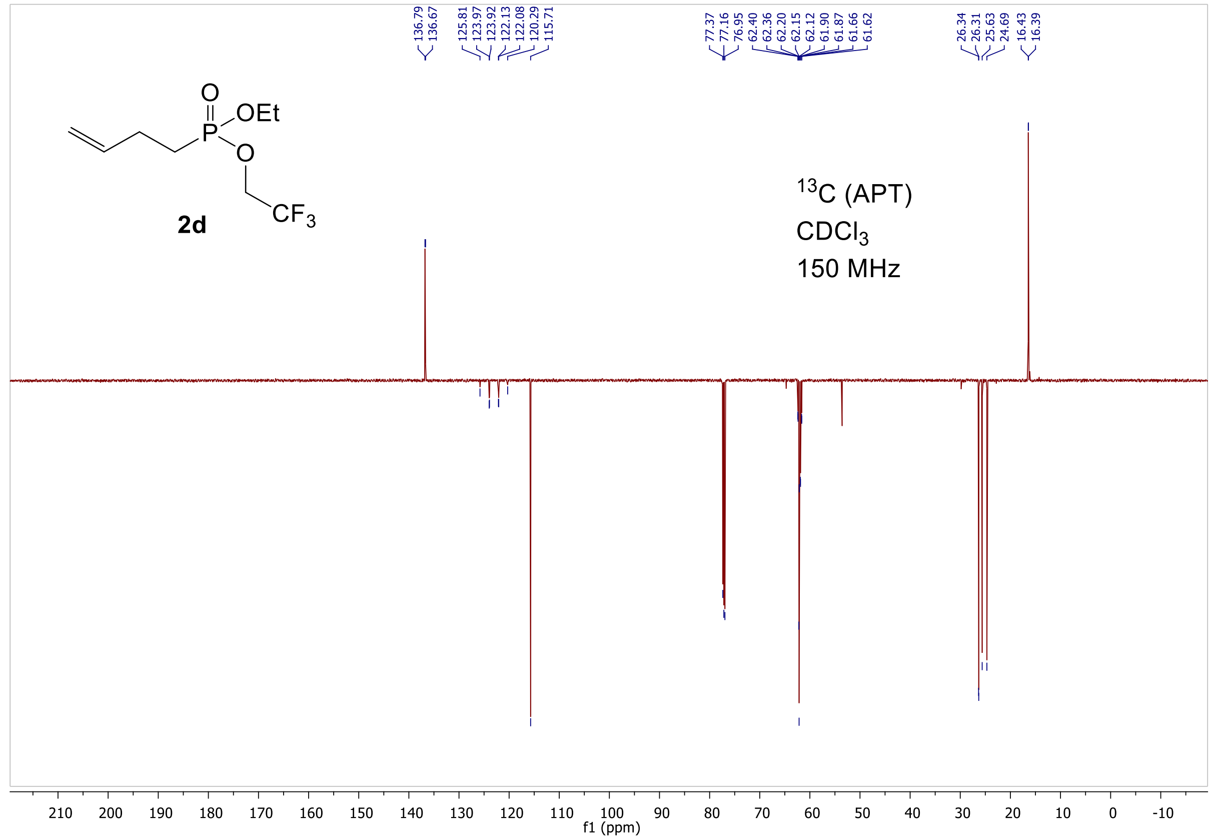
136.79
136.67
125.81
123.97
123.92
122.13
122.08
120.29
115.71
77.37
77.16
76.95
62.40
62.36
62.20
62.15
62.12
61.90
61.87
61.66
61.62
26.34
26.31
25.63
24.69
16.43
16.39
OEt
O
P
O
CF3
2d
13C (APT)
CDCl3
150 MHz
210
200
190
180
170
160
150
140
130
120
110
100
90
80
70
60
50
40
30
20
10
0
-10
f1 (ppm)

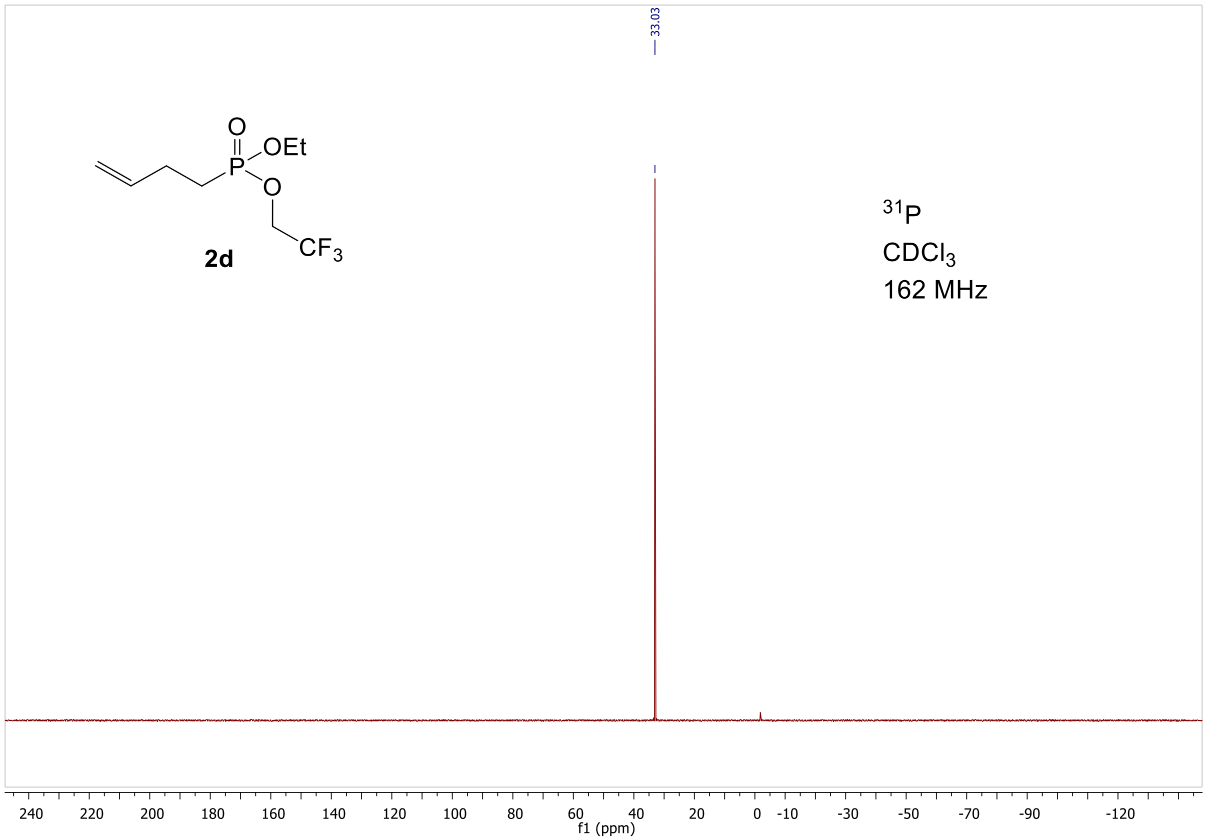
33.03
OEt
O
CF3
2d
31P
CDCl3
162 MHz
240 220 200 180 160 140 120 100 80 60 40 20 0 -10 -30 -50 -70 -90 -120
f1 (ppm)

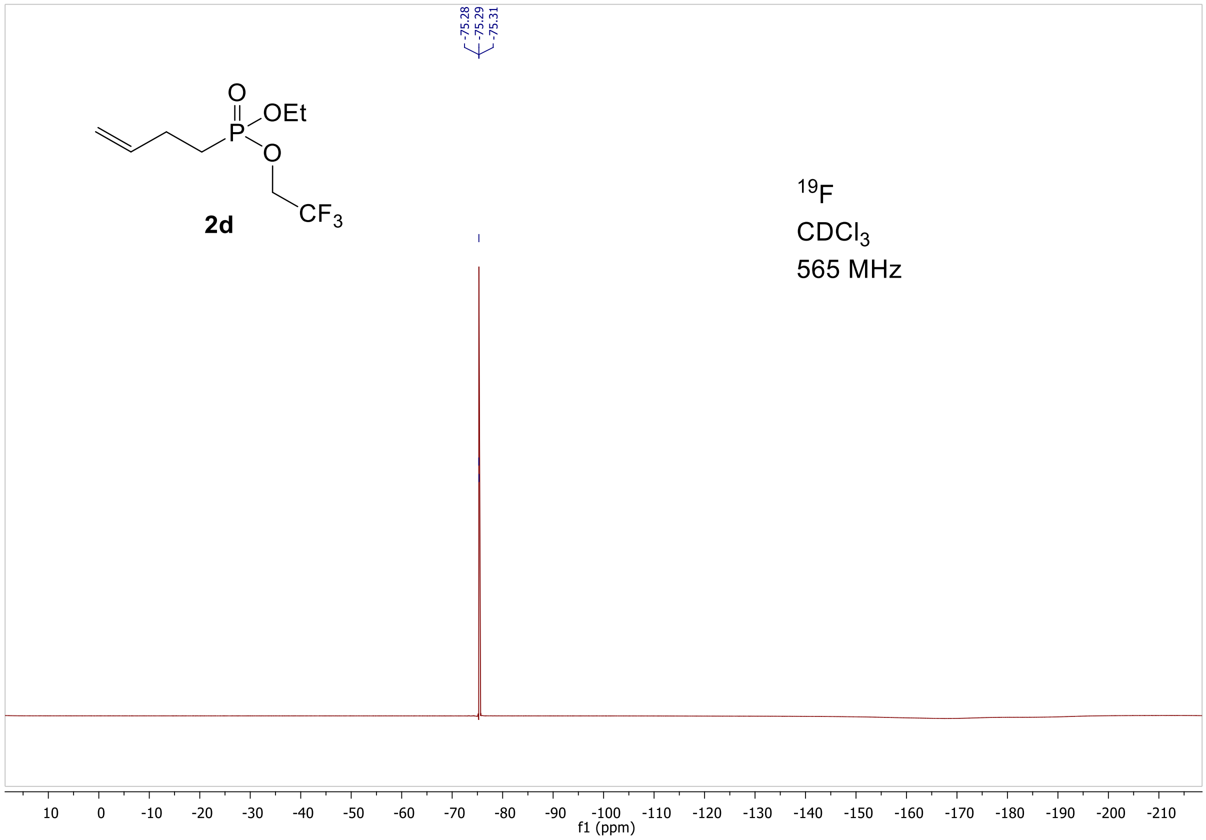
-75.28
-75.29
-75.31
OEt
O
CF3
2d
19F
CDCl3
565 MHz
10 0 -10 -20 -30 -40 -50 -60 -70 -80 -90 -100 -110 -120 -130 -140 -150 -160 -170 -180 -190 -200 -210
f1 (ppm)

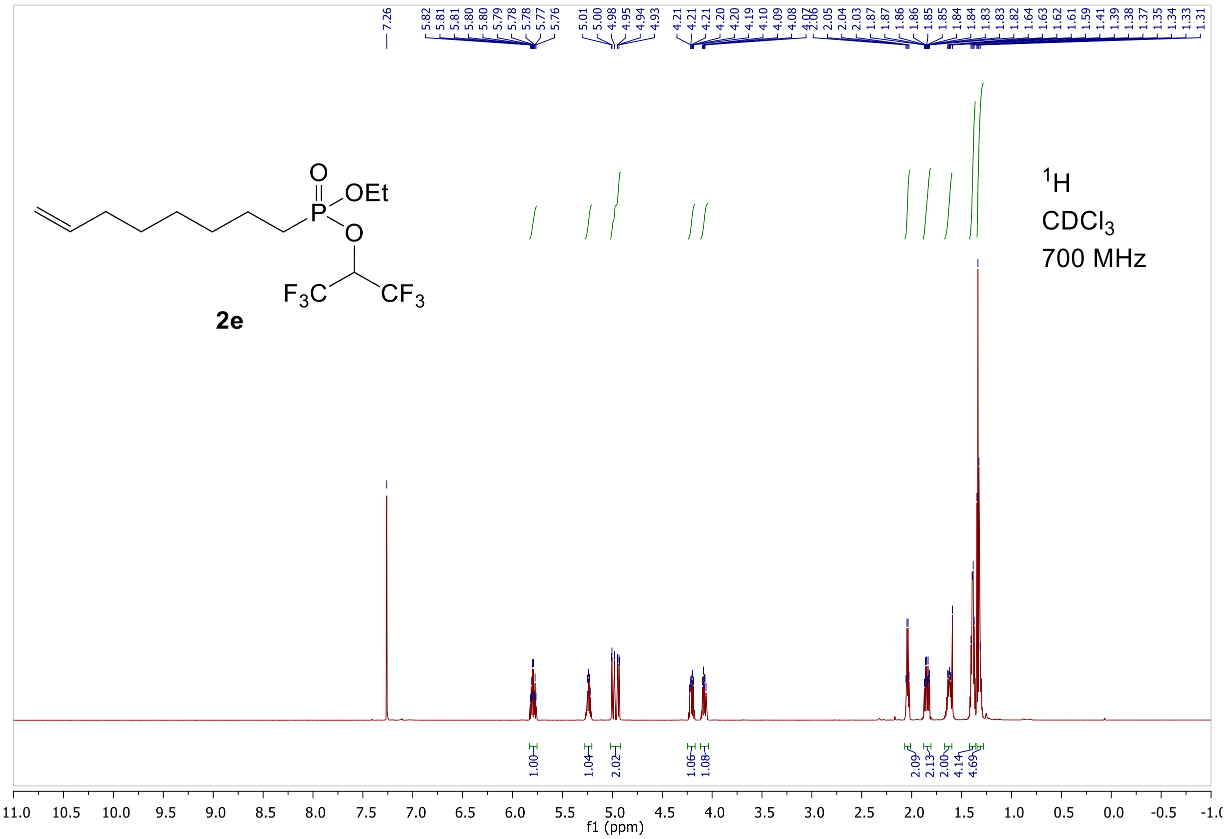
7.26
5.82 5.81 5.81 5.80 5.79 5.78 5.78 5.77 5.76
5.01 5.00 4.98 4.95 4.94 4.93
4.21 4.21 4.21 4.20 4.20 4.19 4.10 4.09 4.08
2.05 2.04 2.03 1.87 1.87 1.86 1.85 1.85 1.84 1.84 1.83 1.83 1.82 1.64 1.63 1.62 1.61 1.59 1.41 1.39 1.38 1.37 1.35 1.34 1.33 1.31
O
OEt
P
O
F3C
CF3
2e
1H
CDCl3
700 MHz
1.00 1.04 2.02 1.06 1.08 2.09 2.13 2.00 4.14 4.69
11.0 10.5 10.0 9.5 9.0 8.5 8.0 7.5 7.0 6.5 6.0 5.5 5.0 4.5 4.0 3.5 3.0 2.5 2.0 1.5 1.0 0.5 0.0 -0.5
f1 (ppm)

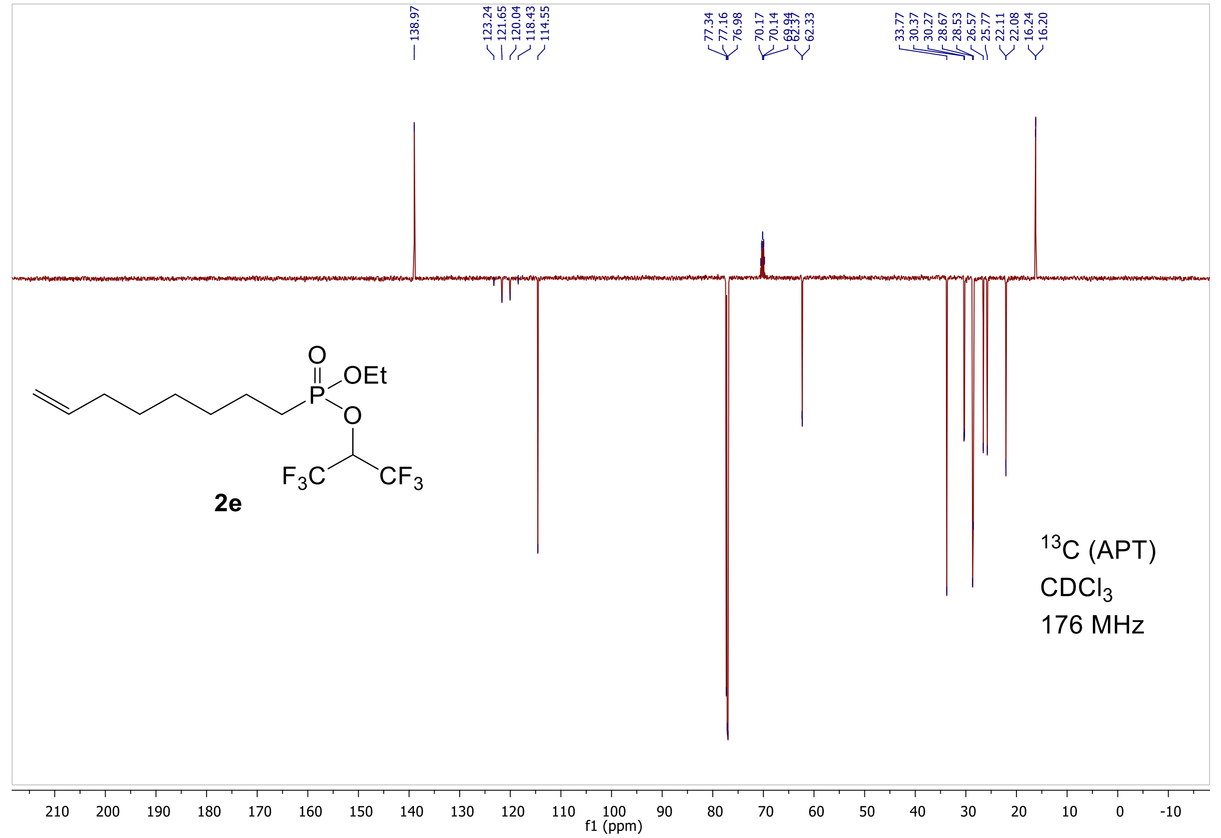
138.97
123.24 121.65 120.04 118.43 114.55
77.34 77.16 76.98 70.17 70.14 62.33
33.77 30.37 30.27 28.67 28.53 26.57 25.77 22.11 22.08 16.24 16.20
O
OEt
P
O
F3C
CF3
2e
13C (APT)
CDCl3
176 MHz
210 200 190 180 170 160 150 140 130 120 110 100 90 80 70 60 50 40 30 20 10 0 -10
f1 (ppm)

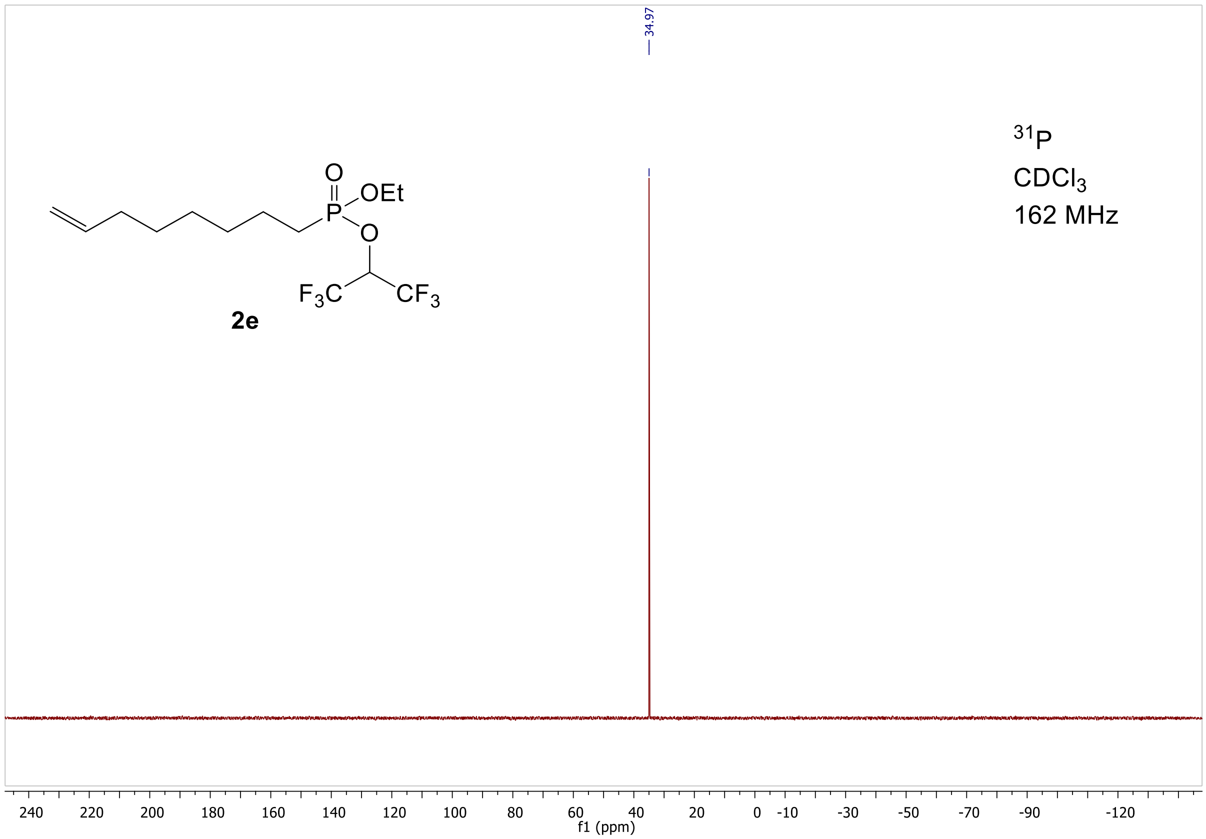

34.97
O
OEt
P
O
F3C
CF3
2e
31P
CDCl3
162 MHz
240 220 200 180 160 140 120 100 80 60 40 20 0 -10 -30 -50 -70 -90 -120
f1 (ppm)

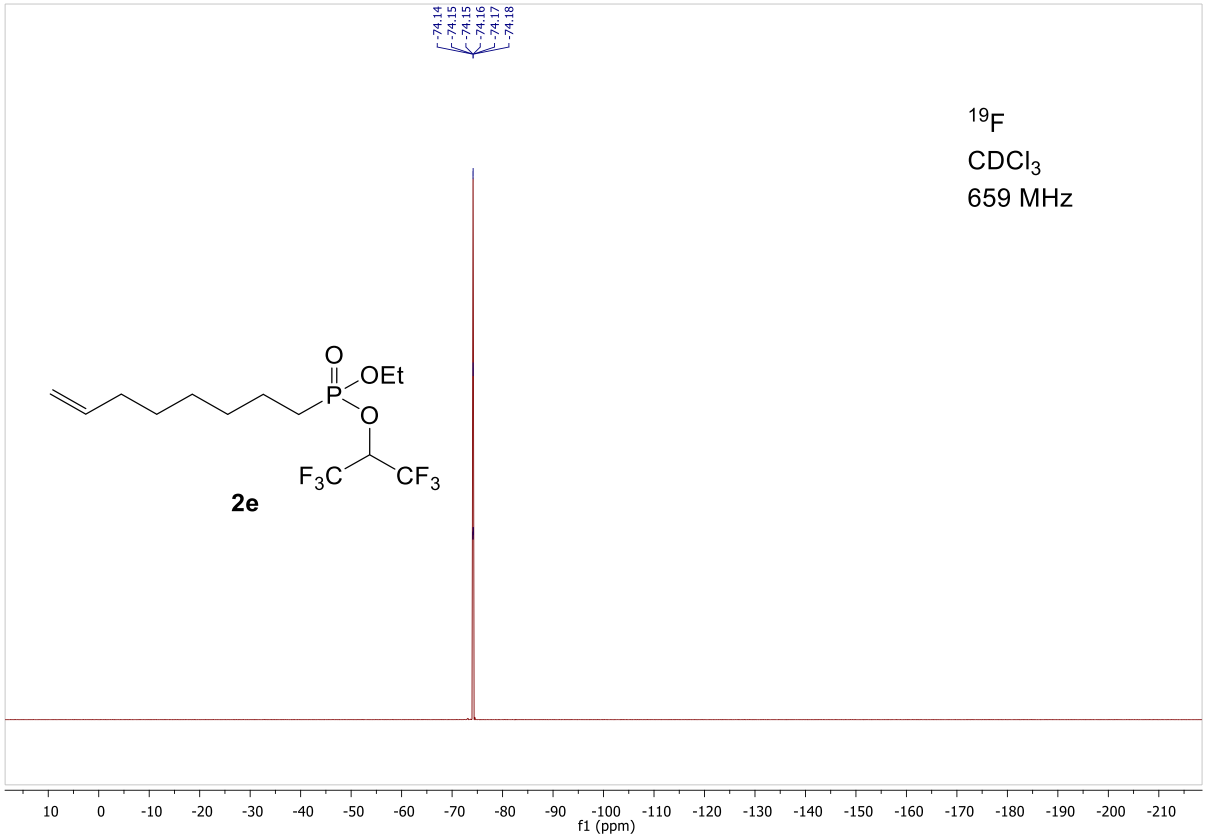

-74.14
-74.15
-74.15
-74.16
-74.17
-74.18
19F
CDCl3
659 MHz
O
OEt
P
O
F3C
CF3
2e
10 0 -10 -20 -30 -40 -50 -60 -70 -80 -90 -100 -110 -120 -130 -140 -150 -160 -170 -180 -190 -200 -210
f1 (ppm)

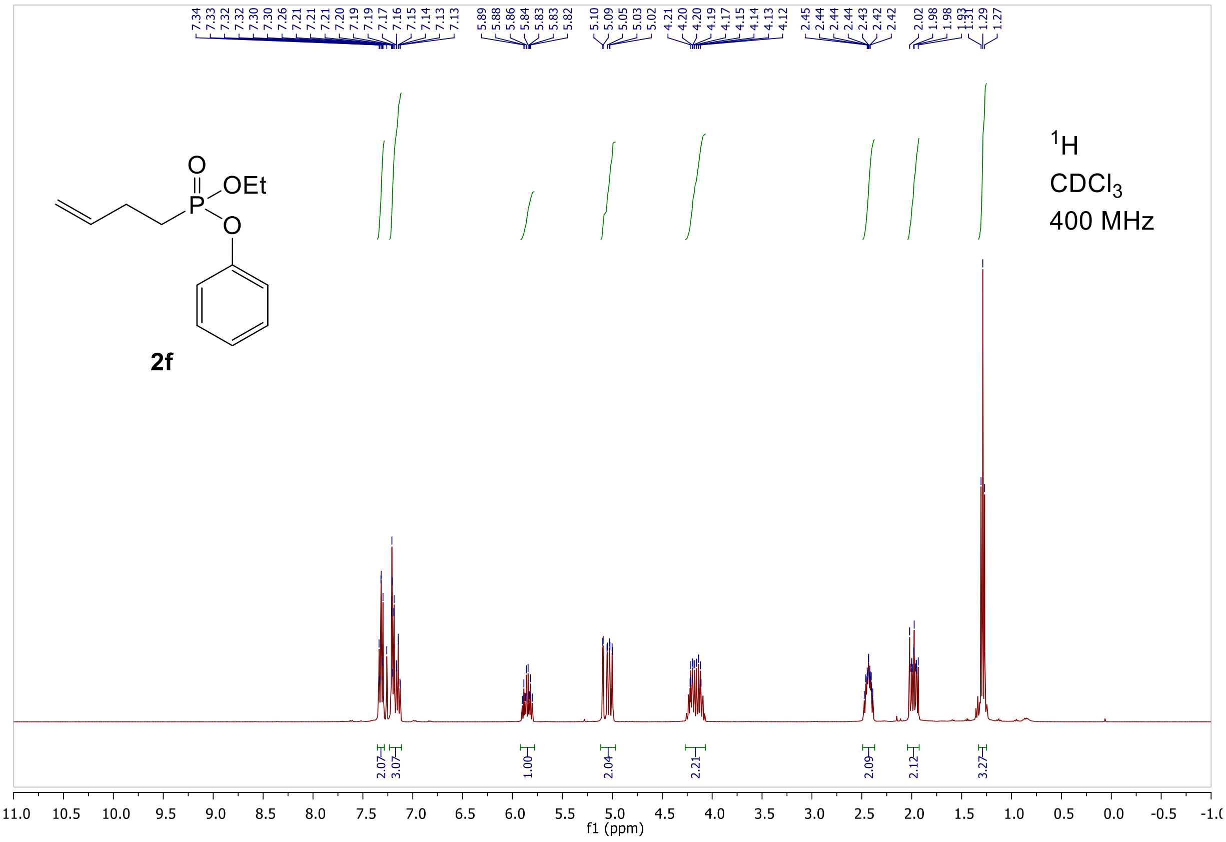

O
OEt
P
O
2f
1H
CDCl3
400 MHz
f1 (ppm)

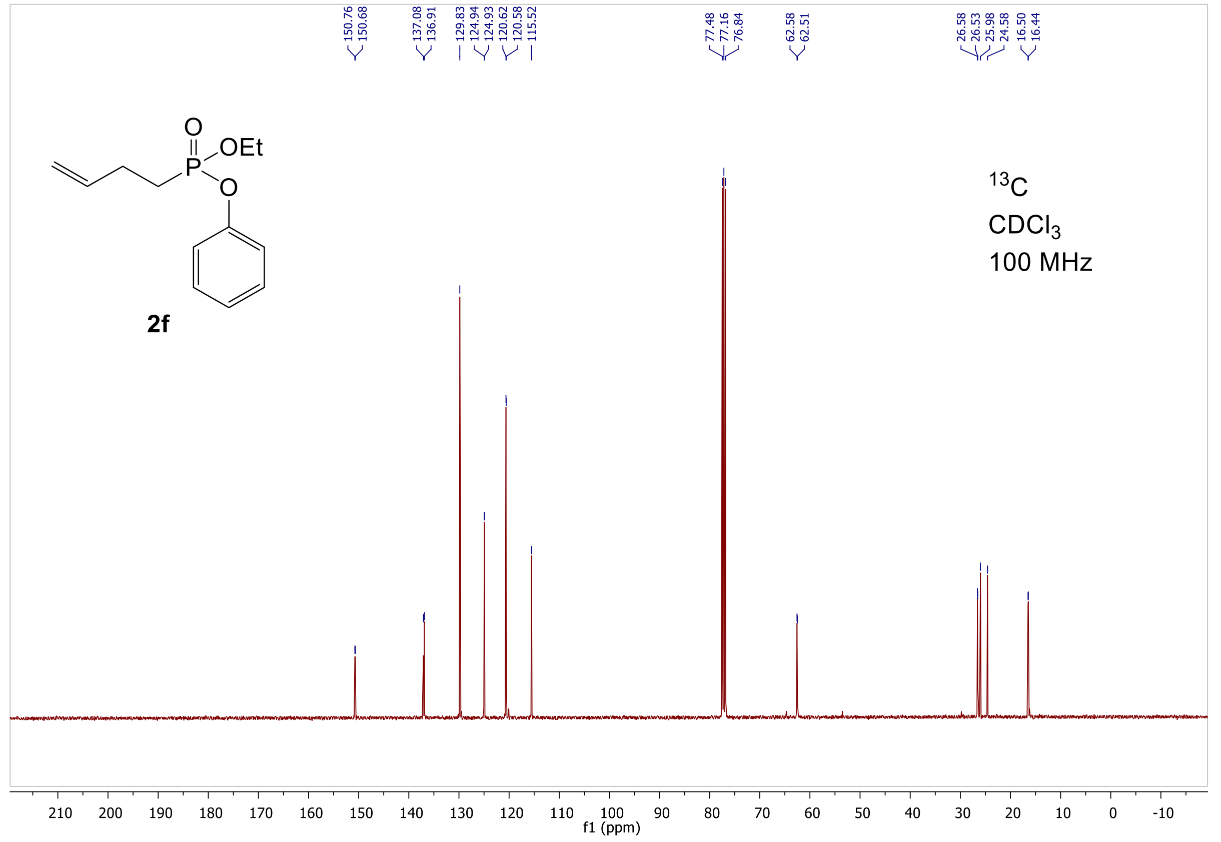

O
OEt
P
O
2f
13C
CDCl3
100 MHz
f1 (ppm)

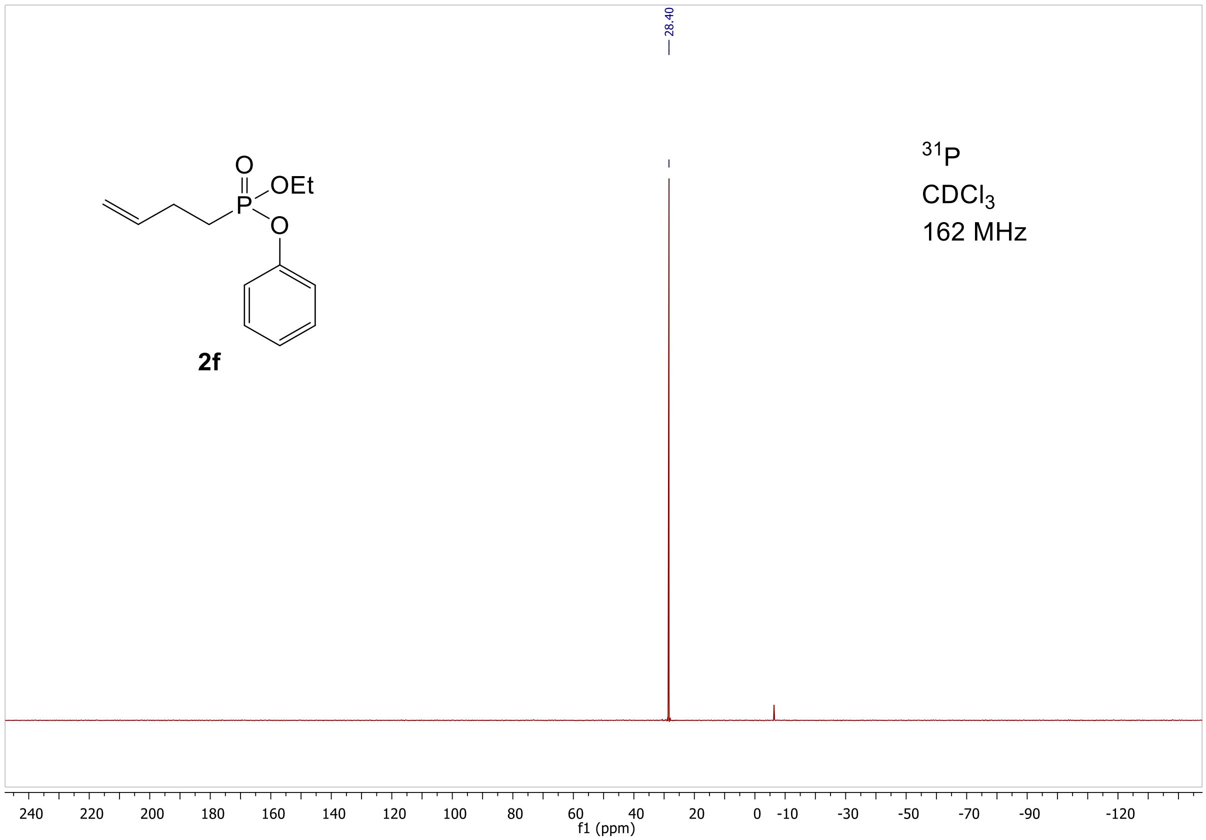
28.40
O
OEt
P
O
2f
^{31}P
CDCl$_3$
162 MHz
240 220 200 180 160 140 120 100 80 60 40 20 0 -10 -30 -50 -70 -90 -120
f1 (ppm)

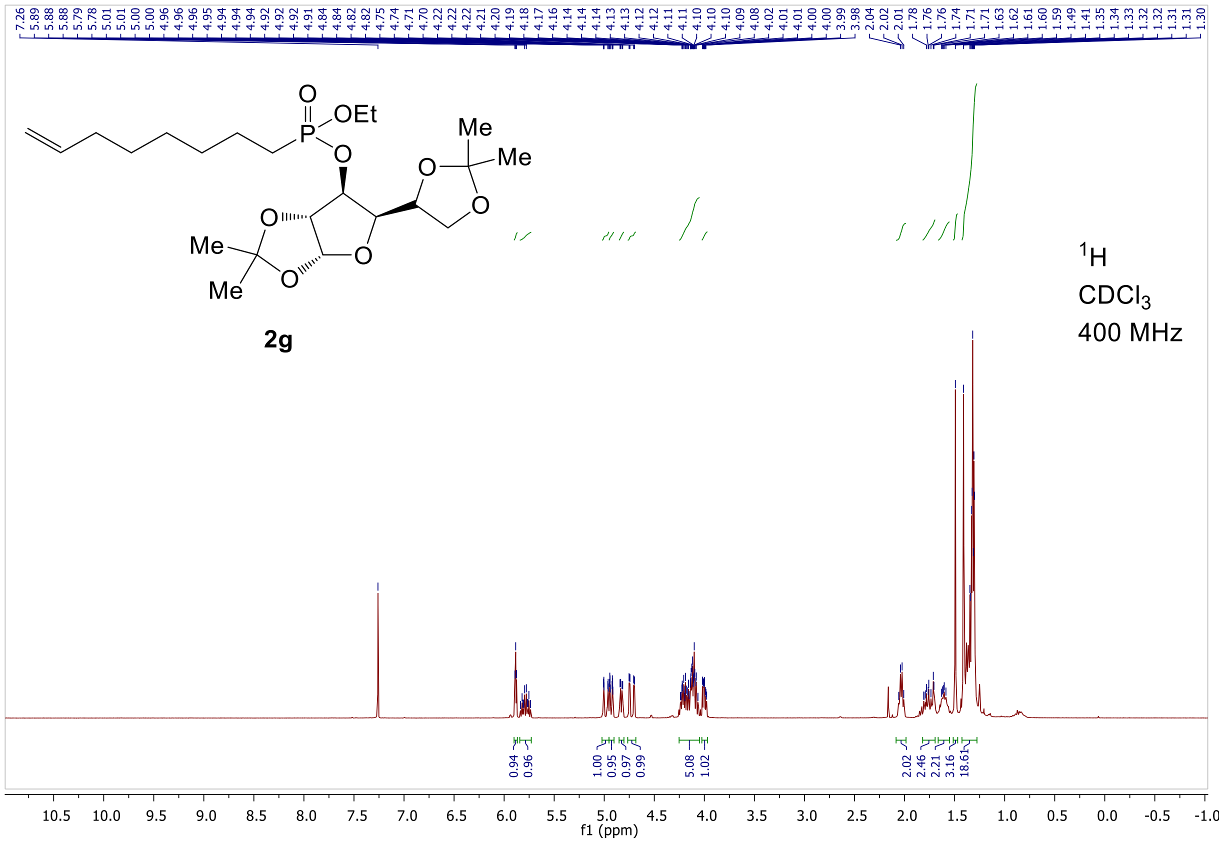

2g
^{1}H
$CDCl_3$
400 MHz
f1 (ppm)

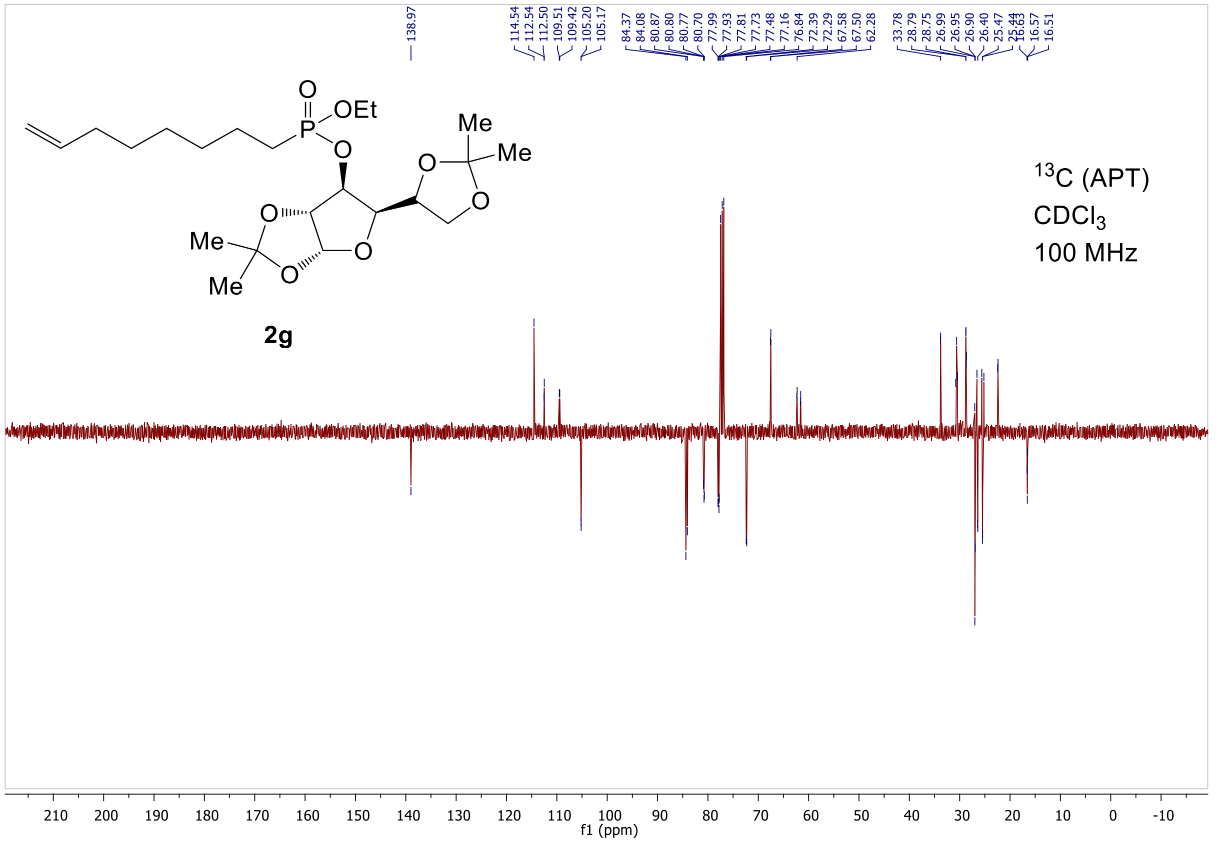

2g
^{13}C (APT)
$CDCl_3$
100 MHz
f1 (ppm)

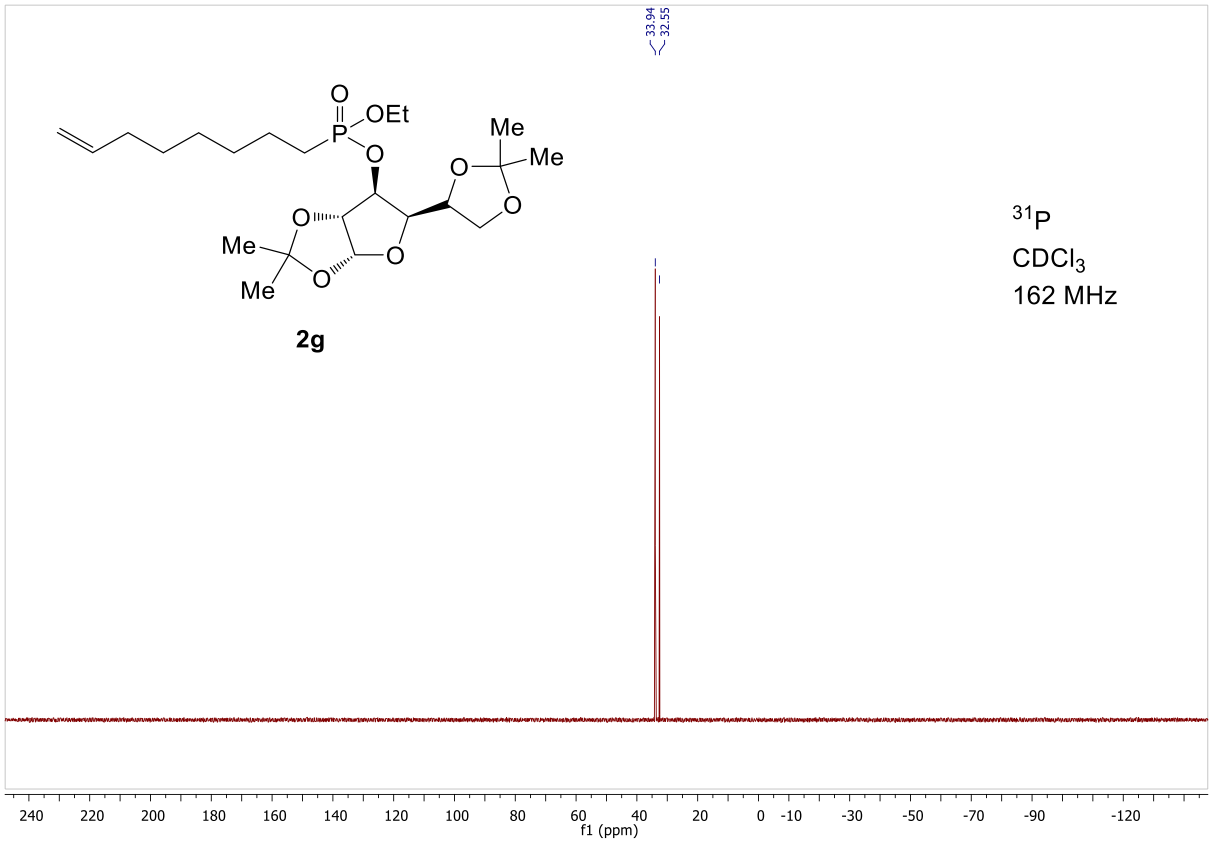
33.94
32.55
O
OEt
P
O
Me
Me
O
O
O
Me
O
Me
O
2g
31P
CDCl3
162 MHz
240 220 200 180 160 140 120 100 80 60 40 20 0 -10 -30 -50 -70 -90 -120
f1 (ppm)

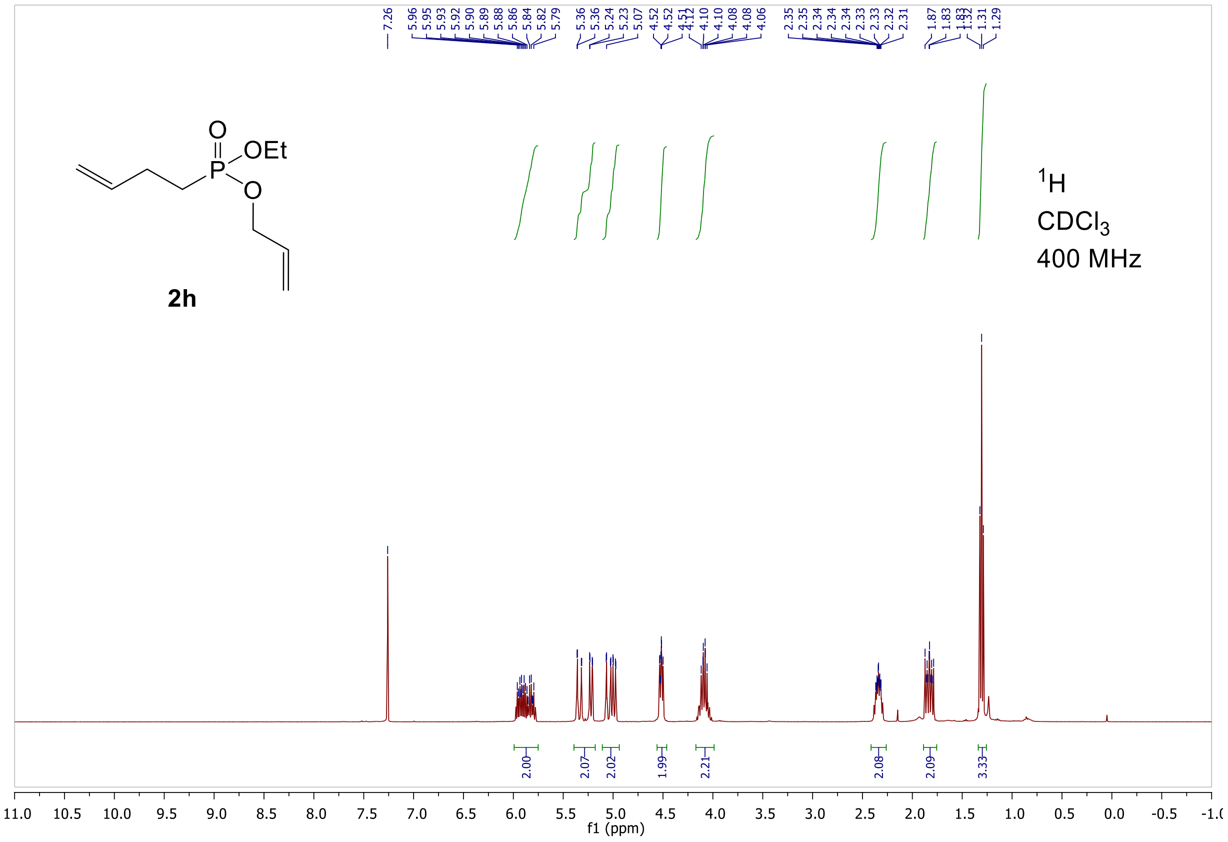
2h
OEt
O
P
O
^{1}H
CDCl$_3$
400 MHz
f1 (ppm)

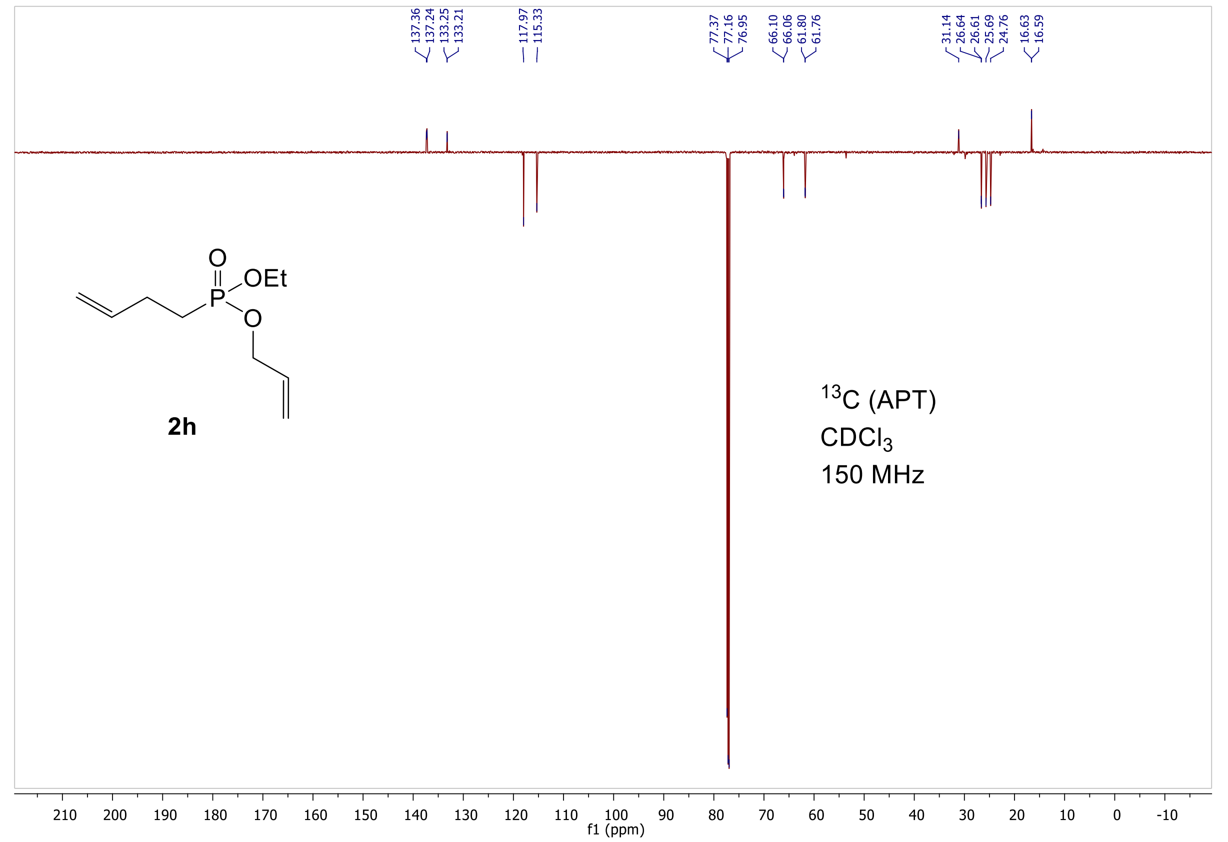
2h
OEt
O
P
O
^{13}C (APT)
CDCl$_3$
150 MHz
f1 (ppm)

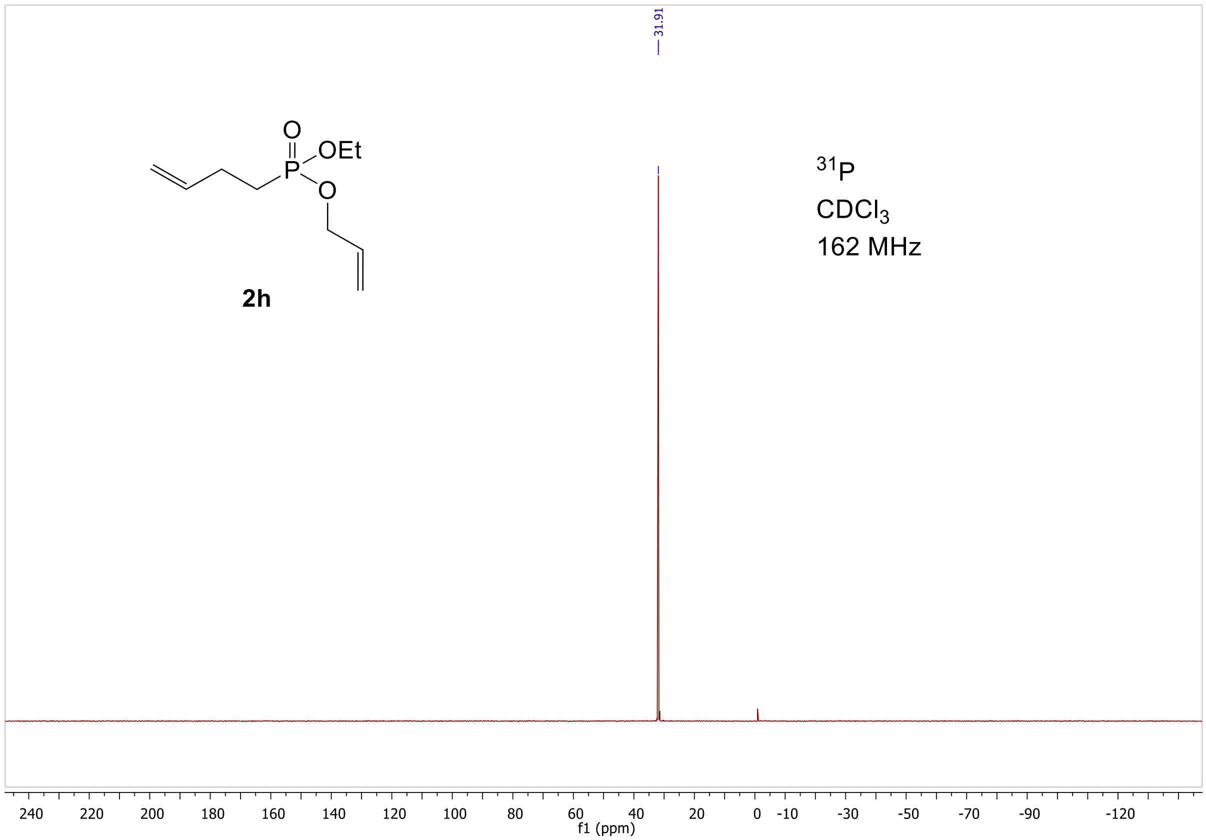

31.91
O
OEt
P
O
2h
^{31}P
$CDCl_3$
162 MHz
240 220 200 180 160 140 120 100 80 60 40 20 0 -10 -30 -50 -70 -90 -120
f1 (ppm)

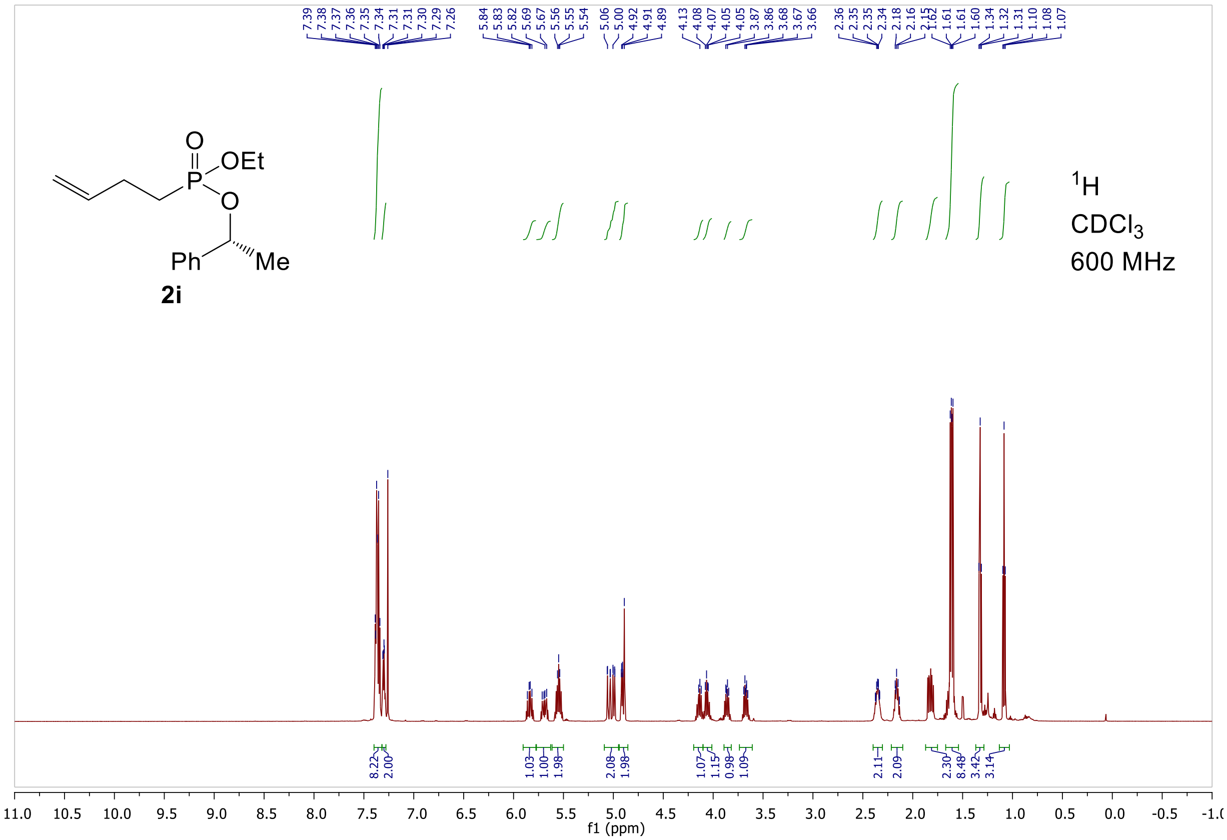
OEt
Ph
Me
2i
^{1}H
$CDCl_3$
600 MHz
f1 (ppm)

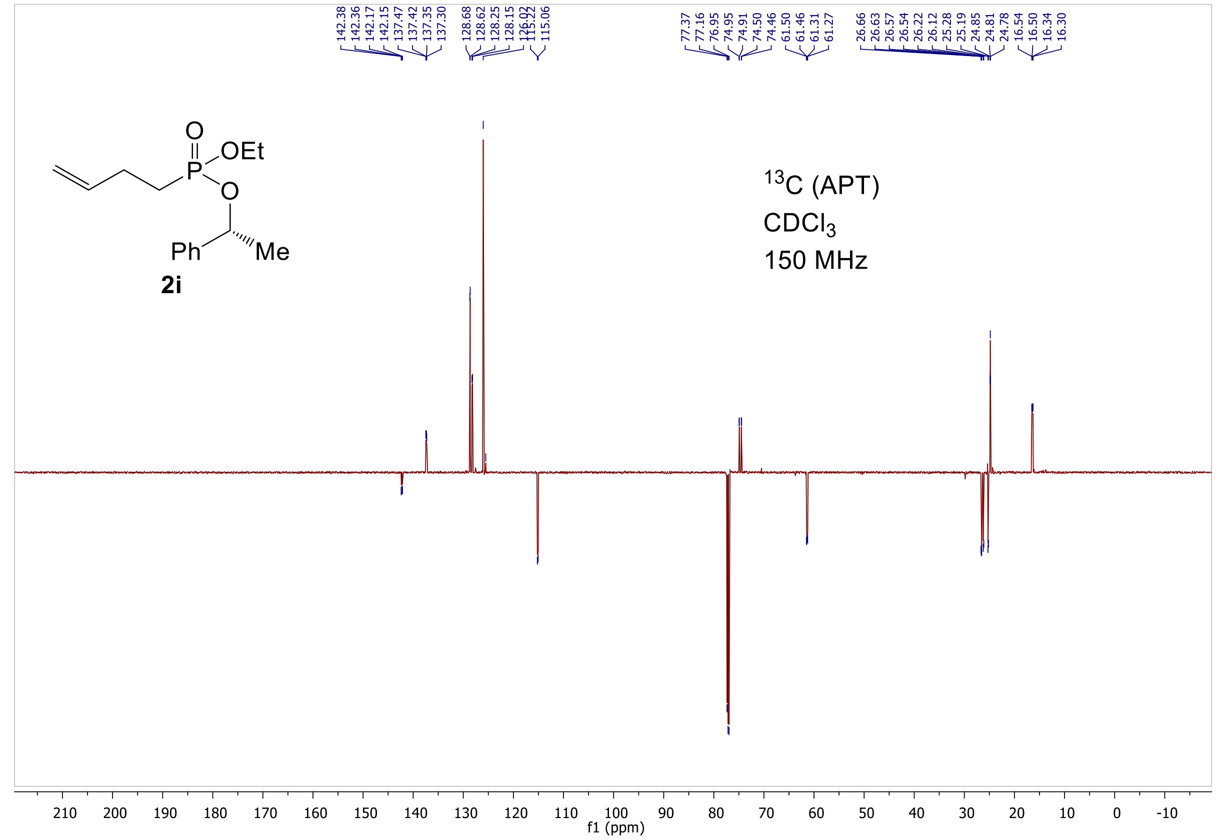
OEt
Ph
Me
2i
^{13}C (APT)
$CDCl_3$
150 MHz
f1 (ppm)

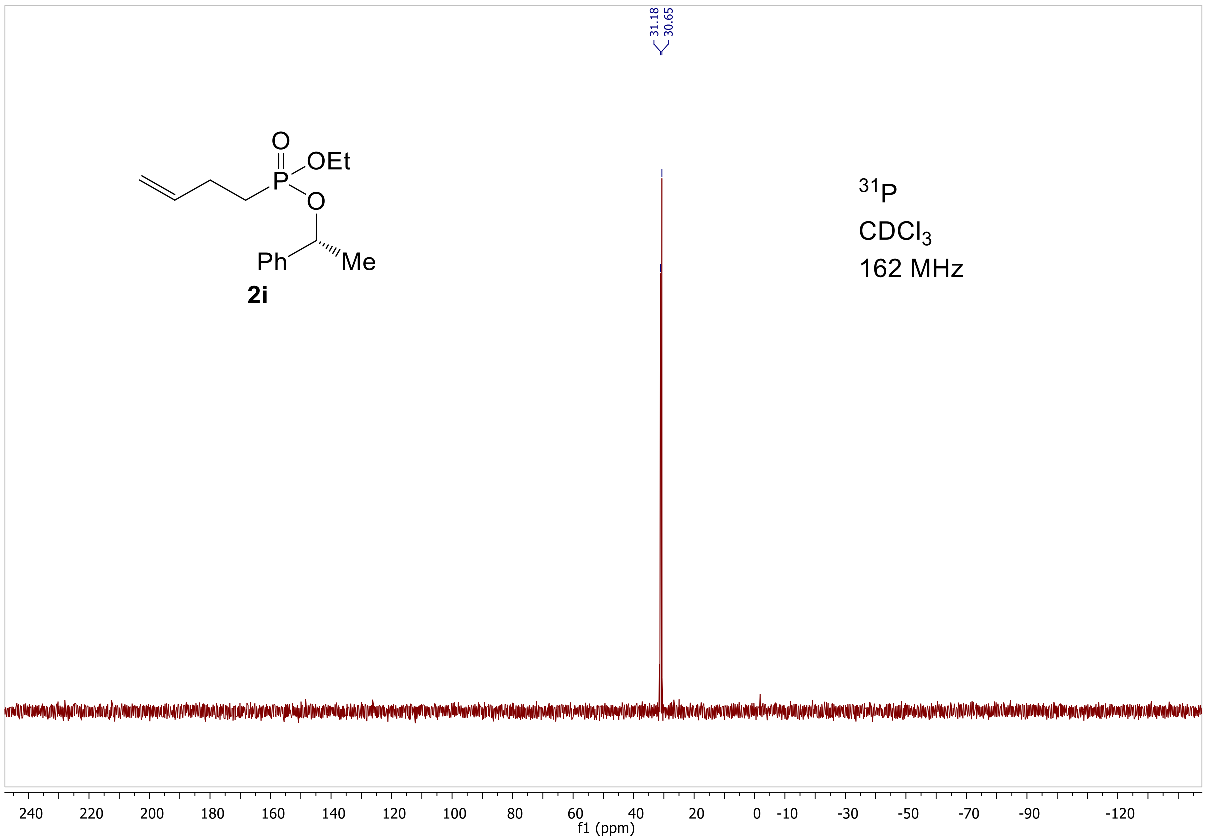
31.18
30.65
O
OEt
P
O
Ph
Me
2i
^{31}P
$CDCl_3$
162 MHz
240 220 200 180 160 140 120 100 80 60 40 20 0 -10 -30 -50 -70 -90 -120
f1 (ppm)

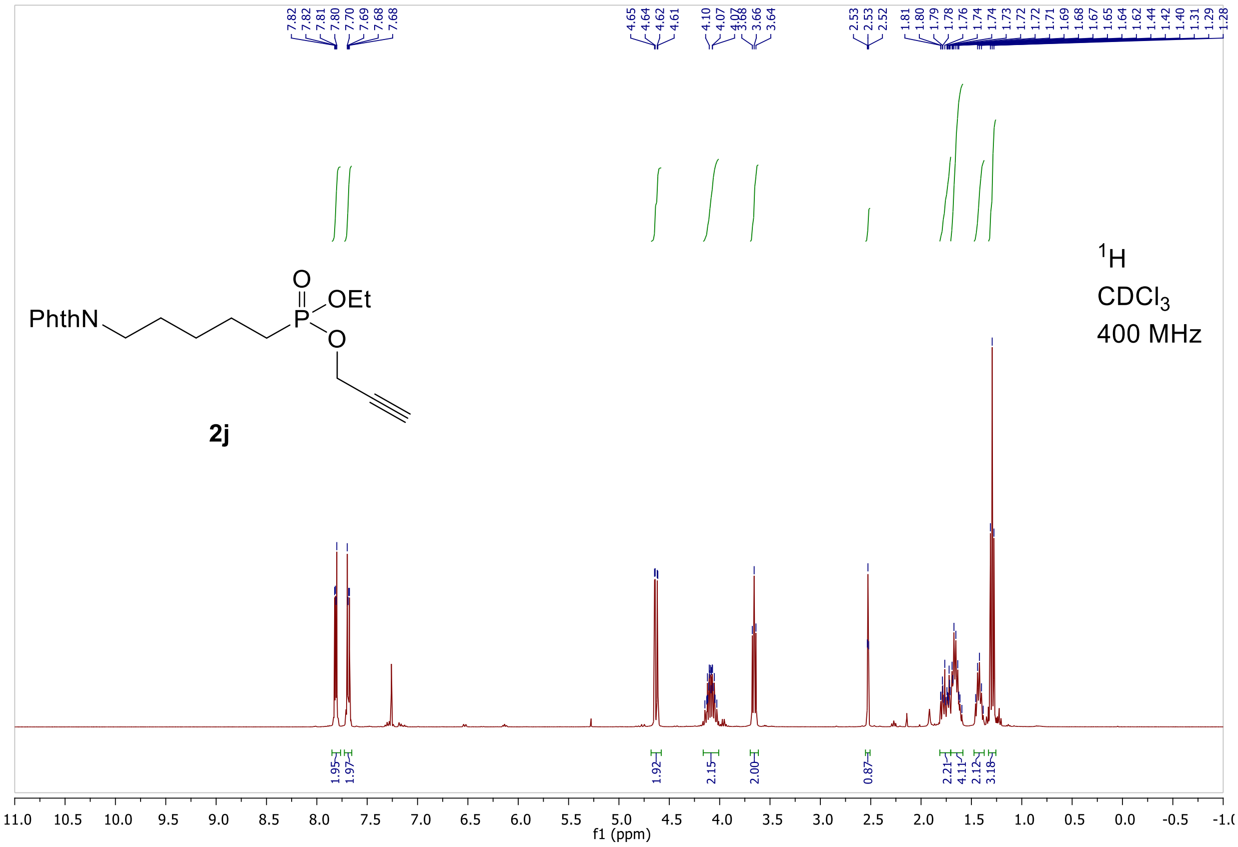
PhthN
OEt
2j
^{1}H
CDCl$_3$
400 MHz
f1 (ppm)

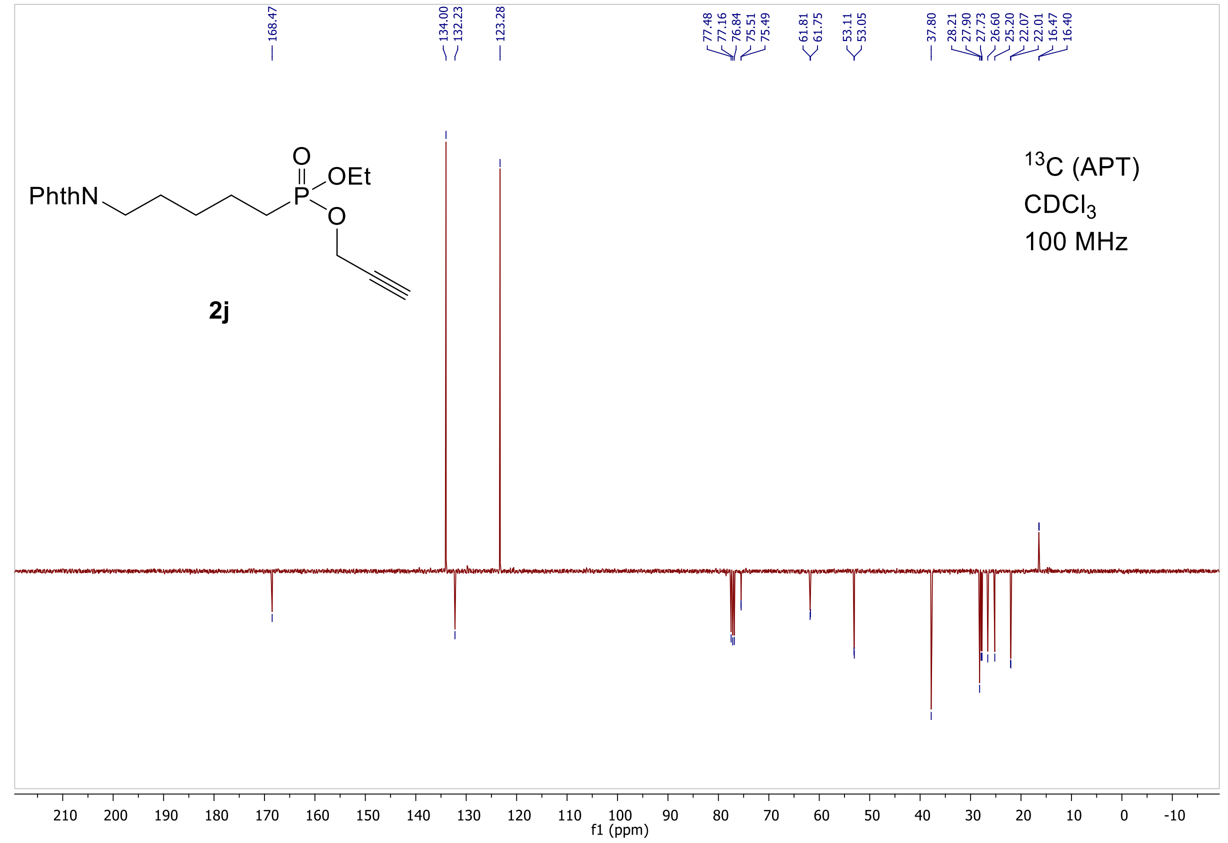
PhthN
OEt
2j
^{13}C (APT)
CDCl$_3$
100 MHz
f1 (ppm)

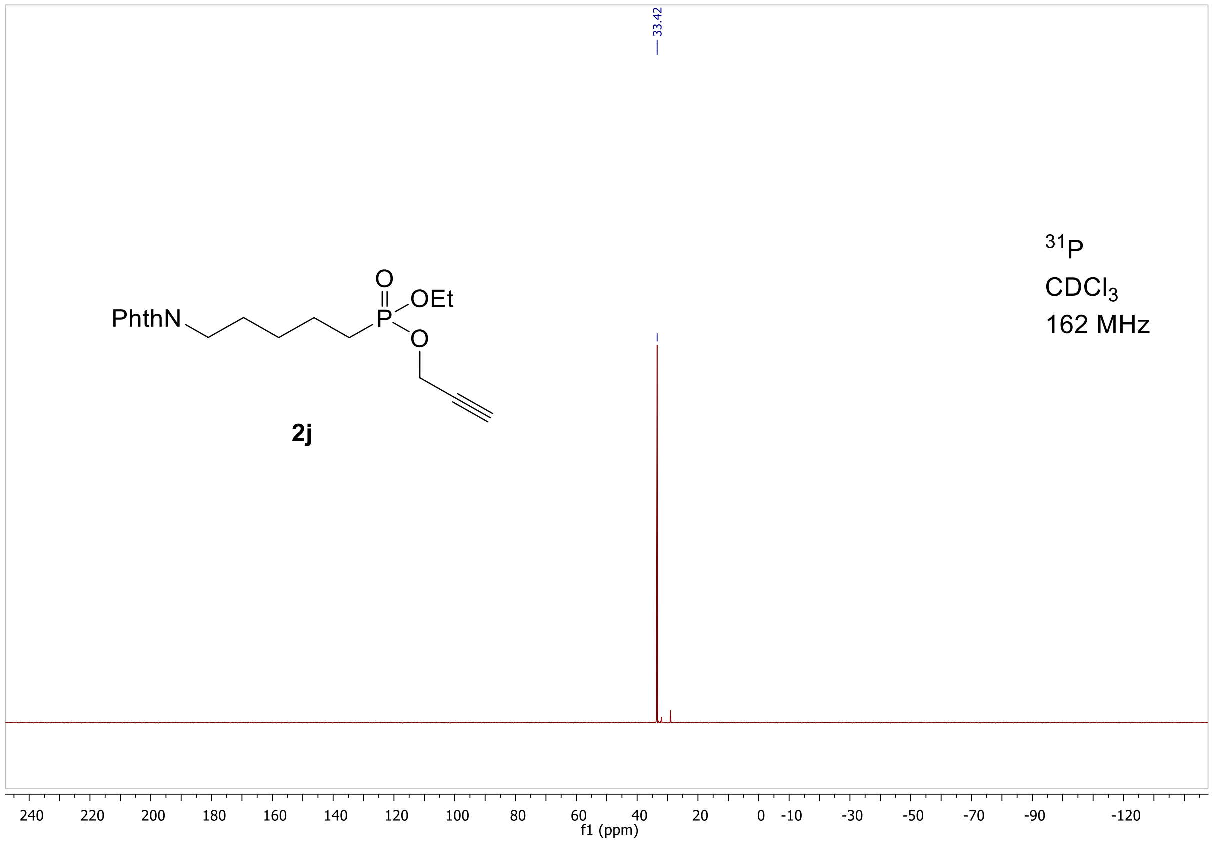
33.42
PhthN
O
OEt
P
O
2j
31P
CDCl3
162 MHz
240
220
200
180
160
140
120
100
80
60
40
20
0
-10
-30
-50
-70
-90
-120
f1 (ppm)

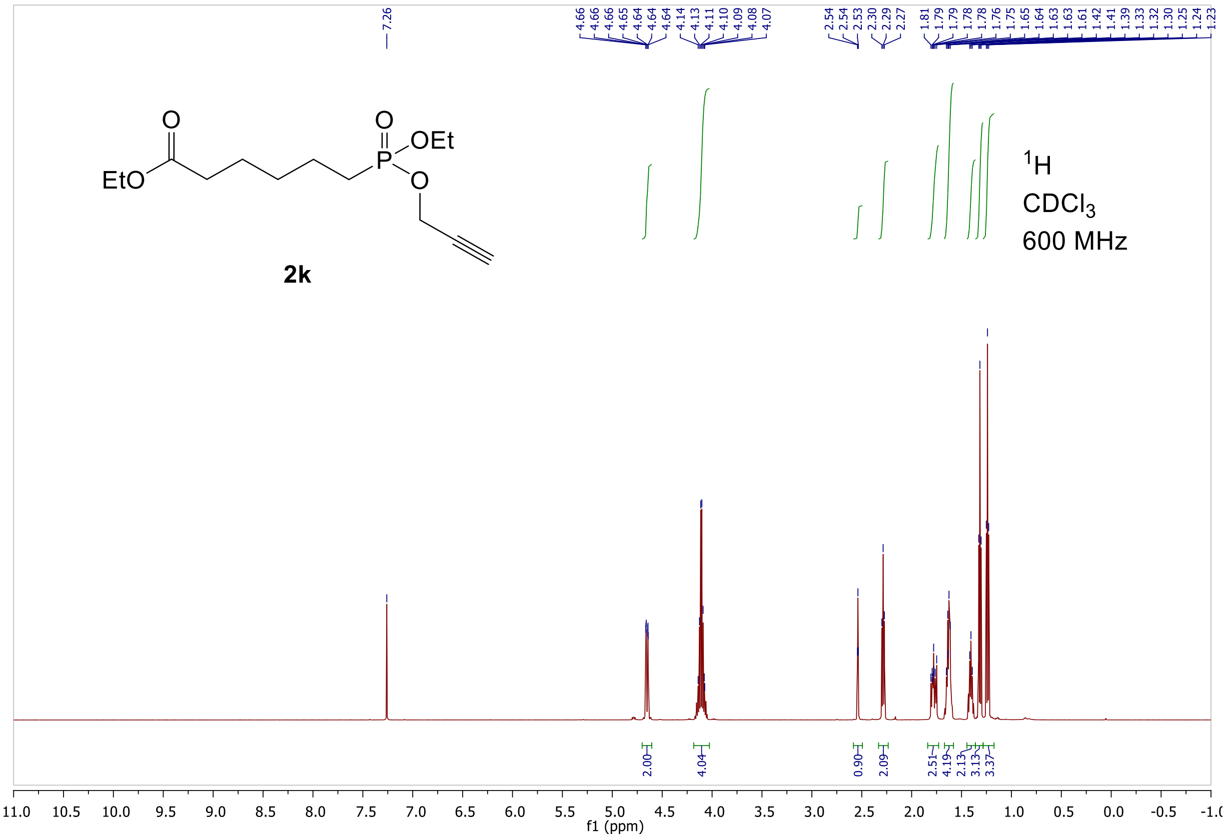

7.26
4.66 4.66 4.66 4.65 4.64 4.64 4.64 4.14 4.13 4.11 4.10 4.09 4.08 4.07
2.54 2.54 2.53 2.30 2.29 2.27
1.81 1.79 1.79 1.78 1.78 1.76 1.75 1.65 1.64 1.63 1.63 1.61 1.42 1.41 1.39 1.33 1.32 1.30 1.25 1.24 1.23
O
O
OEt
EtO
P
O
2k
^{1}H
CDCl$_3$
600 MHz
2.00
4.04
0.90
2.09
2.51
4.19
2.15
3.13
3.37
11.0 10.5 10.0 9.5 9.0 8.5 8.0 7.5 7.0 6.5 6.0 5.5 5.0 4.5 4.0 3.5 3.0 2.5 2.0 1.5 1.0 0.5 0.0 -0.5
f1 (ppm)

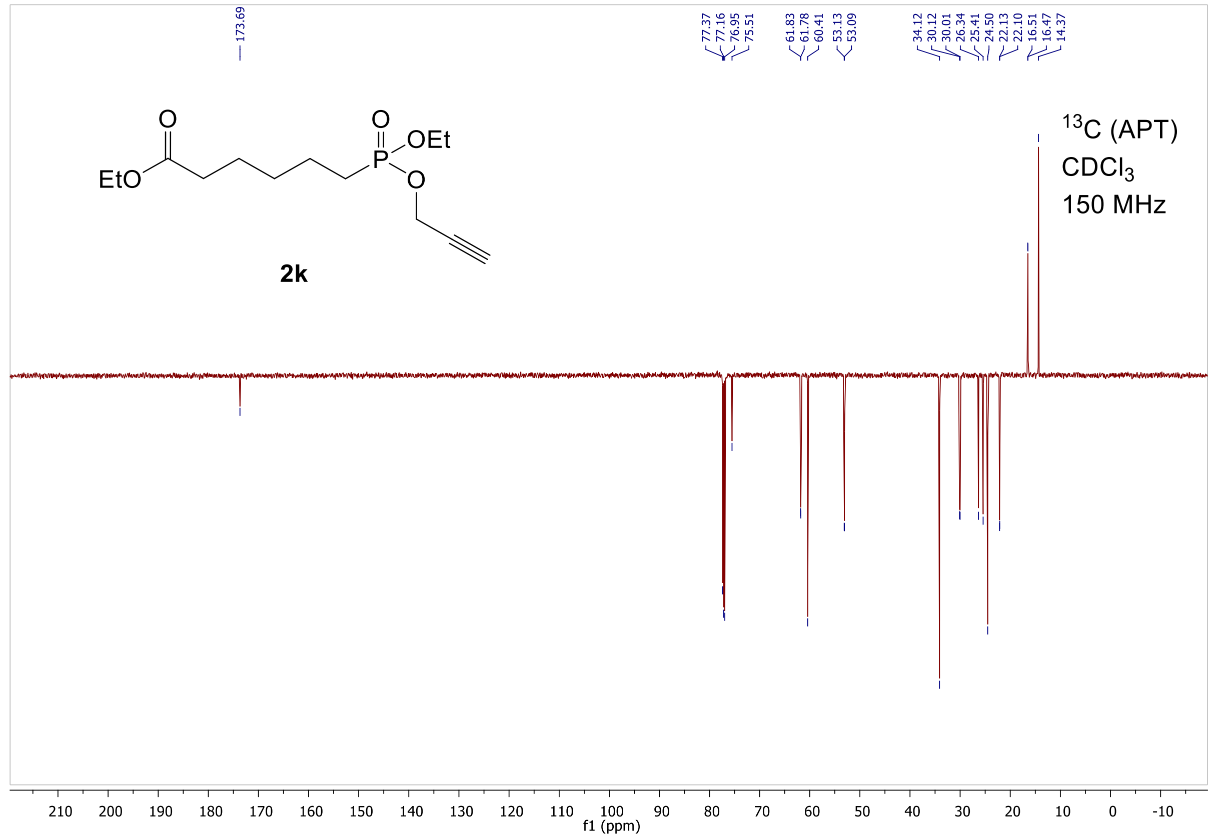

173.69
77.37 77.16 76.95 75.51
61.83 61.78 60.41 53.13 53.09
34.12 30.12 30.01 26.34 25.41 24.50 22.13 22.10 16.51 16.47 14.37
O
O
OEt
EtO
P
O
2k
^{13}C (APT)
CDCl$_3$
150 MHz
210 200 190 180 170 160 150 140 130 120 110 100 90 80 70 60 50 40 30 20 10 0 -10
f1 (ppm)

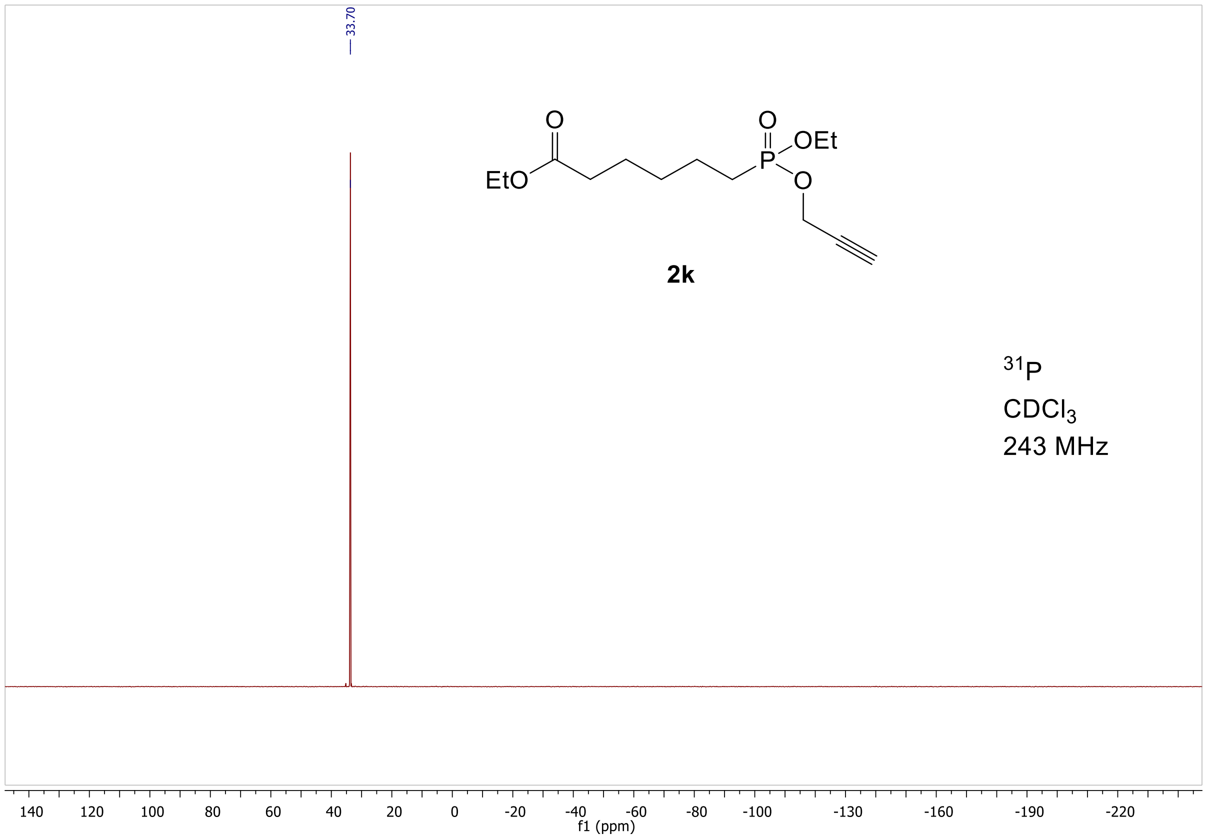

33.70
EtO
O
O
P
OEt
O
2k
^{31}P
$CDCl_3$
243 MHz
140 120 100 80 60 40 20 0 -20 -40 -60 -80 -100 -130 -160 -190 -220
f1 (ppm)

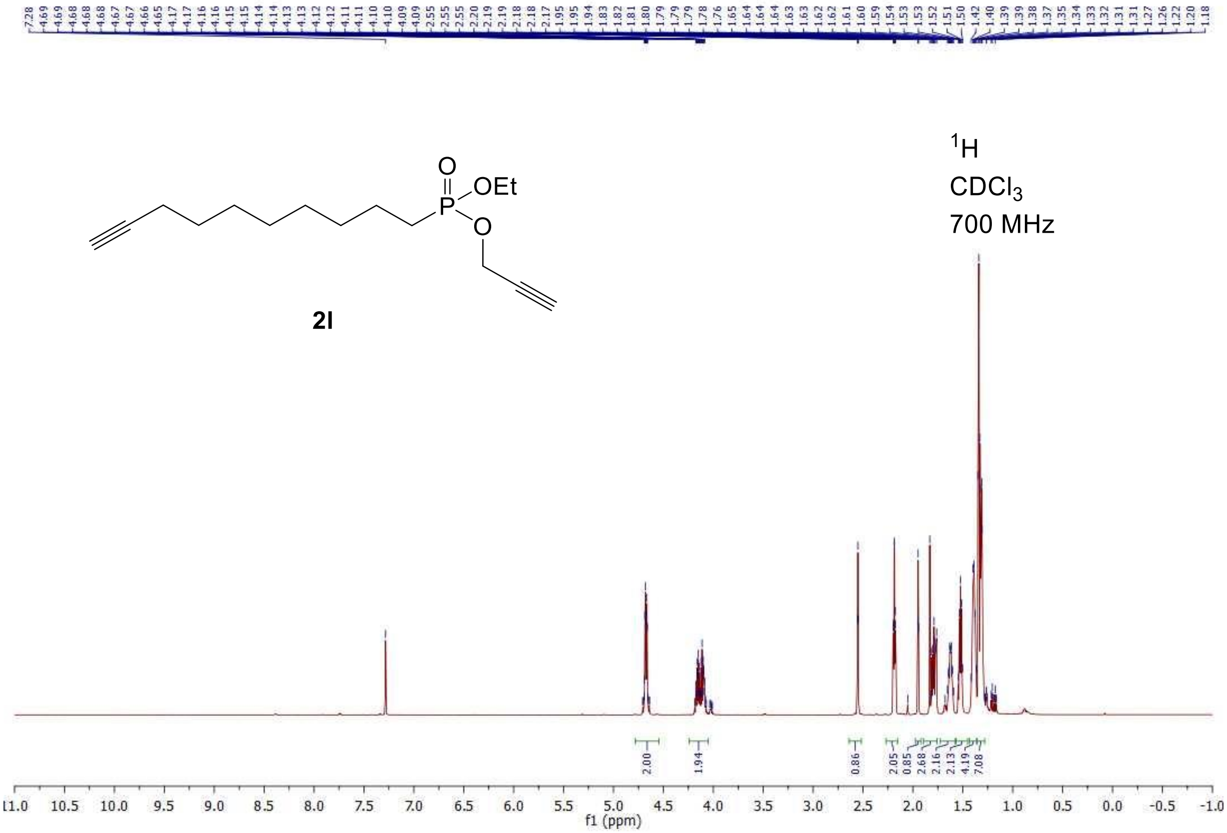

O
OEt
P
O
2l
^{1}H
$CDCl_3$
700 MHz
2.00
1.94
0.86
2.05
0.85
2.68
2.16
2.13
4.19
7.08
f1 (ppm)

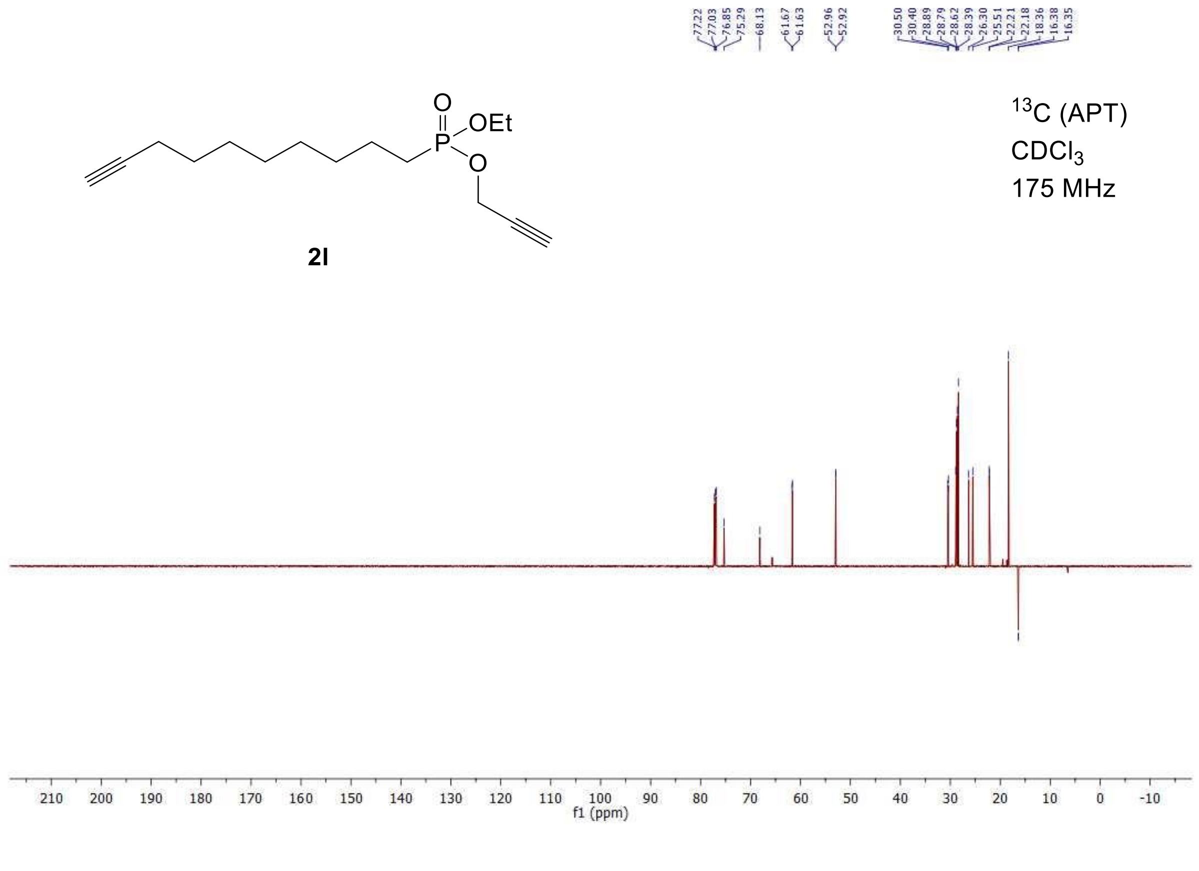

77.22
77.03
76.85
75.29
68.13
61.67
61.63
52.96
52.92
30.50
30.40
28.89
28.79
28.62
28.39
26.30
25.51
22.21
22.18
18.36
16.38
16.35
O
OEt
P
O
2l
^{13}C (APT)
$CDCl_3$
175 MHz
f1 (ppm)

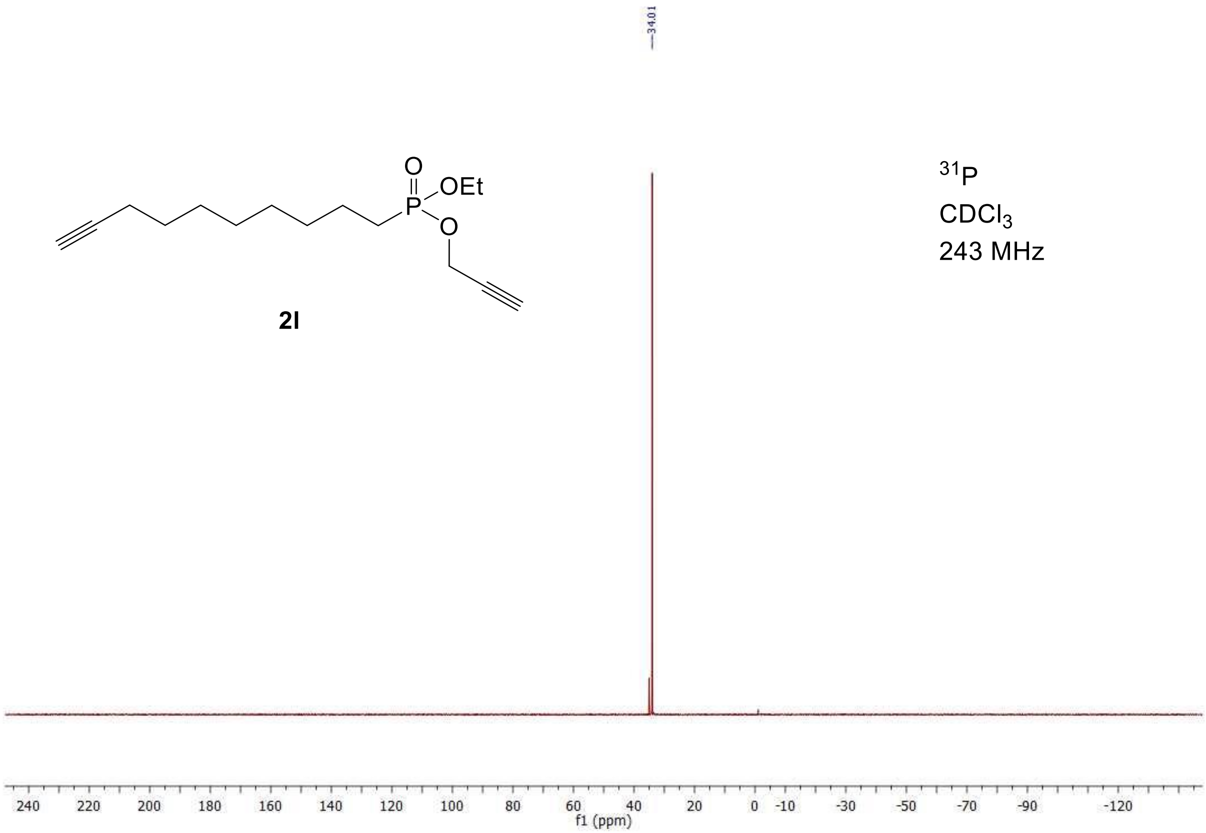

OEt
2l
34.01
^{31}P
$CDCl_3$
243 MHz
240
220
200
180
160
140
120
100
80
60
40
20
0
-10
-30
-50
-70
-90
-120
f1 (ppm)

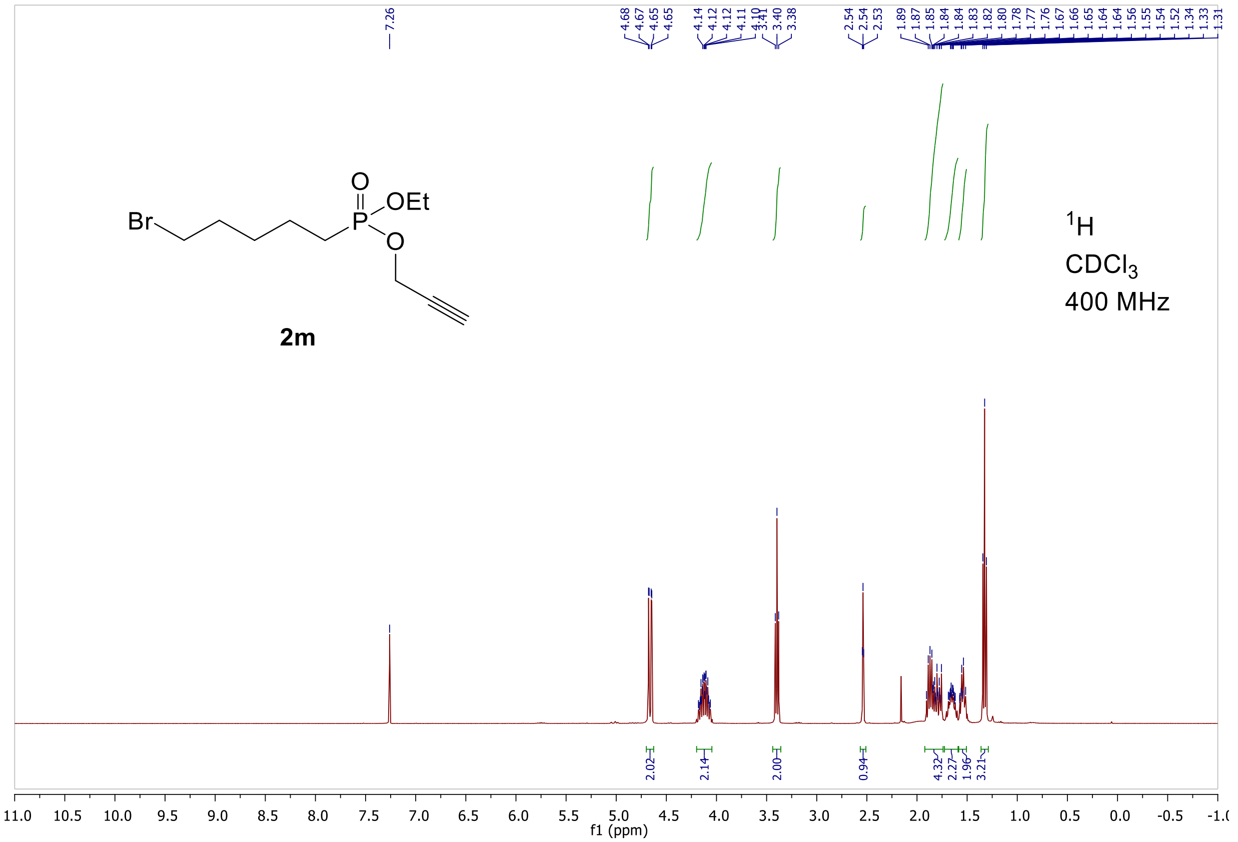

2m
1H
CDCl3
400 MHz
f1 (ppm)

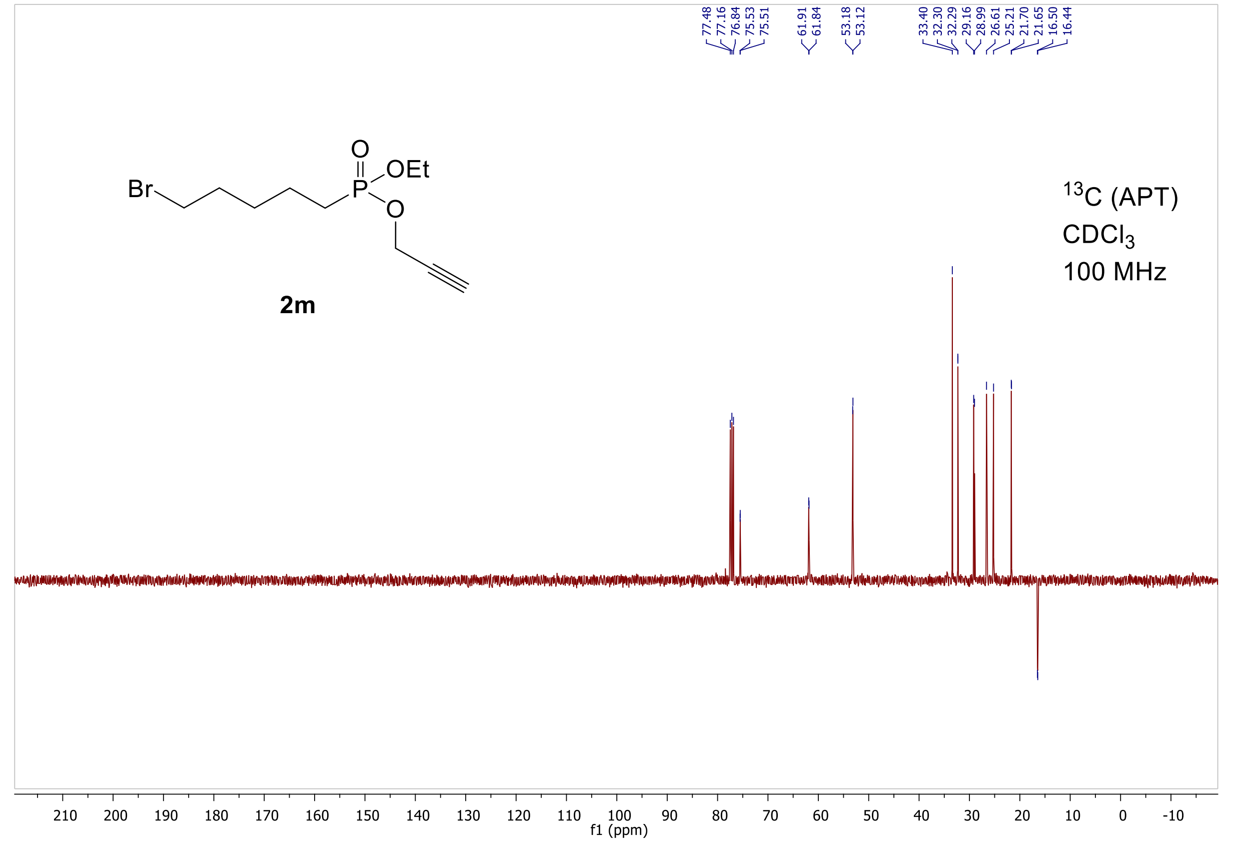

2m
13C (APT)
CDCl3
100 MHz
f1 (ppm)

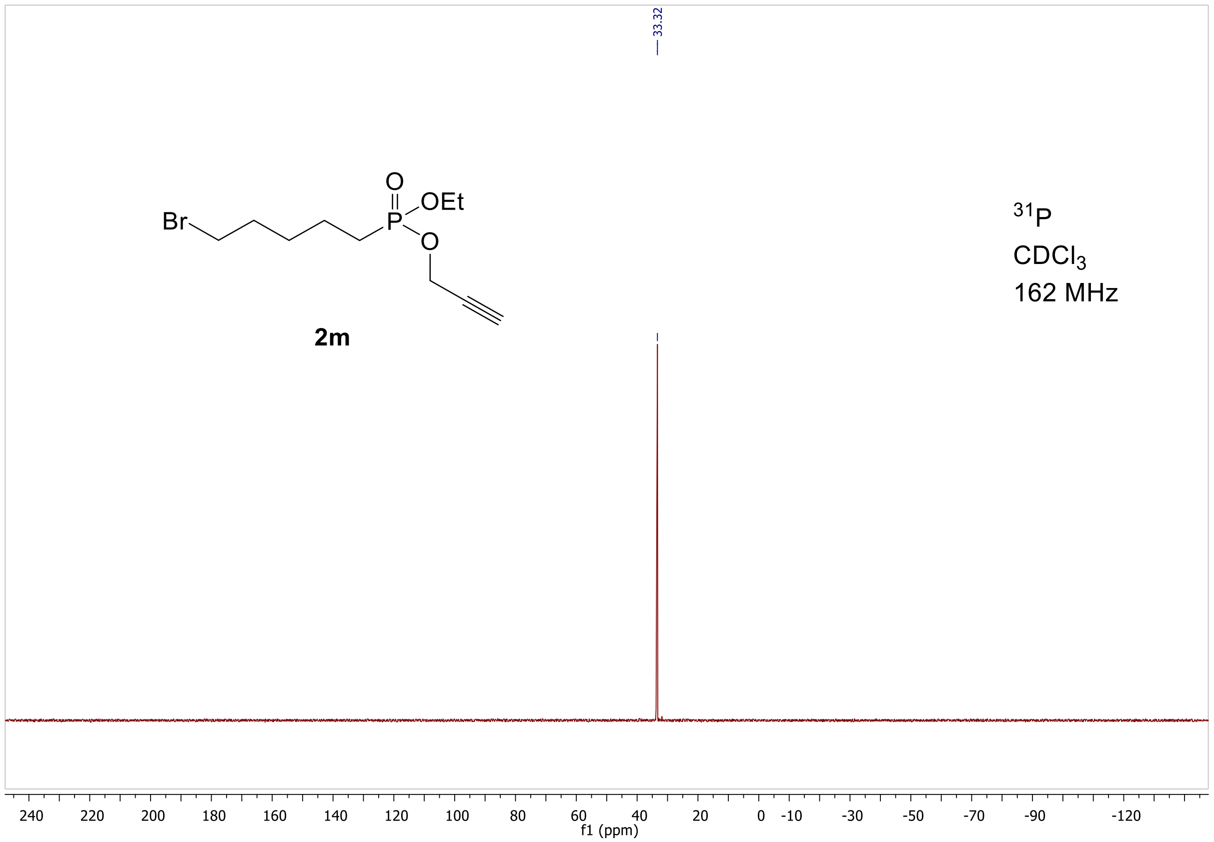

33.32
Br
O
OEt
P
O
2m
^{31}P
CDCl$_3$
162 MHz
240 220 200 180 160 140 120 100 80 60 40 20 0 -10 -30 -50 -70 -90 -120
f1 (ppm)

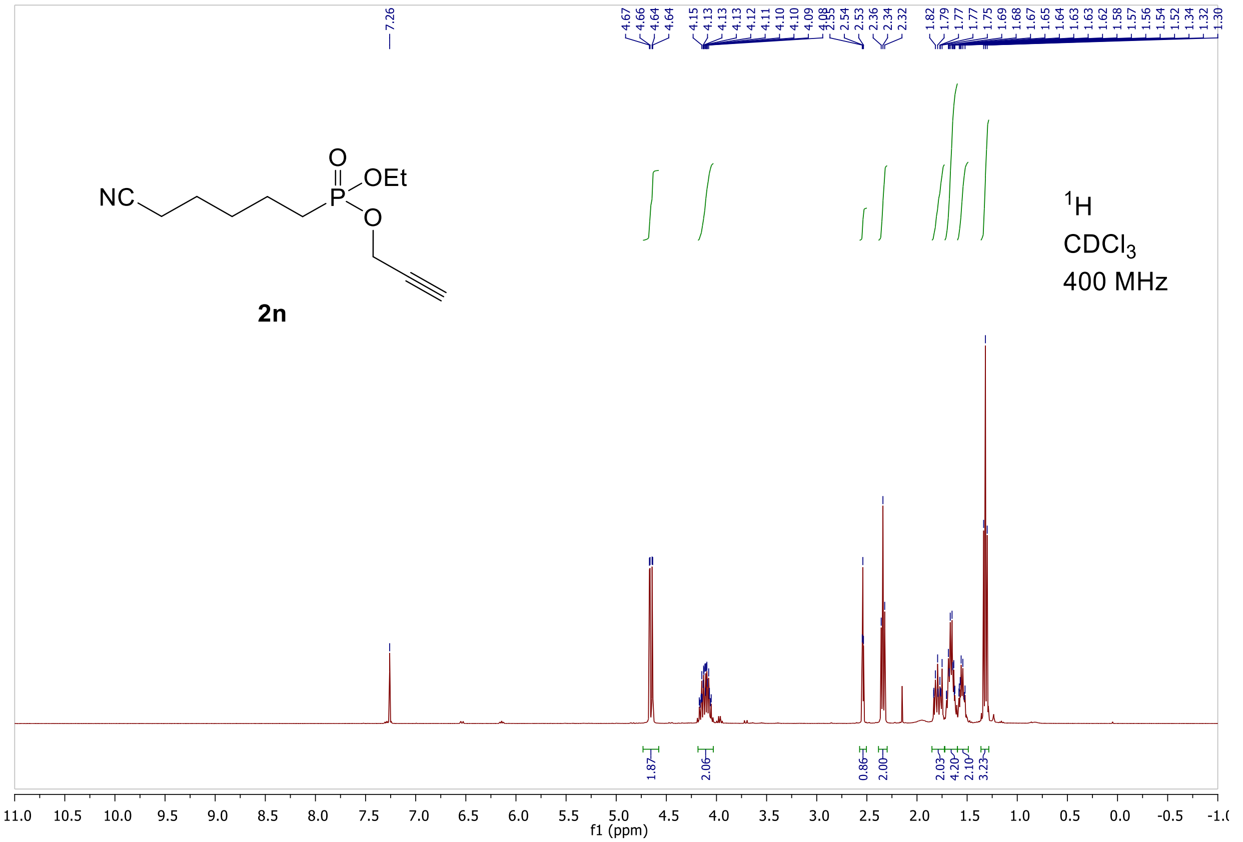
7.26
4.67
4.66
4.64
4.64
4.15
4.13
4.13
4.13
4.12
4.11
4.10
4.10
4.09
4.08
2.55
2.54
2.53
2.36
2.34
2.32
1.82
1.79
1.77
1.77
1.75
1.69
1.68
1.67
1.65
1.64
1.63
1.63
1.62
1.58
1.57
1.56
1.54
1.52
1.34
1.32
1.30
NC
O
P
OEt
O
2n
1H
CDCl3
400 MHz
1.87
2.06
0.86
2.00
2.03
4.20
2.10
3.23
f1 (ppm)

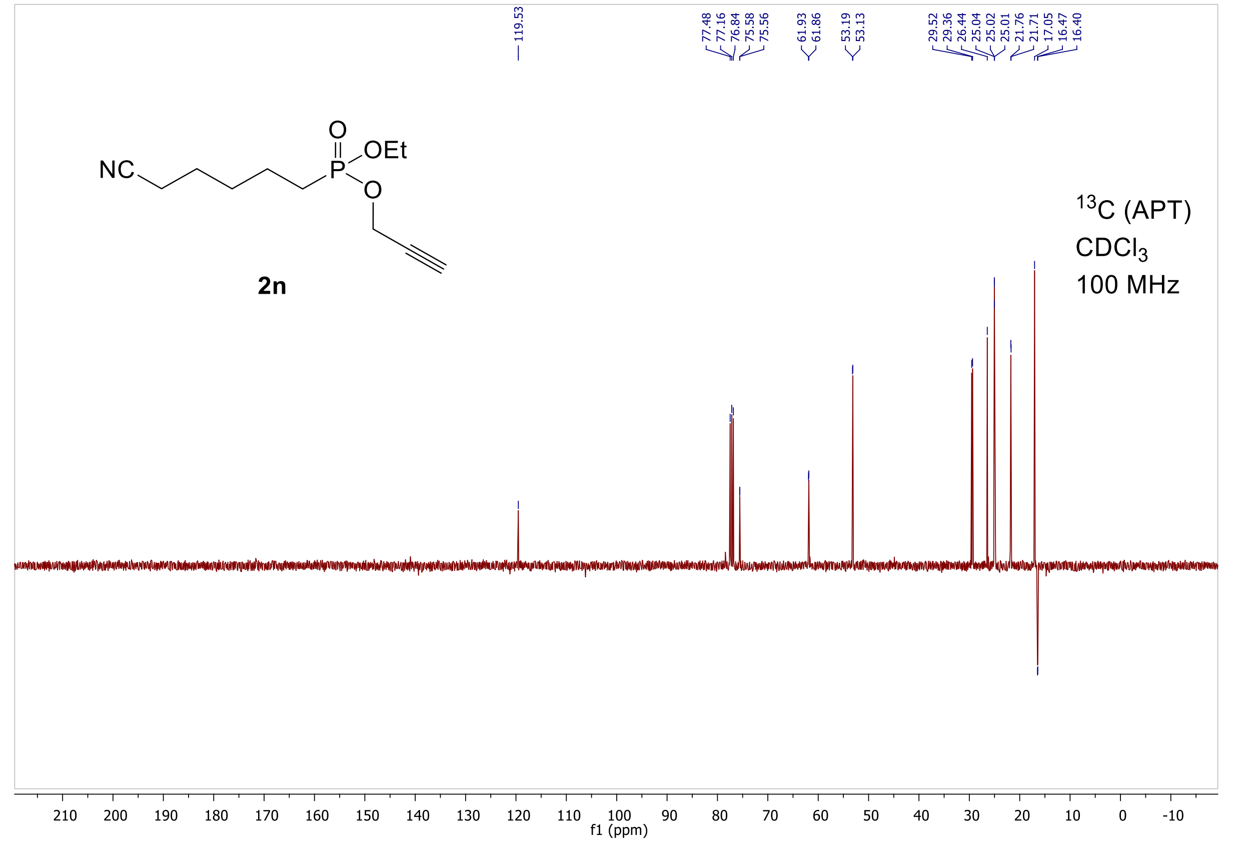
119.53
77.48
77.16
76.84
75.58
75.56
61.93
61.86
53.19
53.13
29.52
29.36
26.44
25.04
25.02
25.01
21.76
21.71
17.05
16.47
16.40
NC
O
P
OEt
O
2n
13C (APT)
CDCl3
100 MHz
f1 (ppm)

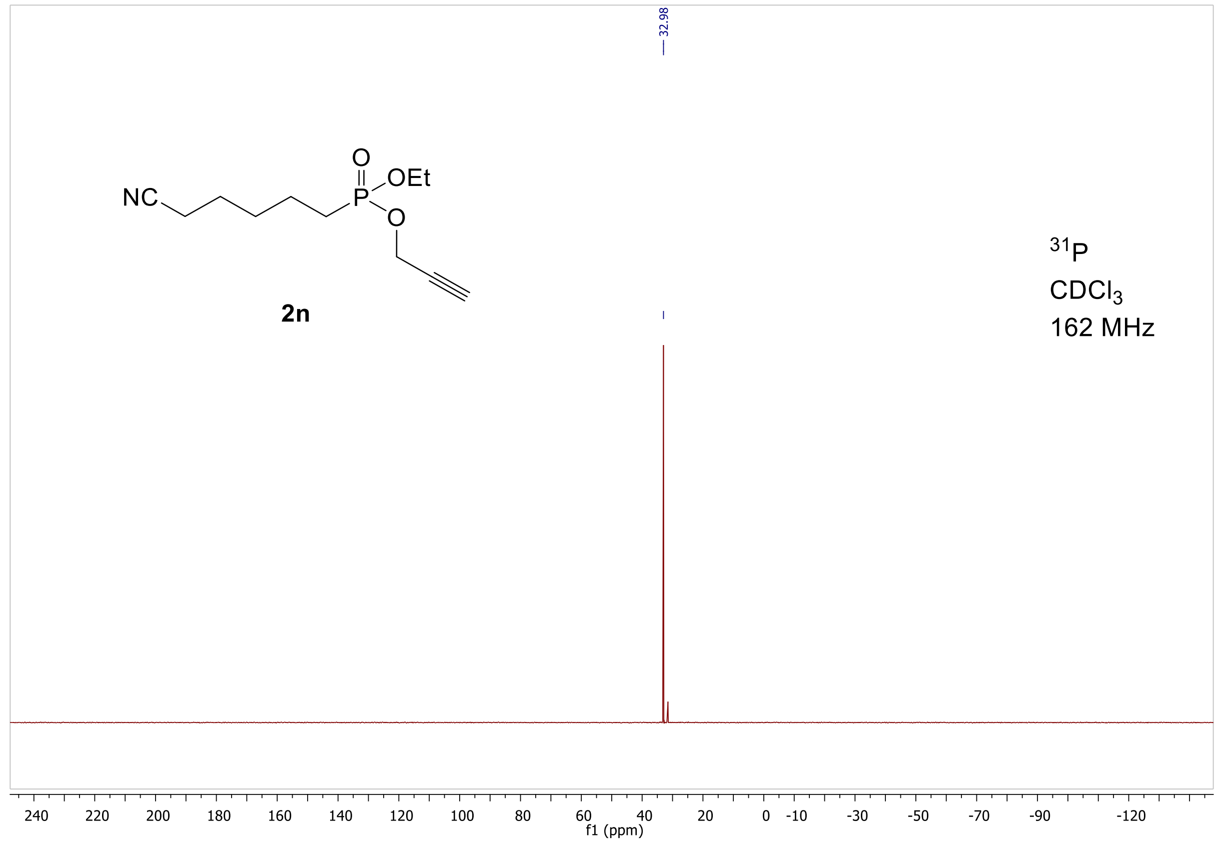

32.98
NC
O
OEt
P
O
2n
31P
CDCl3
162 MHz
240
220
200
180
160
140
120
100
80
60
40
20
0
-10
-30
-50
-70
-90
-120
f1 (ppm)

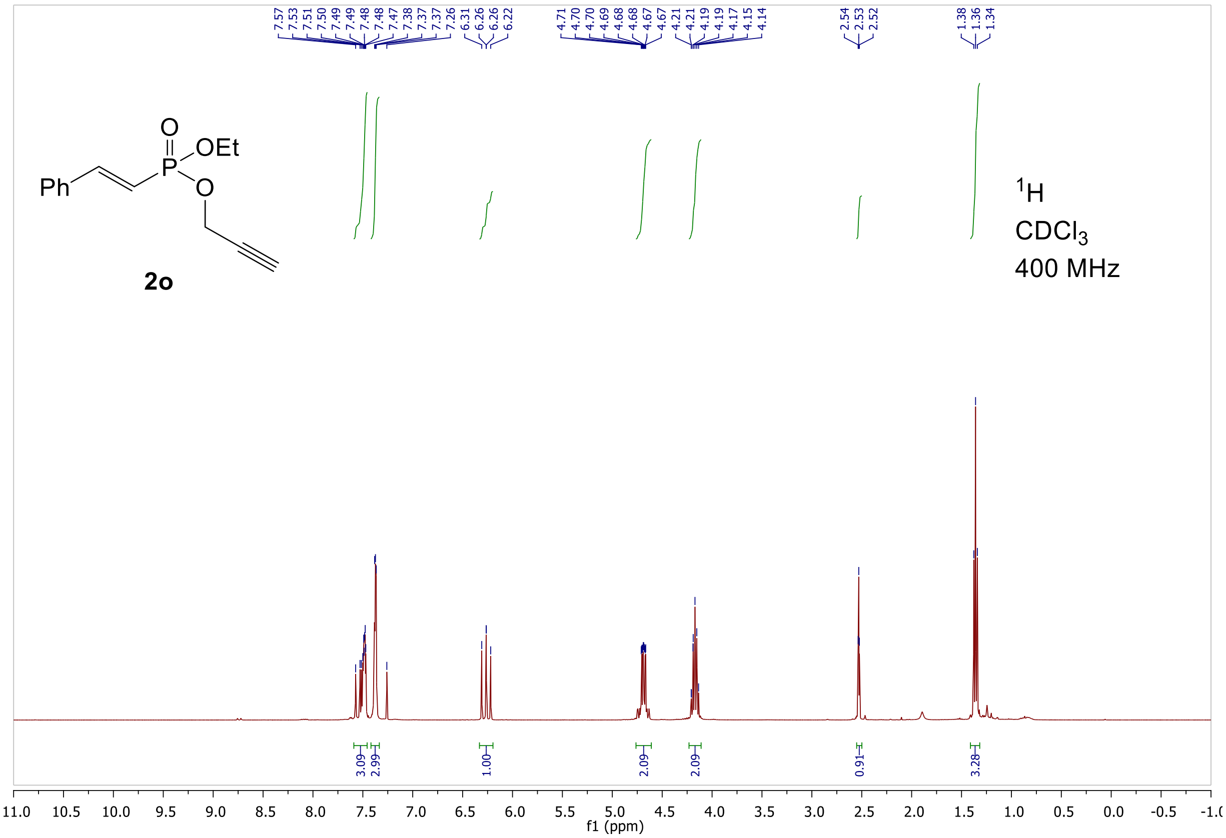
7.57 7.53 7.51 7.50 7.49 7.49 7.48 7.48 7.47 7.38 7.37 7.37 7.26 6.31 6.26 6.26 6.22
4.71 4.70 4.70 4.69 4.68 4.68 4.67 4.67 4.21 4.21 4.19 4.19 4.17 4.15 4.14
2.54 2.53 2.52
1.38 1.36 1.34
OEt
Ph
O
2o
^{1}H
$CDCl_3$
400 MHz
3.09 2.99 1.00 2.09 2.09 0.91 3.28
f1 (ppm)

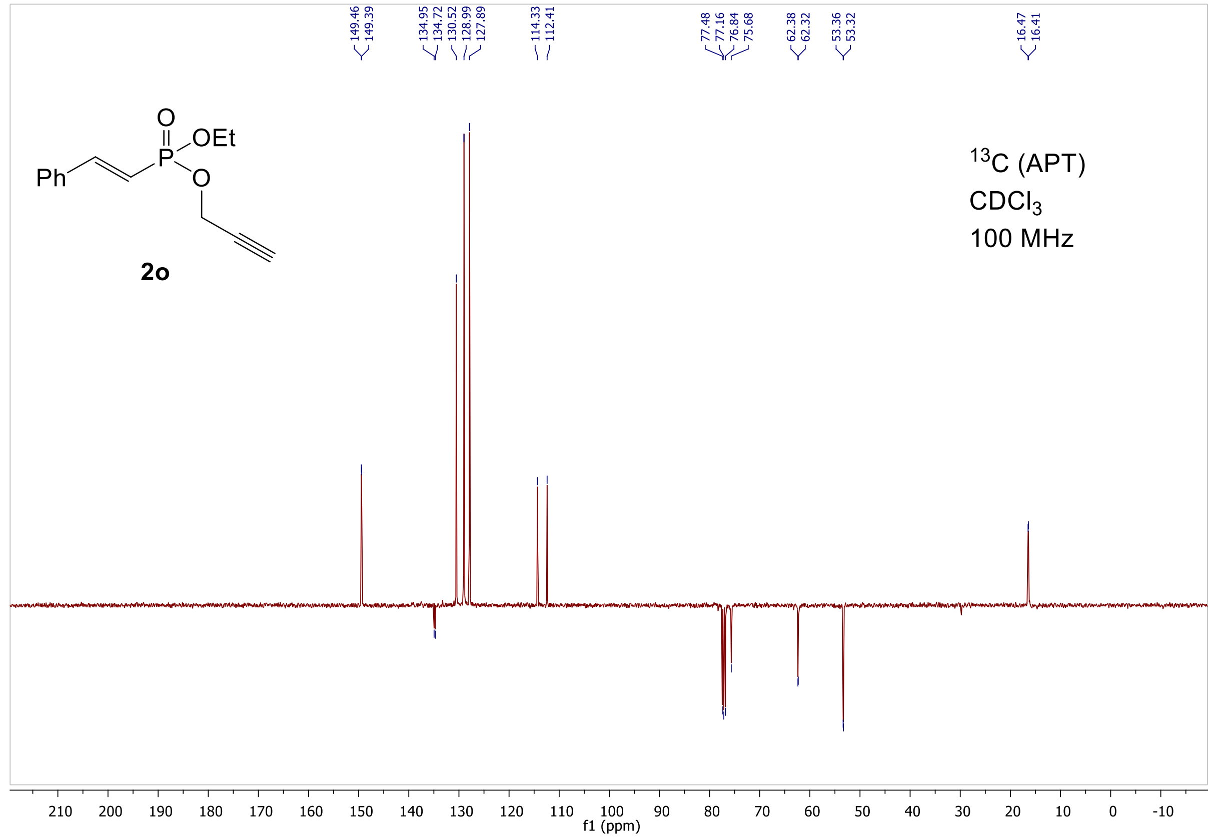
149.46 149.39
134.95 134.72 130.52 128.99 127.89
114.33 112.41
77.48 77.16 76.84 75.68
62.38 62.32
53.36 53.32
16.47 16.41
OEt
Ph
O
2o
^{13}C (APT)
$CDCl_3$
100 MHz
f1 (ppm)

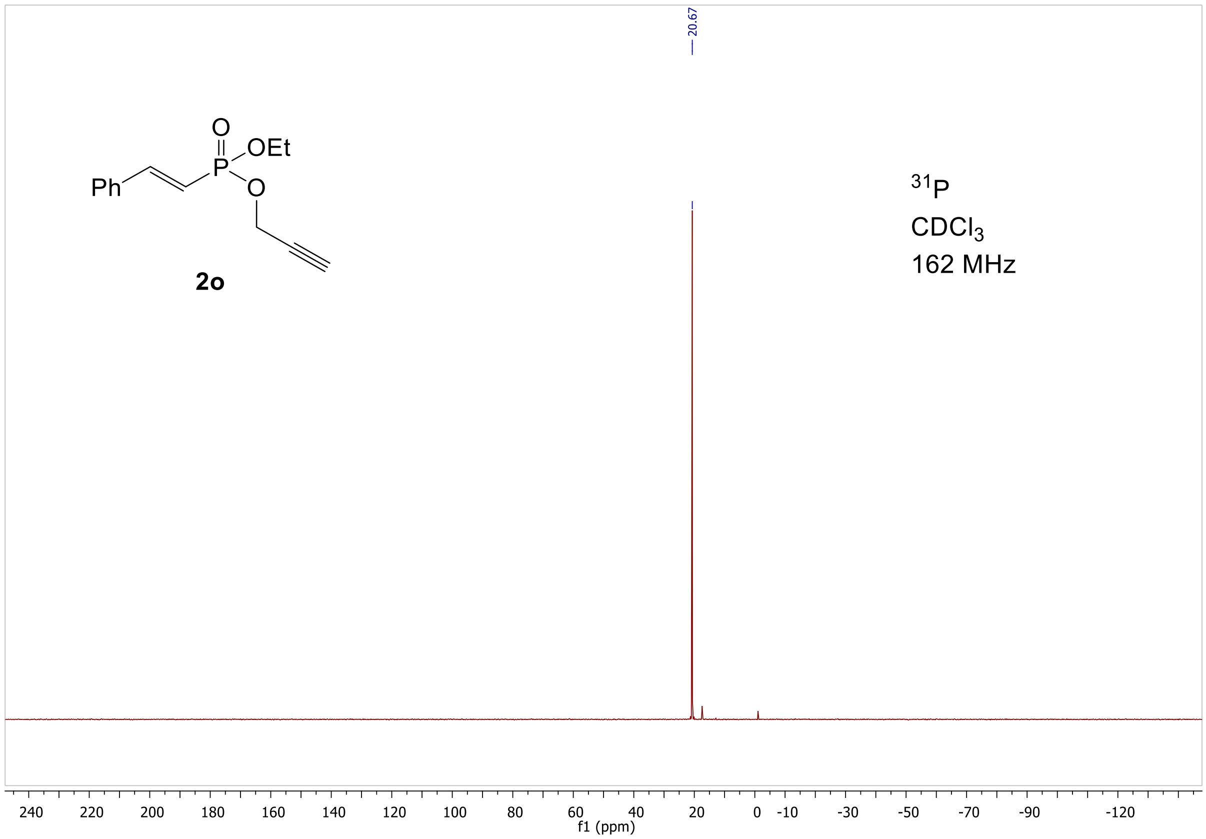
20.67
OEt
Ph
O
O
2o
31P
CDCl3
162 MHz
240
220
200
180
160
140
120
100
80
60
40
20
0
-10
-30
-50
-70
-90
-120
f1 (ppm)

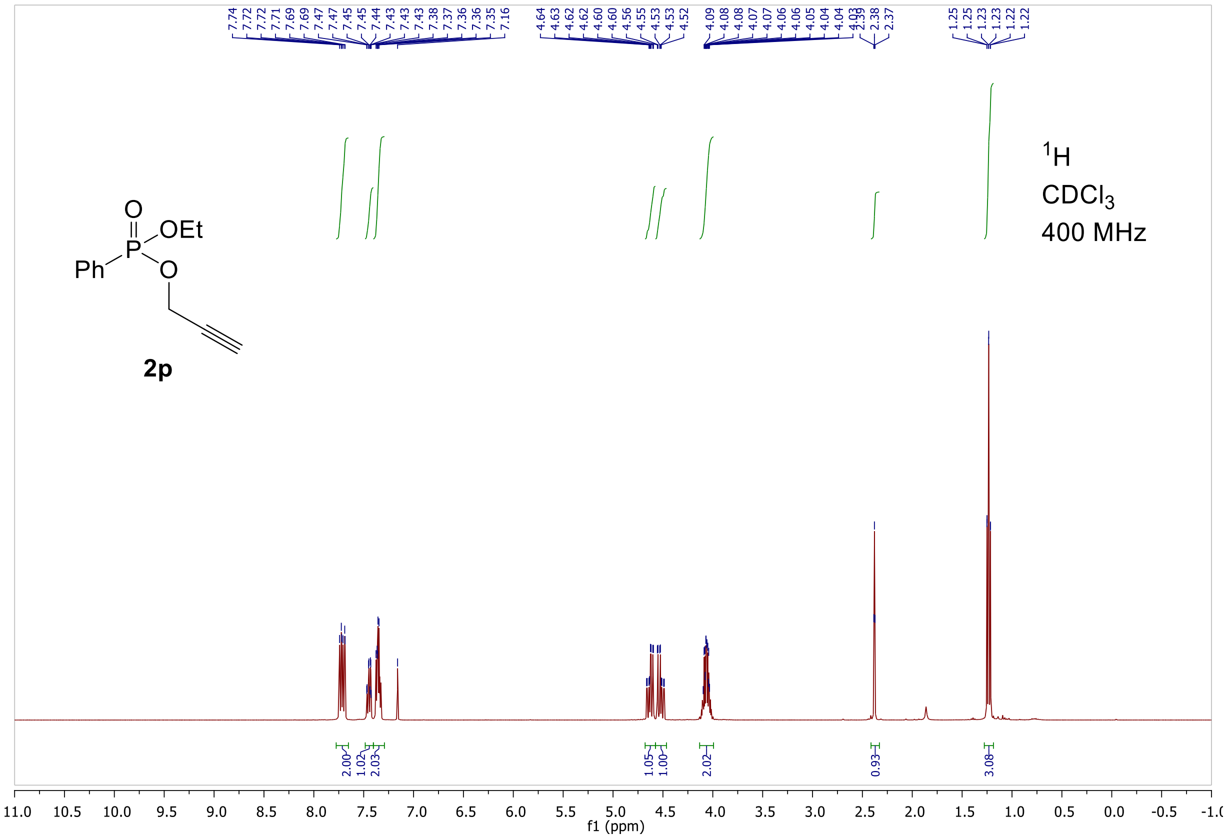

2p
1H
CDCl3
400 MHz
f1 (ppm)

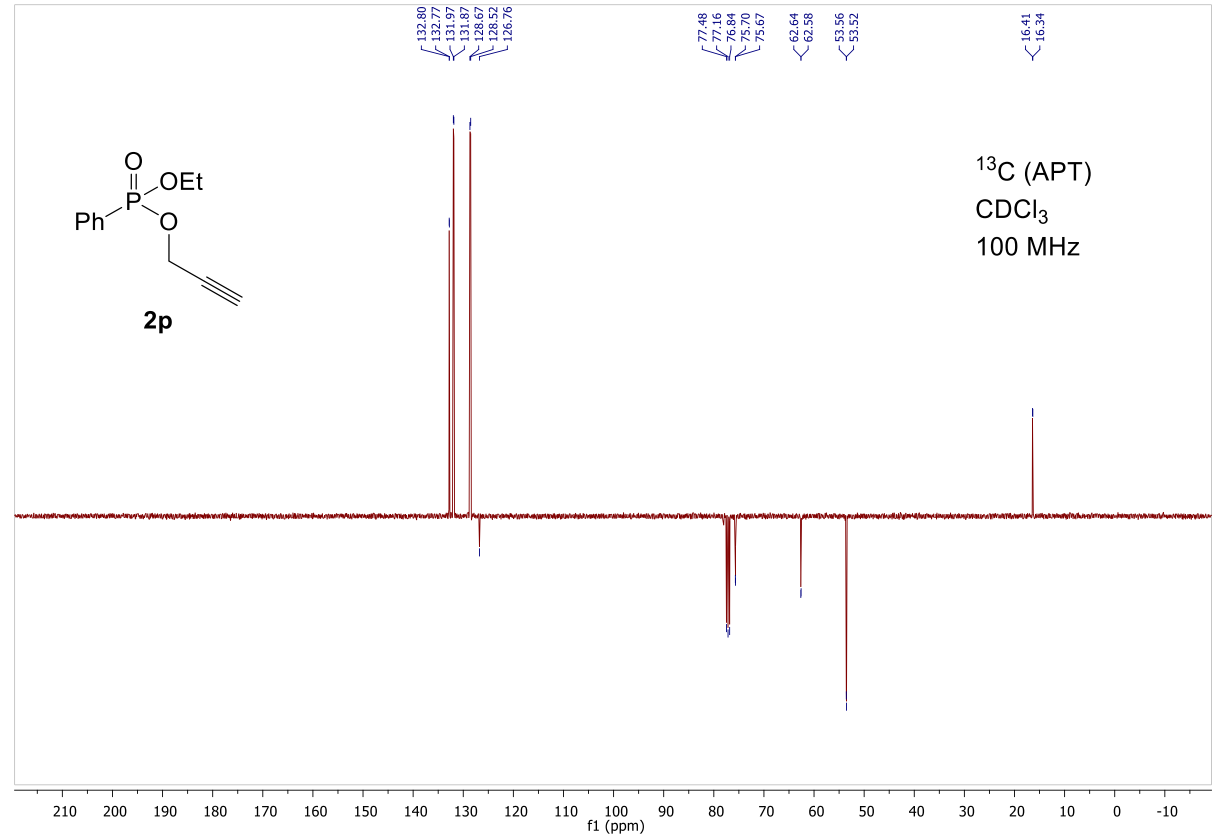

2p
13C (APT)
CDCl3
100 MHz
f1 (ppm)

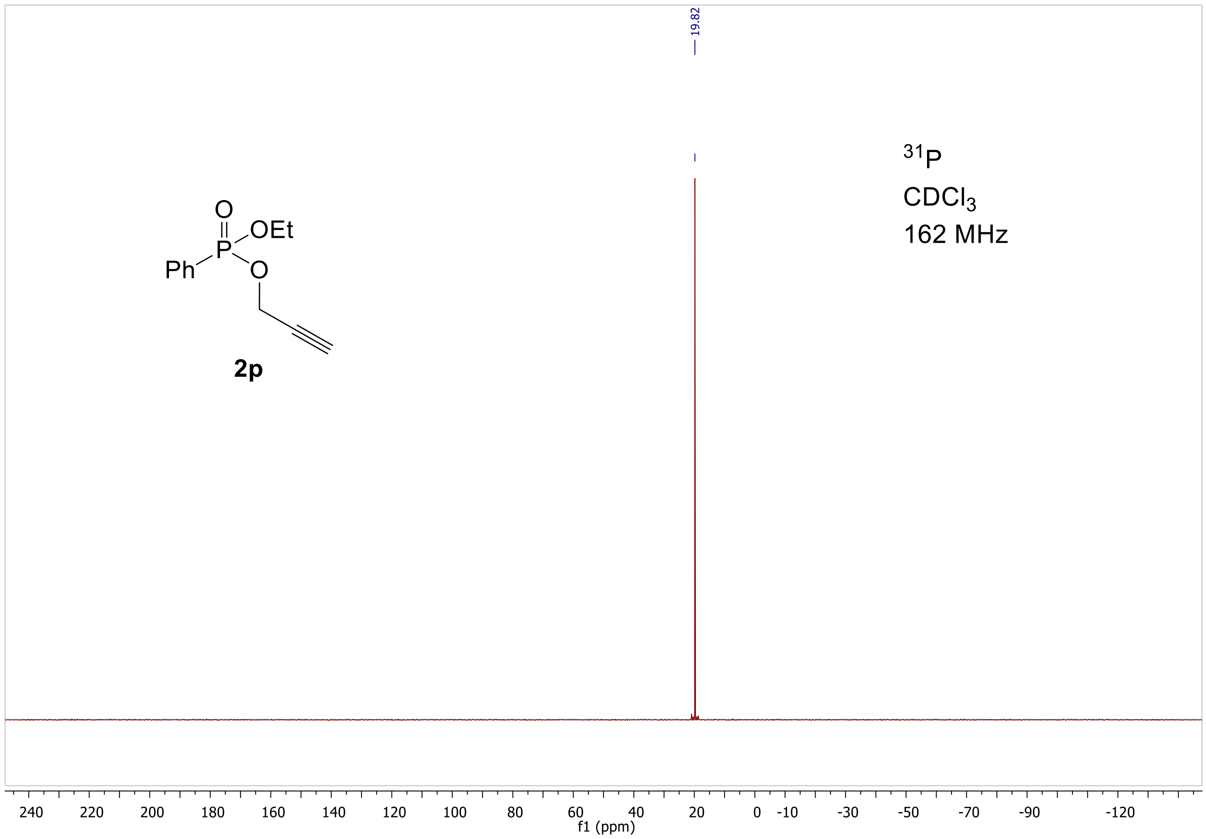

19.82
2p
OEt
Ph
^{31}P
$CDCl_3$
162 MHz
240 220 200 180 160 140 120 100 80 60 40 20 0 -10 -30 -50 -70 -90 -120
f1 (ppm)

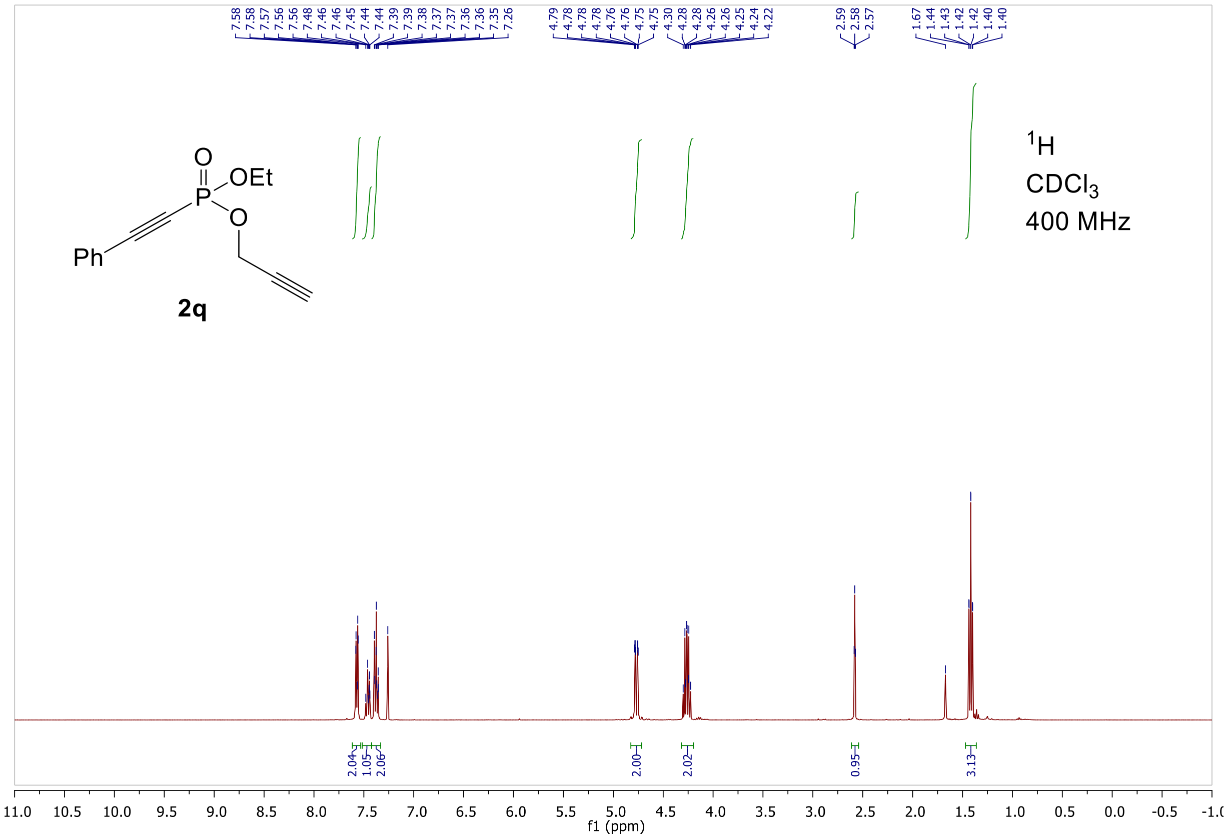

2q
¹H
CDCl₃
400 MHz
f1 (ppm)

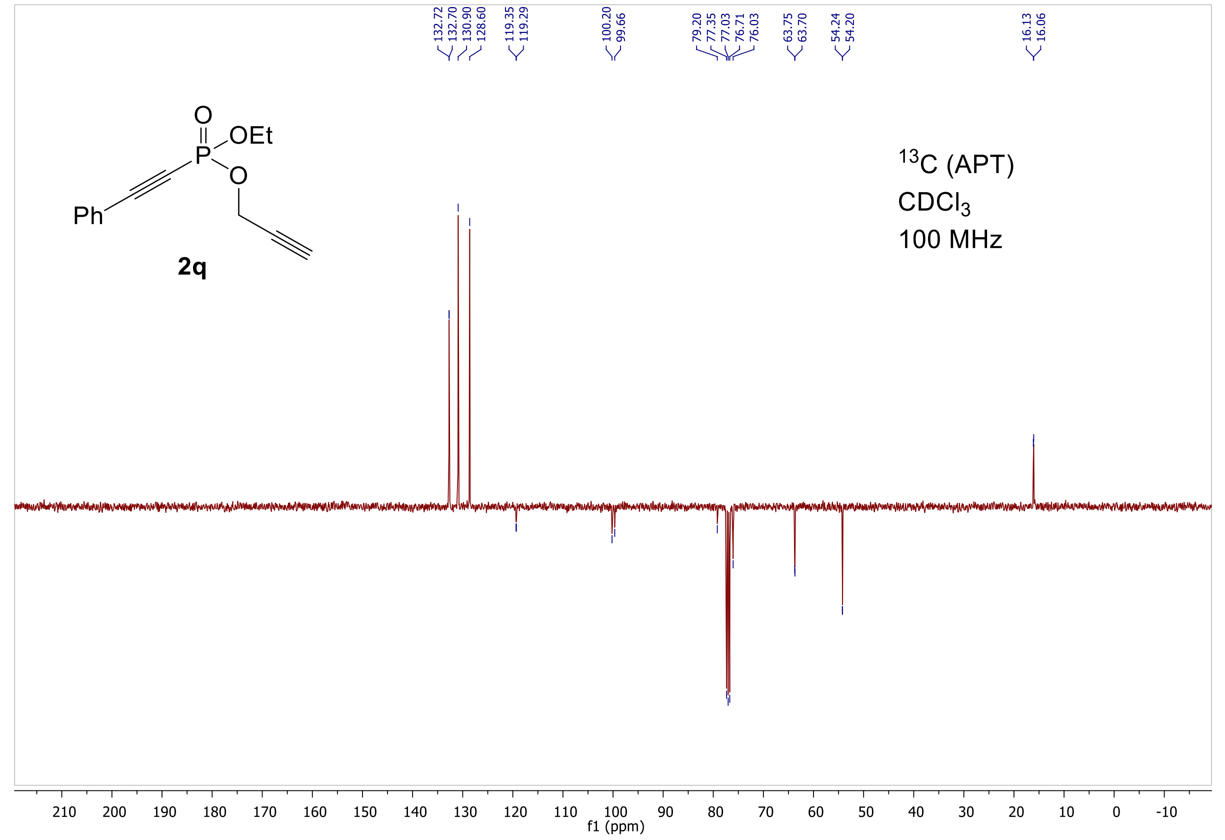

2q
¹³C (APT)
CDCl₃
100 MHz
f1 (ppm)

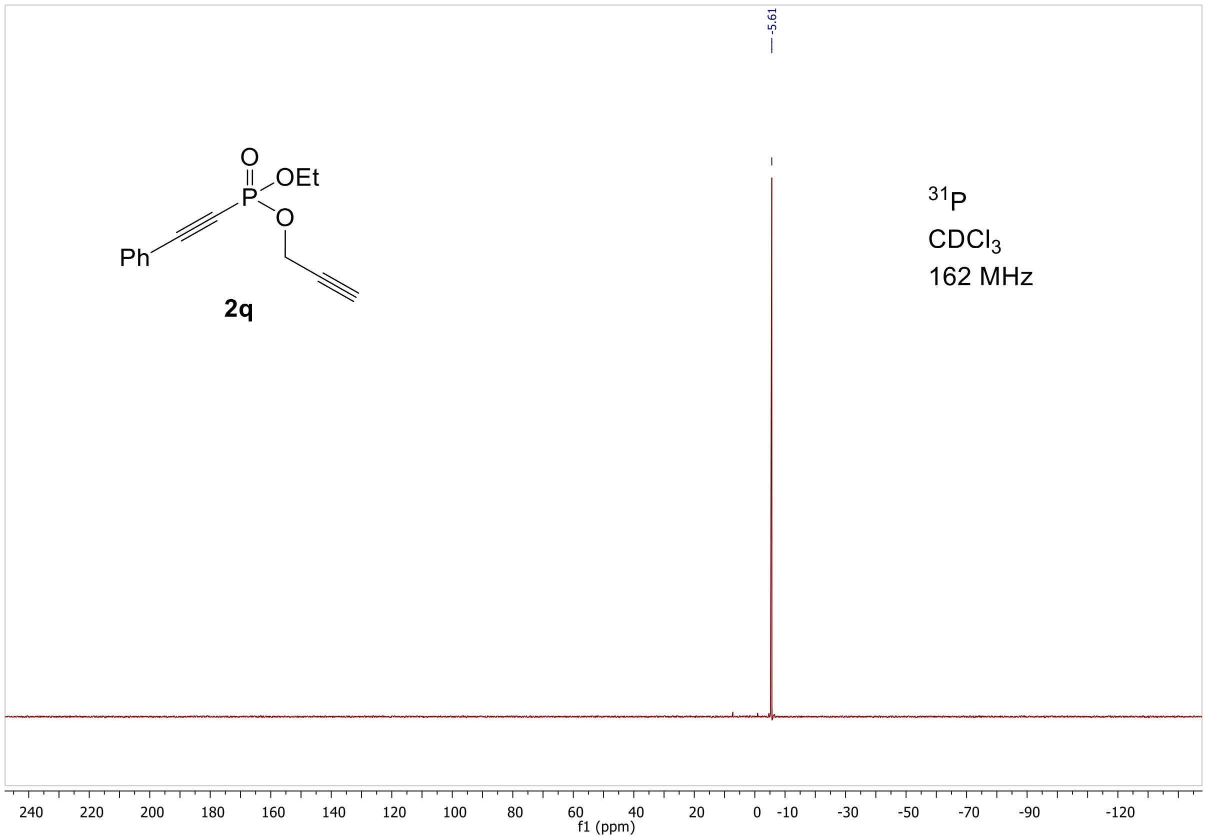
-5.61
O
OEt
P
O
Ph
2q
31P
CDCl3
162 MHz
240 220 200 180 160 140 120 100 80 60 40 20 0 -10 -30 -50 -70 -90 -120
f1 (ppm)

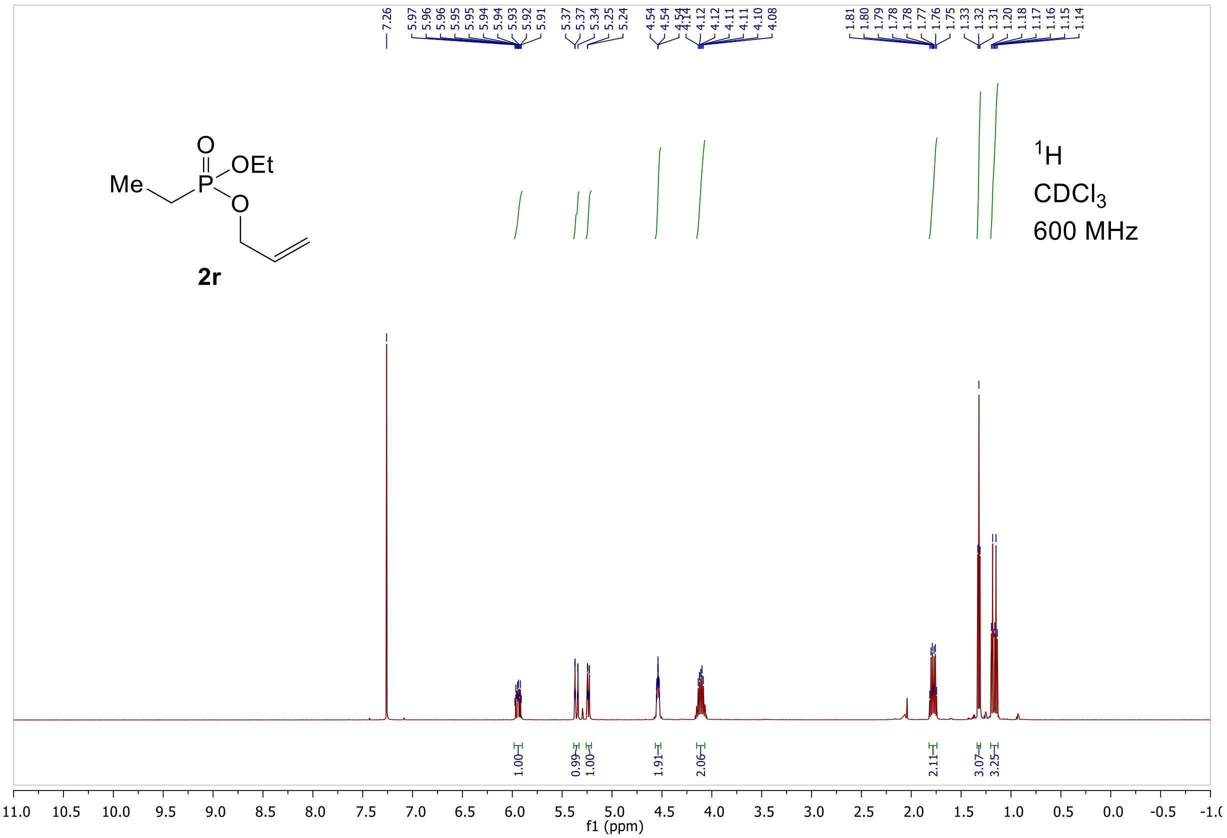

Me
P
O
OEt
O
2r
^{1}H
$CDCl_3$
600 MHz
f1 (ppm)

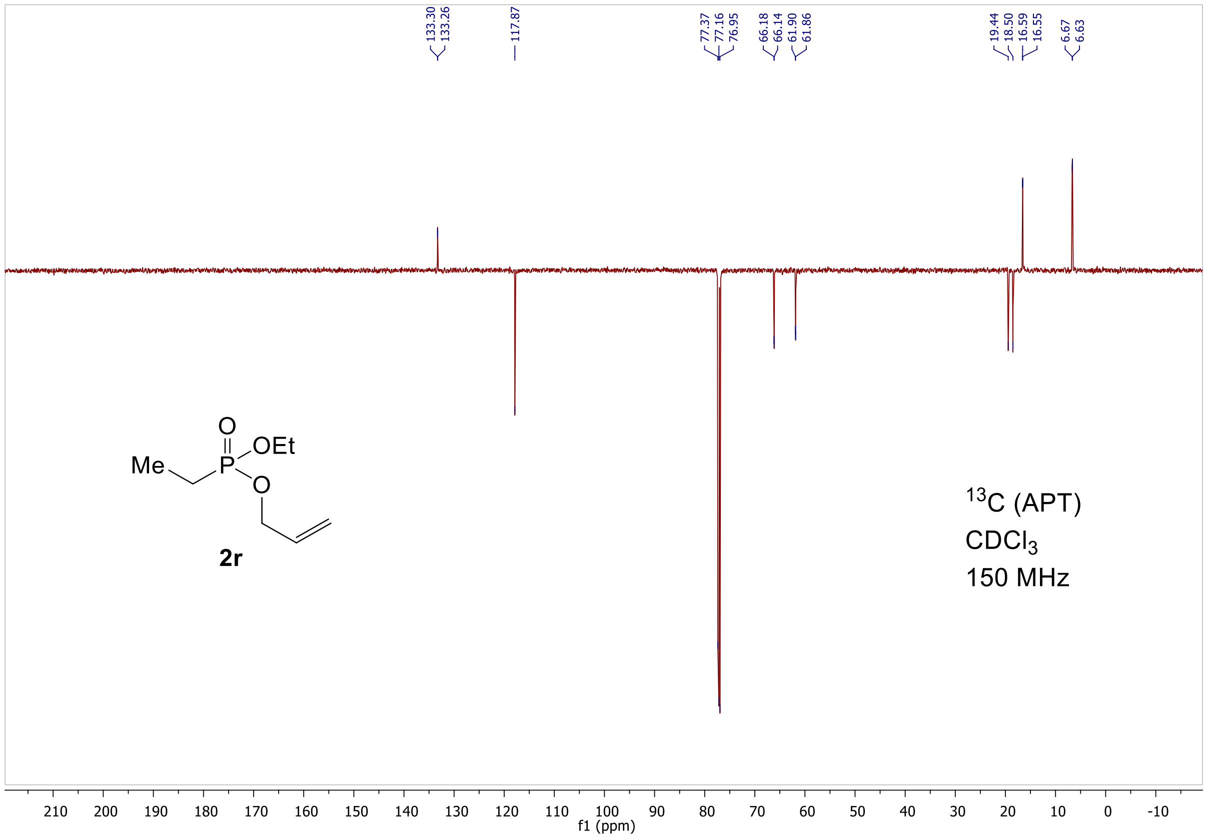

Me
P
O
OEt
O
2r
^{13}C (APT)
$CDCl_3$
150 MHz
f1 (ppm)

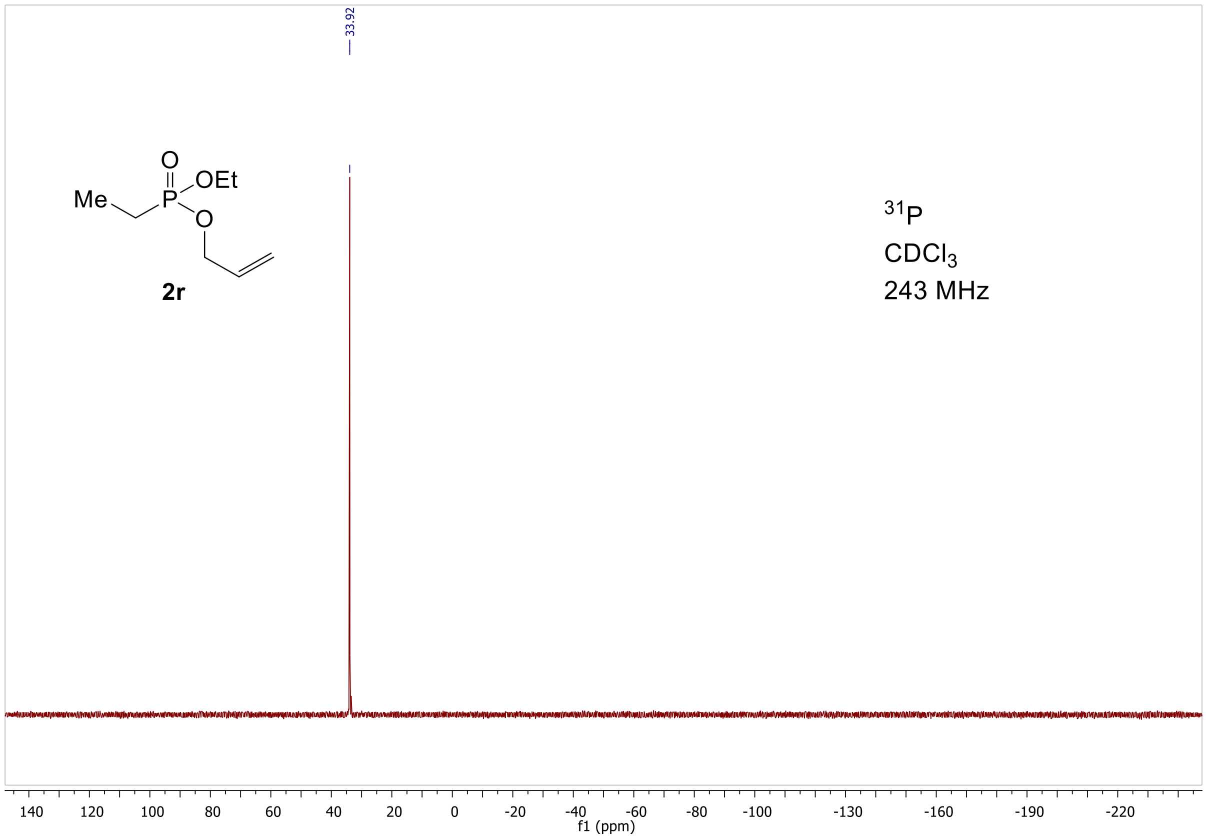
33.92
O
OEt
Me
P
O
2r
^{31}P
$CDCl_3$
243 MHz
140 120 100 80 60 40 20 0 -20 -40 -60 -80 -100 -130 -160 -190 -220
f1 (ppm)

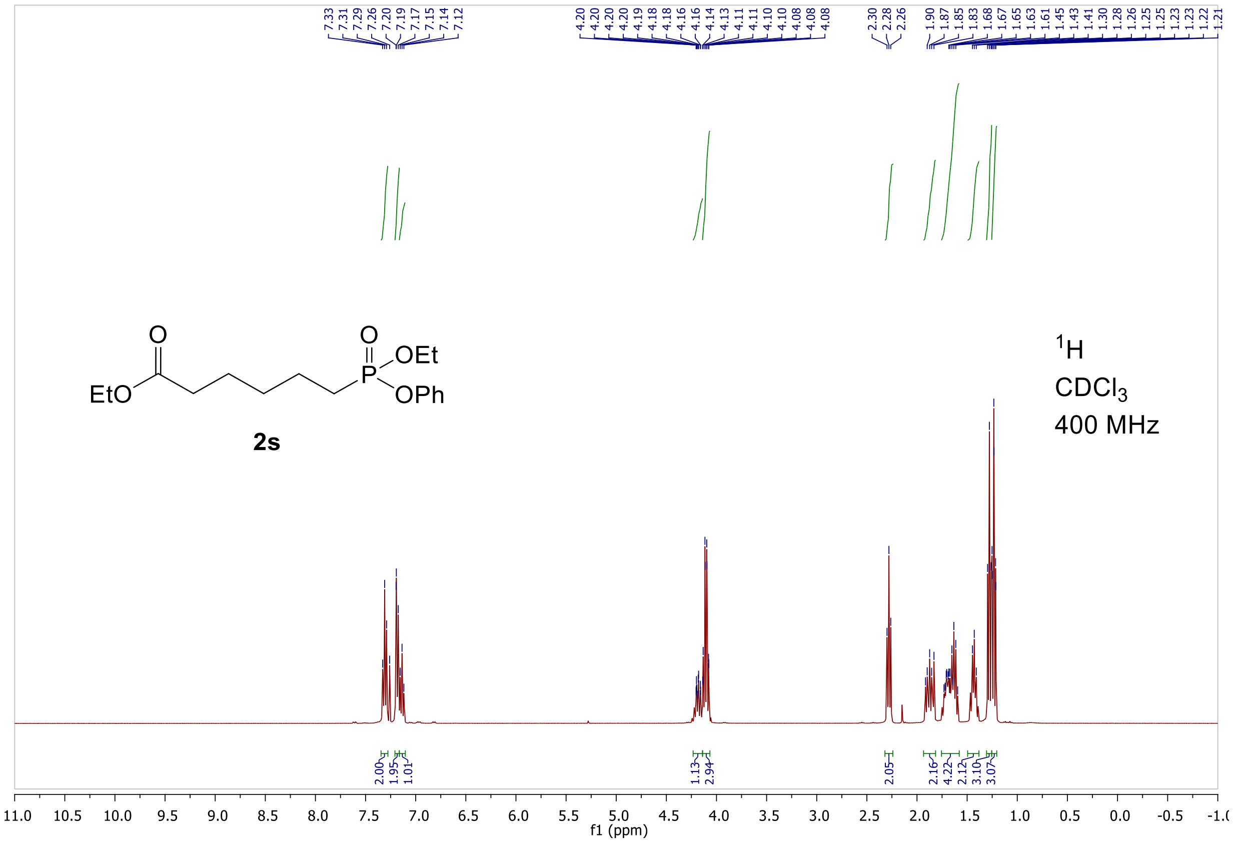

7.33 7.31 7.29 7.26 7.20 7.19 7.17 7.15 7.14 7.12
4.20 4.20 4.20 4.20 4.19 4.18 4.18 4.16 4.16 4.14 4.13 4.11 4.11 4.10 4.10 4.08 4.08 4.08
2.30 2.28 2.26
1.90 1.87 1.85 1.83 1.68 1.67 1.65 1.63 1.61 1.45 1.43 1.41 1.30 1.28 1.26 1.25 1.25 1.23 1.23 1.22 1.21
O
EtO
O
P
OEt
OPh
2s
1H
CDCl3
400 MHz
2.00 1.95 1.01
1.13 2.94
2.05
2.16 4.22 2.12 3.10 3.07
11.0 10.5 10.0 9.5 9.0 8.5 8.0 7.5 7.0 6.5 6.0 5.5 5.0 4.5 4.0 3.5 3.0 2.5 2.0 1.5 1.0 0.5 0.0 -0.5
f1 (ppm)

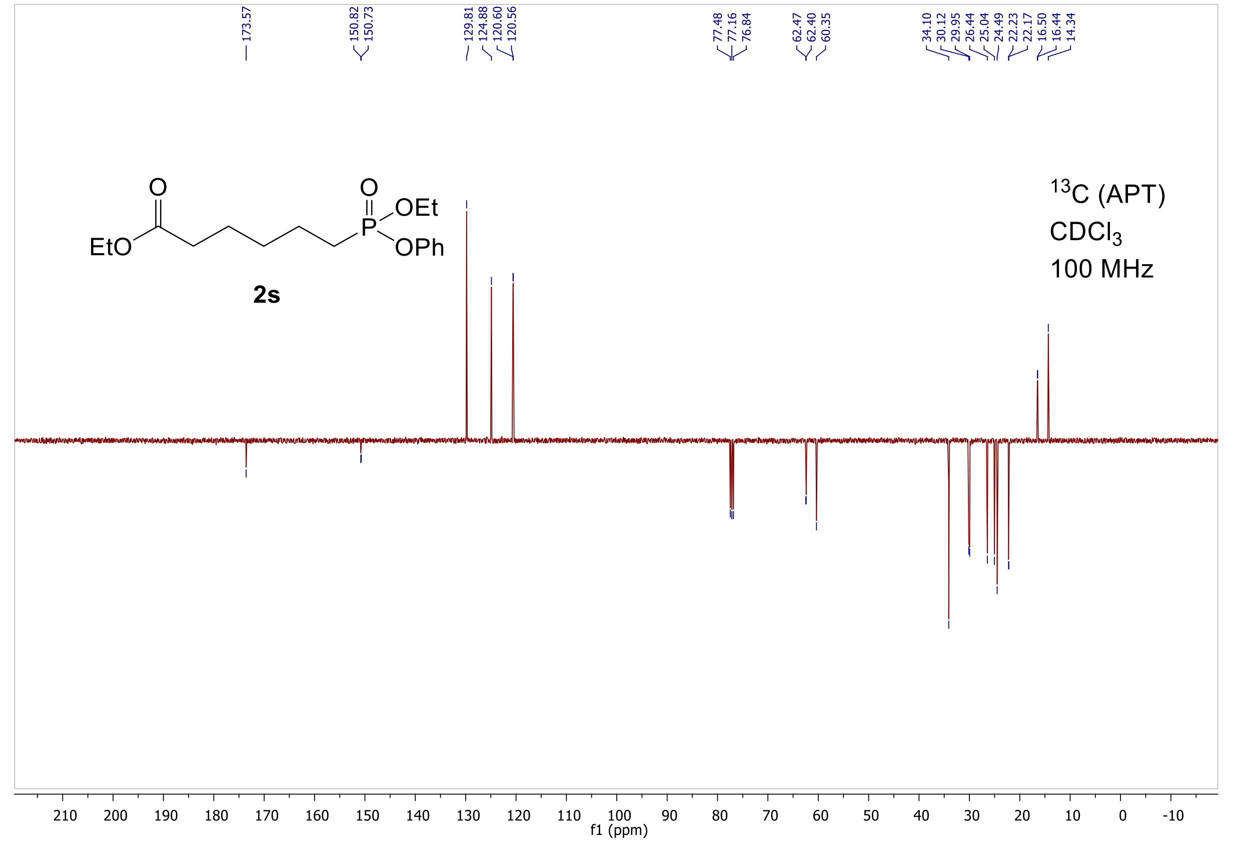

173.57
150.82 150.73
129.81 124.88 120.60 120.56
77.48 77.16 76.84
62.47 62.40 60.35
34.10 30.12 29.95 26.44 25.04 24.49 22.23 22.17 16.50 16.44 14.34
O
EtO
O
P
OEt
OPh
2s
13C (APT)
CDCl3
100 MHz
210 200 190 180 170 160 150 140 130 120 110 100 90 80 70 60 50 40 30 20 10 0 -10
f1 (ppm)

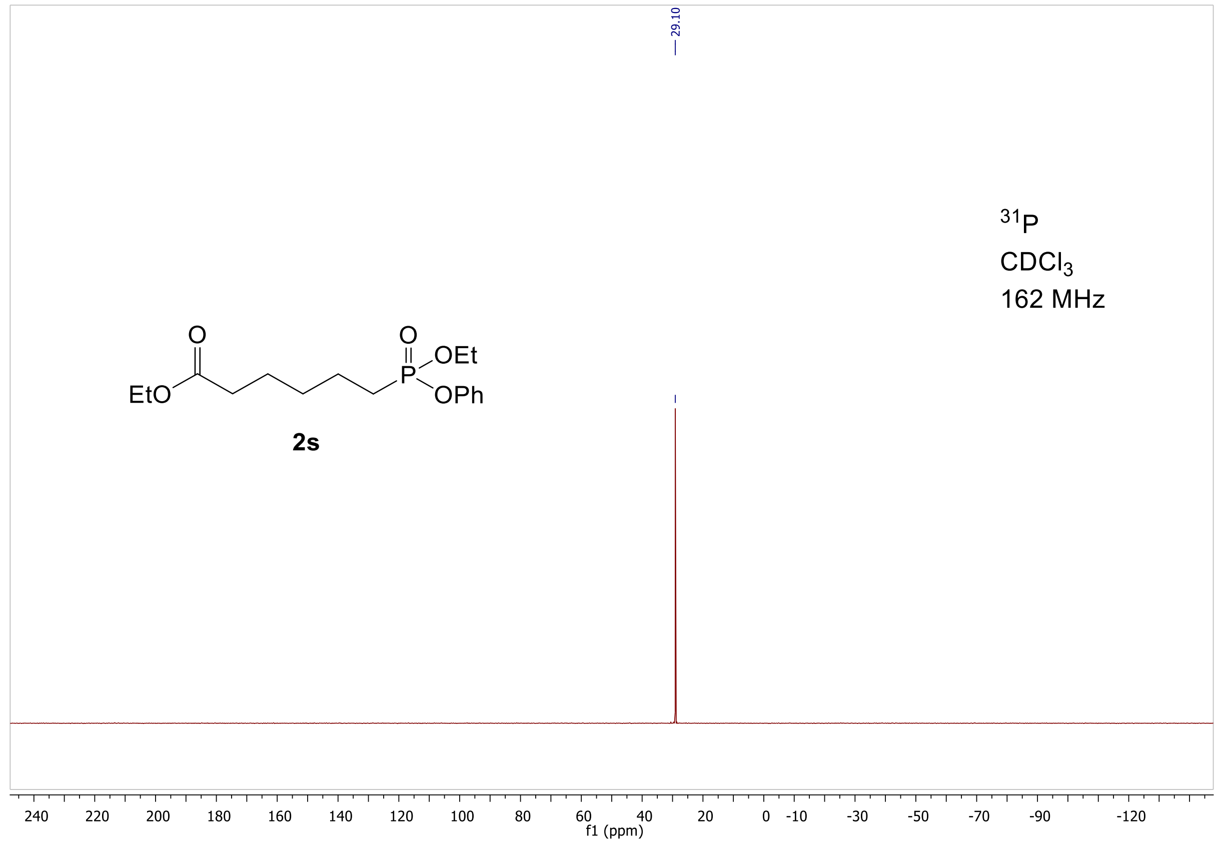
29.10
^{31}P
$CDCl_3$
162 MHz
EtO
O
OEt
OPh
P
2s
240 220 200 180 160 140 120 100 80 60 40 20 0 -10 -30 -50 -70 -90 -120
f1 (ppm)

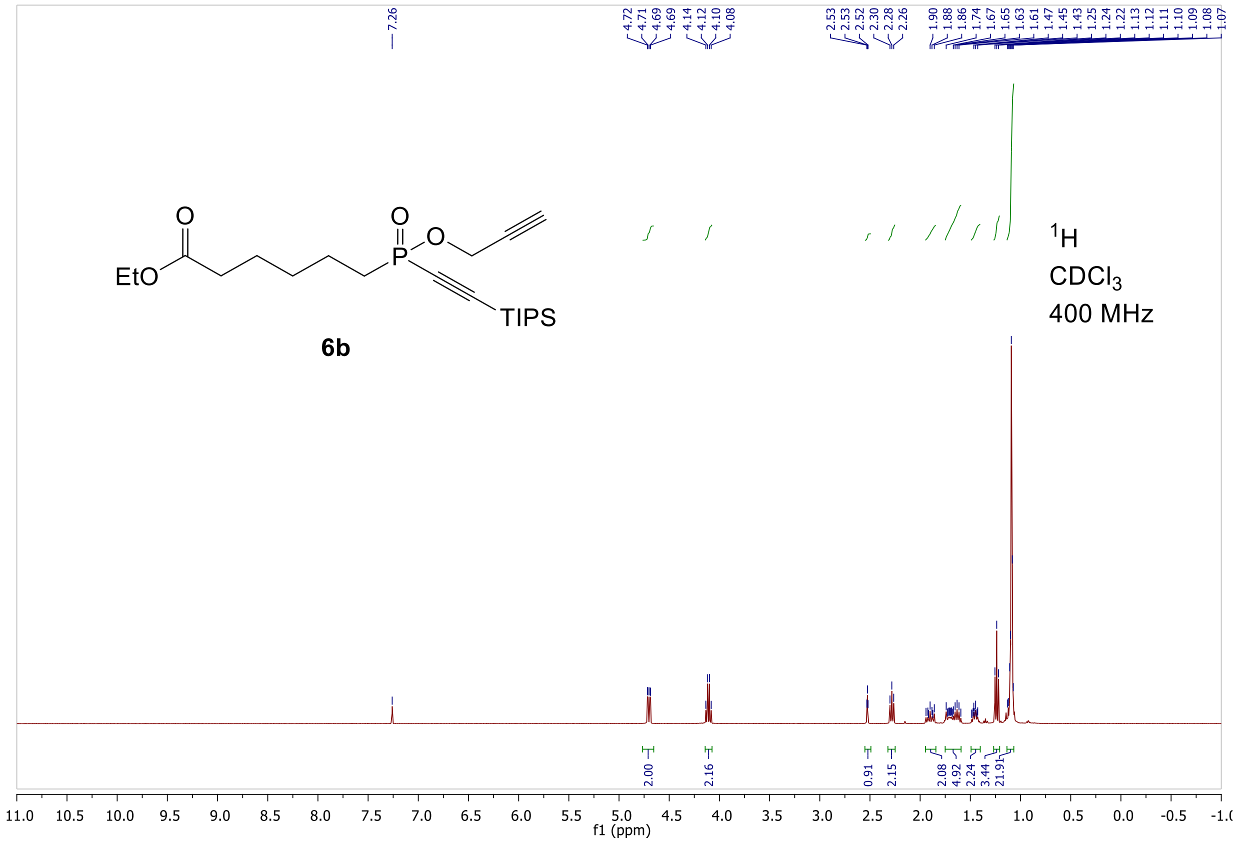
6b
1H
CDCl3
400 MHz
f1 (ppm)

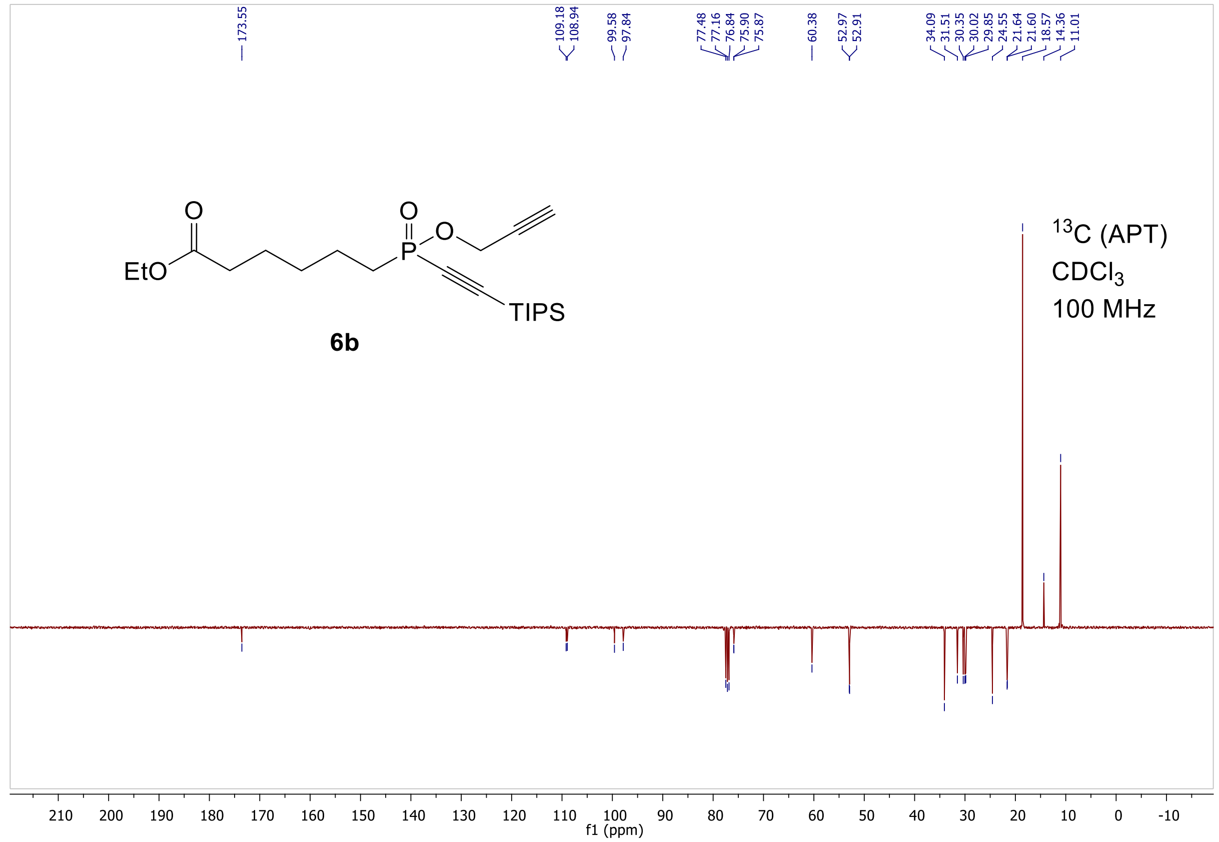
6b
13C (APT)
CDCl3
100 MHz
f1 (ppm)

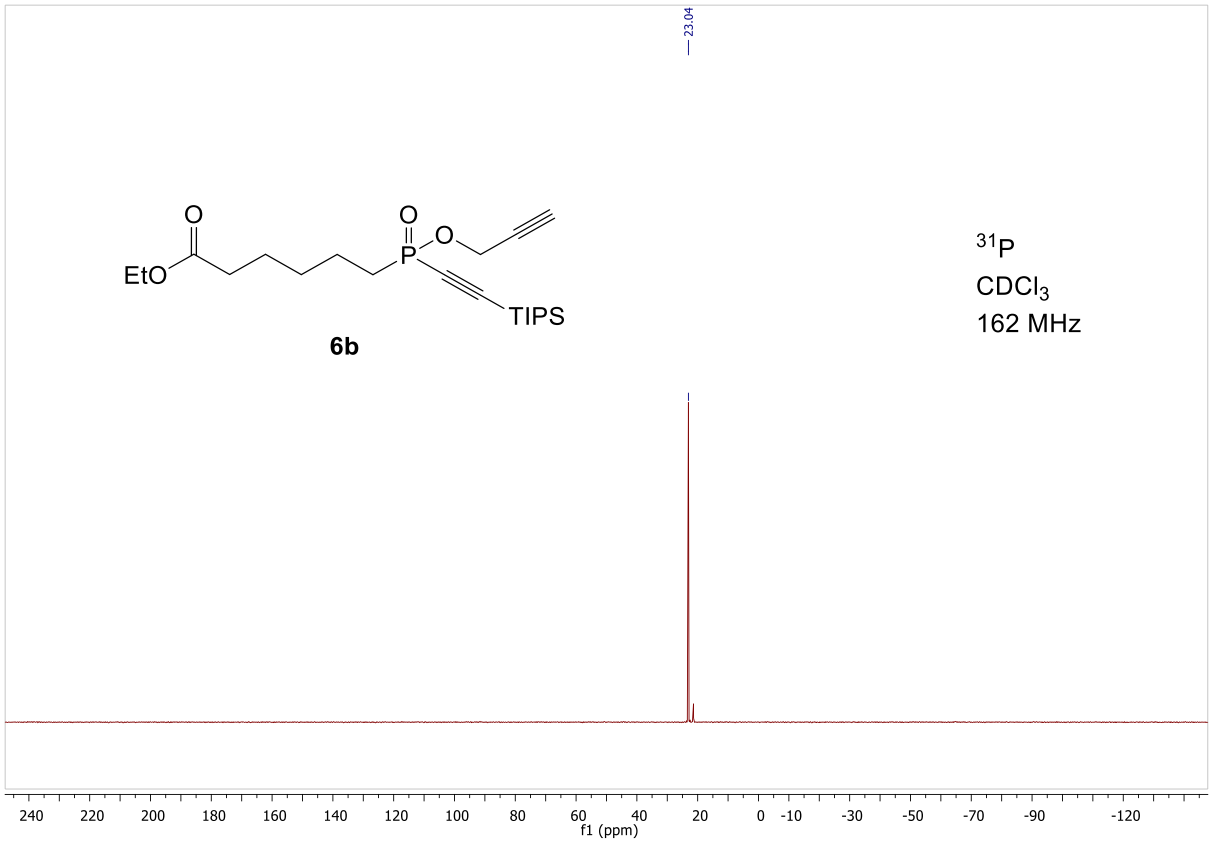
23.04
EtO
O
O
P
O
TIPS
6b
31P
CDCl3
162 MHz
240
220
200
180
160
140
120
100
80
60
40
20
0
-10
-30
-50
-70
-90
-120
f1 (ppm)

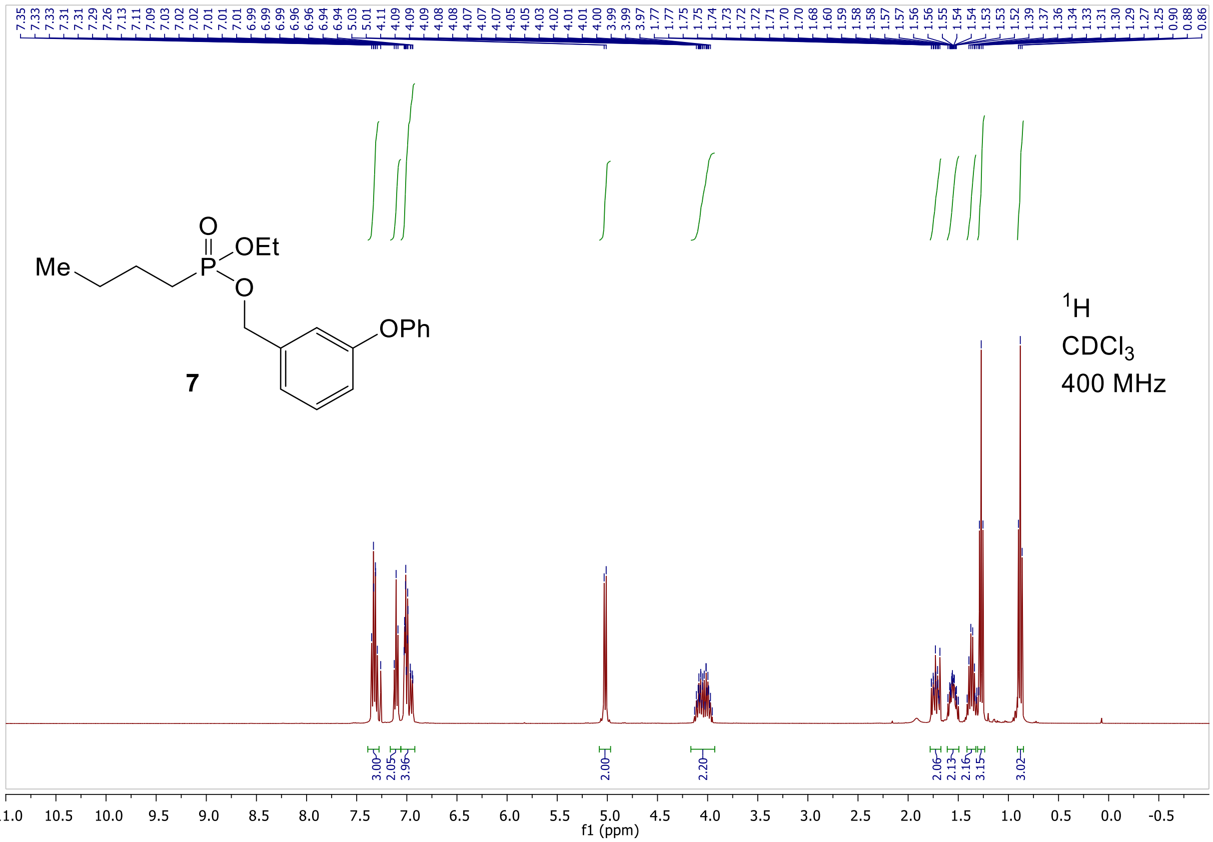
7.35 7.33 7.33 7.31 7.29 7.26 7.13 7.11 7.09 7.03 7.02 7.02 7.01 7.01 7.01 6.99 6.99 6.96 6.96 6.94 6.94 5.03 5.01 4.11 4.09 4.09 4.09 4.08 4.08 4.07 4.07 4.05 4.05 4.03 4.02 4.01 4.01 4.00 3.99 3.99 3.97 1.77 1.77 1.75 1.74 1.73 1.72 1.72 1.71 1.70 1.68 1.60 1.59 1.58 1.58 1.57 1.56 1.55 1.54 1.54 1.53 1.53 1.53 1.52 1.39 1.37 1.36 1.34 1.33 1.31 1.30 1.29 1.27 1.25 0.90 0.88 0.86
O
OEt
Me
P
O
OPh
7
1H
CDCl3
400 MHz
3.00 2.05 3.96 2.00 2.20 2.06 2.13 2.16 3.15 3.02
11.0 10.5 10.0 9.5 9.0 8.5 8.0 7.5 7.0 6.5 6.0 5.5 5.0 4.5 4.0 3.5 3.0 2.5 2.0 1.5 1.0 0.5 0.0 -0.5
f1 (ppm)

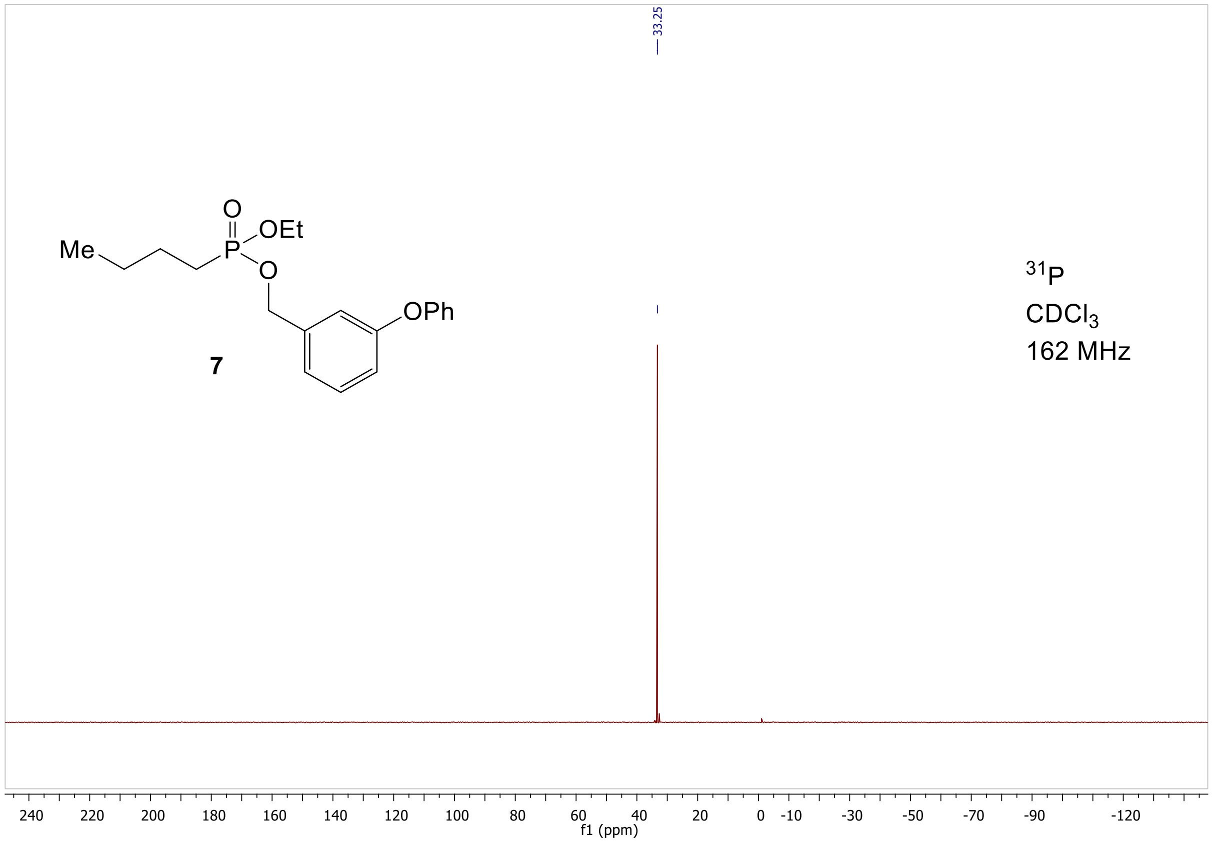
33.25
O
OEt
Me
P
O
OPh
7
31P
CDCl3
162 MHz
240 220 200 180 160 140 120 100 80 60 40 20 0 -10 -30 -50 -70 -90 -120
f1 (ppm)

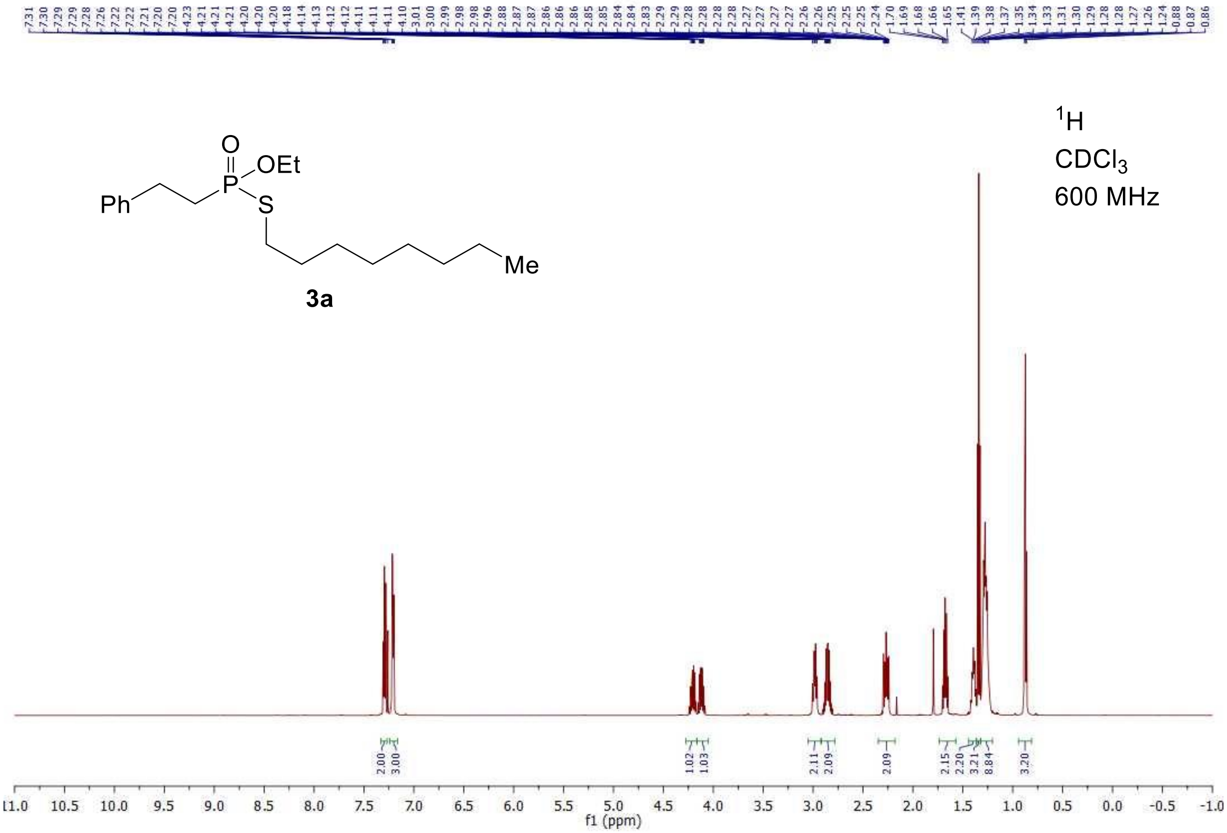
O
OEt
Ph
P
S
Me
3a
1H
CDCl3
600 MHz
2.00
3.00
1.02
1.03
2.11
2.09
2.09
2.15
2.20
3.21
8.84
3.20
f1 (ppm)

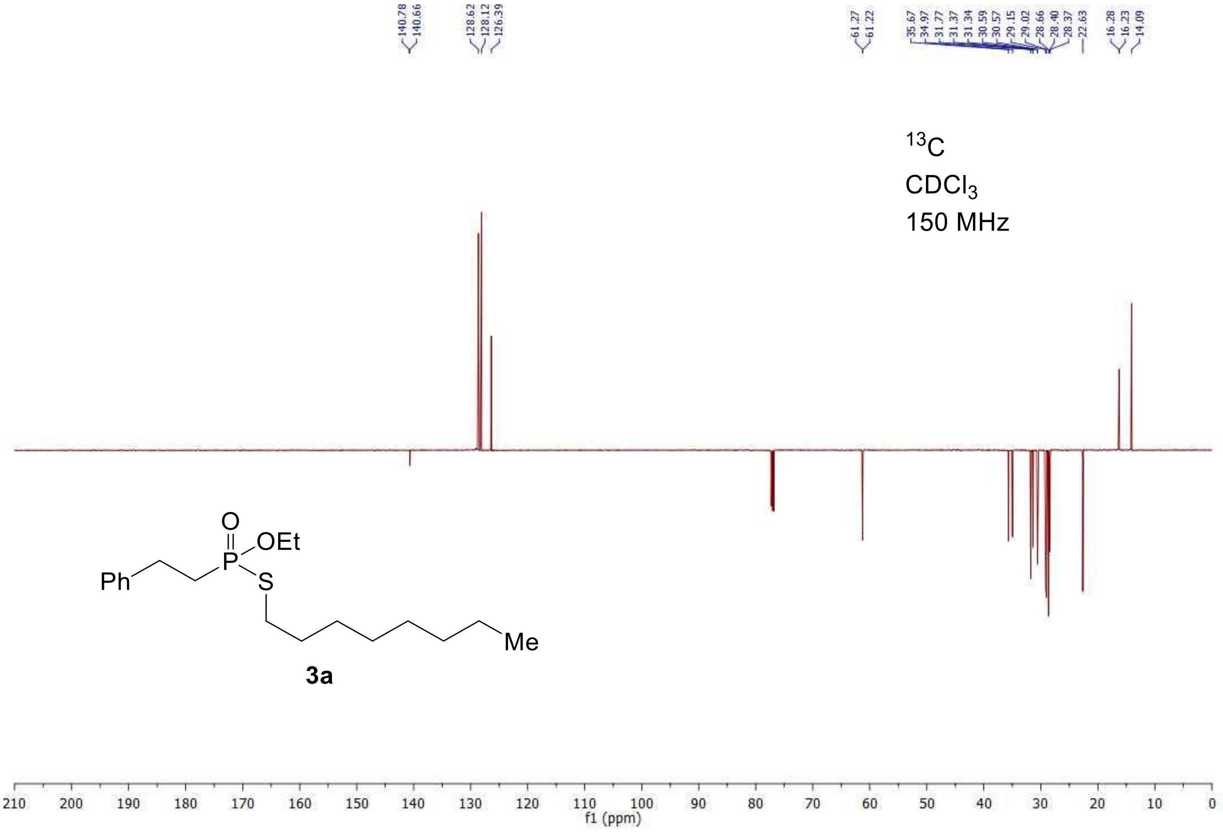
140.78
140.66
128.62
128.12
126.39
61.27
61.22
35.67
34.97
31.77
31.37
31.34
30.59
30.57
29.15
29.02
28.66
28.40
28.37
22.63
16.28
16.23
14.09
13C
CDCl3
150 MHz
O
OEt
Ph
P
S
Me
3a
f1 (ppm)

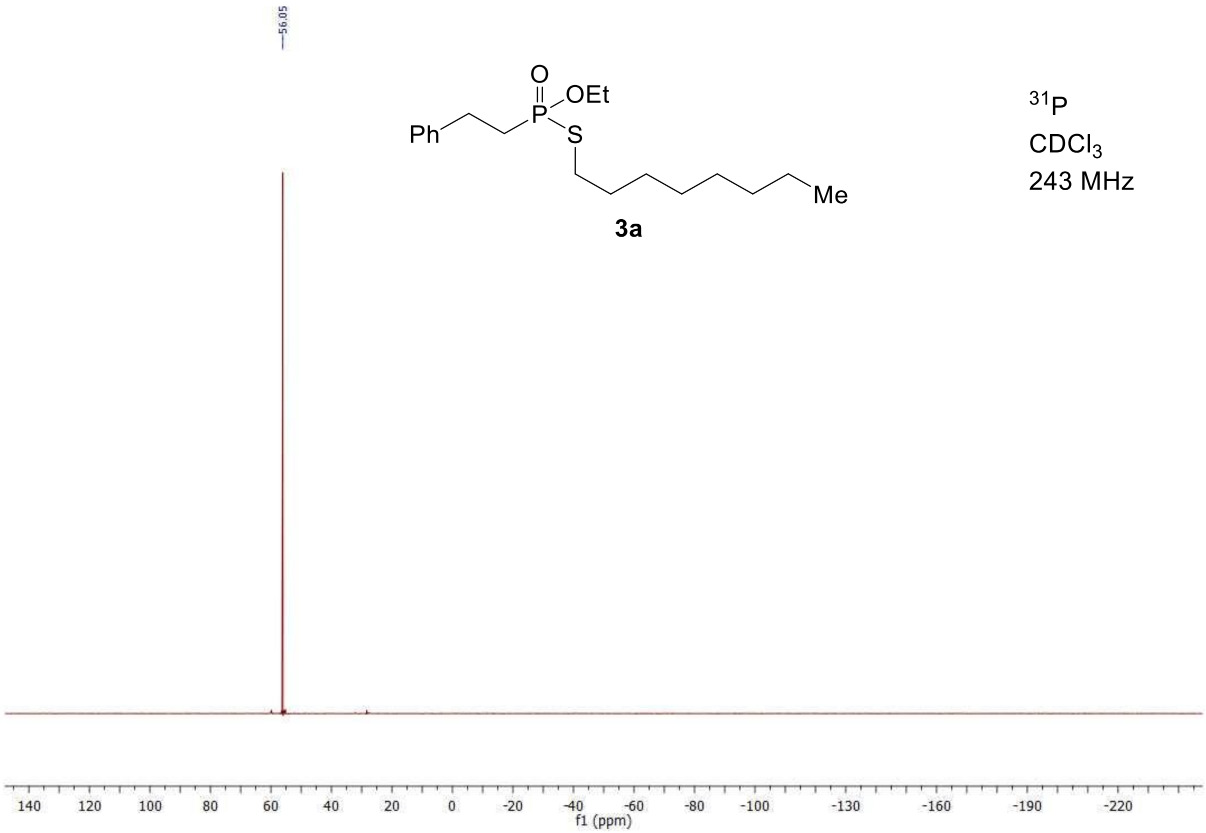

56.05
O
OEt
P
Ph
S
Me
3a
^{31}P
$CDCl_3$
243 MHz
140 120 100 80 60 40 20 0 -20 -40 -60 -80 -100 -130 -160 -190 -220
f1 (ppm)

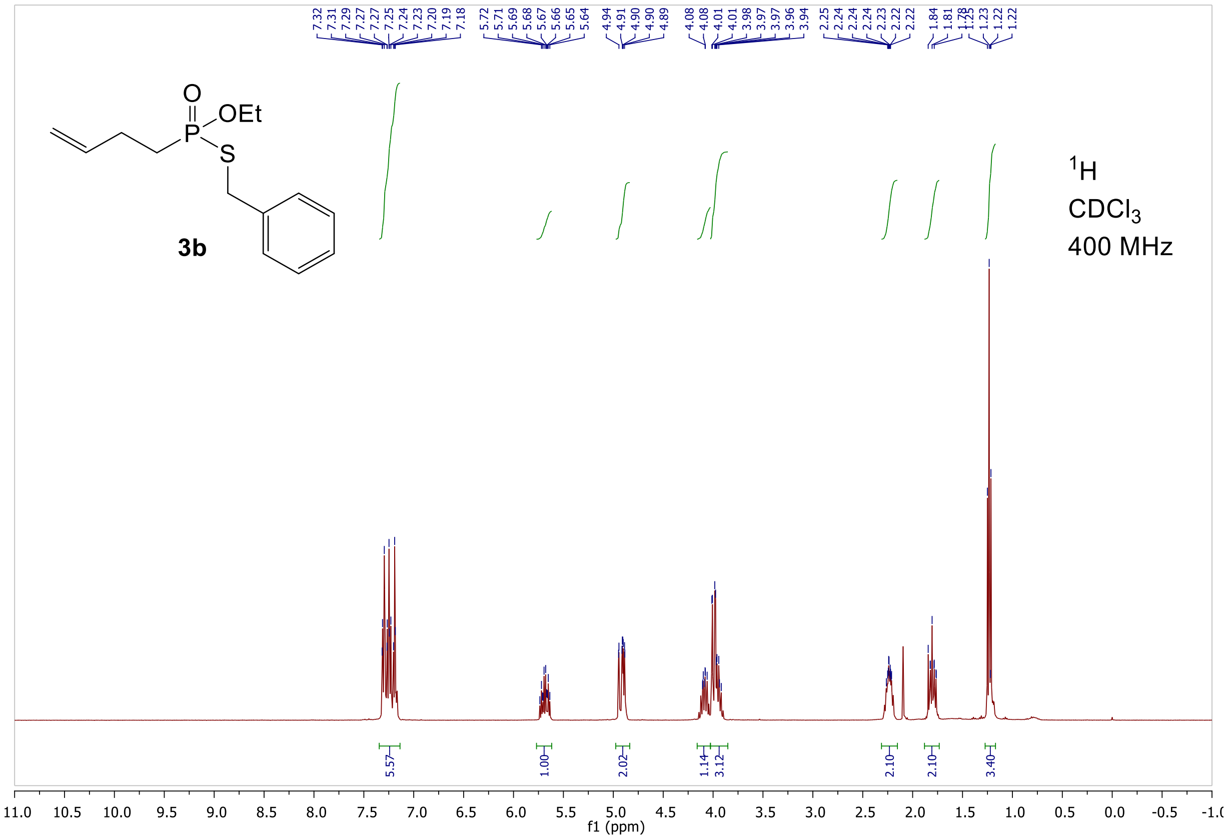

3b
^{1}H
CDCl$_3$
400 MHz
f1 (ppm)

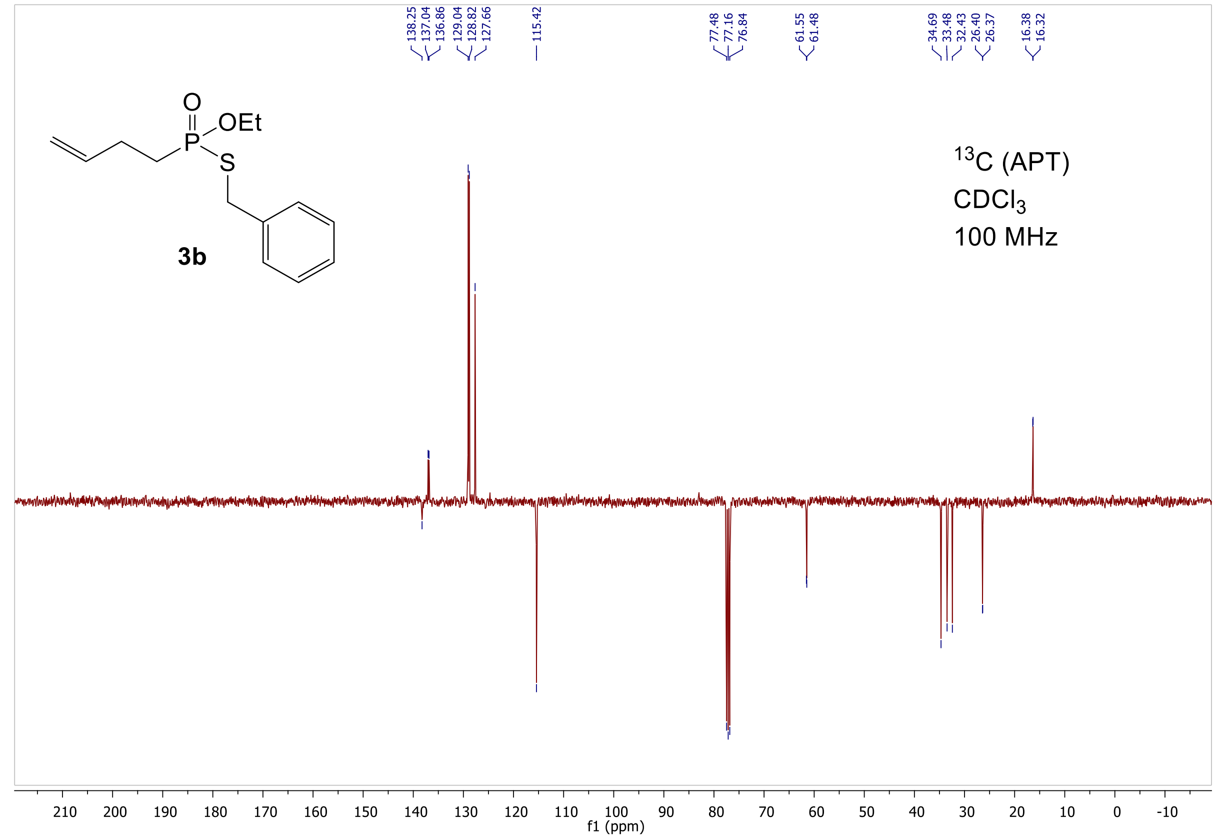

3b
^{13}C (APT)
CDCl$_3$
100 MHz
f1 (ppm)

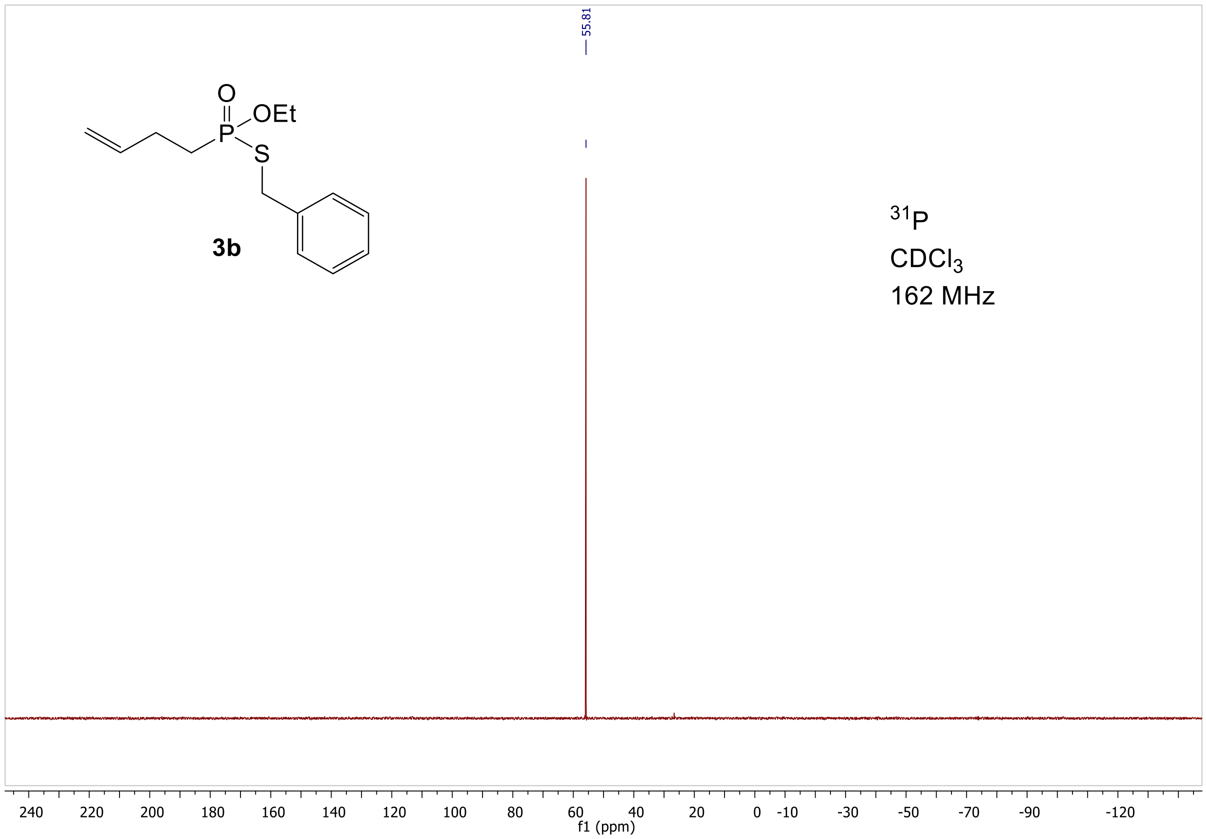

55.81
OEt
O
P
S
3b
31P
CDCl3
162 MHz
240 220 200 180 160 140 120 100 80 60 40 20 0 -10 -30 -50 -70 -90 -120
f1 (ppm)

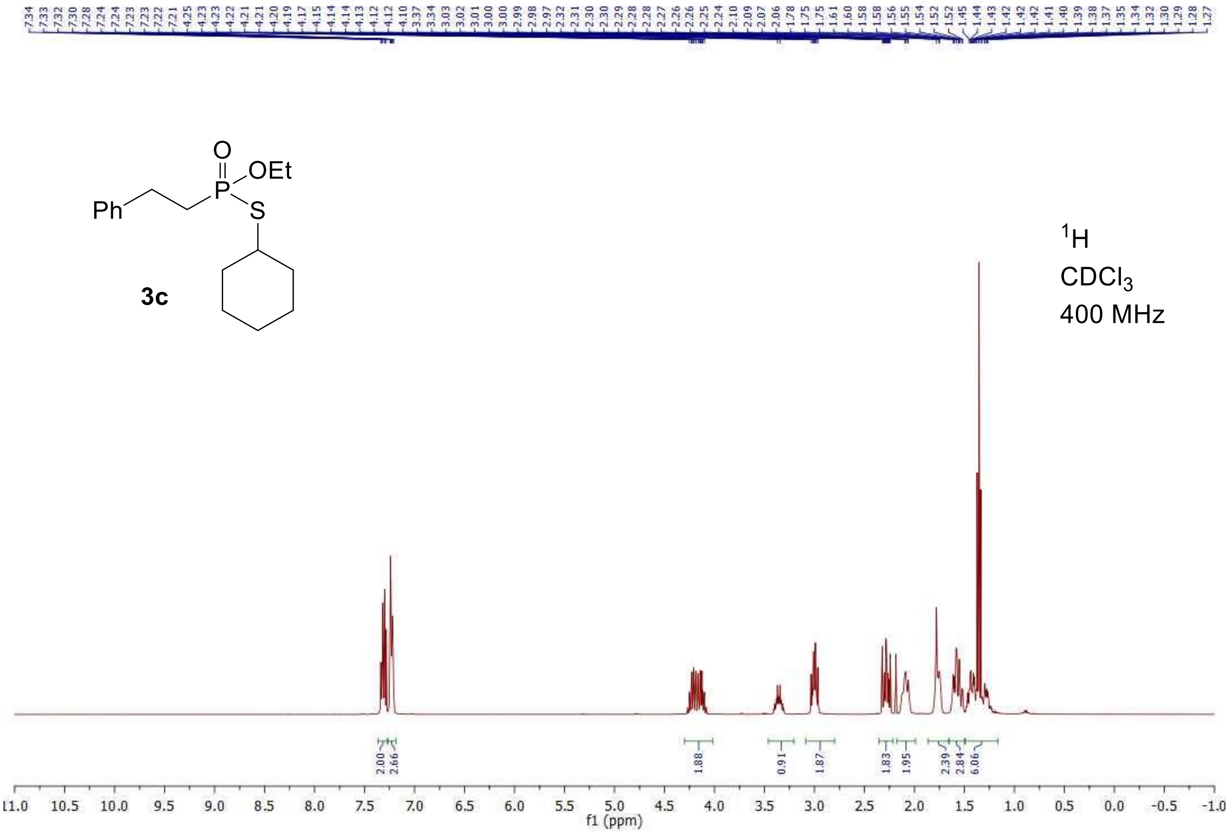
OEt
Ph
S
3c
^{1}H
$CDCl_3$
400 MHz
f1 (ppm)

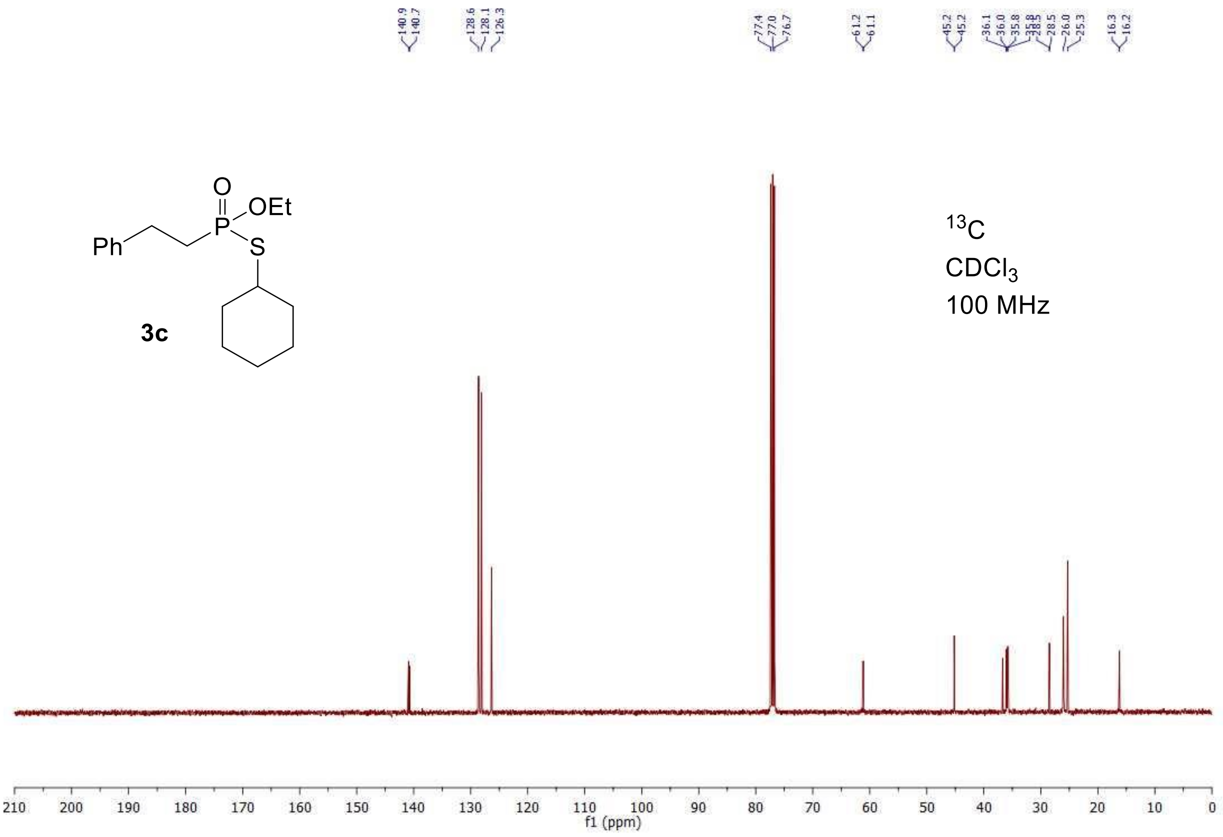
OEt
Ph
S
3c
^{13}C
$CDCl_3$
100 MHz
f1 (ppm)

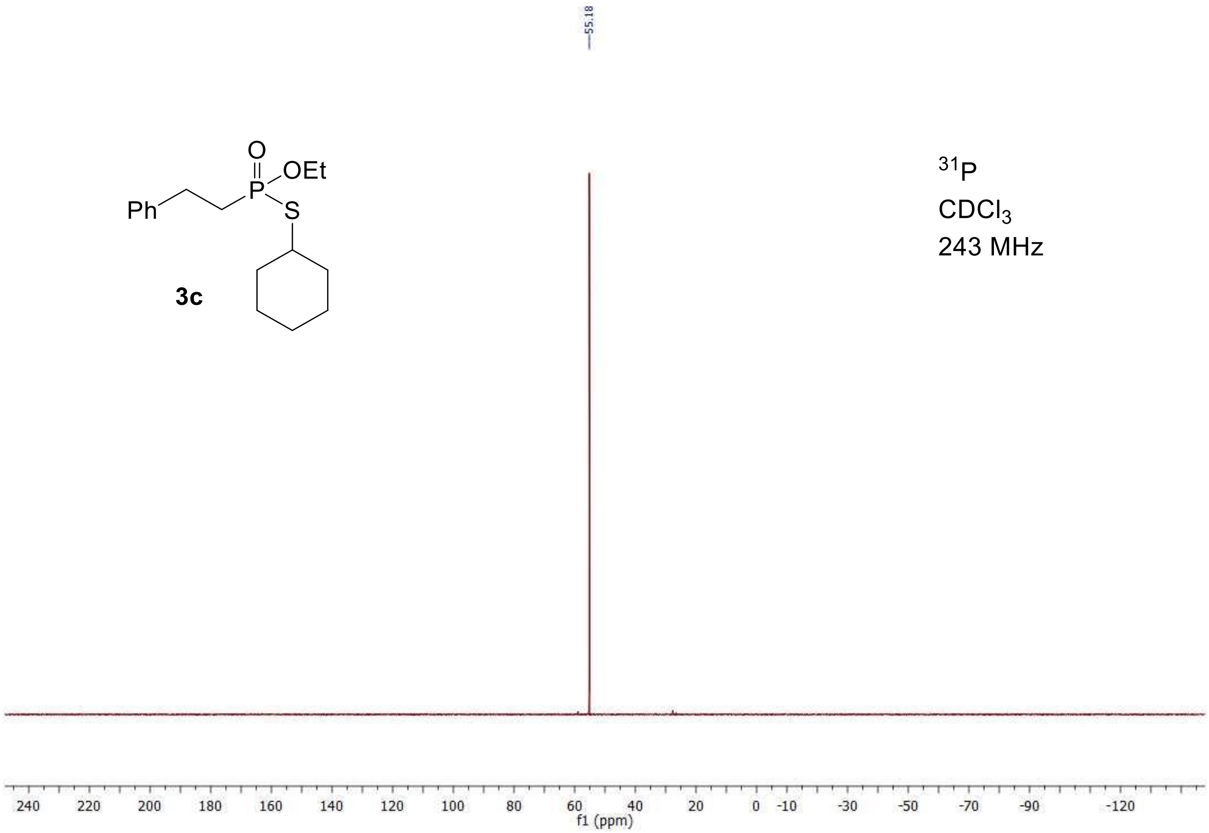

^{31}P
$CDCl_3$
243 MHz

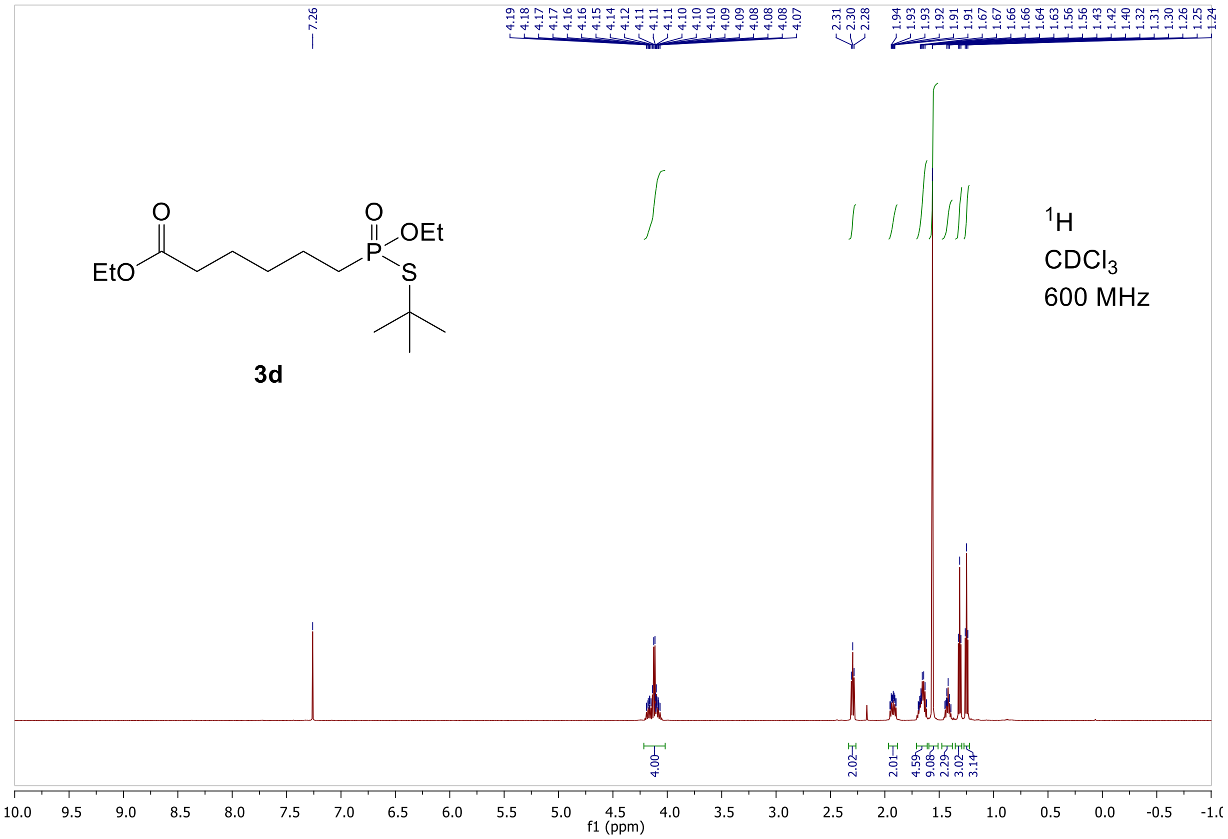

7.26
4.19
4.18
4.17
4.16
4.15
4.14
4.12
4.11
4.11
4.11
4.10
4.10
4.09
4.09
4.08
4.08
4.07
2.31
2.30
2.28
1.94
1.93
1.93
1.92
1.91
1.91
1.67
1.66
1.64
1.63
1.56
1.56
1.43
1.42
1.40
1.32
1.31
1.30
1.26
1.25
1.24
O
O
OEt
EtO
P
S
3d
^{1}H
$CDCl_3$
600 MHz
4.00
2.02
2.01
4.59
9.08
2.29
3.02
3.14
f1 (ppm)

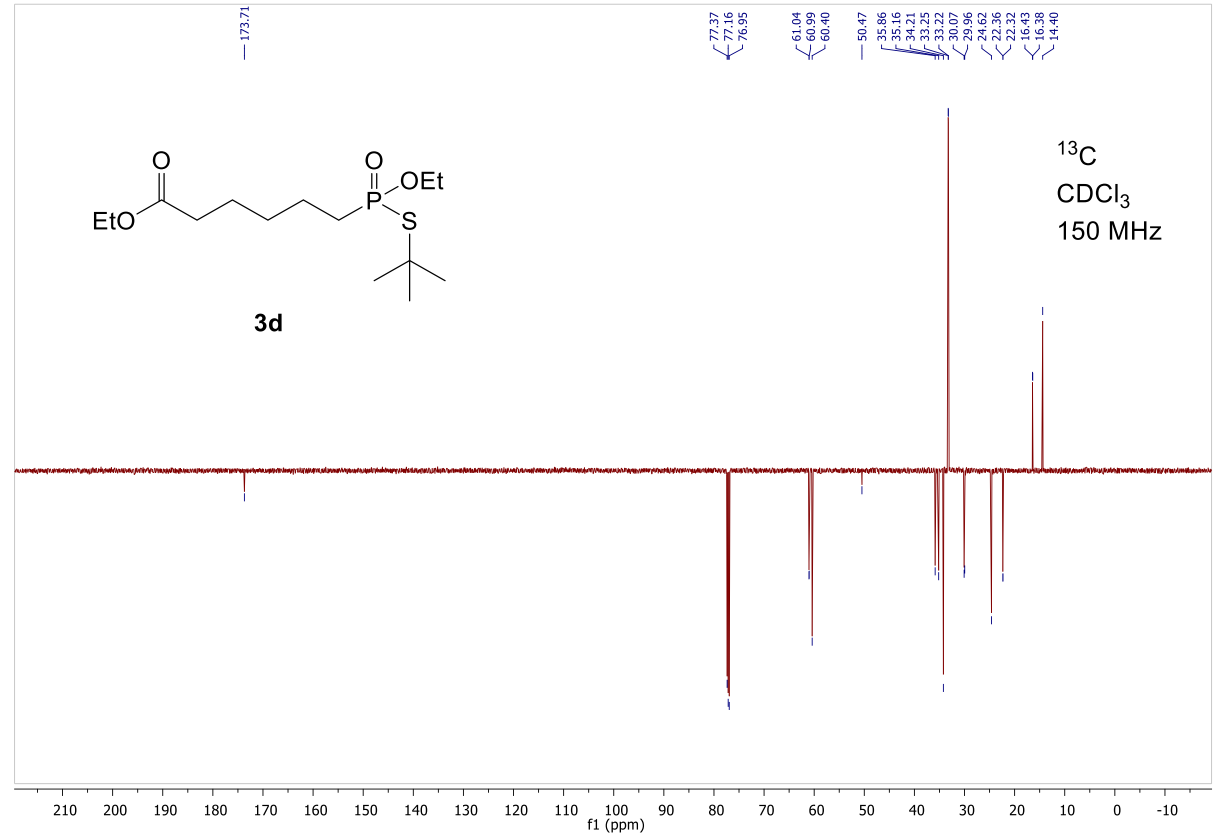

173.71
77.37
77.16
76.95
61.04
60.99
60.40
50.47
35.86
35.16
34.21
33.25
33.22
30.07
29.96
24.62
22.36
22.32
16.43
16.38
14.40
O
O
OEt
EtO
P
S
3d
^{13}C
$CDCl_3$
150 MHz
f1 (ppm)

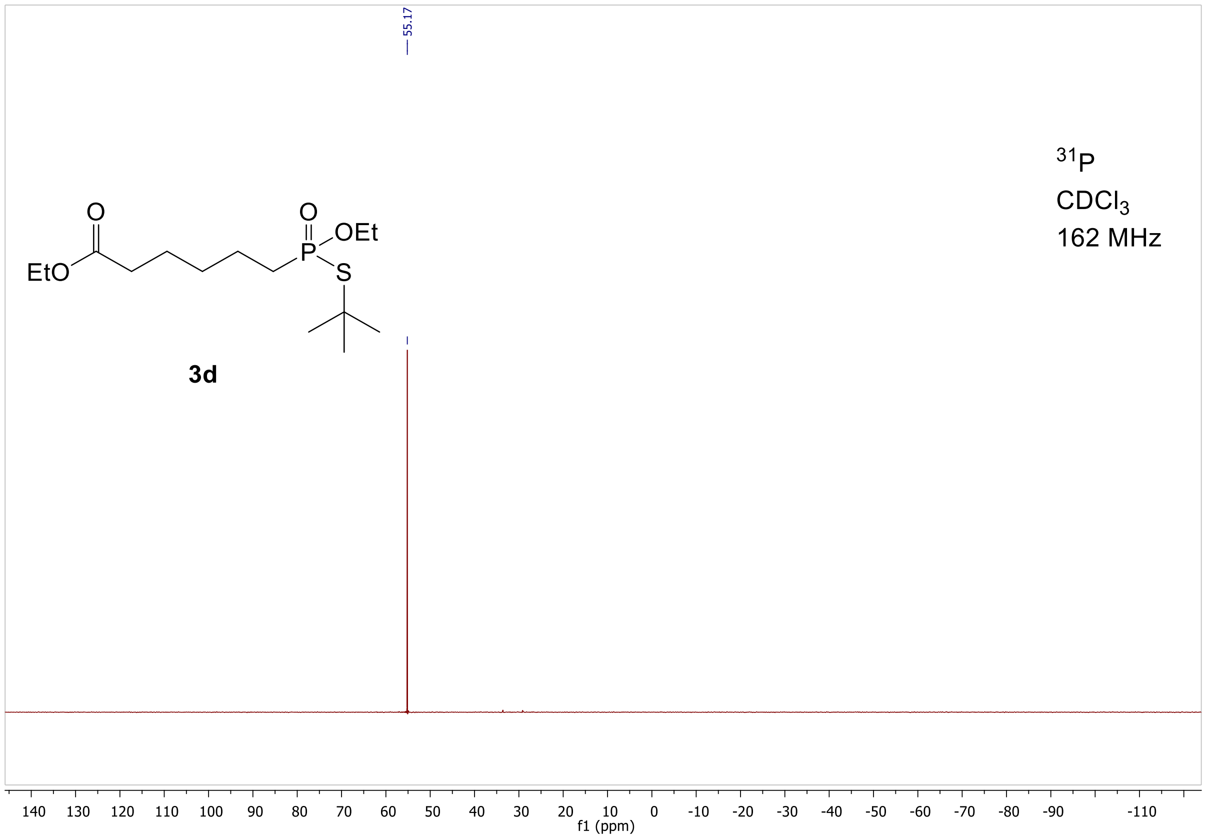

55.17
31P
CDCl3
162 MHz
O
O
OEt
EtO
P
S
3d
140 130 120 110 100 90 80 70 60 50 40 30 20 10 0 -10 -20 -30 -40 -50 -60 -70 -80 -90 -110
f1 (ppm)

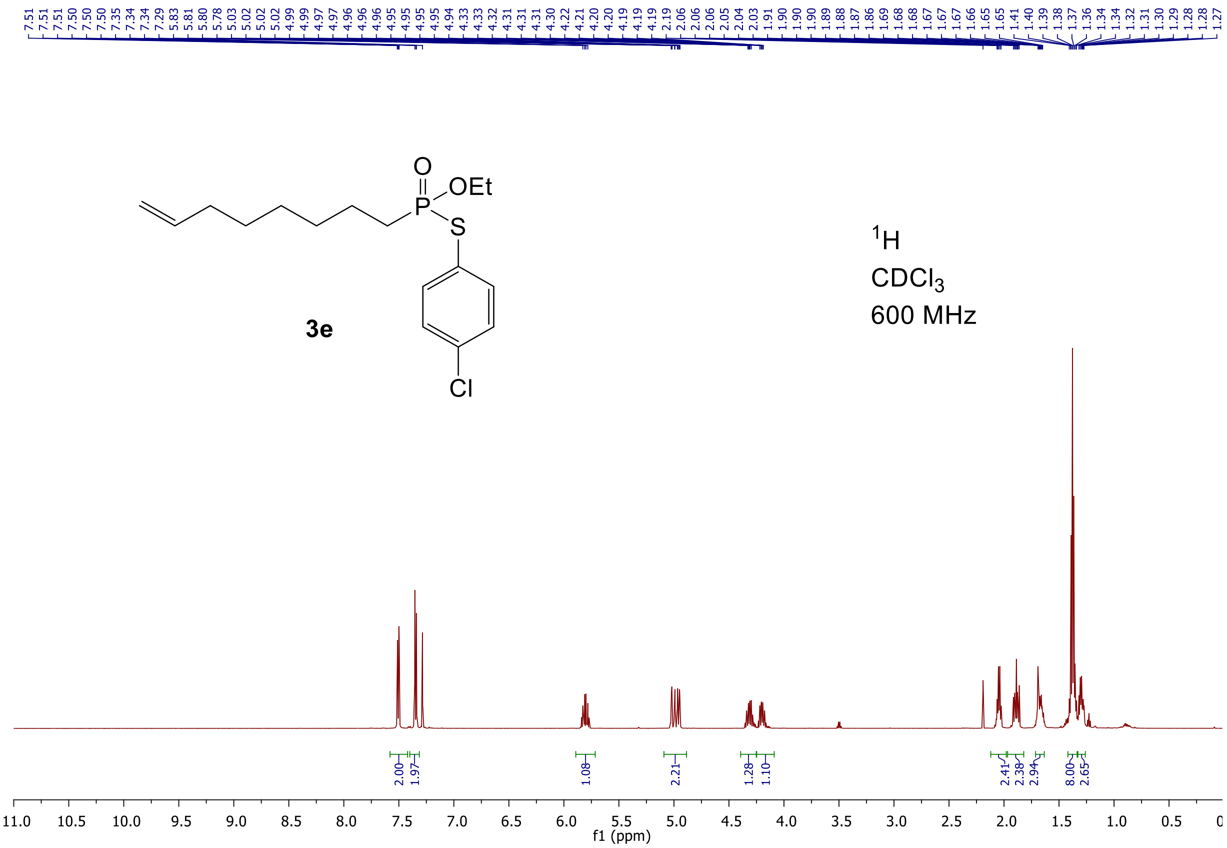

7.51 7.51 7.51 7.50 7.50 7.35 7.34 7.34 7.29 5.83 5.81 5.80 5.78 5.03 5.02 5.02 5.02 4.99 4.97 4.97 4.96 4.96 4.95 4.95 4.95 4.95 4.94 4.33 4.33 4.32 4.31 4.31 4.31 4.30 4.22 4.21 4.20 4.20 4.19 4.19 4.19 2.19 2.06 2.06 2.06 2.05 2.04 2.03 1.91 1.90 1.90 1.90 1.89 1.88 1.87 1.86 1.69 1.68 1.68 1.67 1.67 1.66 1.65 1.65 1.41 1.40 1.39 1.38 1.37 1.36 1.34 1.34 1.32 1.31 1.30 1.29 1.28 1.27
O
OEt
P
S
3e
Cl
1H
CDCl3
600 MHz
2.00 1.97 1.08 2.21 1.28 1.10 2.41 2.38 2.94 8.00 2.65
11.0 10.5 10.0 9.5 9.0 8.5 8.0 7.5 7.0 6.5 6.0 5.5 5.0 4.5 4.0 3.5 3.0 2.5 2.0 1.5 1.0 0.5 0
f1 (ppm)

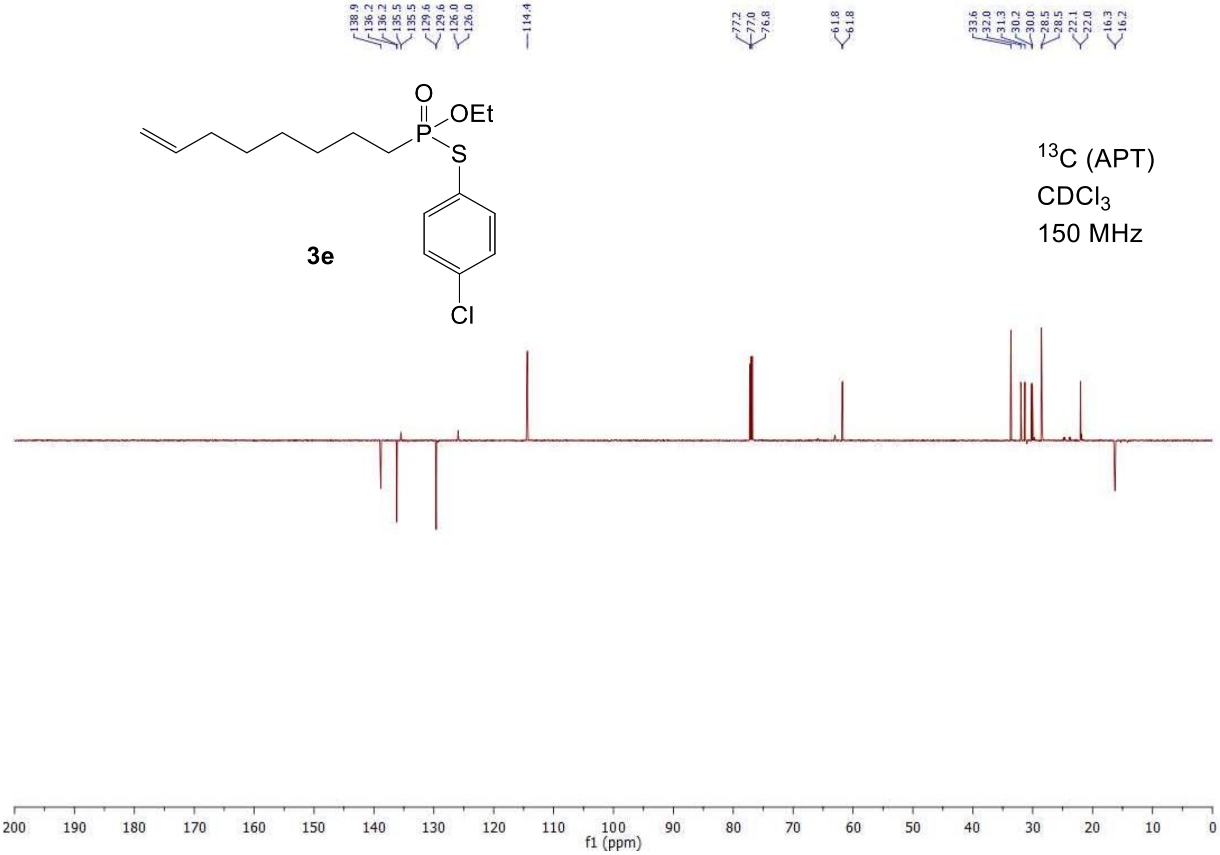

138.9 136.2 136.2 135.5 135.5 129.6 129.6 126.0 126.0 114.4 77.2 77.0 76.8 61.8 61.8 33.6 32.0 31.3 30.2 30.0 28.5 28.5 22.1 22.0 16.3 16.2
O
OEt
P
S
3e
Cl
13C (APT)
CDCl3
150 MHz
200 190 180 170 160 150 140 130 120 110 100 90 80 70 60 50 40 30 20 10 0
f1 (ppm)

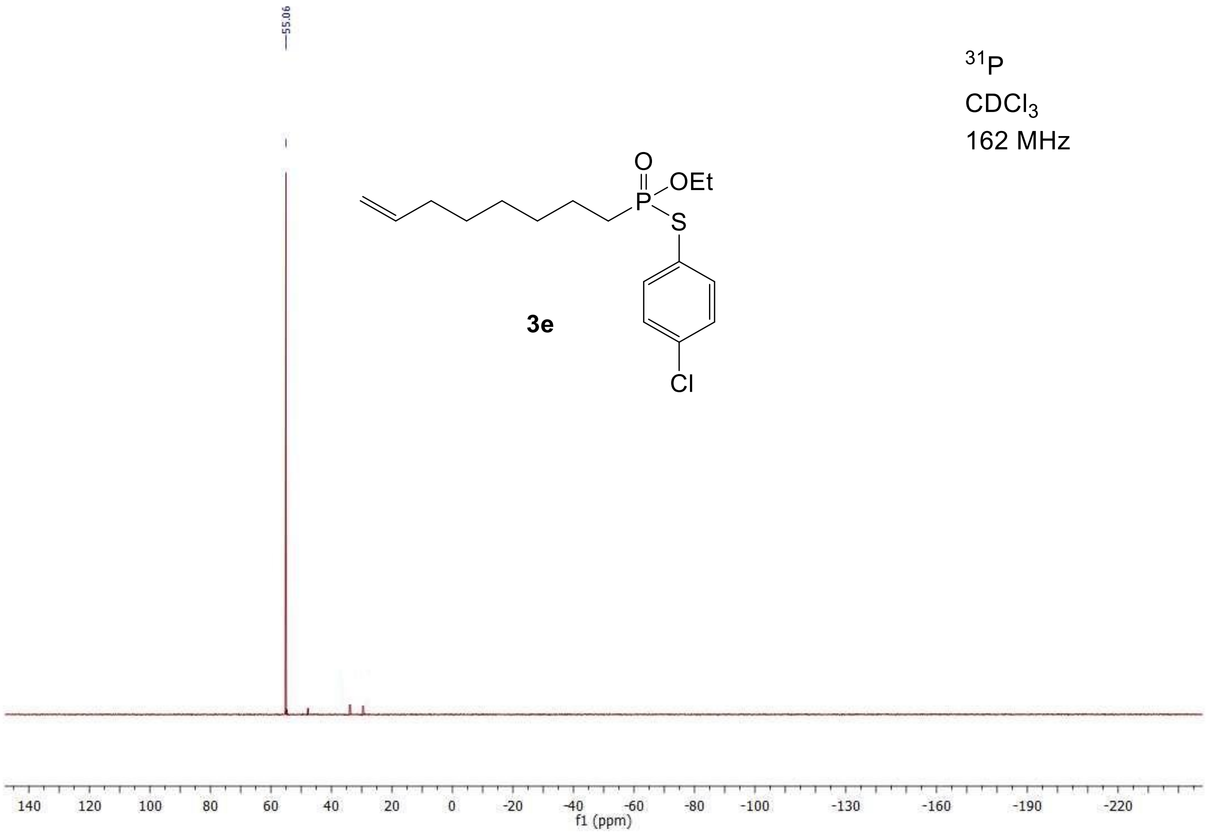

55.06
^{31}P
$CDCl_3$
162 MHz
O
OEt
P
S
Cl
3e
140 120 100 80 60 40 20 0 -20 -40 -60 -80 -100 -130 -160 -190 -220
f1 (ppm)

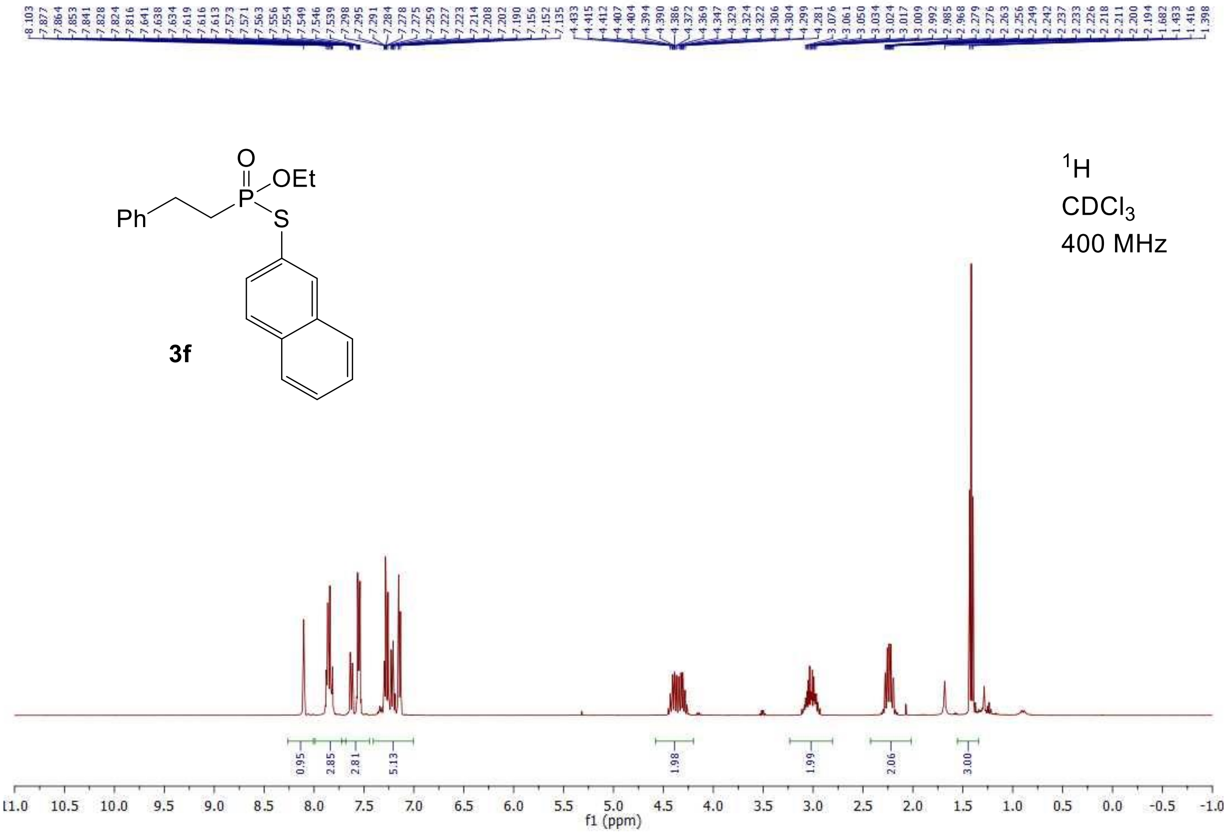

OEt
Ph
S
3f
^{1}H
CDCl$_3$
400 MHz
0.95
2.85
2.81
5.13
1.98
1.99
2.06
3.00
f1 (ppm)

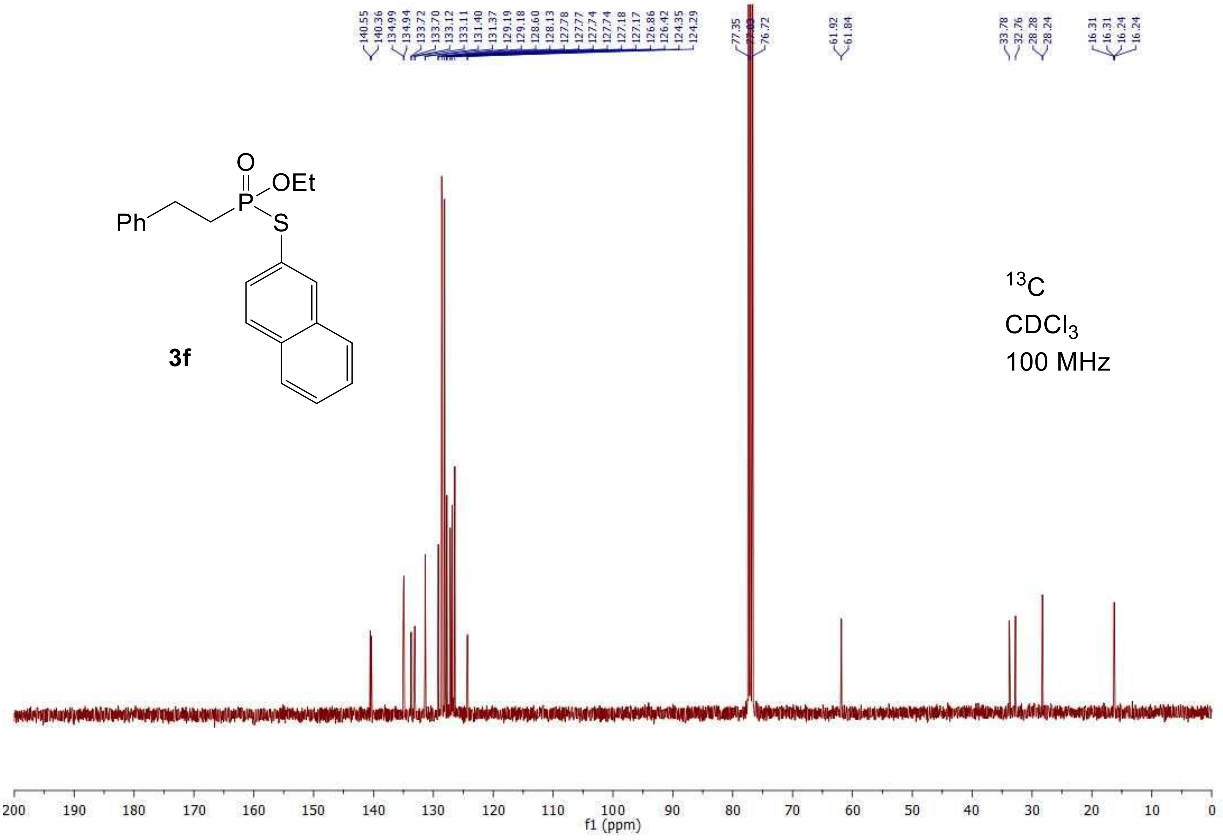

OEt
Ph
S
3f
^{13}C
CDCl$_3$
100 MHz
f1 (ppm)

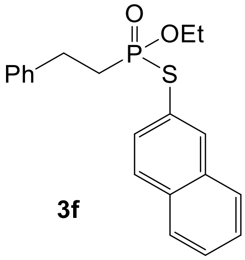

3f

^{31}P
$CDCl_3$
162 MHz

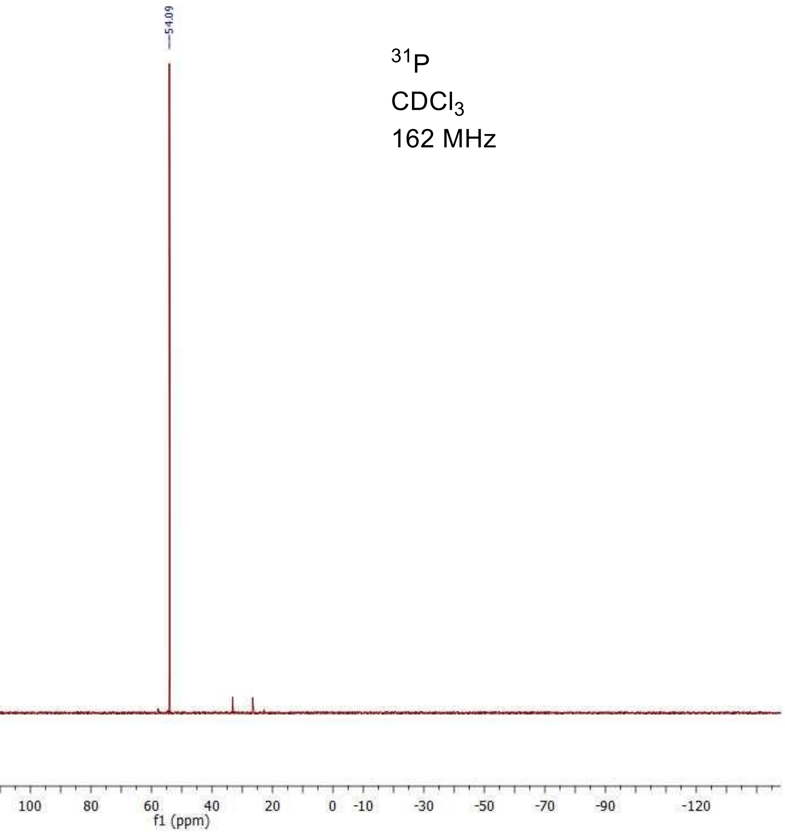

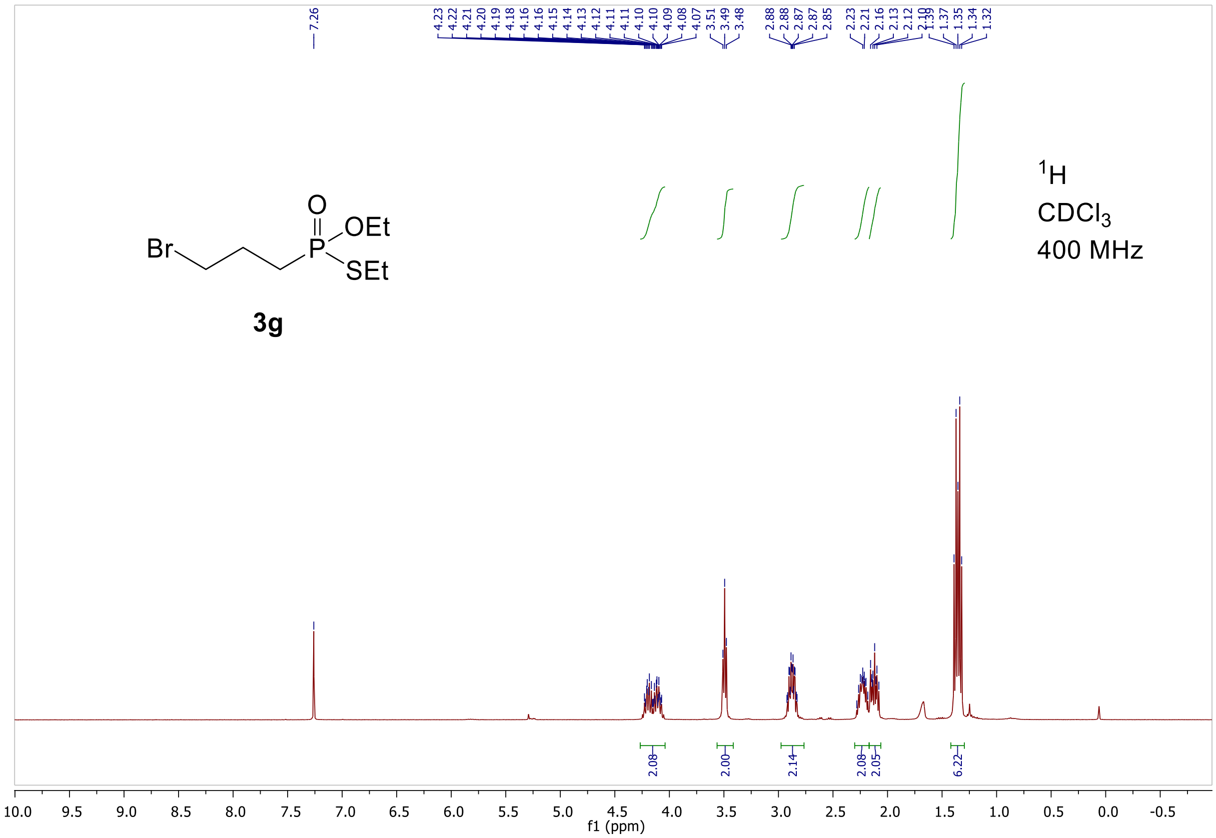
Br
O
OEt
P
SEt
3g
^{1}H
$CDCl_3$
400 MHz
f1 (ppm)

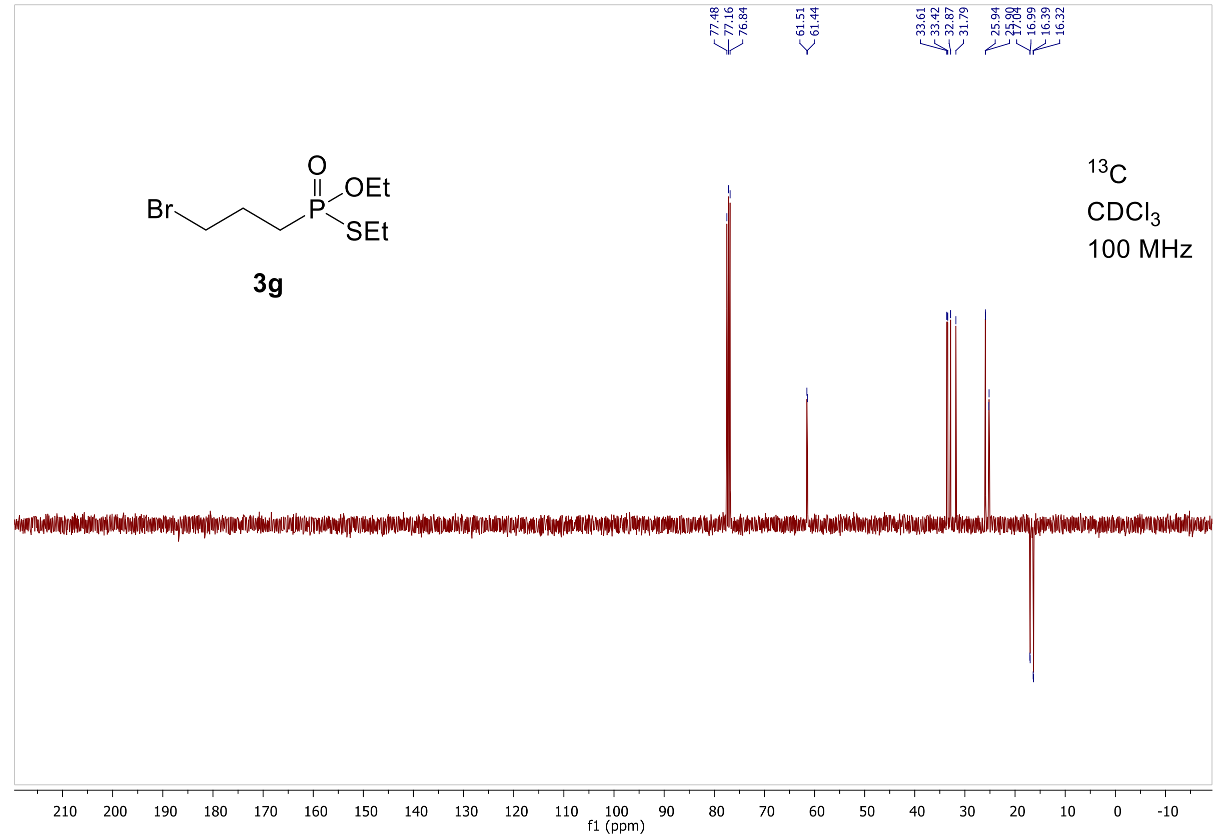
Br
O
OEt
P
SEt
3g
^{13}C
$CDCl_3$
100 MHz
f1 (ppm)

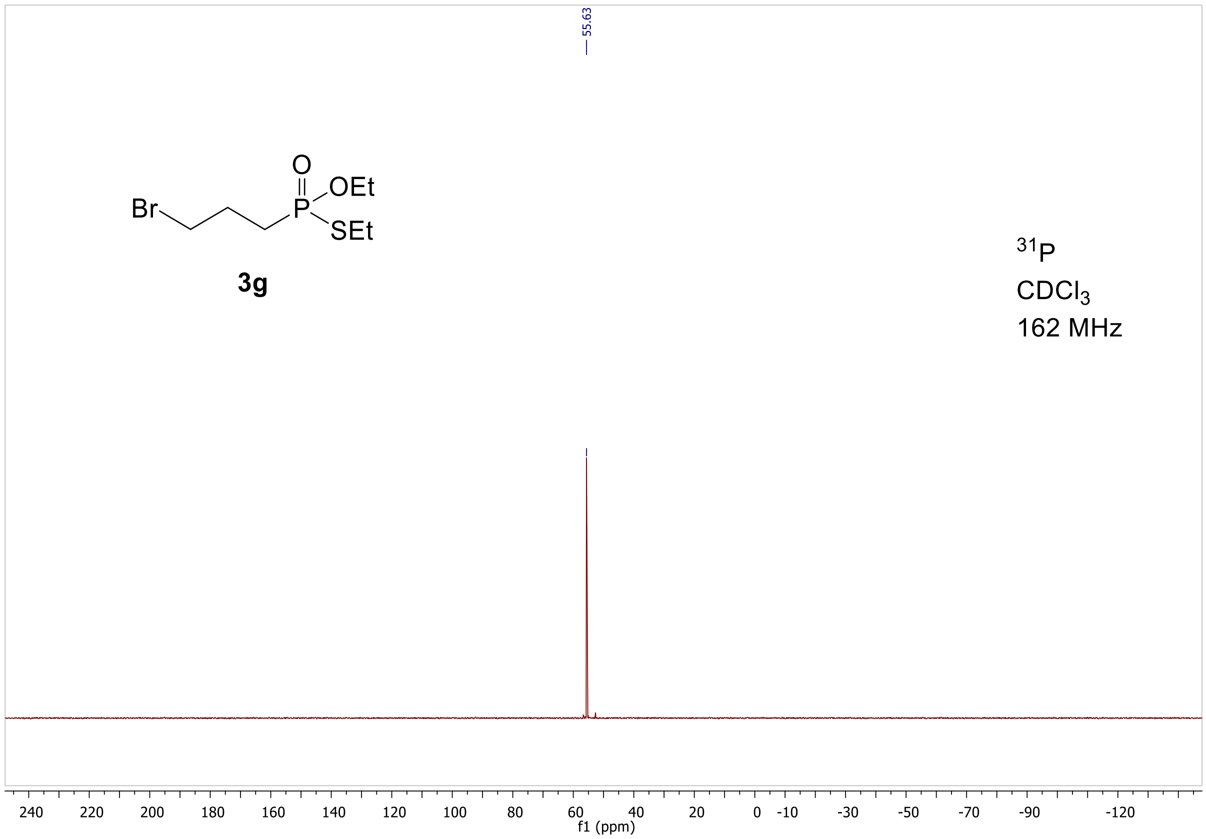

55.63
Br
O
OEt
P
SEt
3g
31P
CDCl3
162 MHz
240 220 200 180 160 140 120 100 80 60 40 20 0 -10 -30 -50 -70 -90 -120
f1 (ppm)

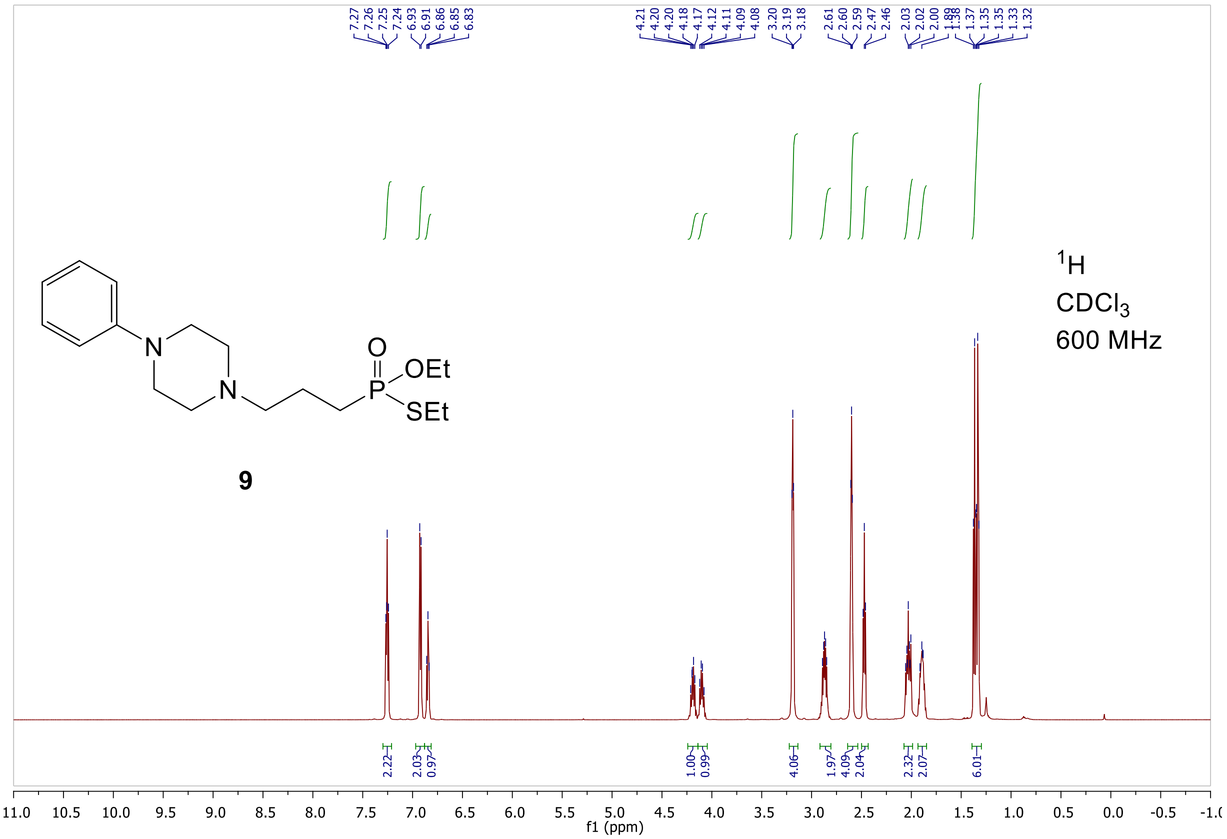
1H
CDCl3
600 MHz
9
f1 (ppm)

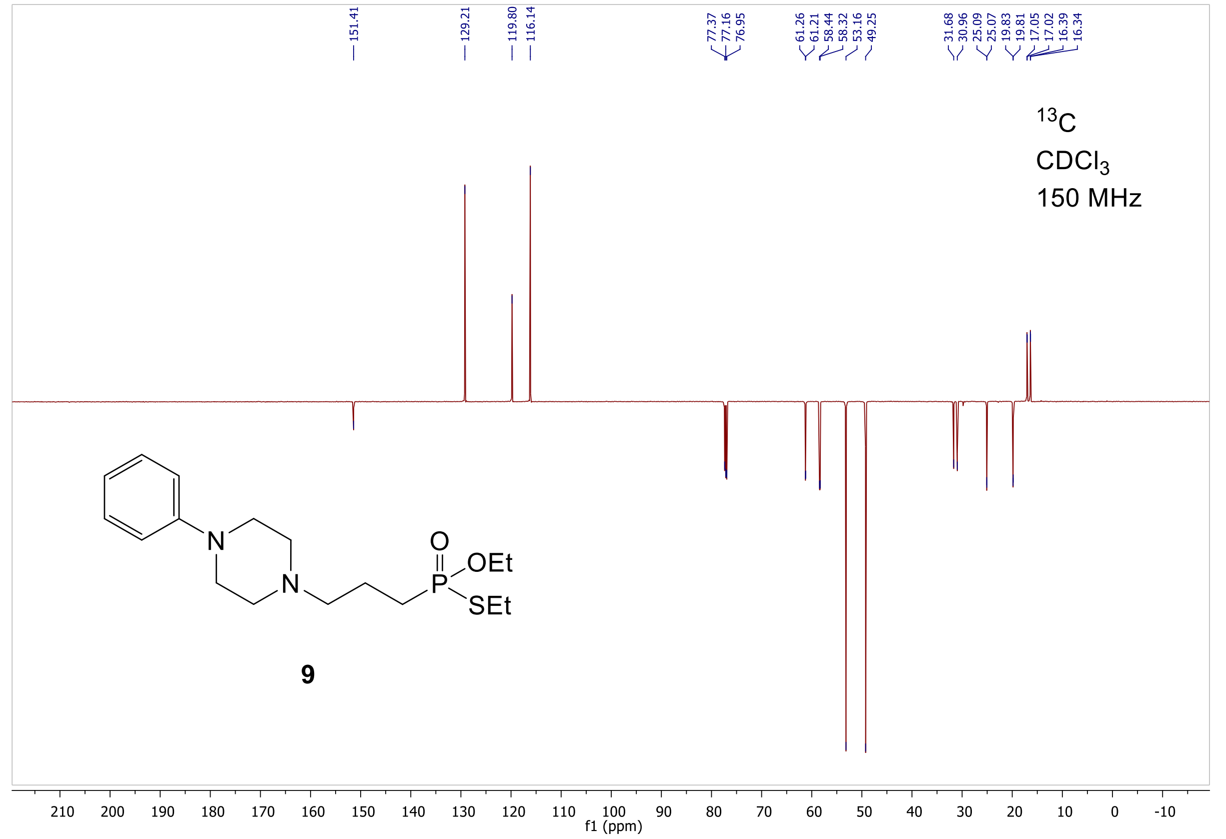
13C
CDCl3
150 MHz
9
f1 (ppm)

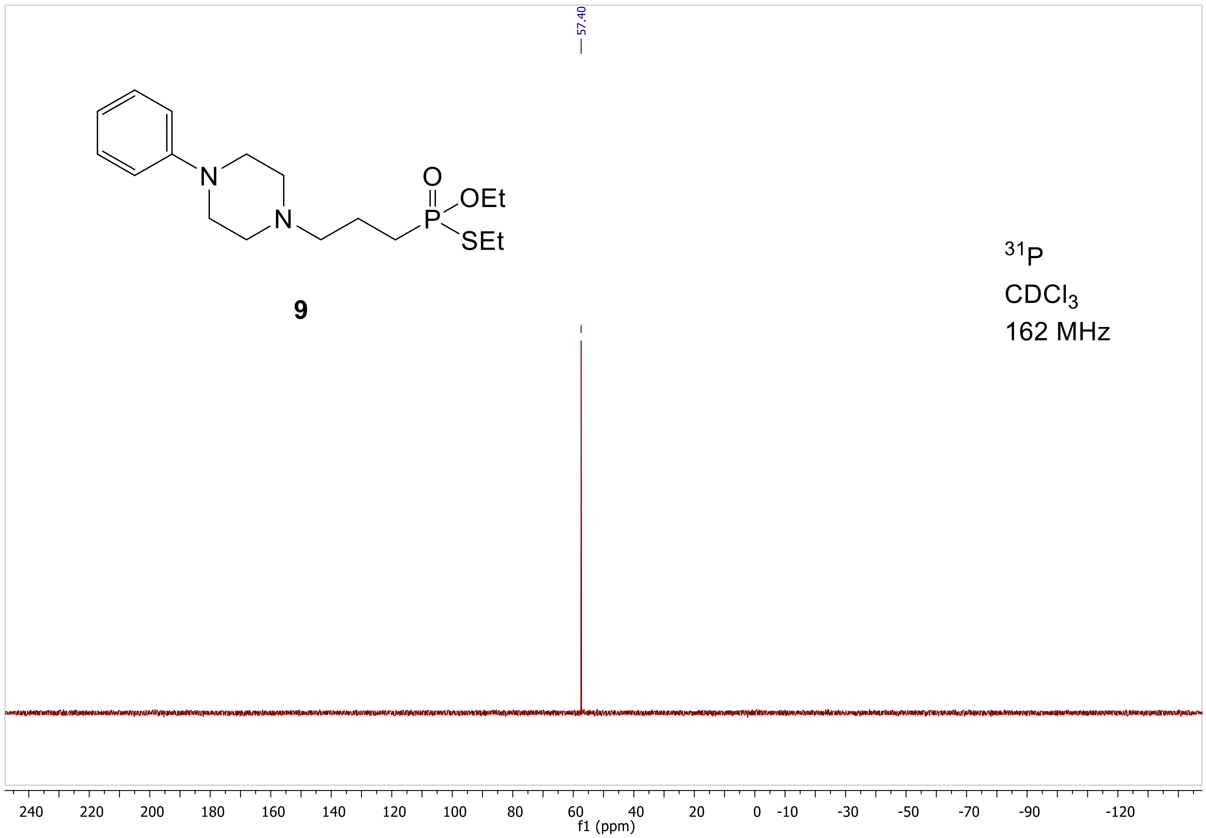

57.40
O
OEt
N
N
P
SEt
9
31P
CDCl3
162 MHz
240 220 200 180 160 140 120 100 80 60 40 20 0 -10 -30 -50 -70 -90 -120
f1 (ppm)

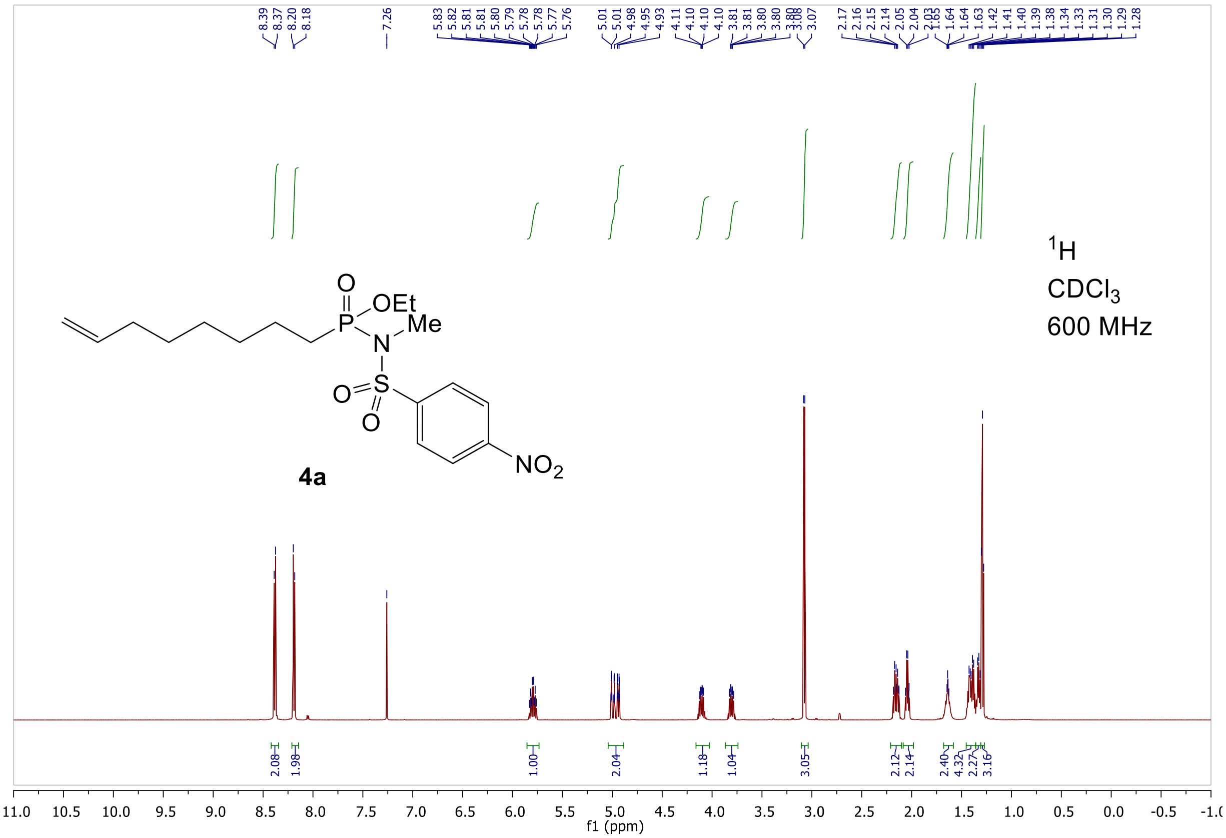

4a
^{1}H
$CDCl_3$
600 MHz
f1 (ppm)

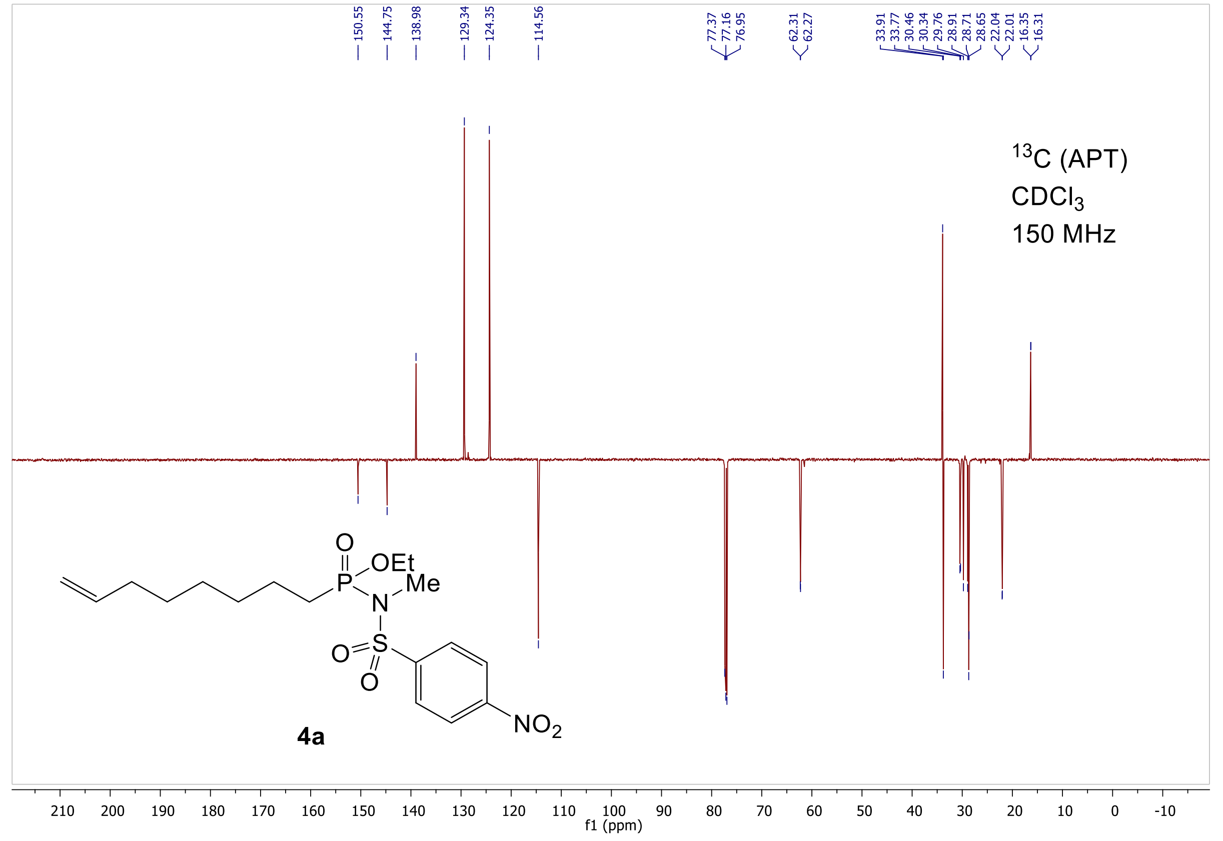

4a
^{13}C (APT)
$CDCl_3$
150 MHz
f1 (ppm)

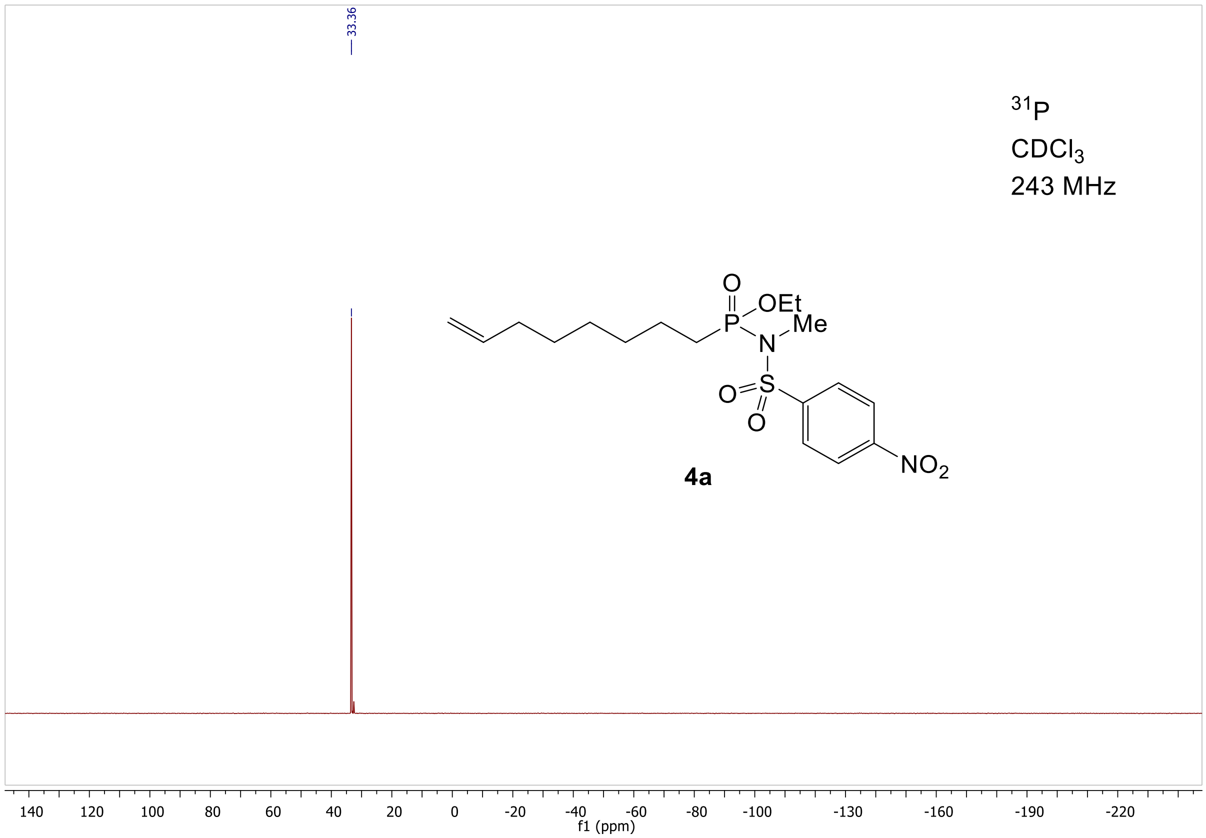

33.36
^{31}P
$CDCl_3$
243 MHz
OEt
Me
N
S
O
P
NO_2
4a
140
120
100
80
60
40
20
0
-20
-40
-60
-80
-100
-130
-160
-190
-220
f1 (ppm)

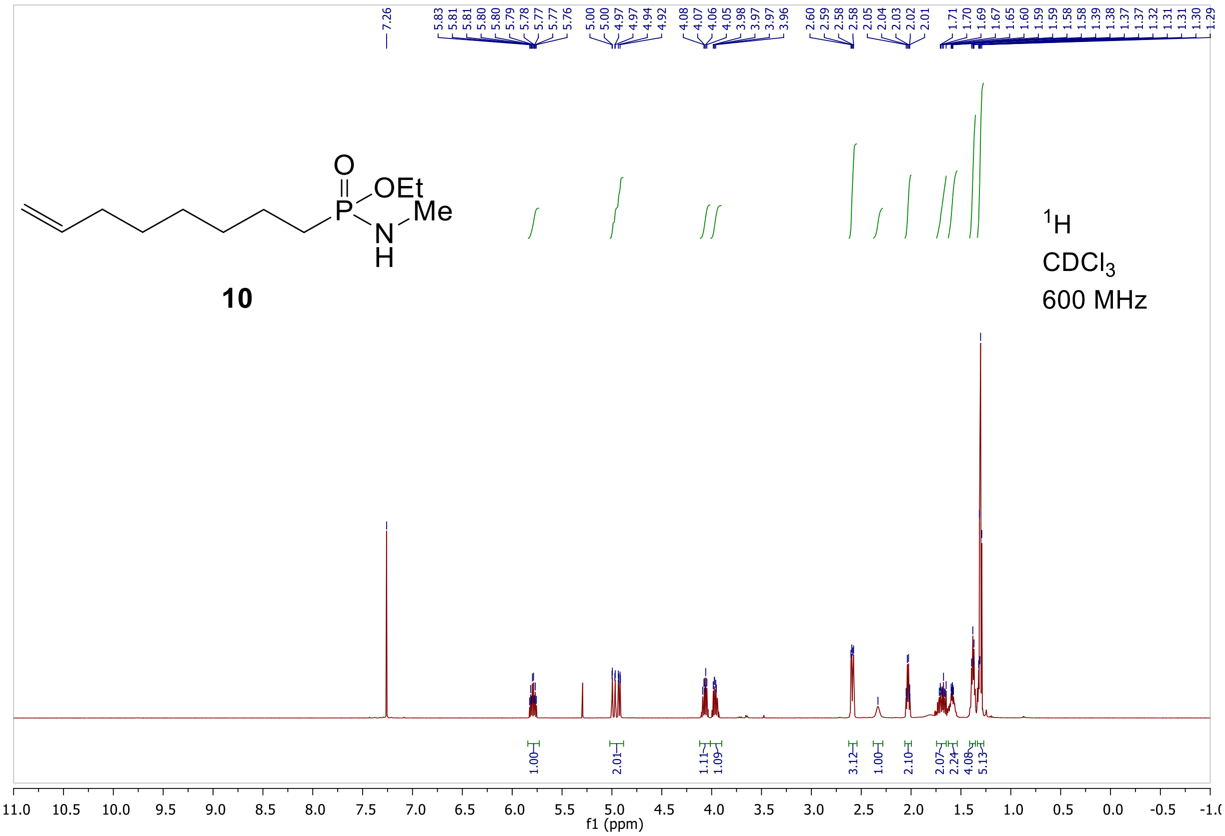

10
^{1}H
CDCl$_3$
600 MHz
f1 (ppm)

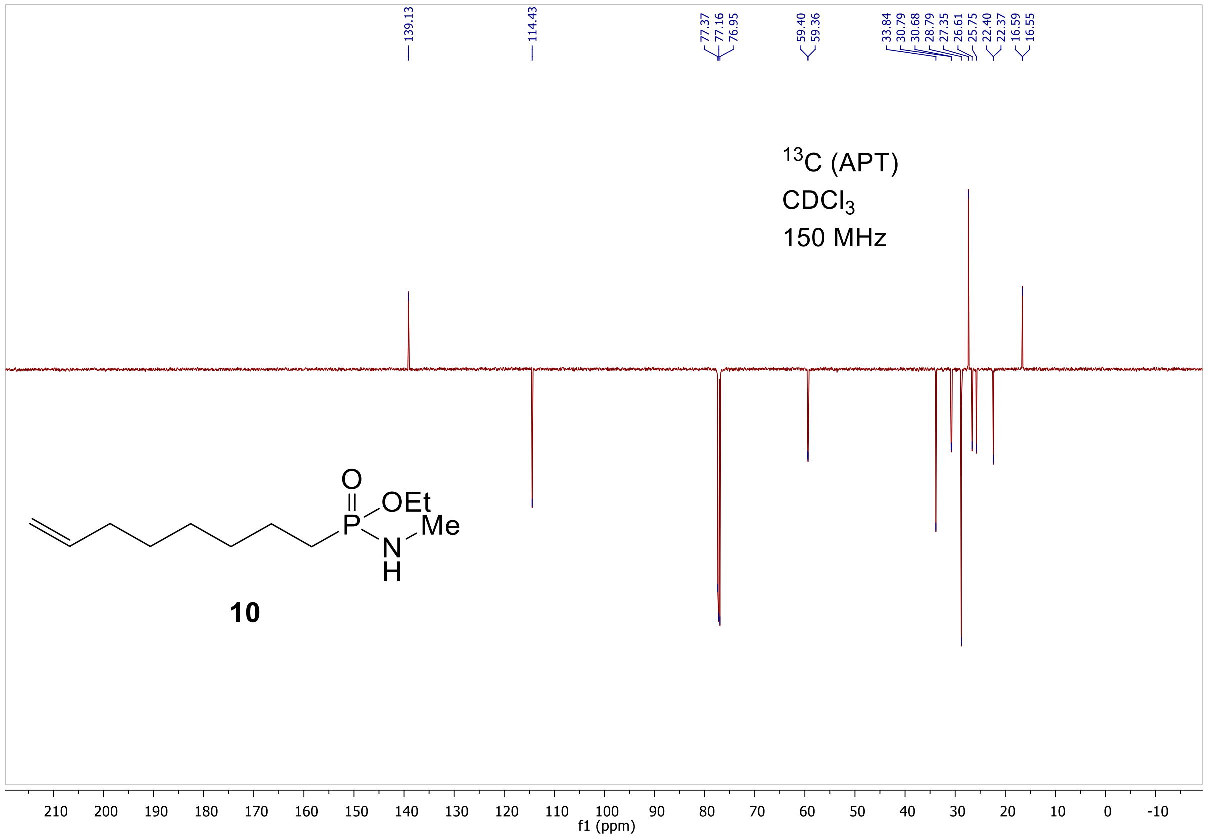

10
^{13}C (APT)
CDCl$_3$
150 MHz
f1 (ppm)

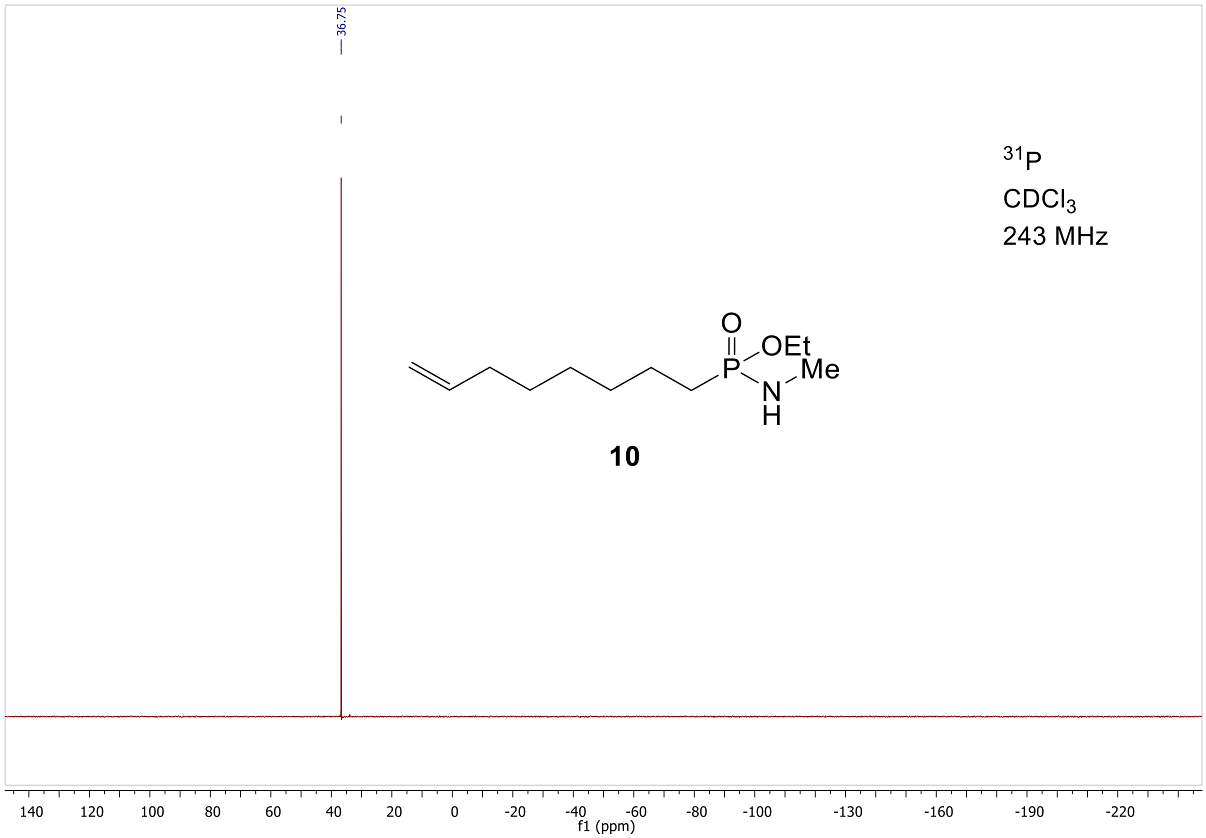
36.75
31P
CDCl3
243 MHz
O
OEt
P
N
Me
H
10
140 120 100 80 60 40 20 0 -20 -40 -60 -80 -100 -130 -160 -190 -220
f1 (ppm)

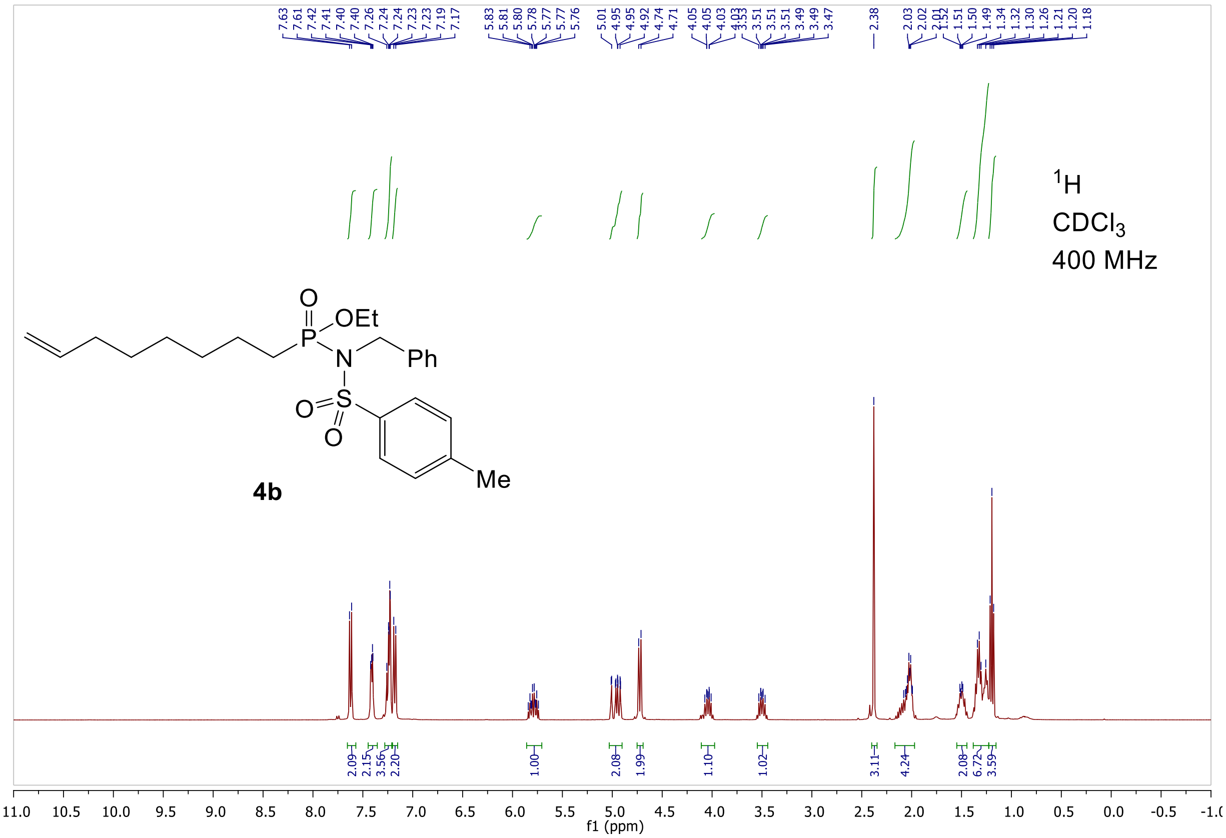

7.63
7.61
7.42
7.41
7.40
7.40
7.26
7.24
7.24
7.23
7.23
7.19
7.17
5.83
5.81
5.80
5.78
5.77
5.77
5.76
5.01
4.95
4.95
4.92
4.74
4.71
4.05
4.05
4.03
3.51
3.51
3.51
3.49
3.49
3.47
2.38
2.03
2.02
1.52
1.51
1.50
1.49
1.34
1.32
1.30
1.26
1.21
1.20
1.18
^{1}H
$CDCl_3$
400 MHz
O
OEt
P
N
Ph
S
O
O
Me
4b
2.09
2.15
3.56
2.20
1.00
2.08
1.99
1.10
1.02
3.11
4.24
2.08
6.72
3.59
11.0
10.5
10.0
9.5
9.0
8.5
8.0
7.5
7.0
6.5
6.0
5.5
5.0
4.5
4.0
3.5
3.0
2.5
2.0
1.5
1.0
0.5
0.0
-0.5
f1 (ppm)

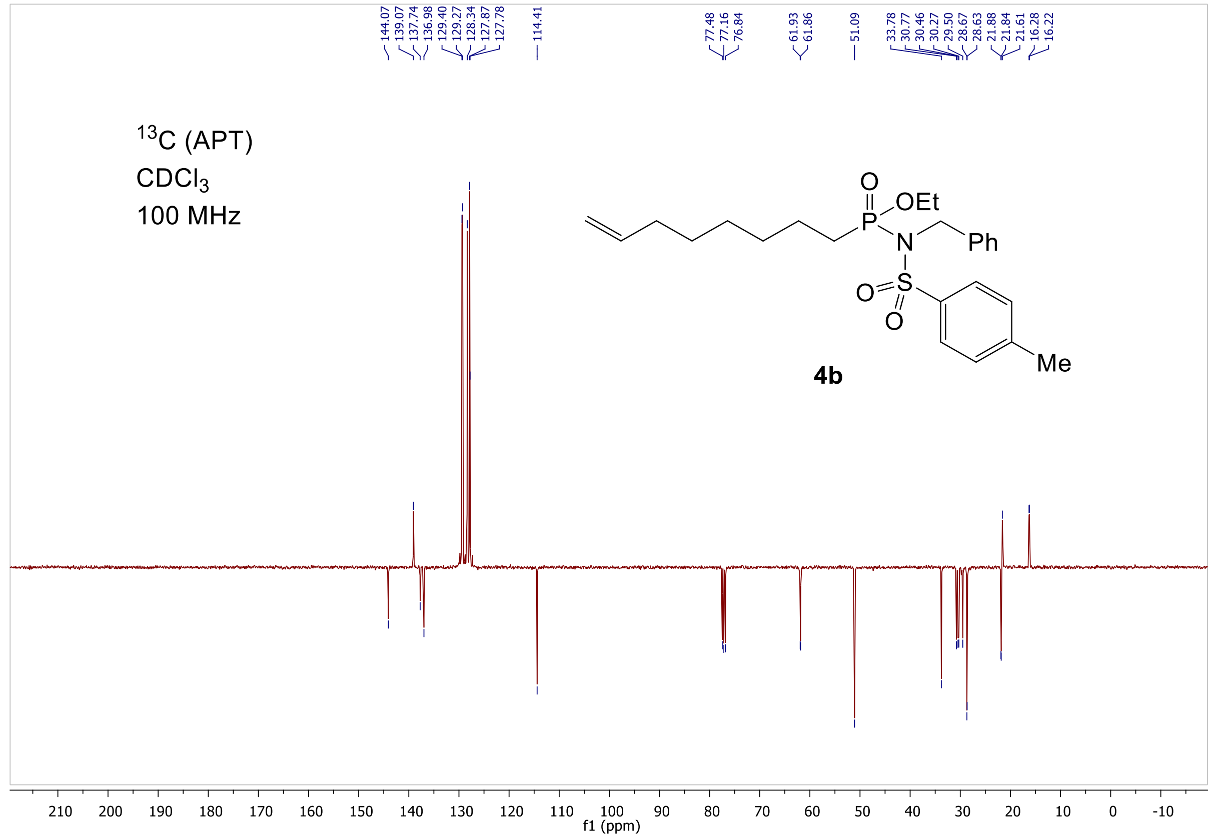

144.07
139.07
137.74
136.98
129.40
129.27
128.34
127.87
127.78
114.41
77.48
77.16
76.84
61.93
61.86
51.09
33.78
30.77
30.46
30.27
29.50
28.67
28.63
21.88
21.84
21.61
16.28
16.22
^{13}C (APT)
$CDCl_3$
100 MHz
O
OEt
P
N
Ph
S
O
O
Me
4b
210
200
190
180
170
160
150
140
130
120
110
100
90
80
70
60
50
40
30
20
10
0
-10
f1 (ppm)

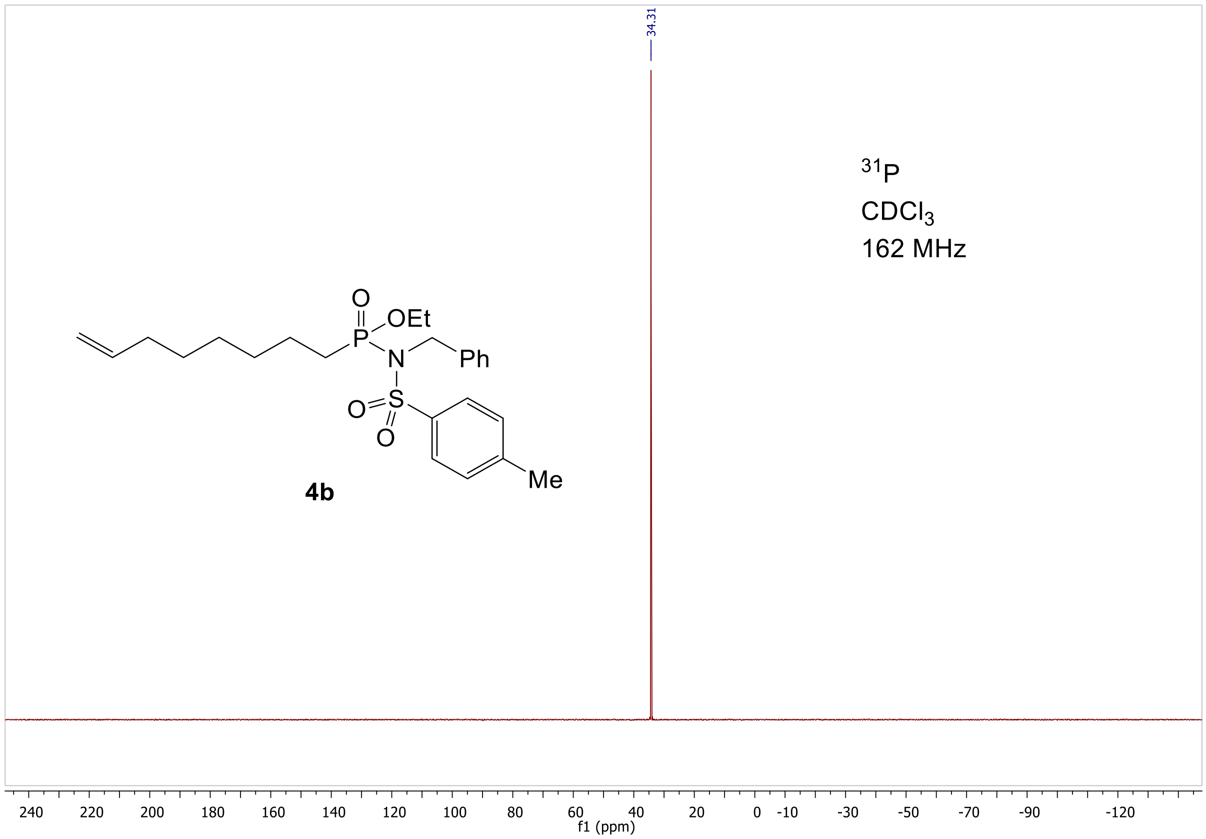

34.31
31P
CDCl3
162 MHz
OEt
Ph
Me
4b
240 220 200 180 160 140 120 100 80 60 40 20 0 -10 -30 -50 -70 -90 -120
f1 (ppm)

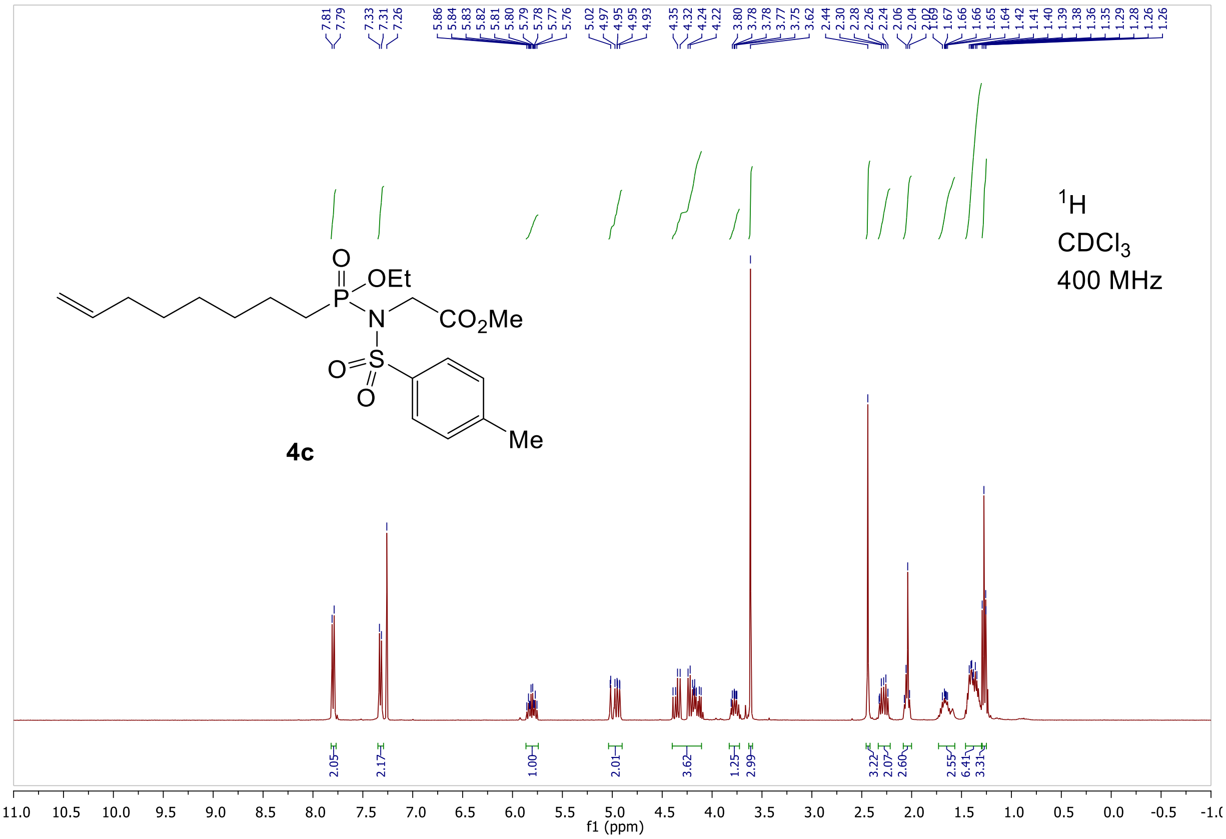

4c
1H
CDCl3
400 MHz
f1 (ppm)

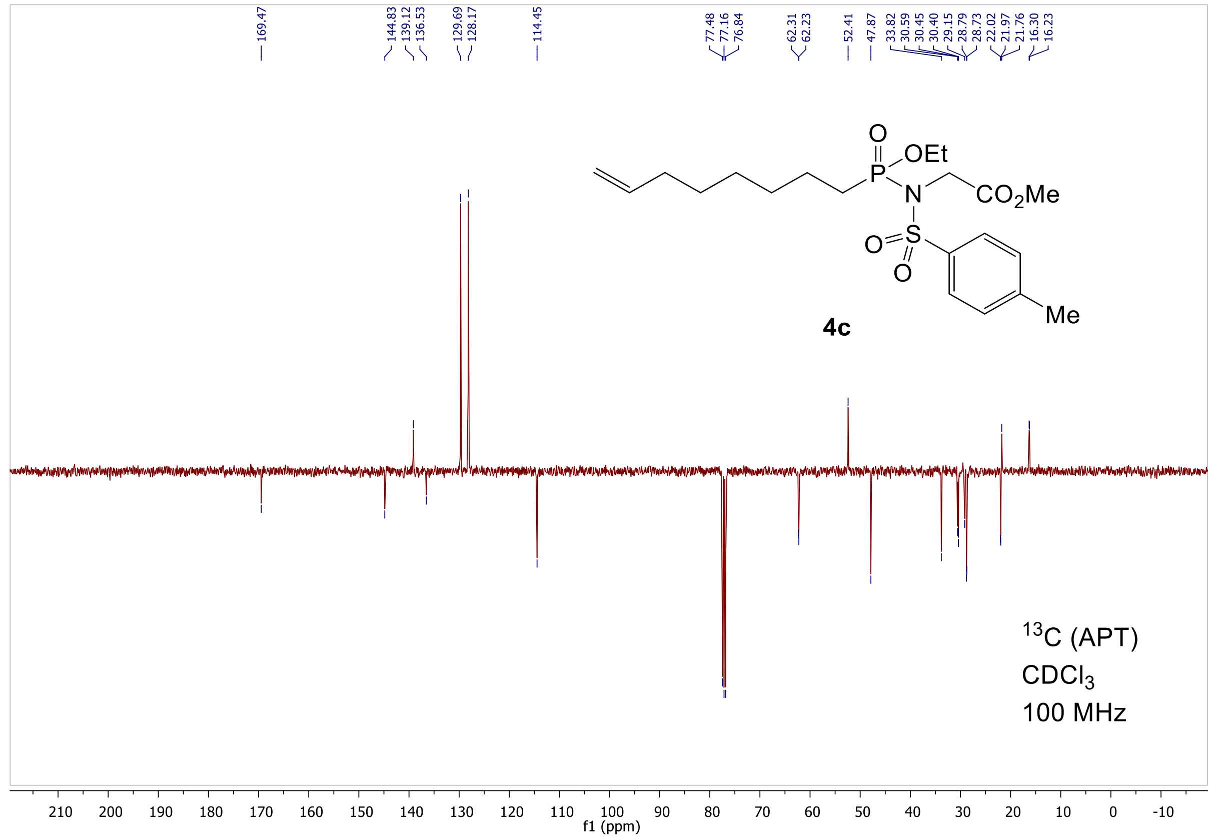

4c
13C (APT)
CDCl3
100 MHz
f1 (ppm)

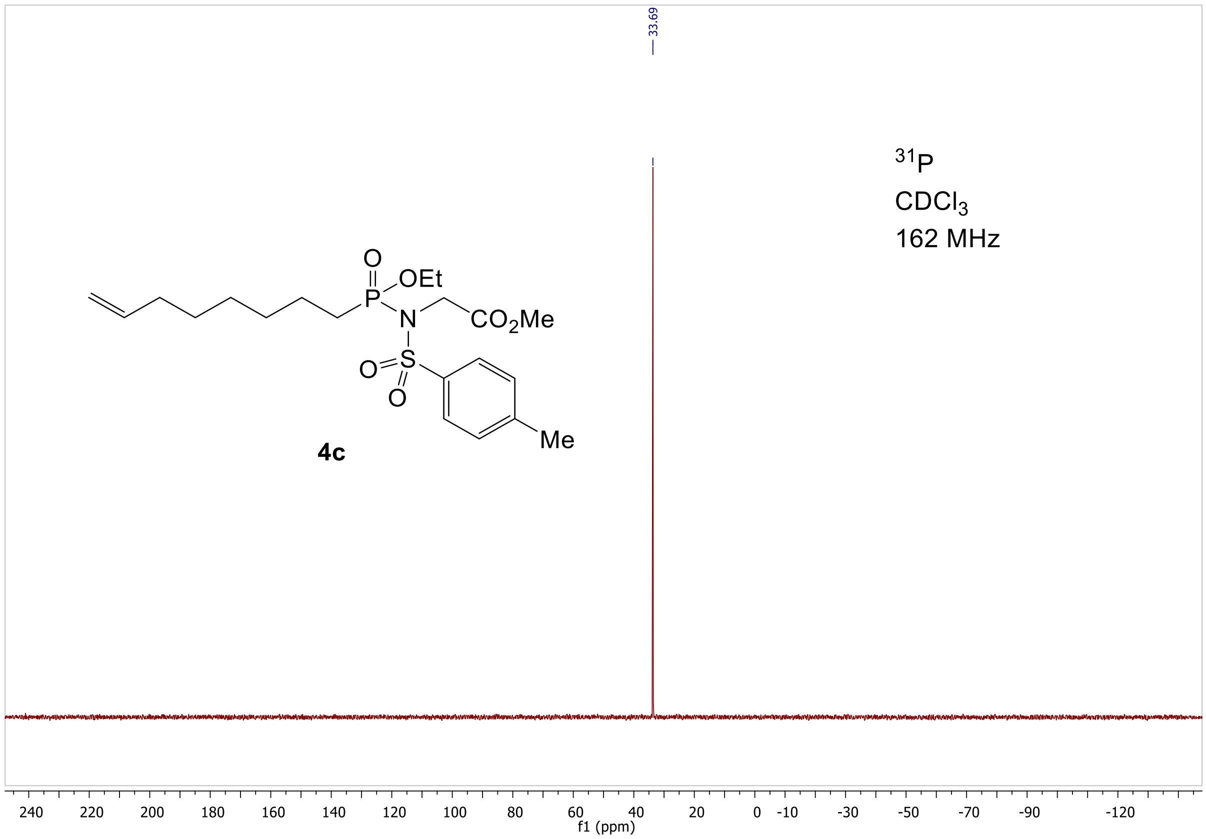
33.69
31P
CDCl3
162 MHz
OEt
CO2Me
Me
4c
240 220 200 180 160 140 120 100 80 60 40 20 0 -10 -30 -50 -70 -90 -120
f1 (ppm)

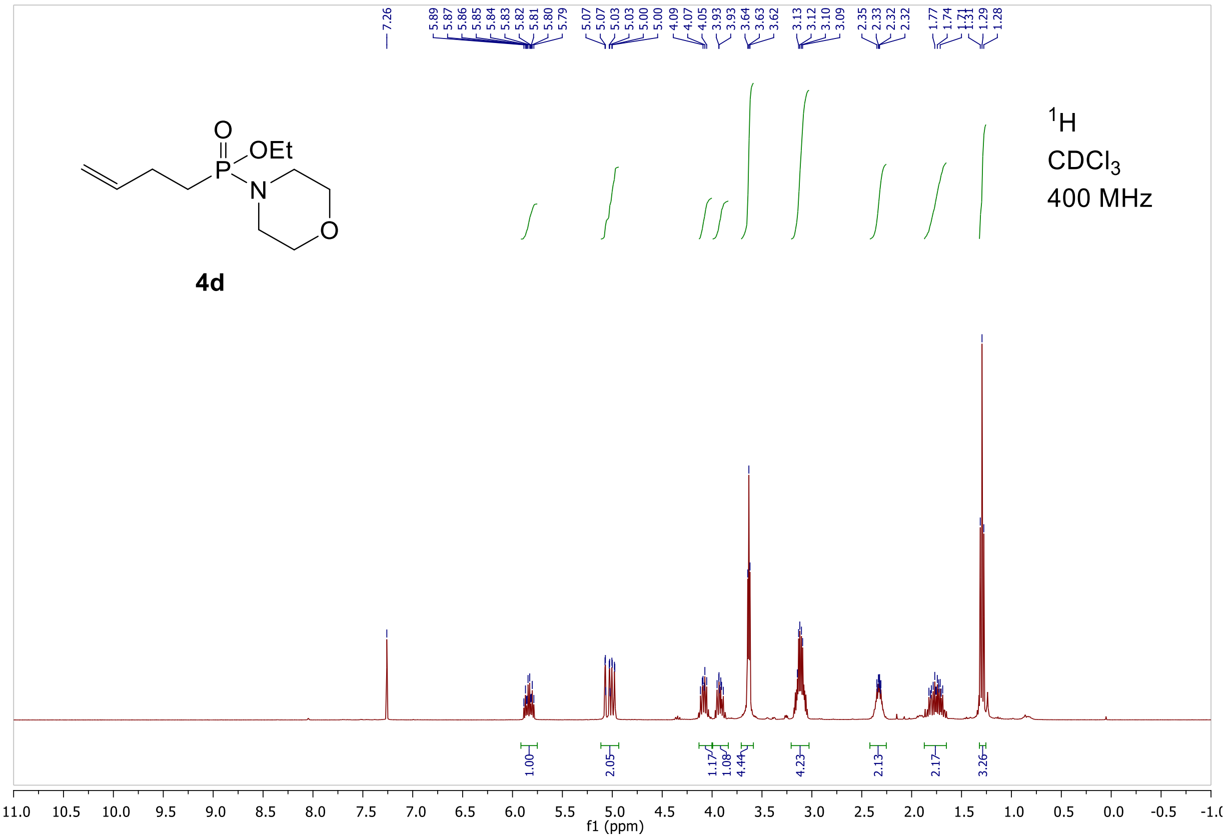

O
OEt
P
N
O
4d
1H
CDCl3
400 MHz
f1 (ppm)

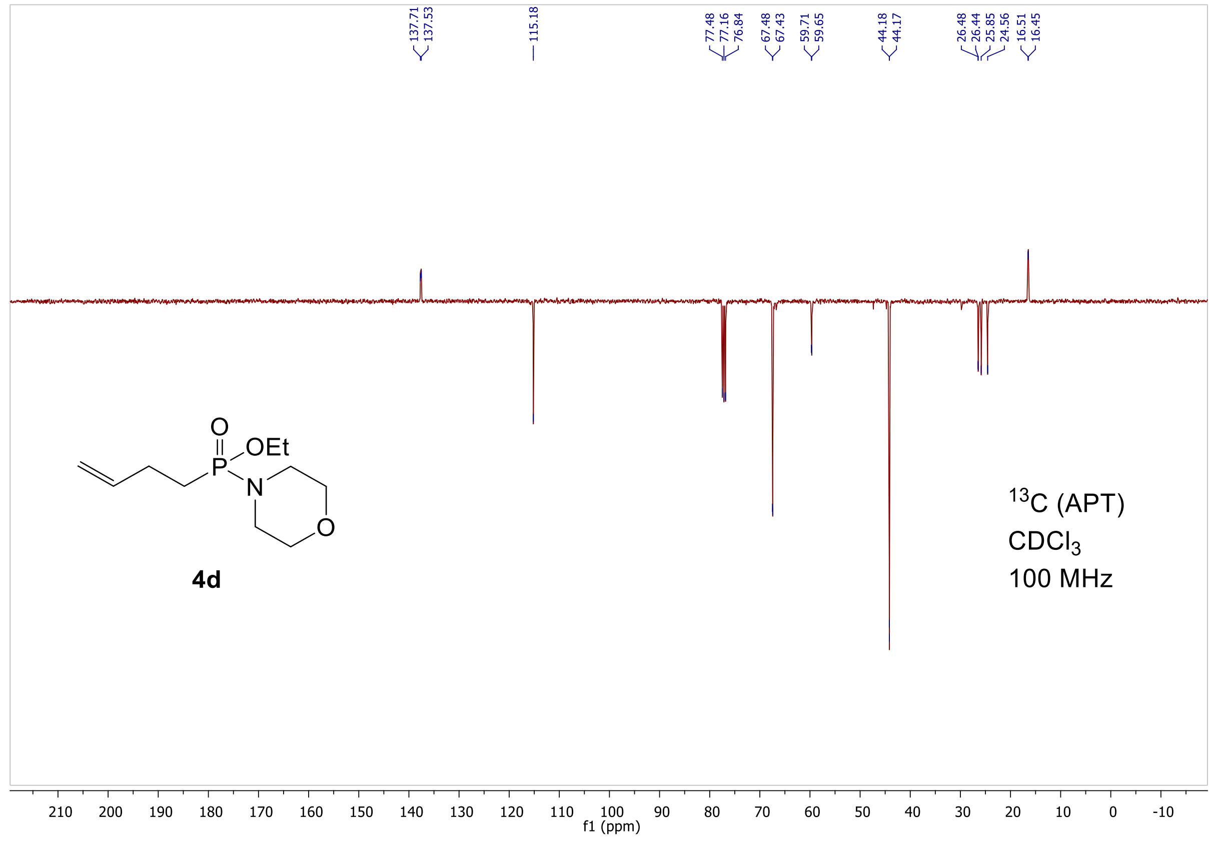

O
OEt
P
N
O
4d
13C (APT)
CDCl3
100 MHz
f1 (ppm)

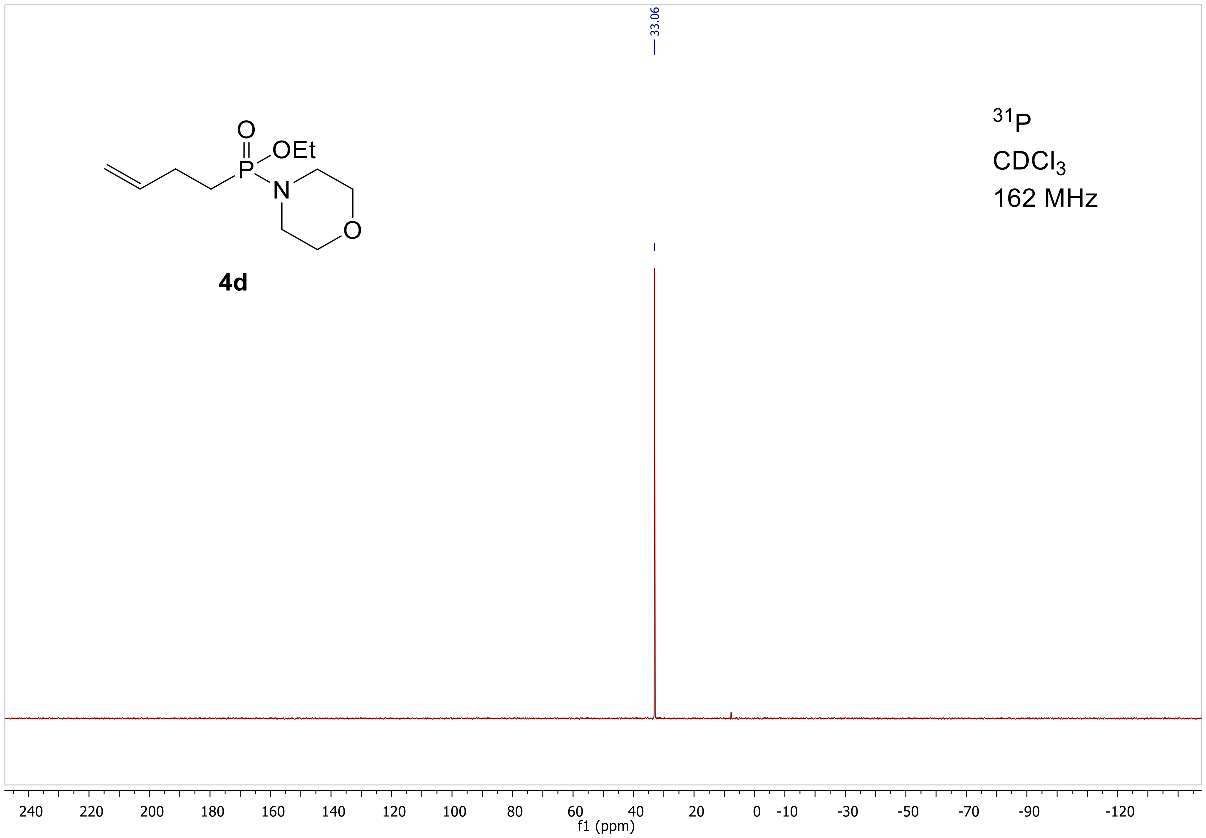
33.06
OEt
N
O
4d
31P
CDCl3
162 MHz
240
220
200
180
160
140
120
100
80
60
40
20
0
-10
-30
-50
-70
-90
-120
f1 (ppm)

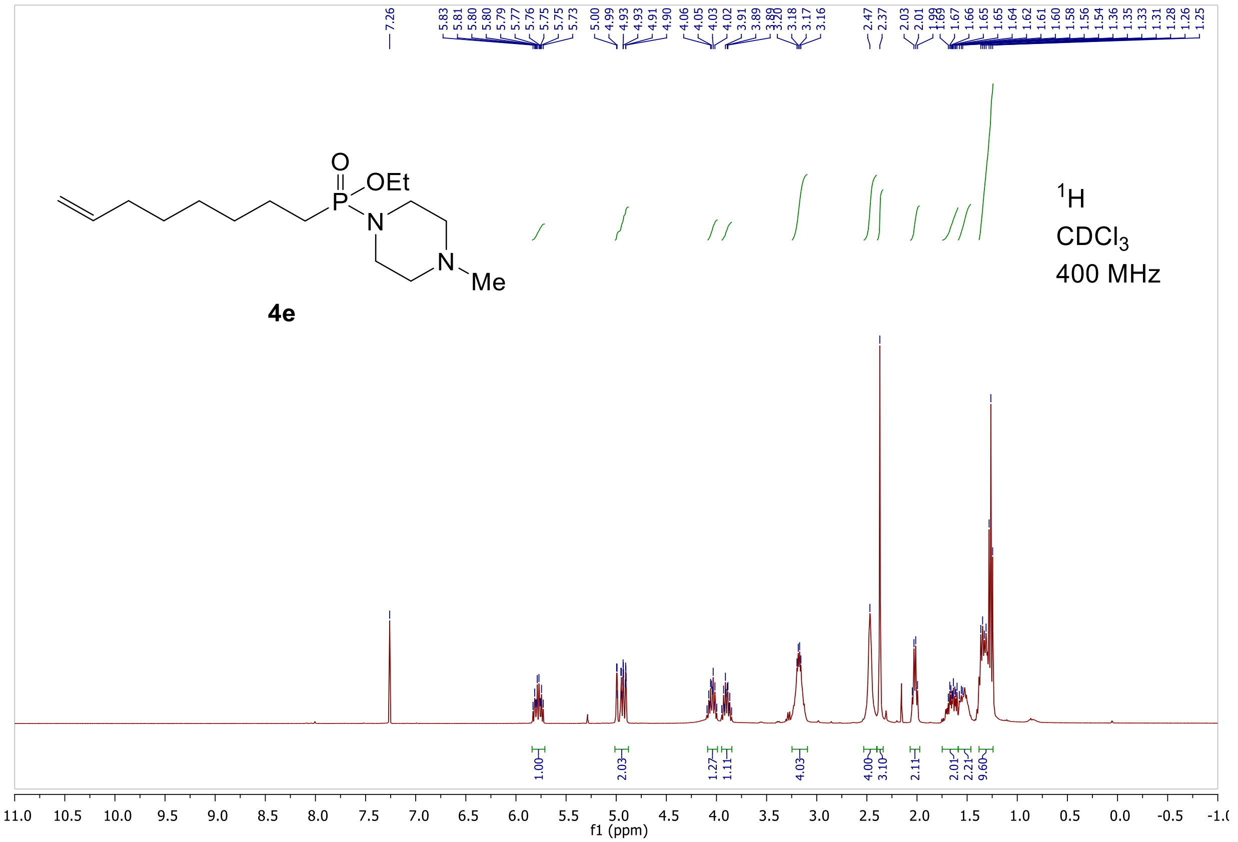

4e
^{1}H
CDCl3
400 MHz
f1 (ppm)

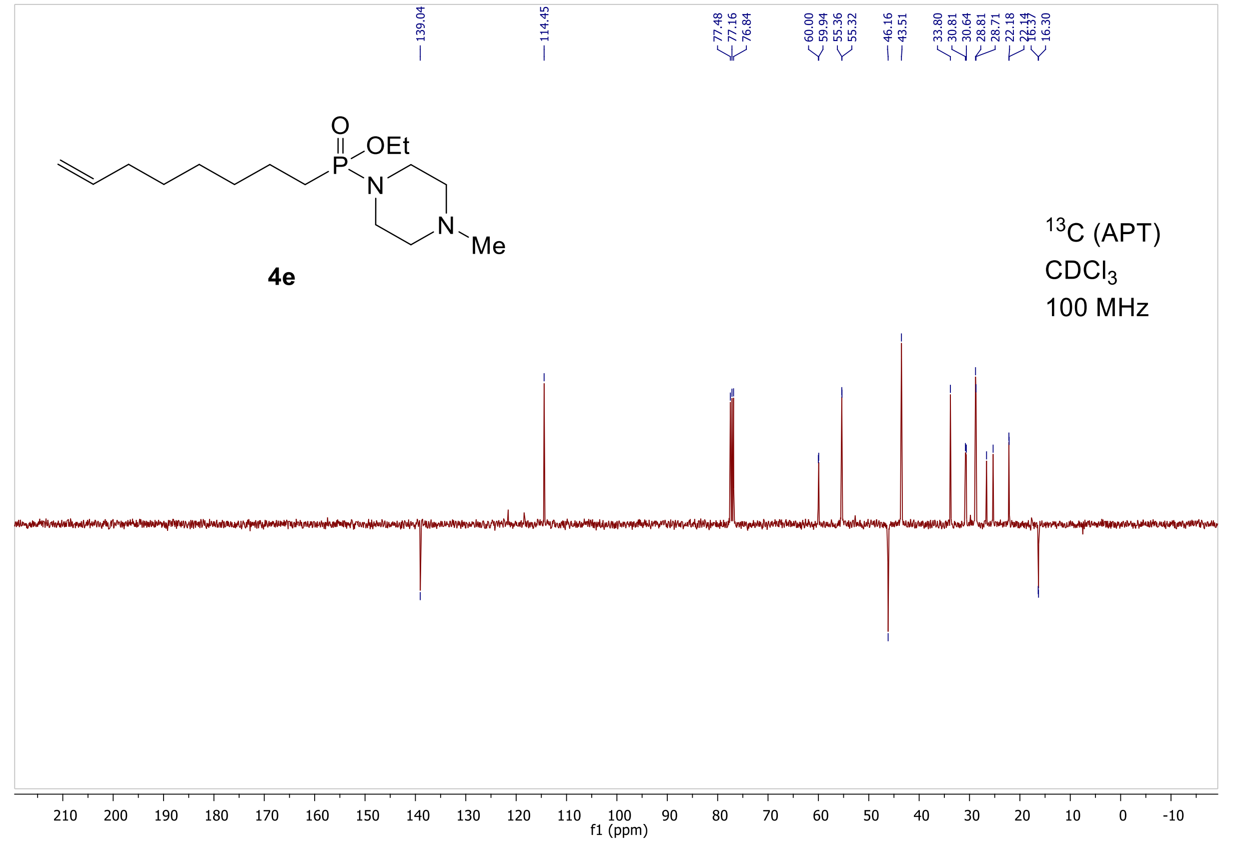

4e
^{13}C (APT)
CDCl3
100 MHz
f1 (ppm)

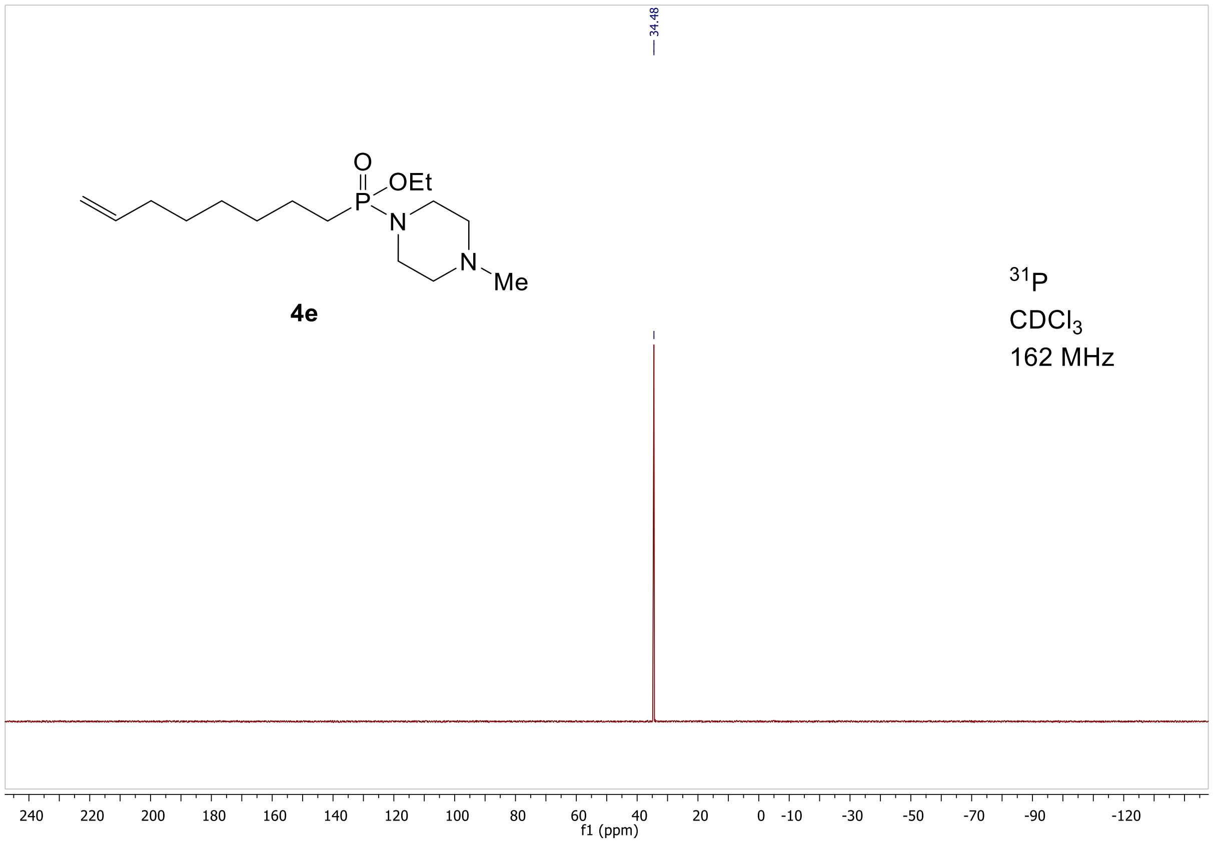
34.48
OEt
N
N
Me
O
P
4e
^{31}P
CDCl$_3$
162 MHz
240 220 200 180 160 140 120 100 80 60 40 20 0 -10 -30 -50 -70 -90 -120
f1 (ppm)

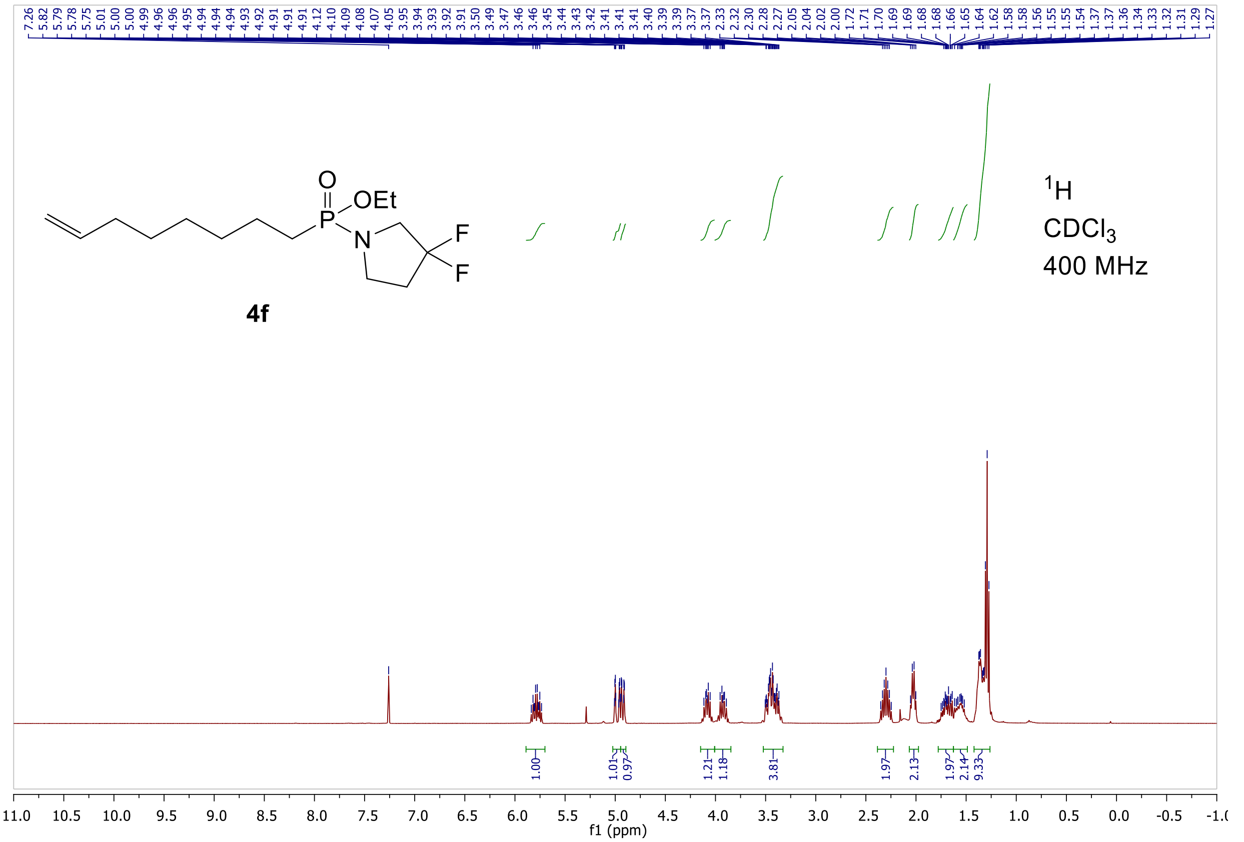

OEt
N
F
F
4f
1H
CDCl3
400 MHz
f1 (ppm)

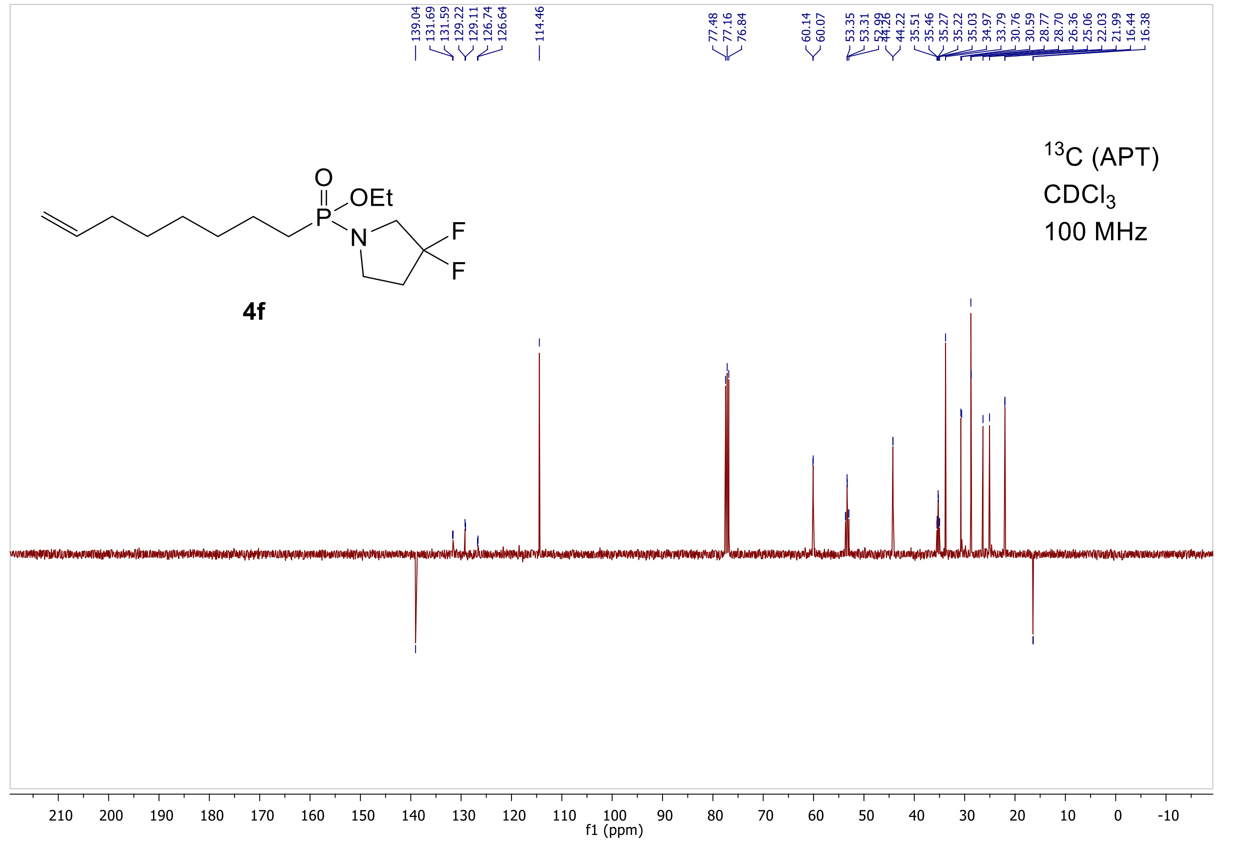

OEt
N
F
F
4f
13C (APT)
CDCl3
100 MHz
f1 (ppm)

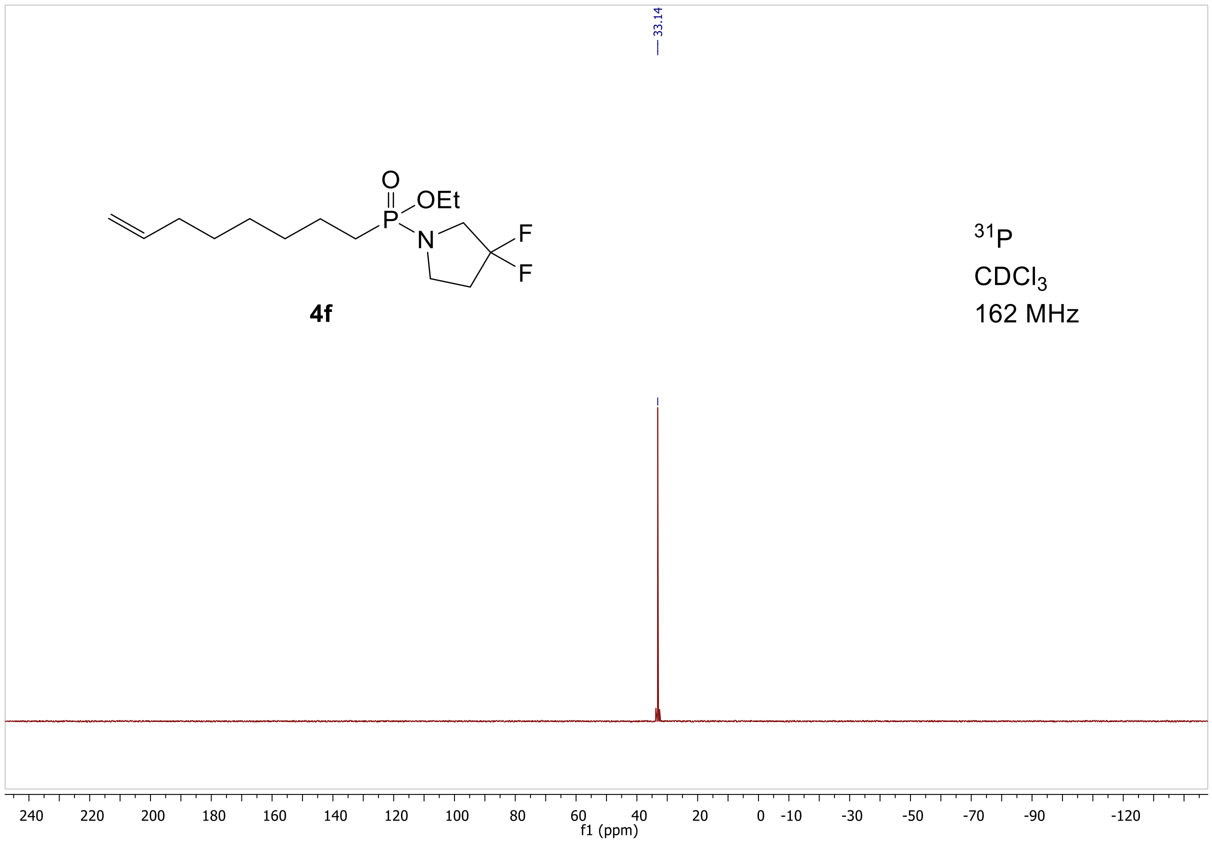
33.14
OEt
N
F
F
4f
31P
CDCl3
162 MHz
f1 (ppm)

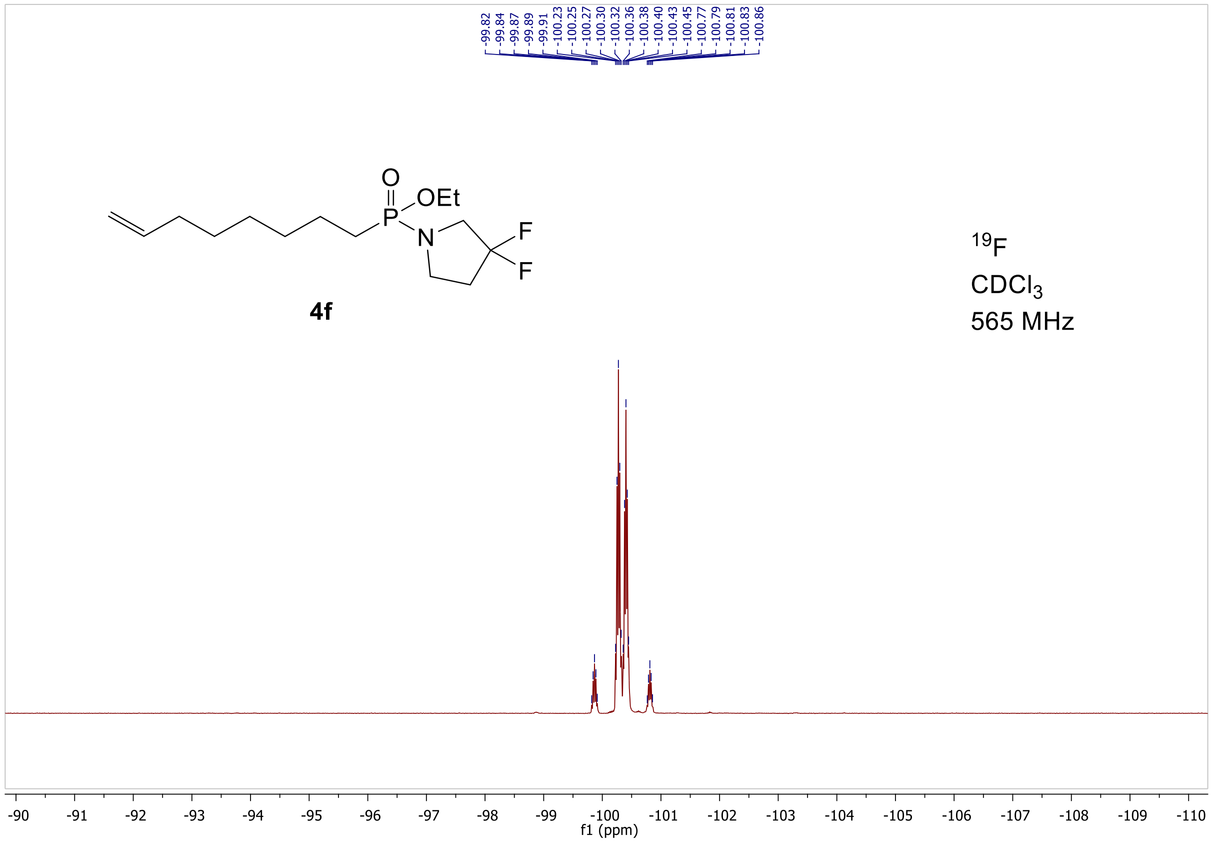
-99.82
-99.84
-99.87
-99.89
-99.91
-100.23
-100.25
-100.27
-100.30
-100.32
-100.36
-100.38
-100.40
-100.43
-100.45
-100.77
-100.79
-100.81
-100.83
-100.86
OEt
N
F
F
4f
19F
CDCl3
565 MHz
f1 (ppm)

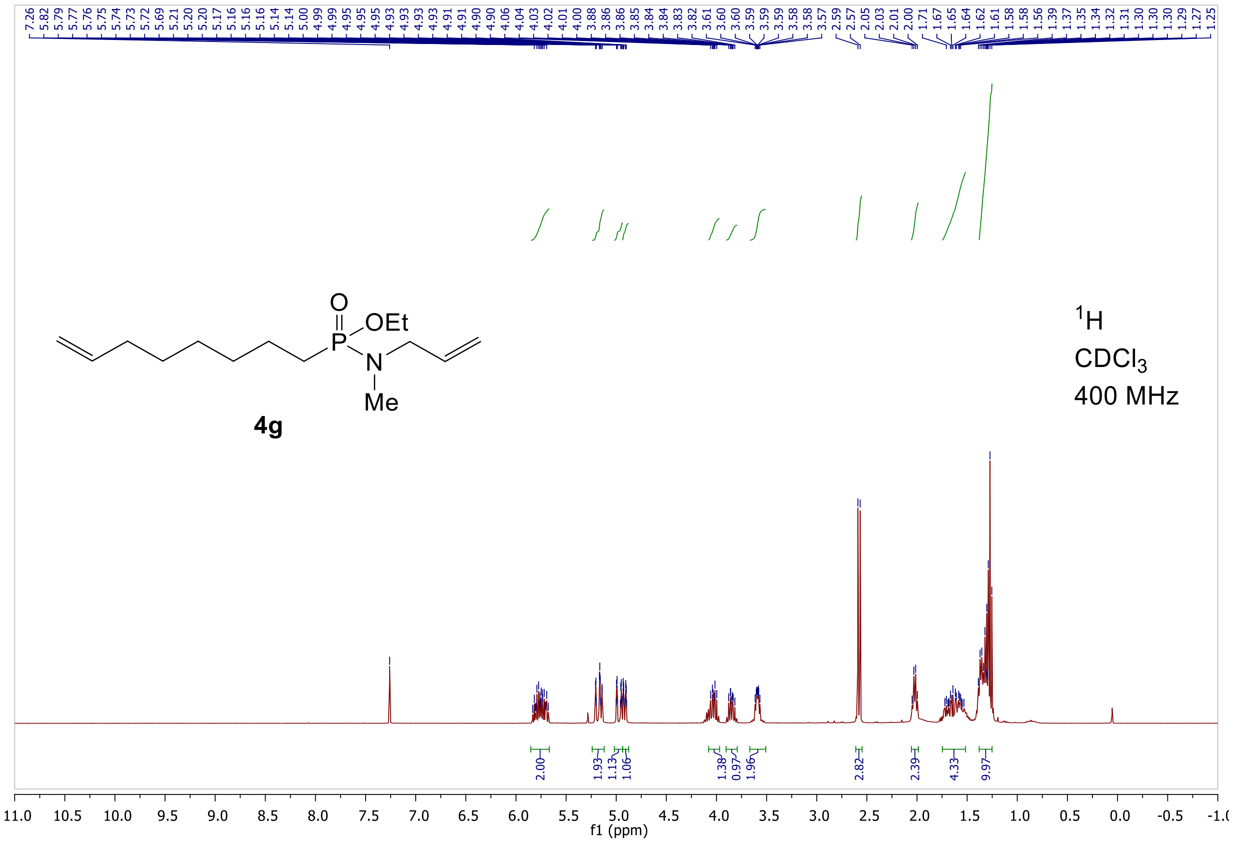
OEt
N
Me
4g
1H
CDCl3
400 MHz
f1 (ppm)

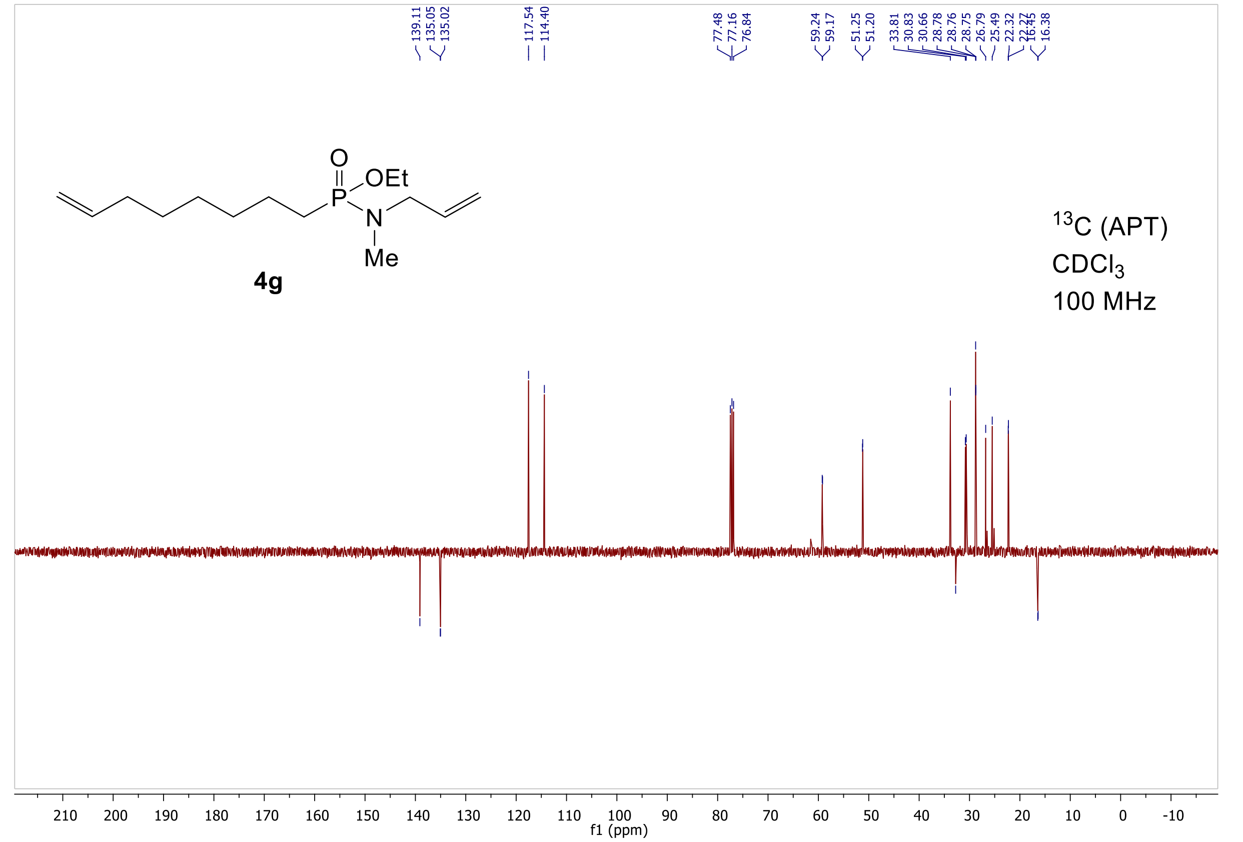
OEt
N
Me
4g
13C (APT)
CDCl3
100 MHz
f1 (ppm)

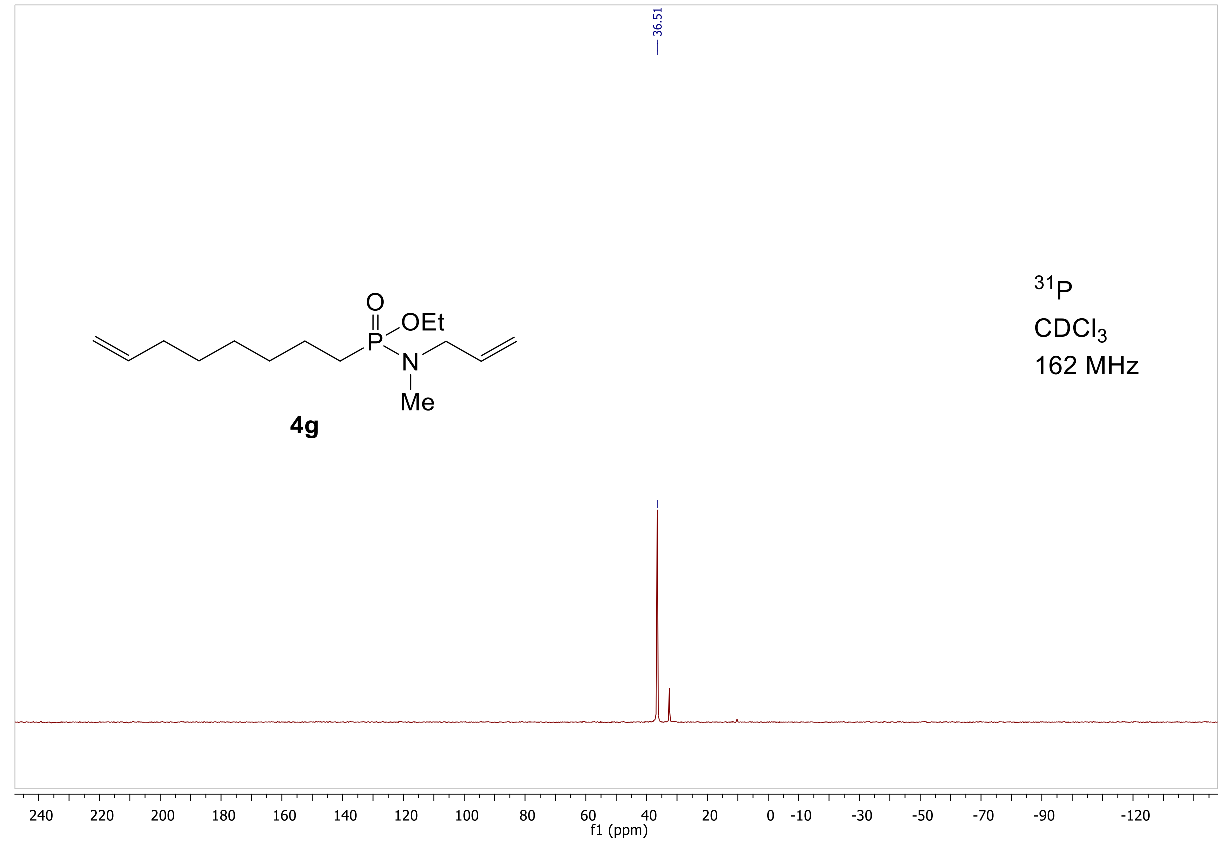

36.51
O
OEt
P
N
Me
4g
^{31}P
CDCl$_3$
162 MHz
240 220 200 180 160 140 120 100 80 60 40 20 0 -10 -30 -50 -70 -90 -120
f1 (ppm)

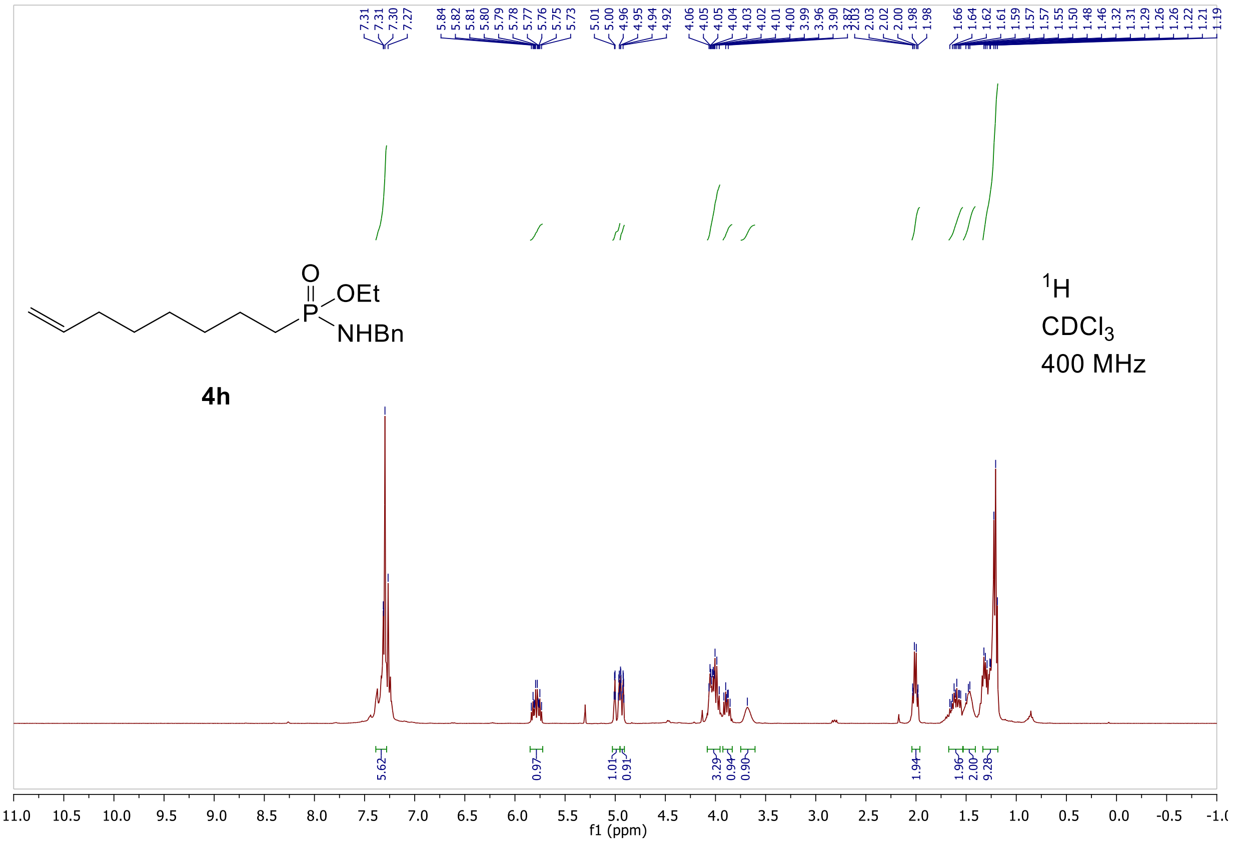
4h
OEt
NHBn
^{1}H
$CDCl_3$
400 MHz
f1 (ppm)

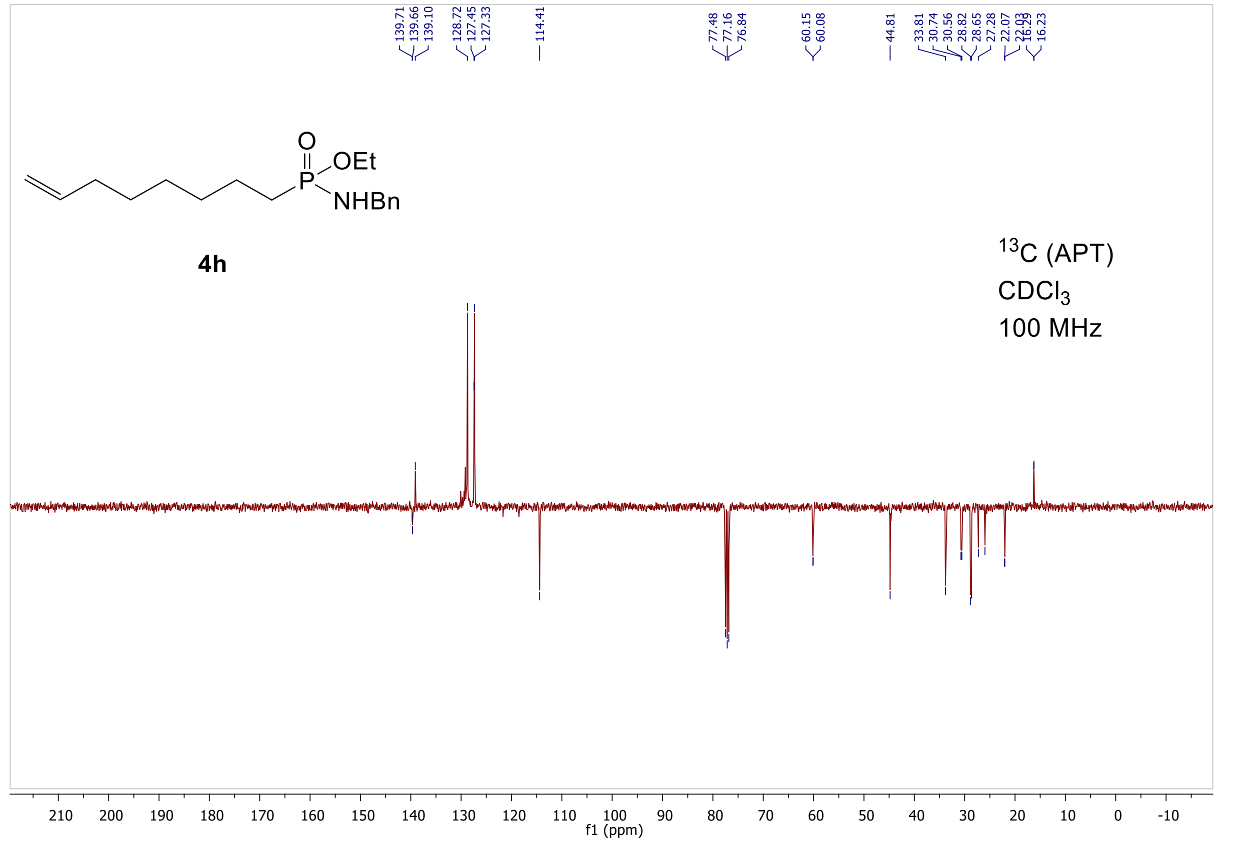
4h
OEt
NHBn
^{13}C (APT)
$CDCl_3$
100 MHz
f1 (ppm)

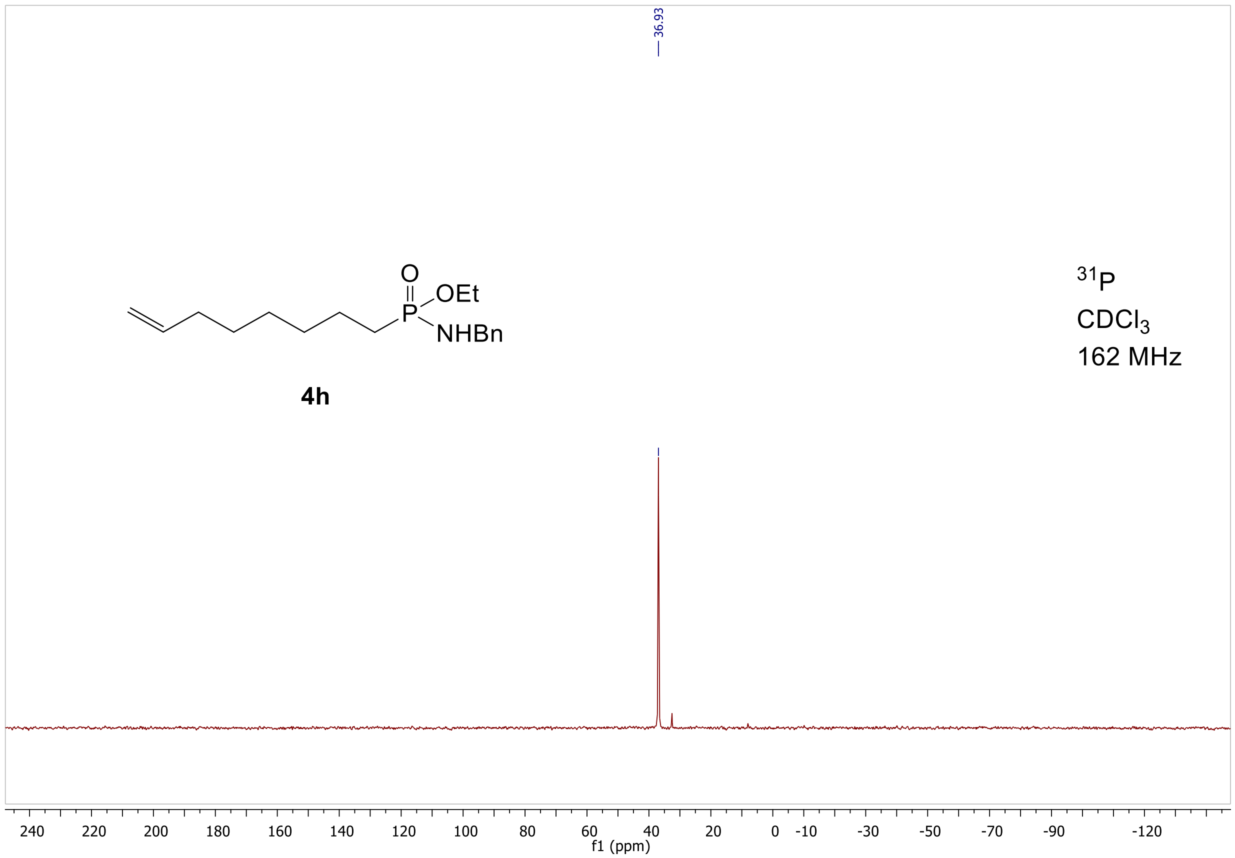
36.93
O
OEt
P
NHBn
4h
31P
CDCl3
162 MHz
240 220 200 180 160 140 120 100 80 60 40 20 0 -10 -30 -50 -70 -90 -120
f1 (ppm)

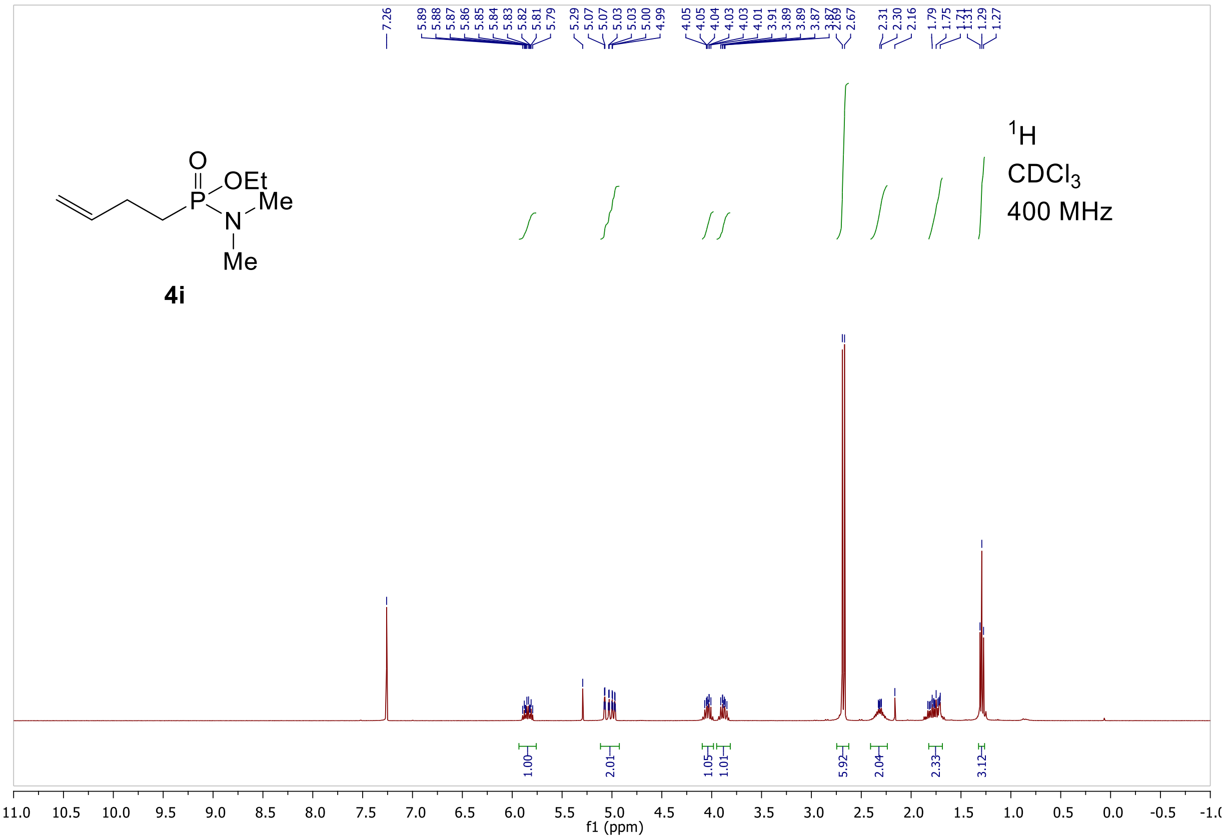
O
OEt
P
N
Me
Me
4i
1H
CDCl3
400 MHz
7.26
5.89 5.88 5.87 5.86 5.85 5.84 5.83 5.82 5.81 5.79
5.29 5.07 5.07 5.03 5.03 5.00 4.99
4.05 4.05 4.04 4.03 4.03 4.01 3.91 3.89 3.89 3.87
2.69 2.67
2.31 2.30 2.16
1.79 1.75 1.71 1.31 1.29 1.27
1.00 2.01 1.05 1.01 5.92 2.04 2.33 3.12
f1 (ppm)

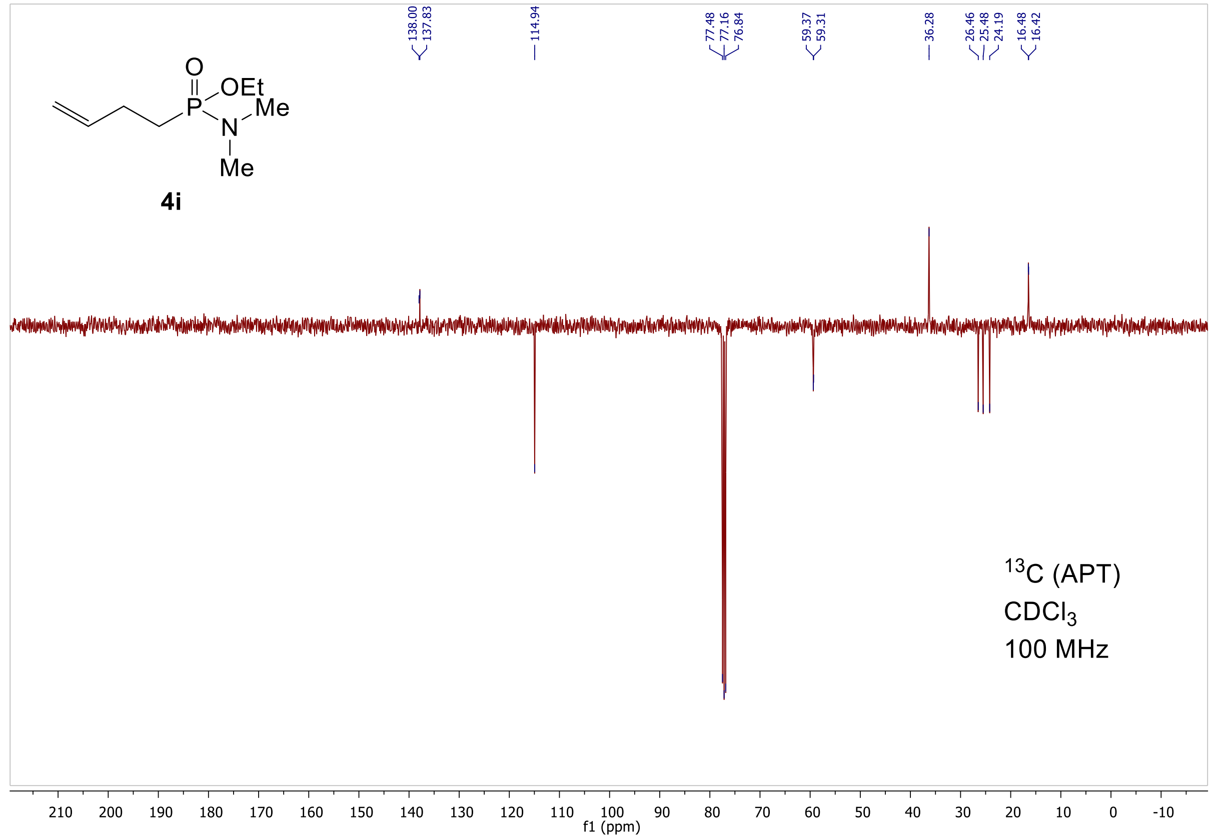
O
OEt
P
N
Me
Me
4i
13C (APT)
CDCl3
100 MHz
138.00 137.83
114.94
77.48 77.16 76.84
59.37 59.31
36.28
26.46 25.48 24.19
16.48 16.42
f1 (ppm)

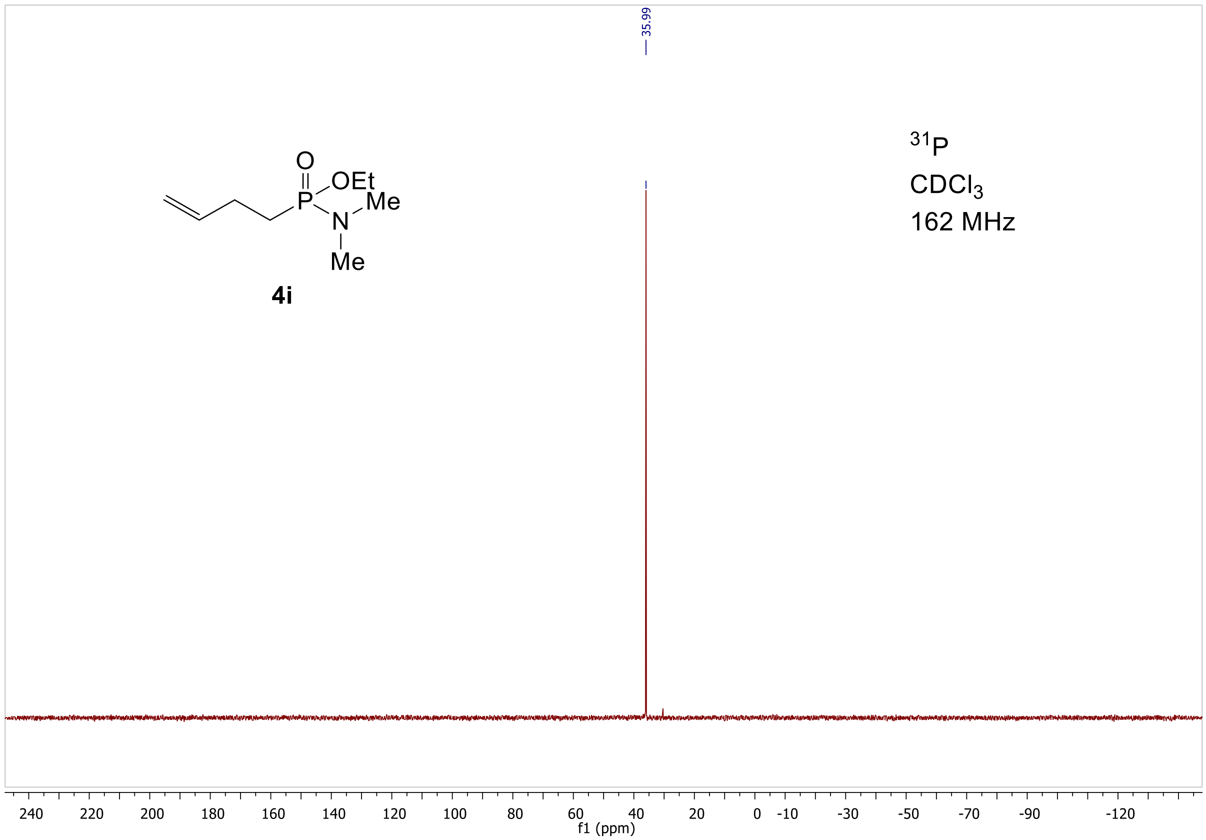
35.99
O
OEt
P
N
Me
Me
4i
31P
CDCl3
162 MHz
240
220
200
180
160
140
120
100
80
60
40
20
0
-10
-30
-50
-70
-90
-120
f1 (ppm)

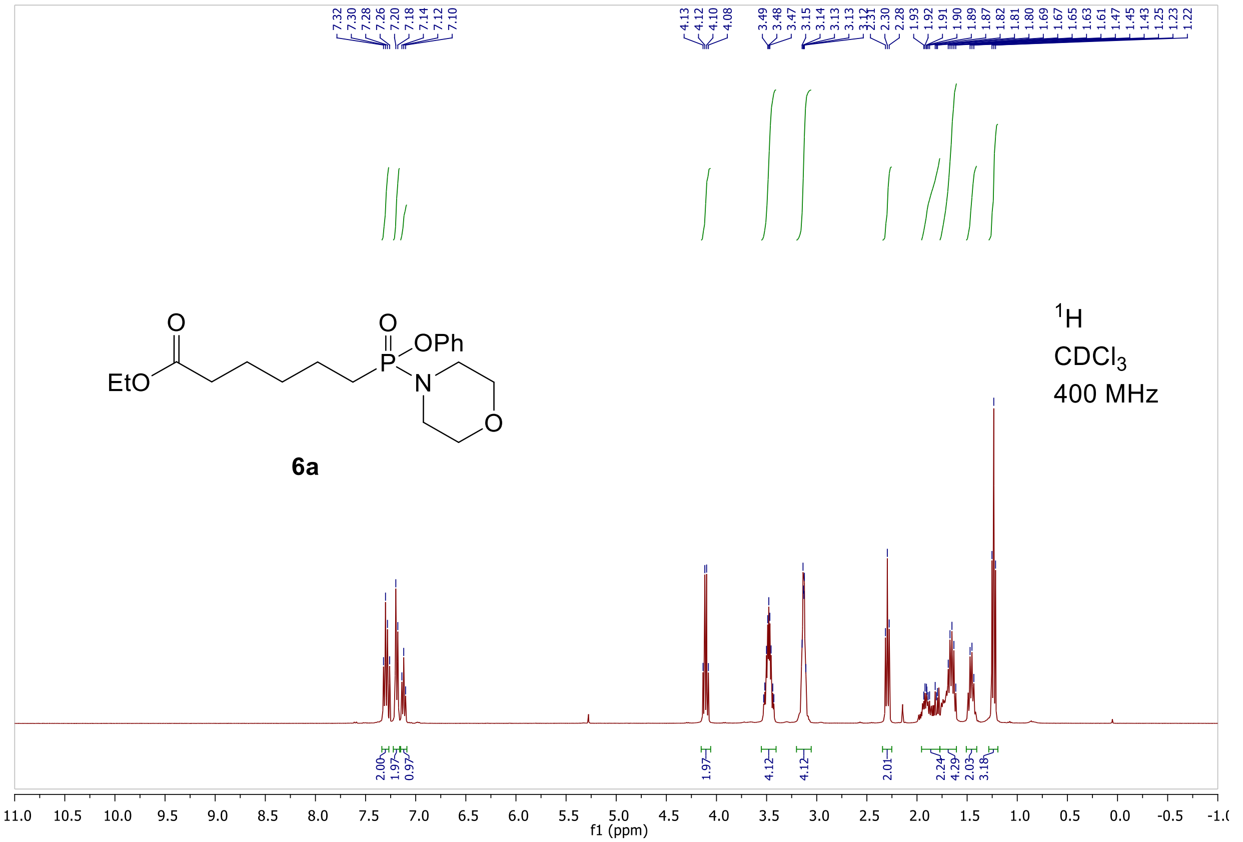
7.32 7.30 7.28 7.26 7.20 7.18 7.14 7.12 7.10
4.13 4.12 4.10 4.08
3.49 3.48 3.47 3.15 3.14 3.13 3.13 3.12 2.31 2.30 2.28
1.93 1.92 1.91 1.90 1.89 1.87 1.82 1.81 1.80 1.69 1.67 1.65 1.63 1.61 1.47 1.45 1.43 1.25 1.23 1.22
EtO
O
P
O
OPh
N
O
6a
^{1}H
$CDCl_3$
400 MHz
2.00 1.97 0.97 1.97 4.12 4.12 2.01 2.24 4.29 2.03 3.18
11.0 10.5 10.0 9.5 9.0 8.5 8.0 7.5 7.0 6.5 6.0 5.5 5.0 4.5 4.0 3.5 3.0 2.5 2.0 1.5 1.0 0.5 0.0 -0.5
f1 (ppm)

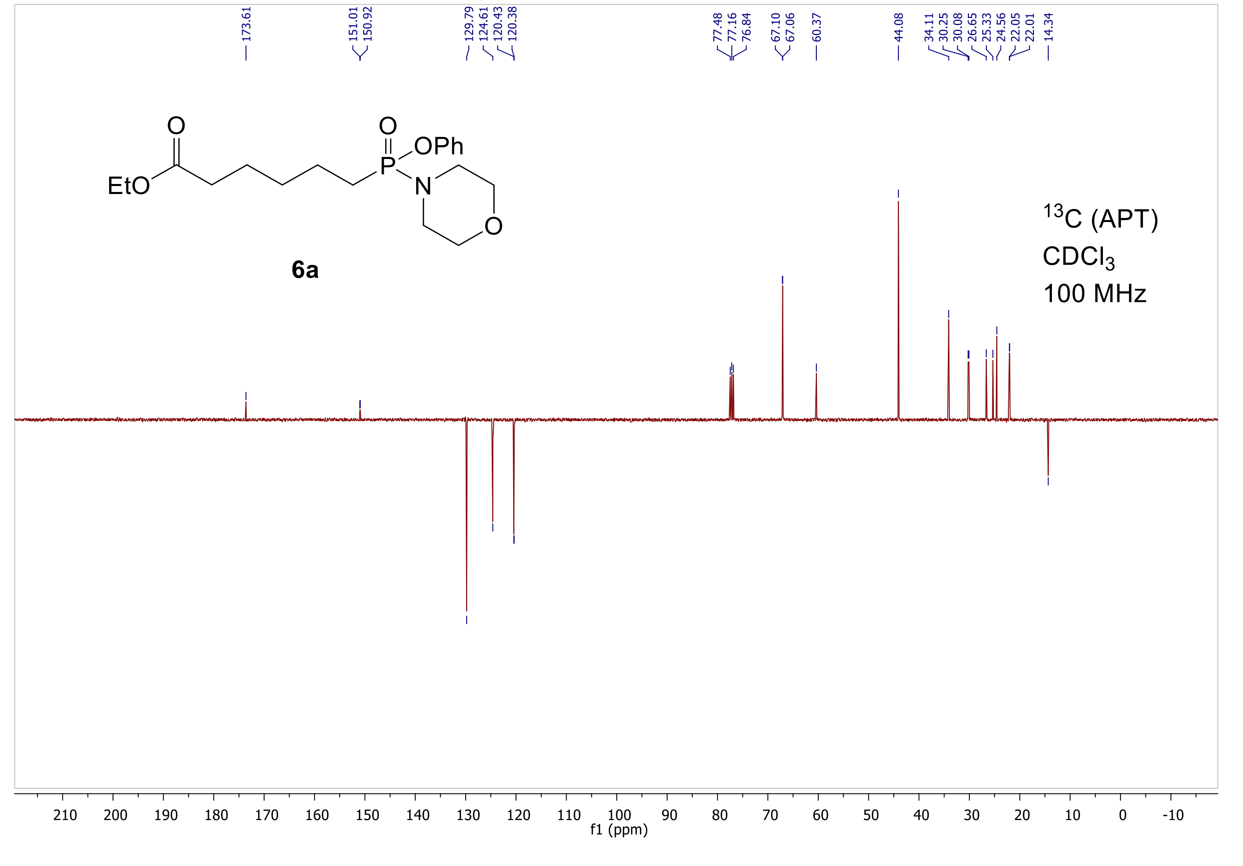
173.61
151.01 150.92
129.79 124.61 120.43 120.38
77.48 77.16 76.84 67.10 67.06 60.37
44.08
34.11 30.25 30.08 26.65 25.33 24.56 22.05 22.01 14.34
EtO
O
P
O
OPh
N
O
6a
^{13}C (APT)
$CDCl_3$
100 MHz
210 200 190 180 170 160 150 140 130 120 110 100 90 80 70 60 50 40 30 20 10 0 -10
f1 (ppm)

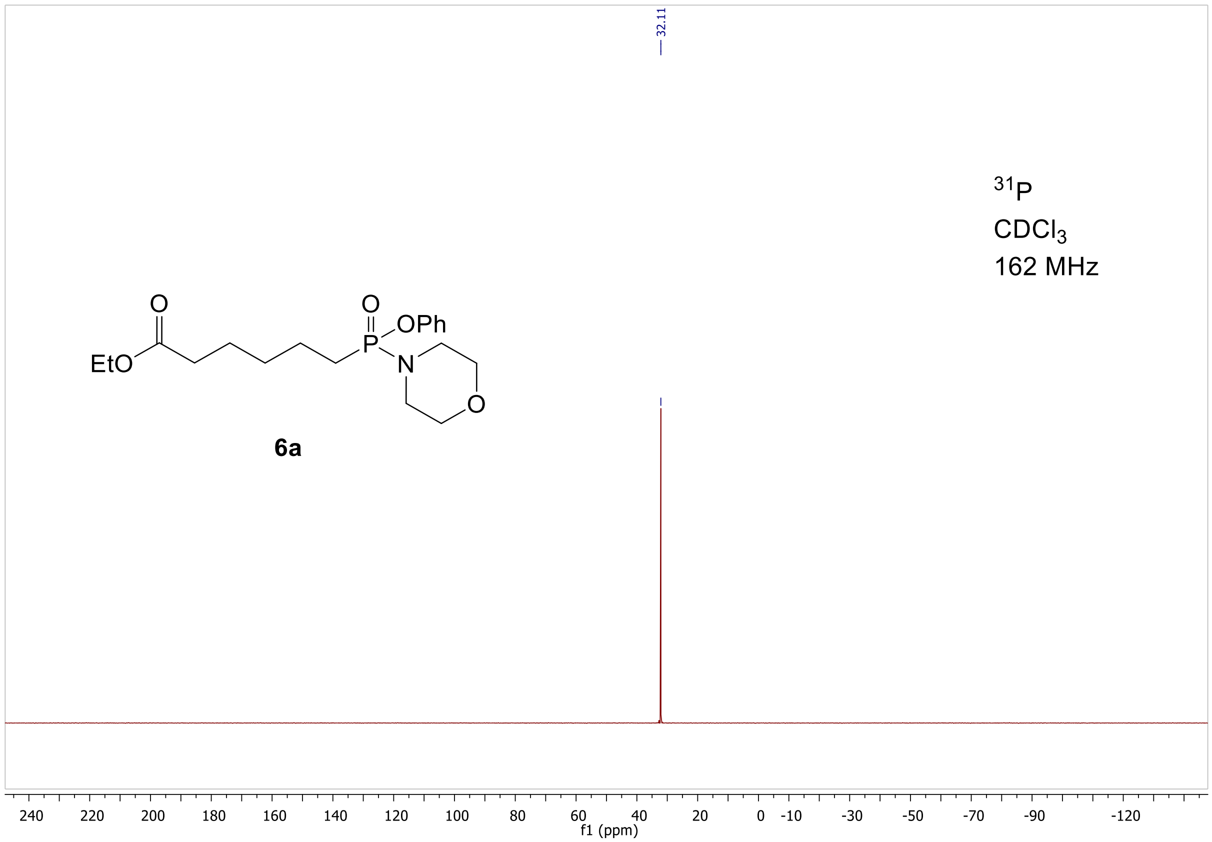

32.11
31P
CDCl3
162 MHz
O
O
OPh
EtO
P
N
O
6a
240
220
200
180
160
140
120
100
80
60
40
20
0
-10
-30
-50
-70
-90
-120
f1 (ppm)

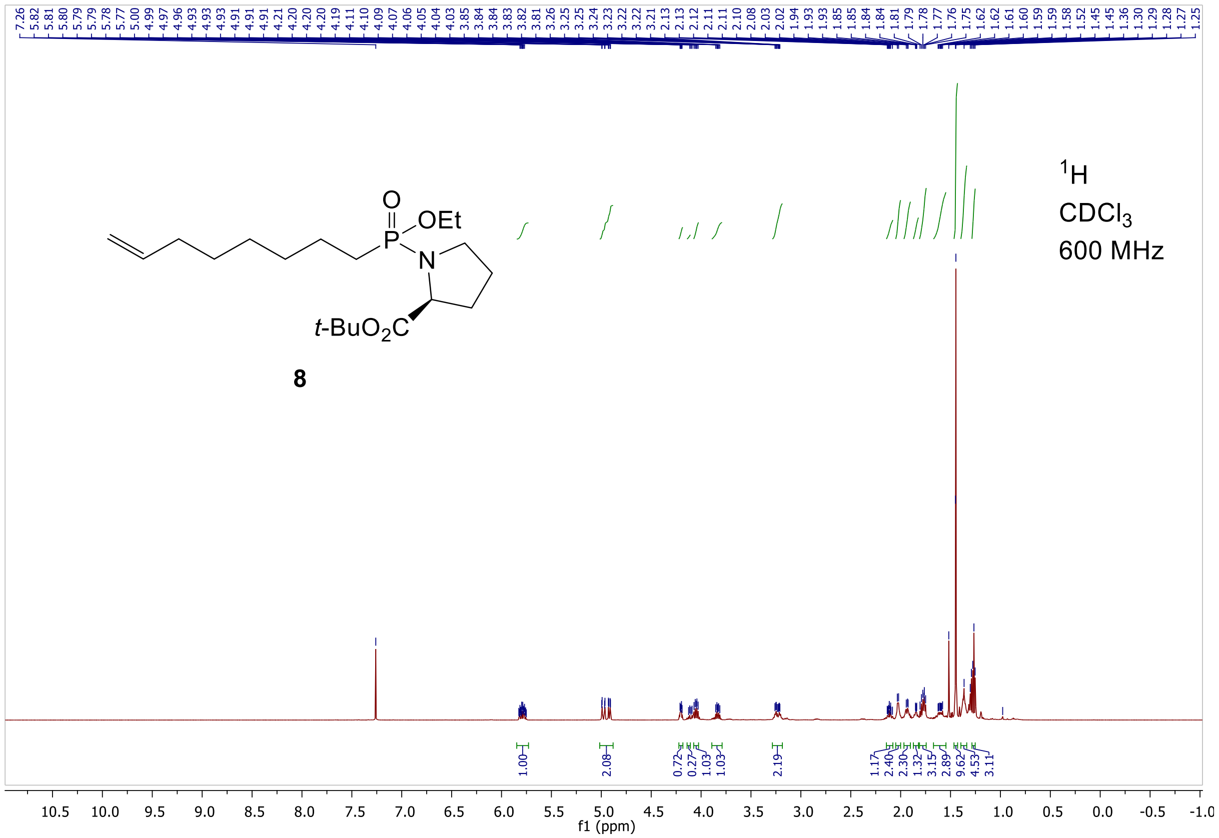

8
^{1}H
$CDCl_3$
600 MHz
f1 (ppm)

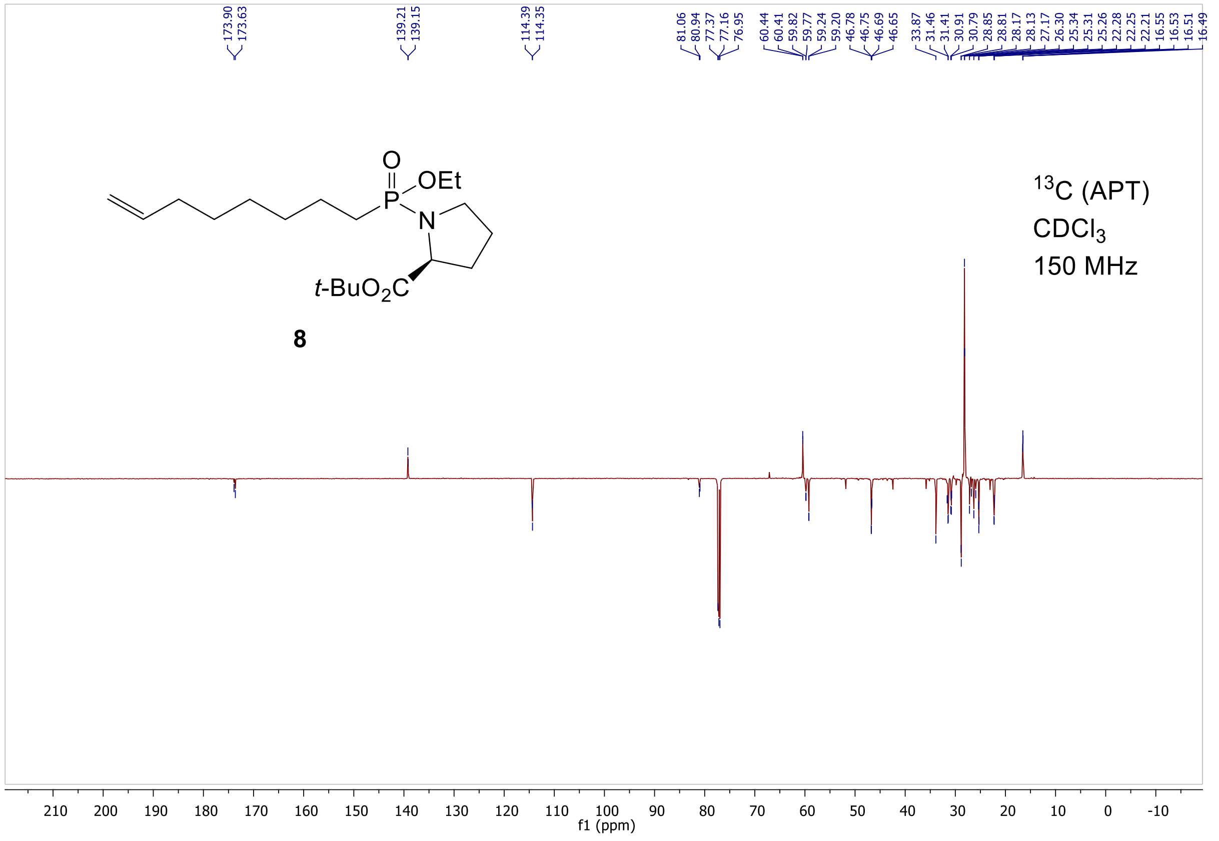

8
^{13}C (APT)
$CDCl_3$
150 MHz
f1 (ppm)

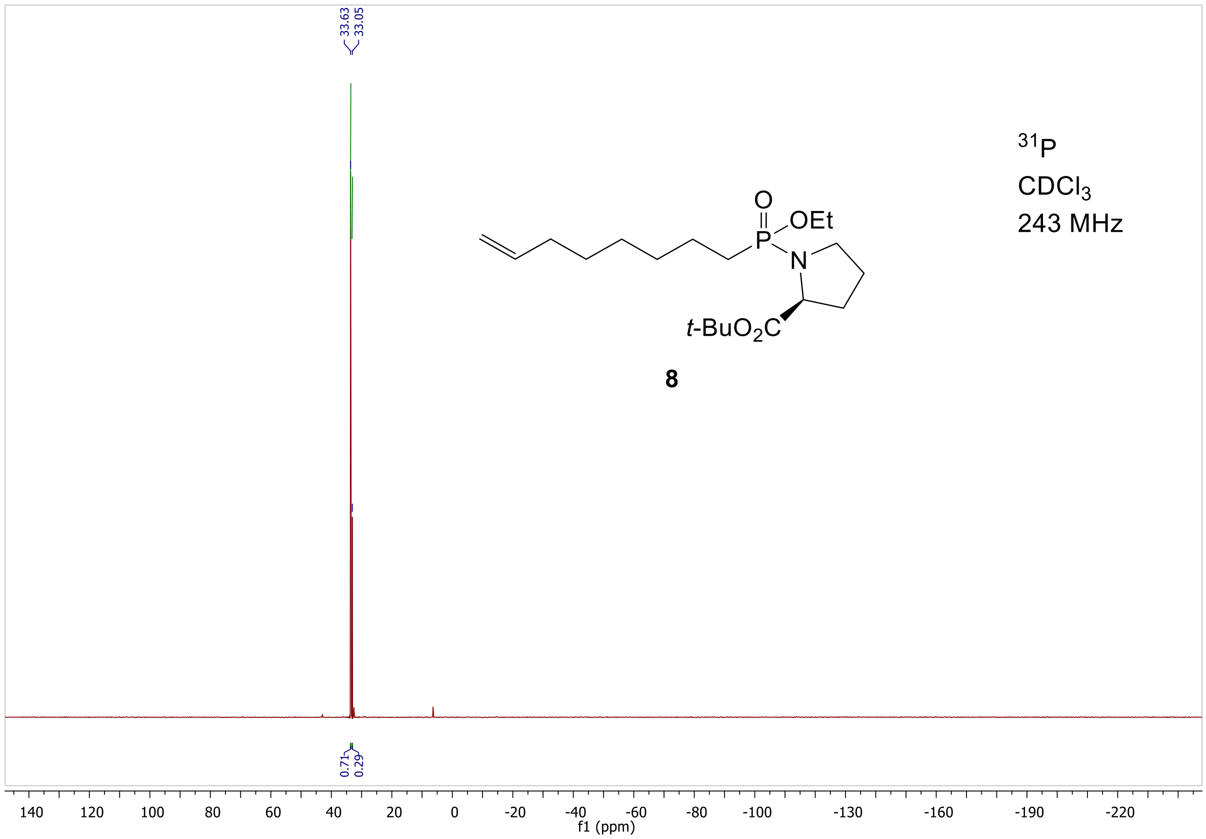

33.63
33.05
^{31}P
$CDCl_3$
243 MHz
O
OEt
P
N
t-BuO2C
8
0.71
0.29
140
120
100
80
60
40
20
0
-20
-40
-60
-80
-100
-130
-160
-190
-220
f1 (ppm)

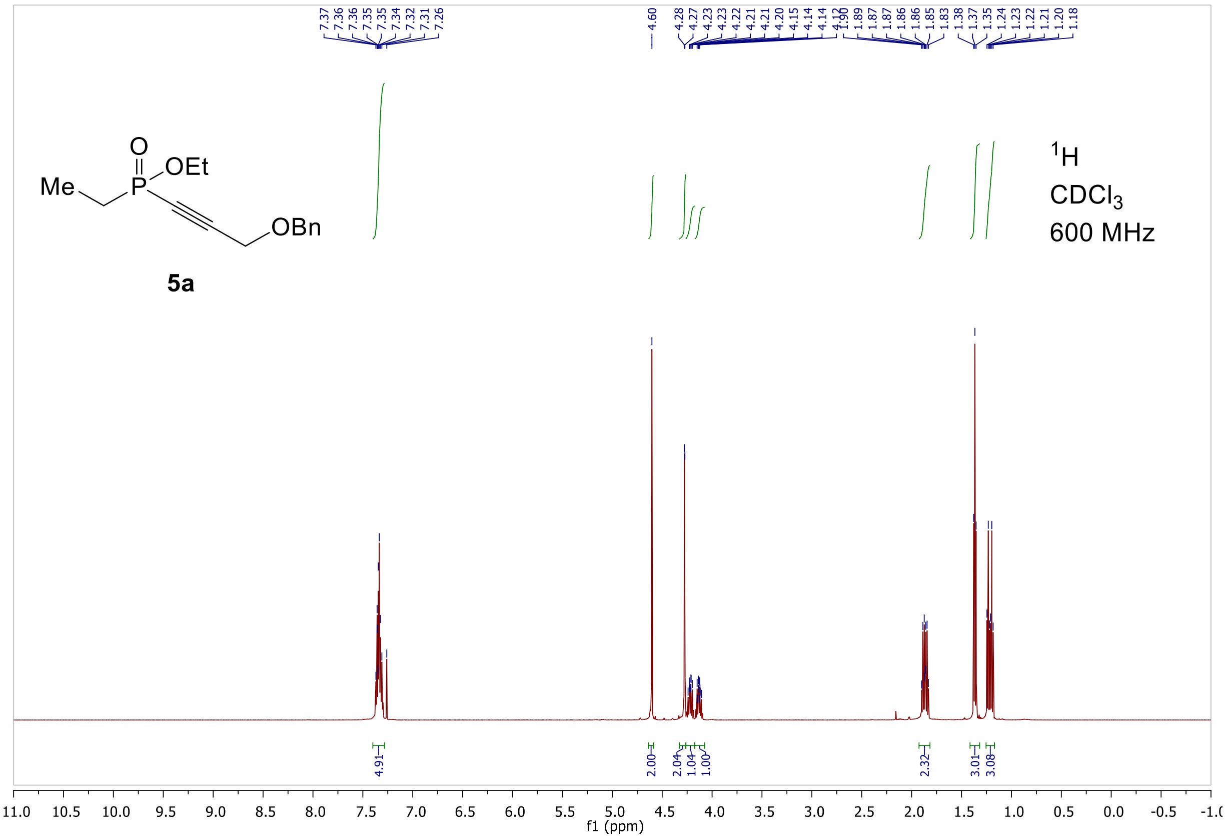
7.37 7.36 7.36 7.35 7.35 7.34 7.32 7.31 7.26
4.60
4.28 4.27 4.23 4.23 4.22 4.21 4.21 4.20 4.15 4.14 4.14 4.12
1.90 1.89 1.87 1.87 1.86 1.85 1.83
1.38 1.37 1.35 1.24 1.23 1.22 1.21 1.20 1.18
O
OEt
Me
P
OBn
5a
^{1}H
$CDCl_3$
600 MHz
4.91
2.00
2.04
1.04
1.00
2.32
3.01
3.08
f1 (ppm)

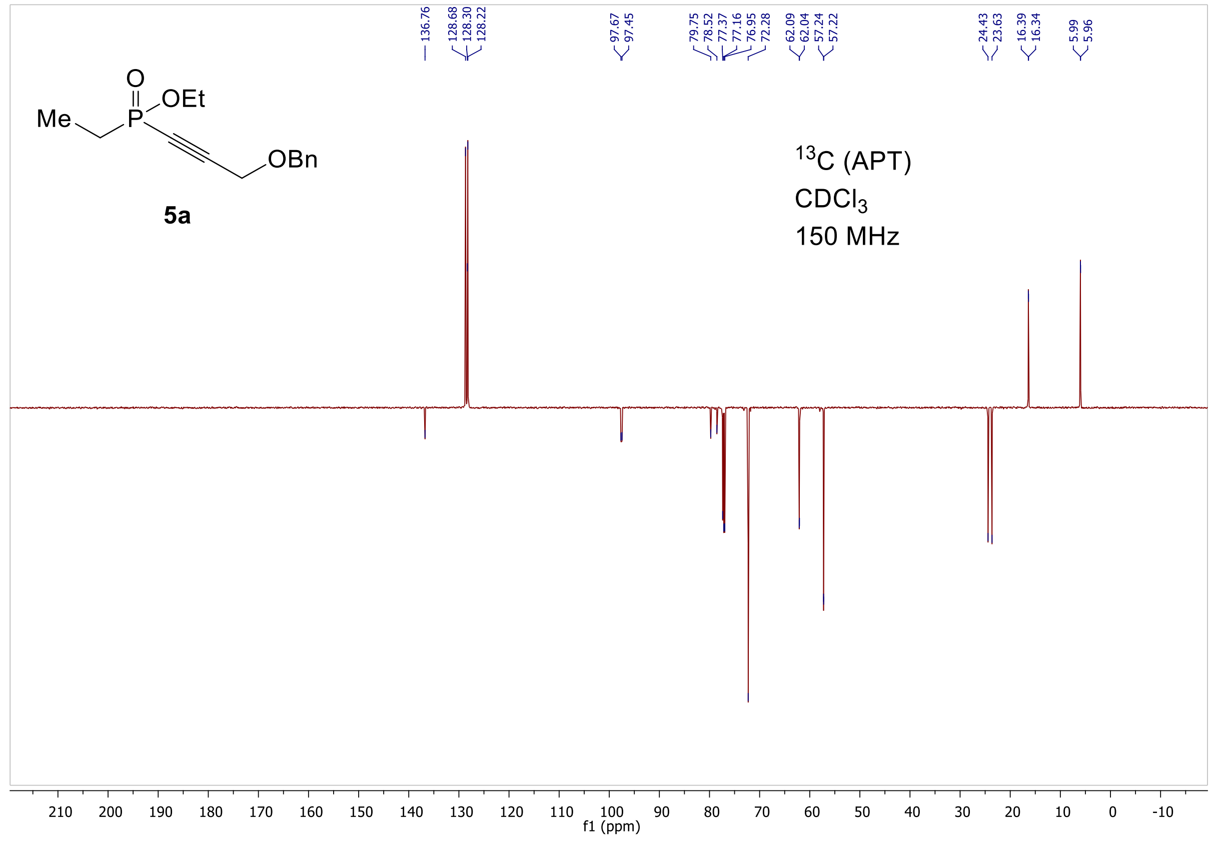
136.76
128.68 128.30 128.22
97.67 97.45
79.75 78.52 77.37 77.16 76.95 72.28
62.09 62.04 57.24 57.22
24.43 23.63
16.39 16.34
5.99 5.96
O
OEt
Me
P
OBn
5a
^{13}C (APT)
$CDCl_3$
150 MHz
f1 (ppm)

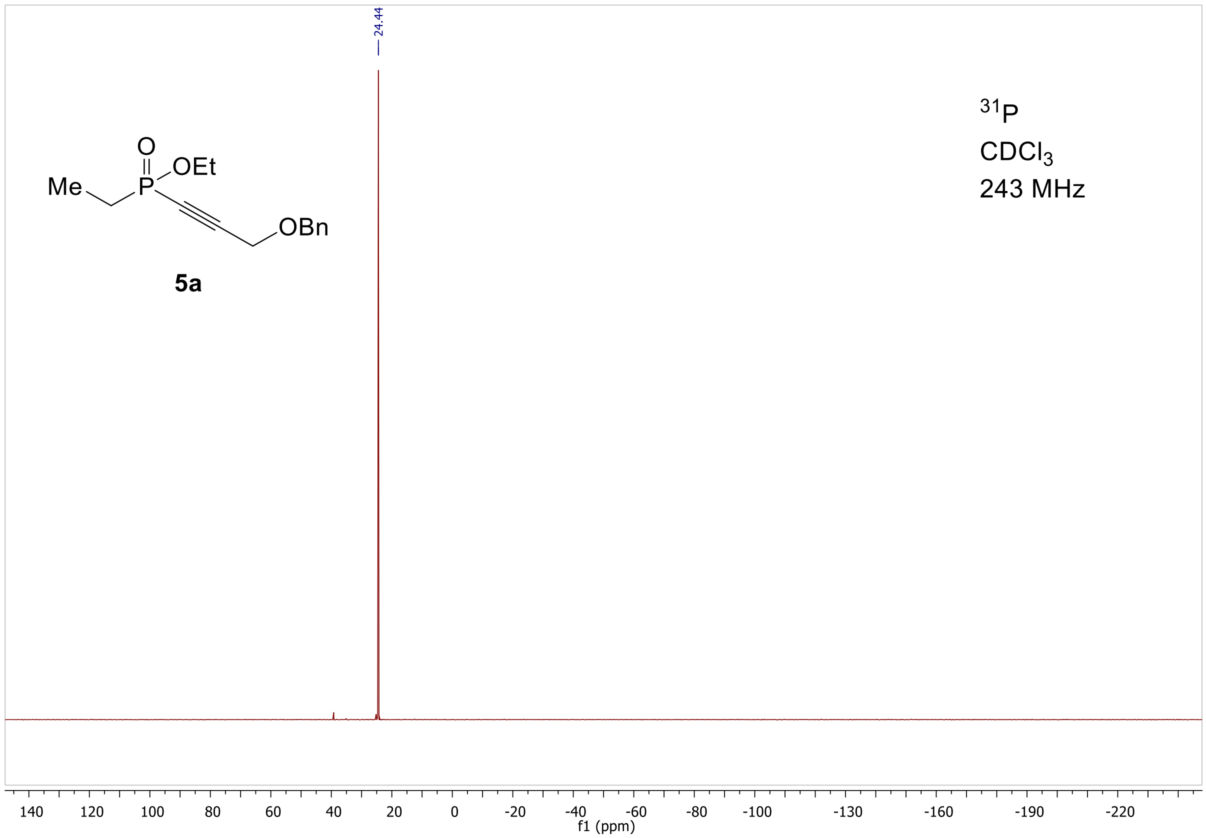

24.44
O
OEt
Me
P
OBn
5a
^{31}P
CDCl$_3$
243 MHz
140 120 100 80 60 40 20 0 -20 -40 -60 -80 -100 -130 -160 -190 -220
f1 (ppm)

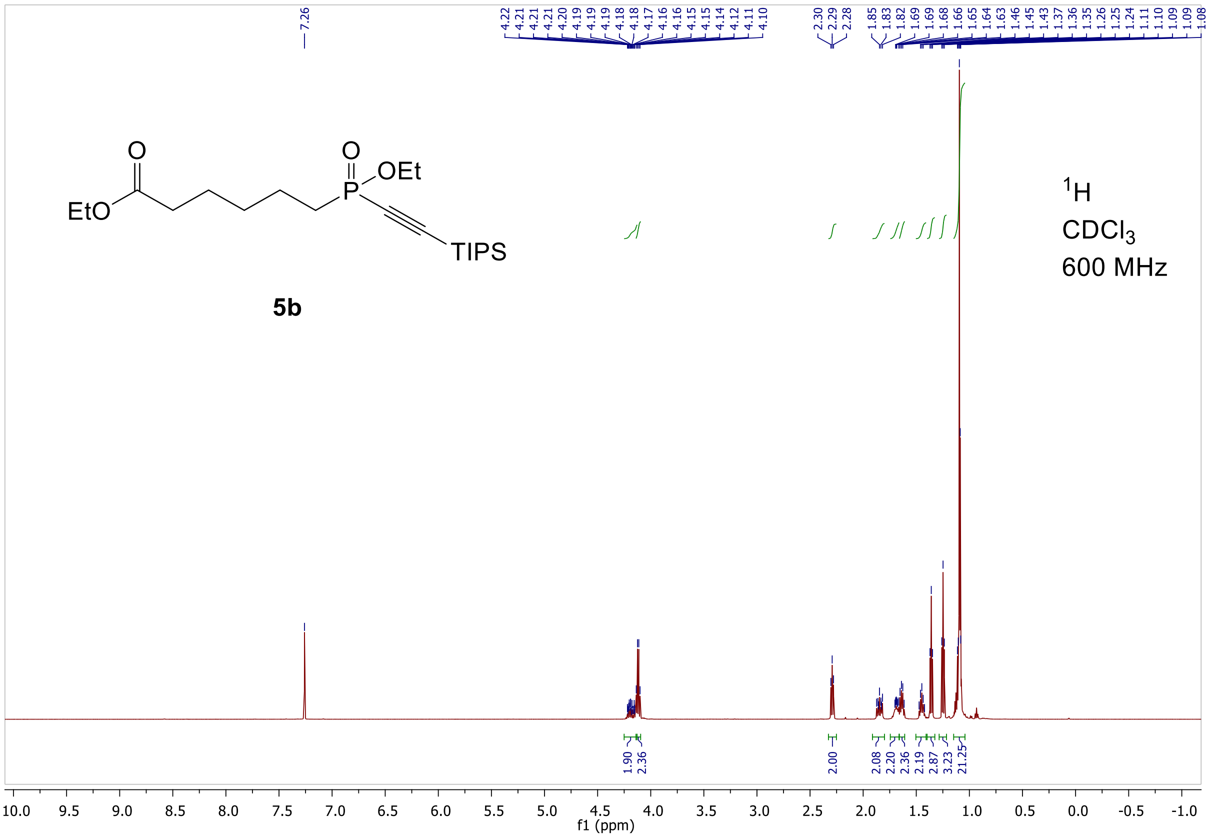

EtO
O
P
O
OEt
TIPS
5b
1H
CDCl3
600 MHz
f1 (ppm)

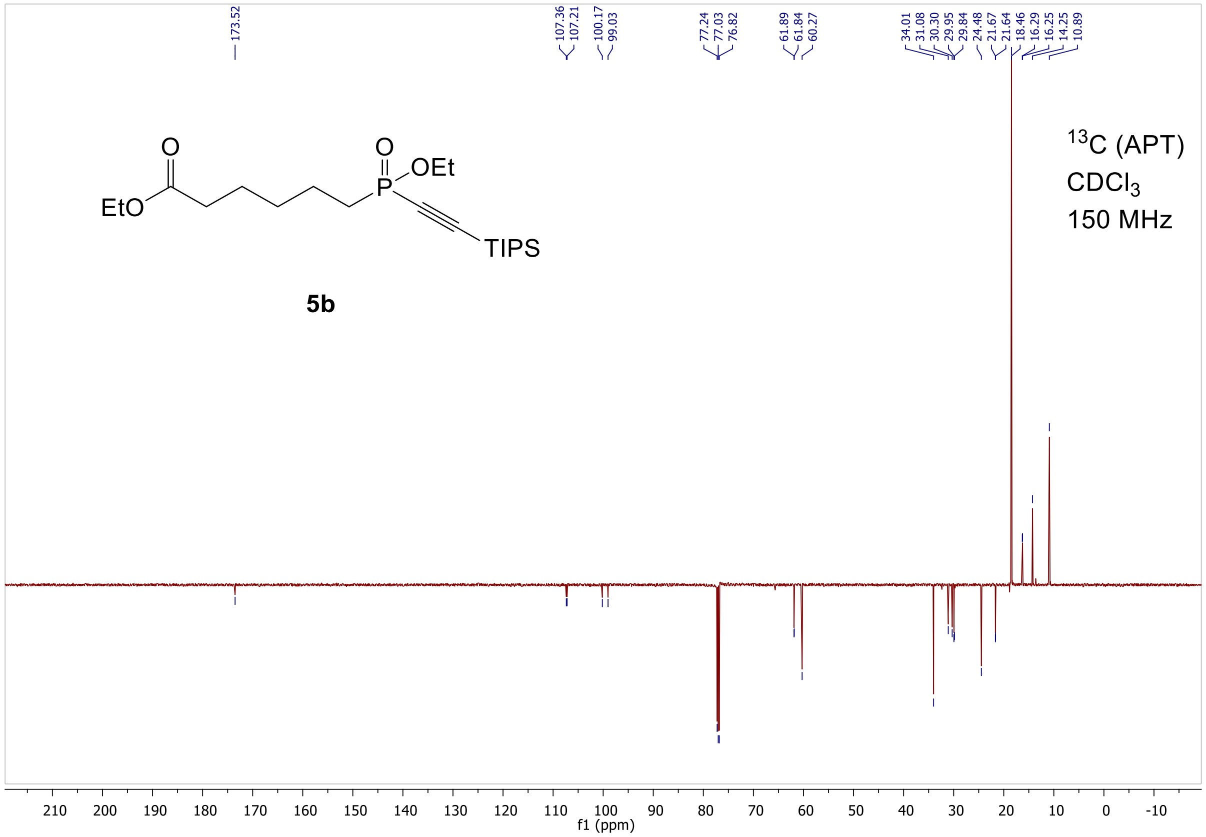

EtO
O
P
O
OEt
TIPS
5b
13C (APT)
CDCl3
150 MHz
f1 (ppm)

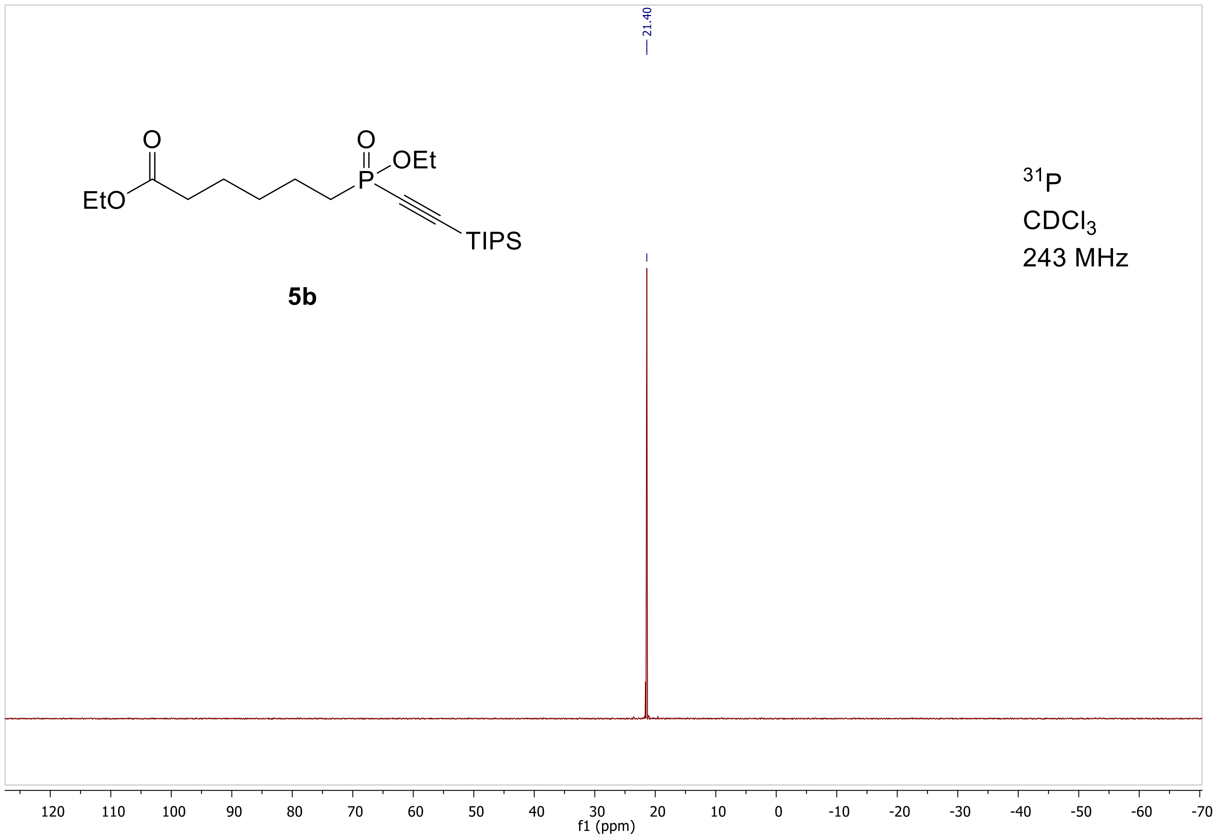
21.40
O
O
OEt
EtO
P
TIPS
5b
^{31}P
CDCl$_3$
243 MHz
120 110 100 90 80 70 60 50 40 30 20 10 0 -10 -20 -30 -40 -50 -60 -70
f1 (ppm)

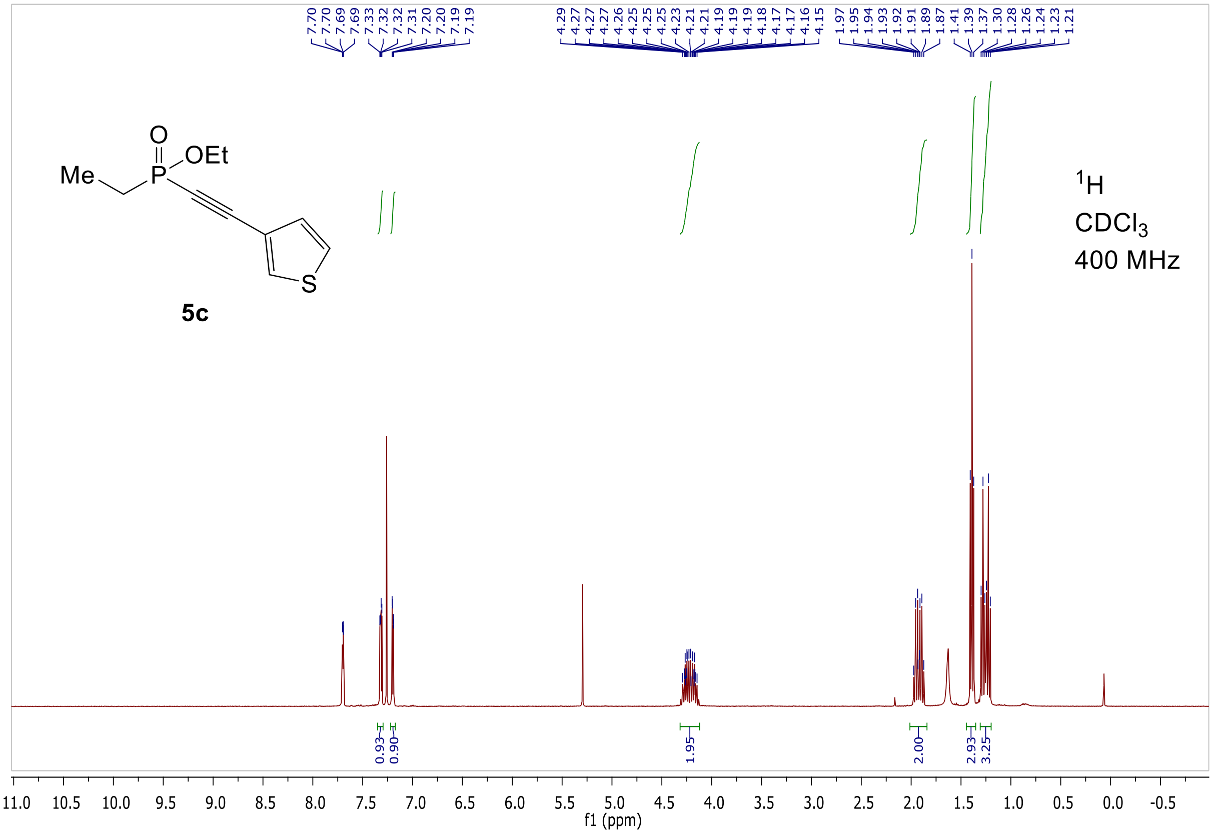
7.70 7.70 7.69 7.69 7.33 7.32 7.32 7.31 7.20 7.20 7.19 7.19
4.29 4.27 4.27 4.26 4.25 4.25 4.25 4.23 4.21 4.21 4.19 4.19 4.19 4.18 4.17 4.17 4.16 4.15
1.97 1.95 1.94 1.93 1.92 1.91 1.89 1.87 1.41 1.39 1.37 1.30 1.28 1.26 1.24 1.23 1.21
O
OEt
Me
P
S
5c
^{1}H
$CDCl_3$
400 MHz
0.93 0.90 1.95 2.00 2.93 3.25
f1 (ppm)

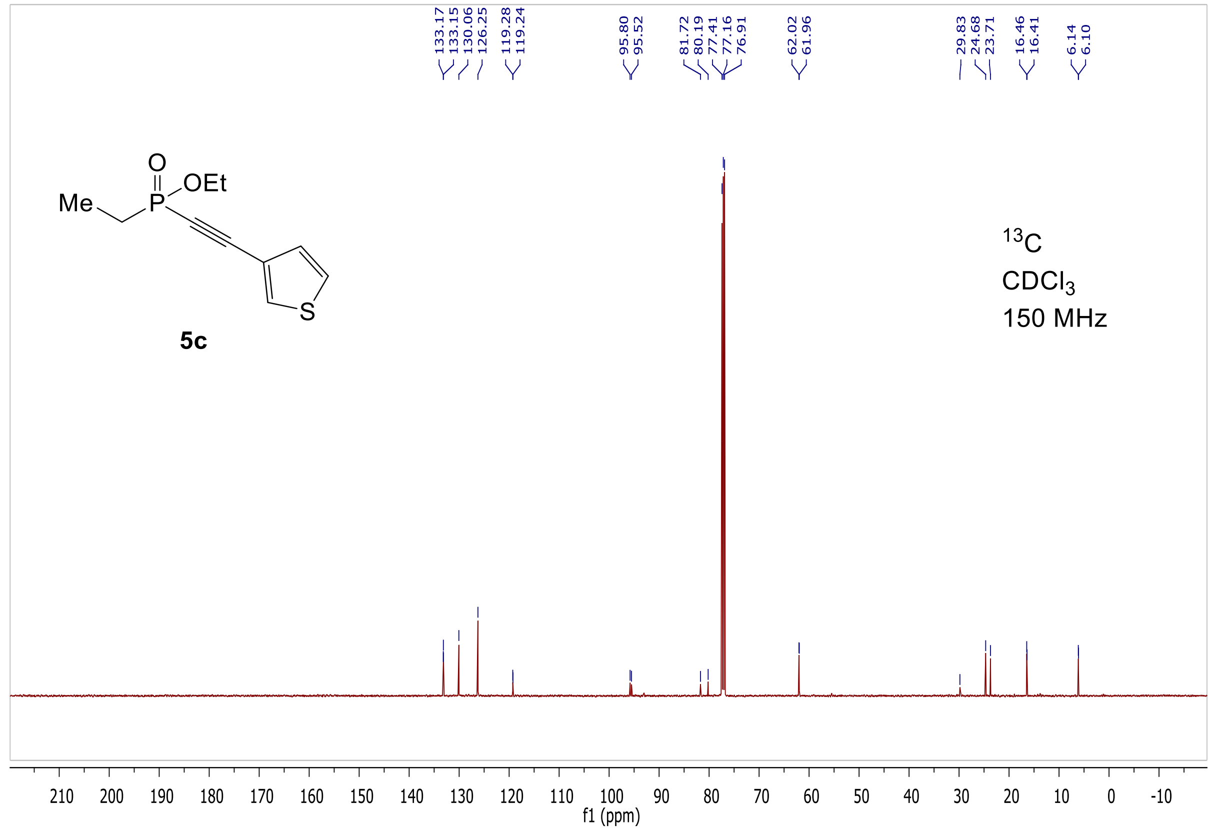
133.17 133.15 130.06 126.25 119.28 119.24
95.80 95.52
81.72 80.19 77.41 77.16 76.91
62.02 61.96
29.83 24.68 23.71
16.46 16.41
6.14 6.10
O
OEt
Me
P
S
5c
^{13}C
$CDCl_3$
150 MHz
f1 (ppm)

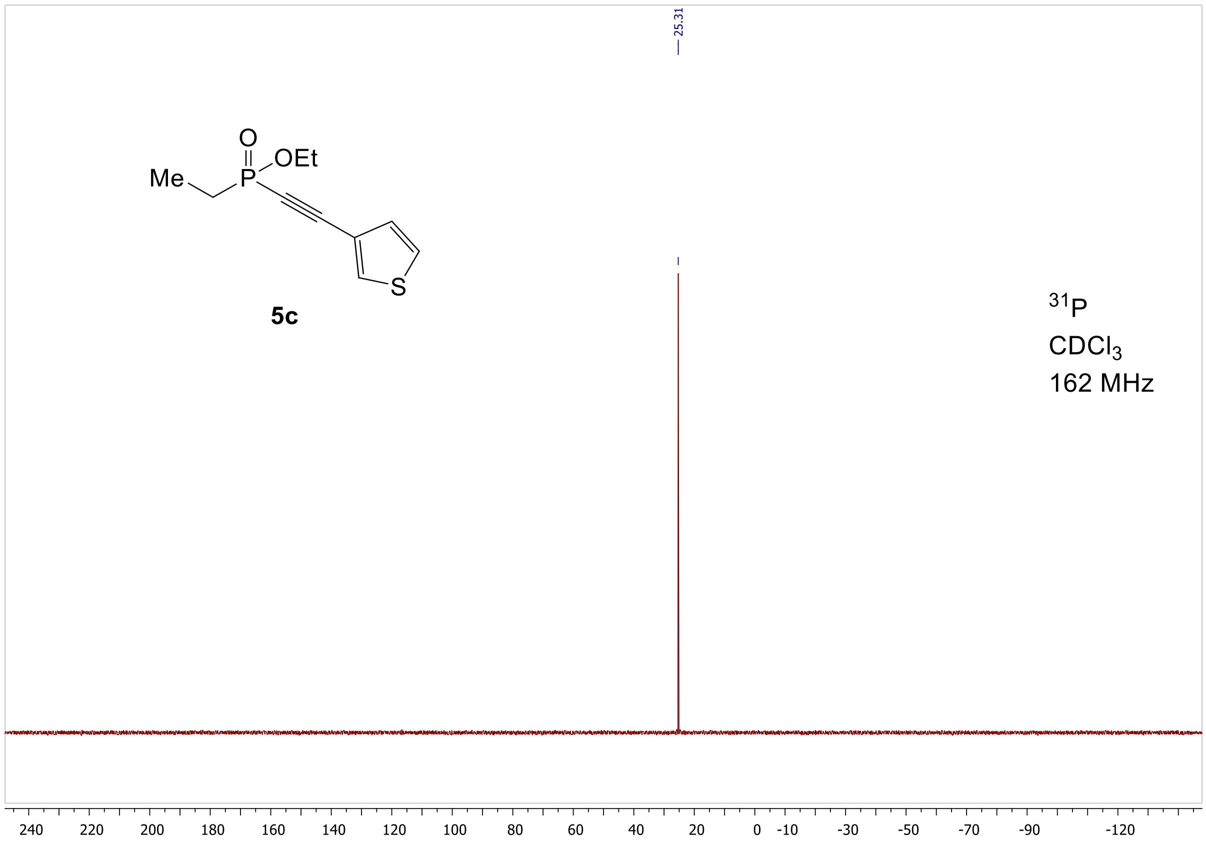
25.31
Me
O
OEt
P
S
5c
^{31}P
$CDCl_3$
162 MHz
240
220
200
180
160
140
120
100
80
60
40
20
0
-10
-30
-50
-70
-90
-120

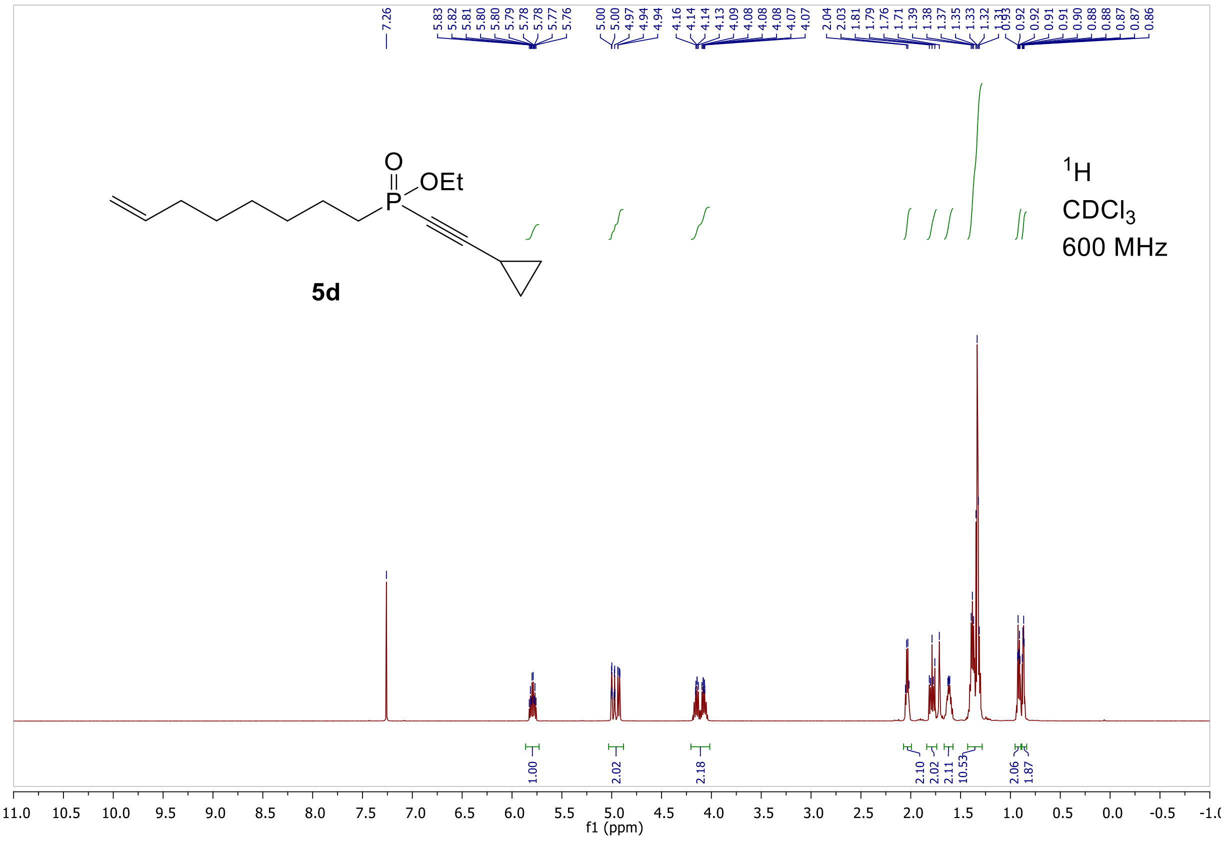

O
OEt
P
5d
^{1}H
CDCl$_3$
600 MHz
f1 (ppm)

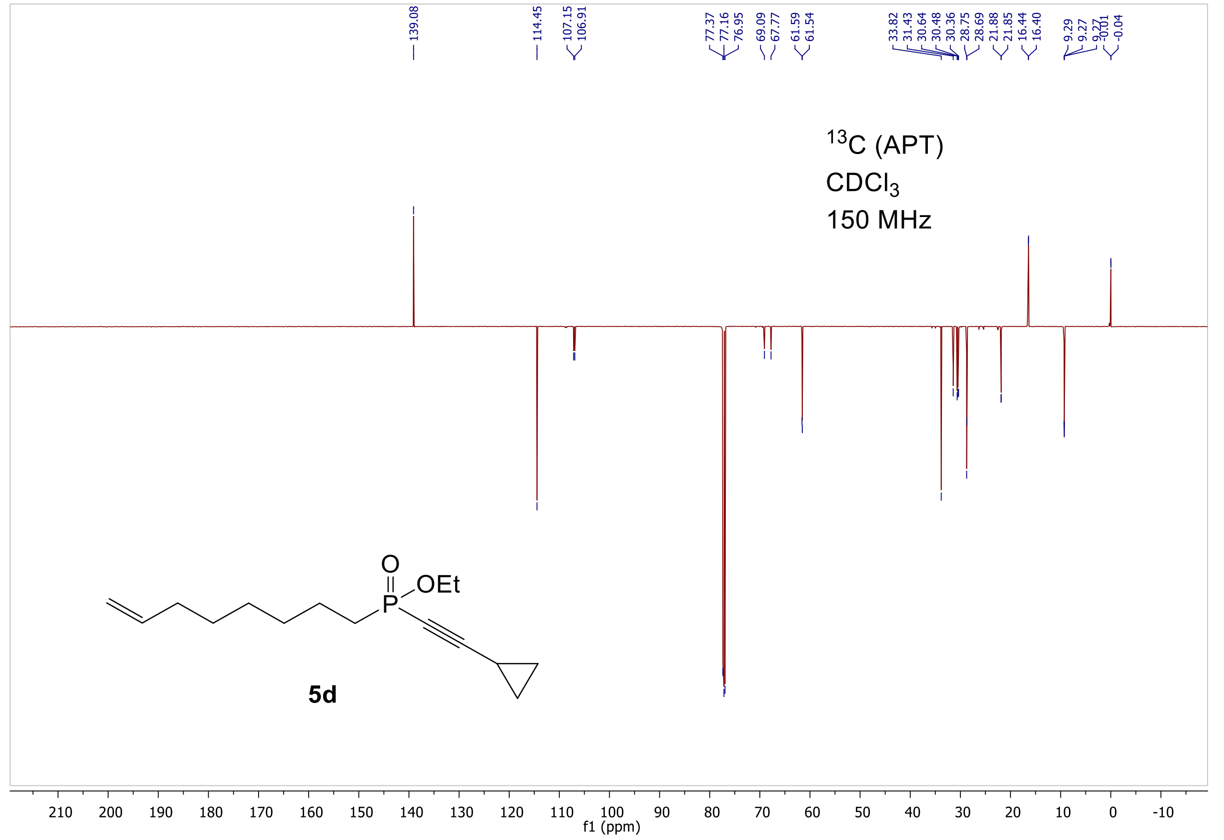

^{13}C (APT)
CDCl$_3$
150 MHz
O
OEt
P
5d
f1 (ppm)

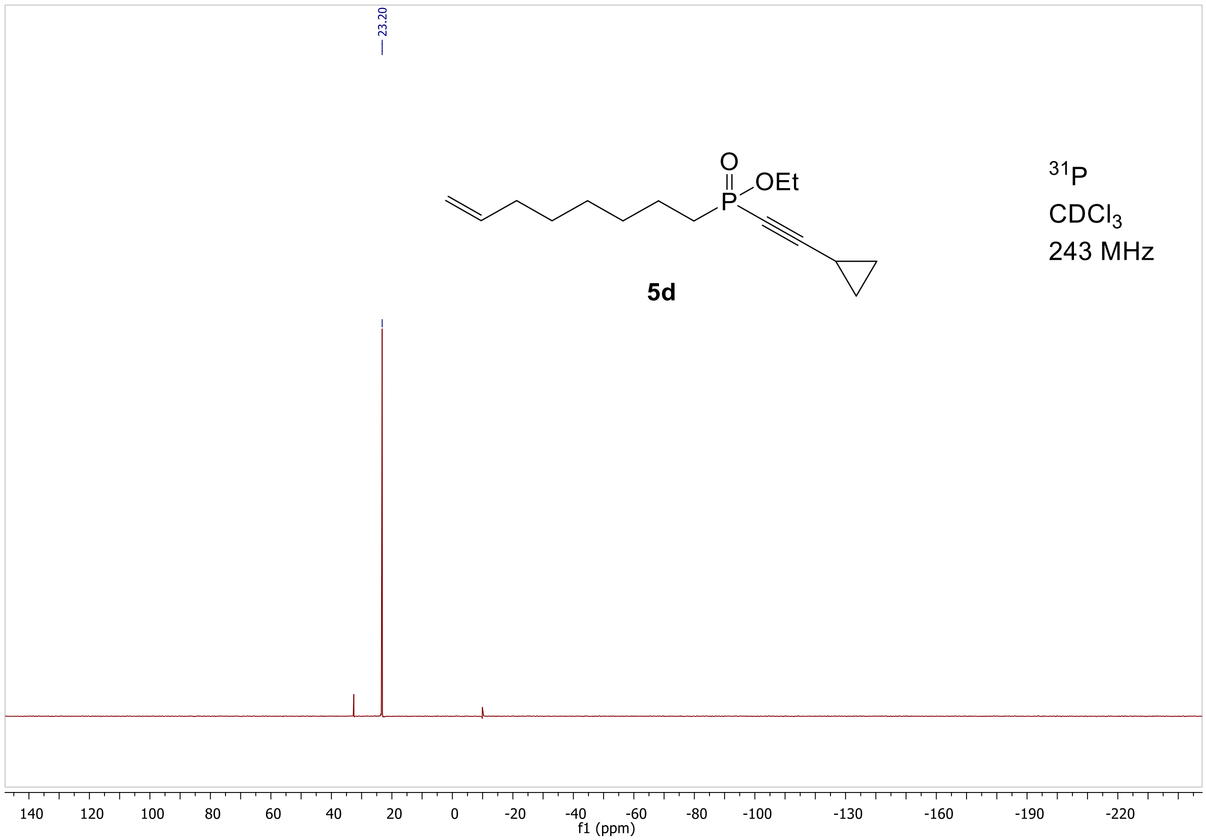

23.20
O
OEt
P
5d
^{31}P
$CDCl_3$
243 MHz
140
120
100
80
60
40
20
0
-20
-40
-60
-80
-100
-130
-160
-190
-220
f1 (ppm)

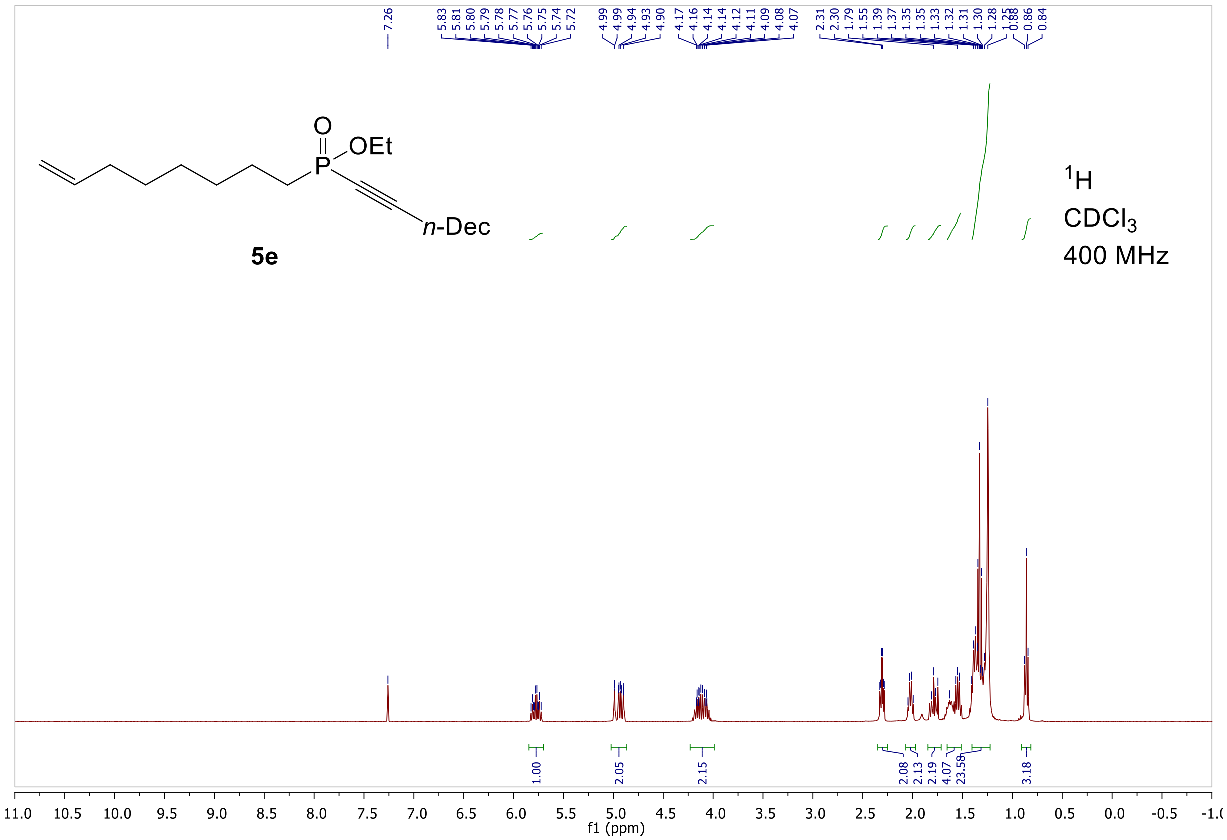

O
OEt
P
n-Dec
5e
^{1}H
CDCl3
400 MHz
f1 (ppm)

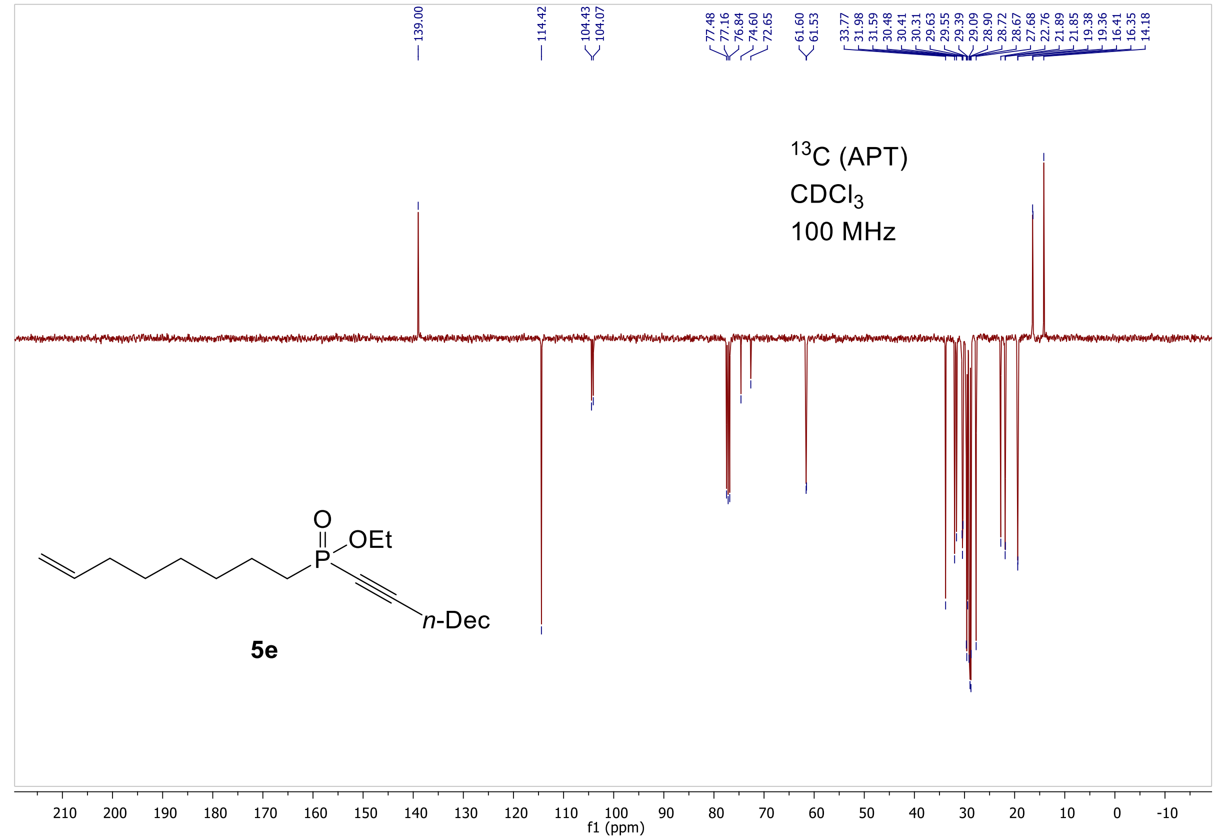

^{13}C (APT)
CDCl3
100 MHz
O
OEt
P
n-Dec
5e
f1 (ppm)

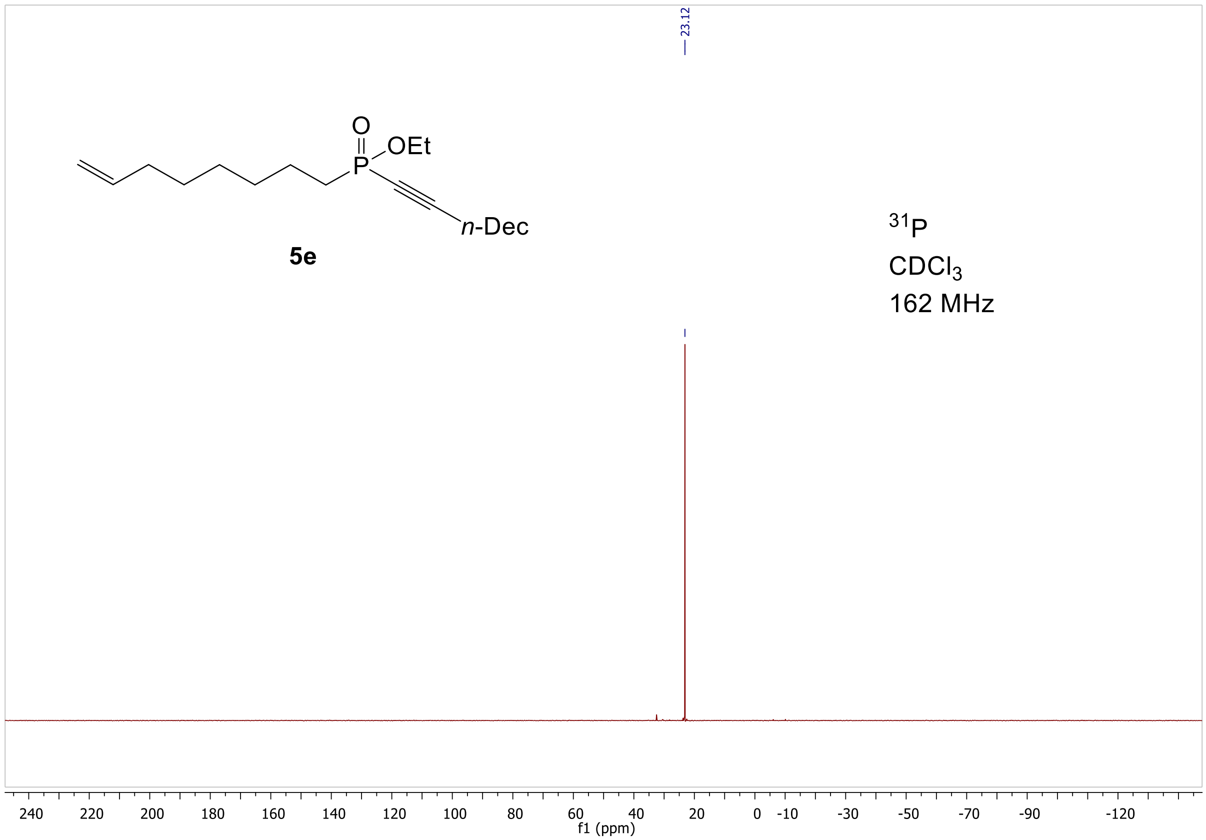

23.12
O
OEt
P
n-Dec
5e
^{31}P
CDCl$_3$
162 MHz
240
220
200
180
160
140
120
100
80
60
40
20
0
-10
-30
-50
-70
-90
-120
f1 (ppm)

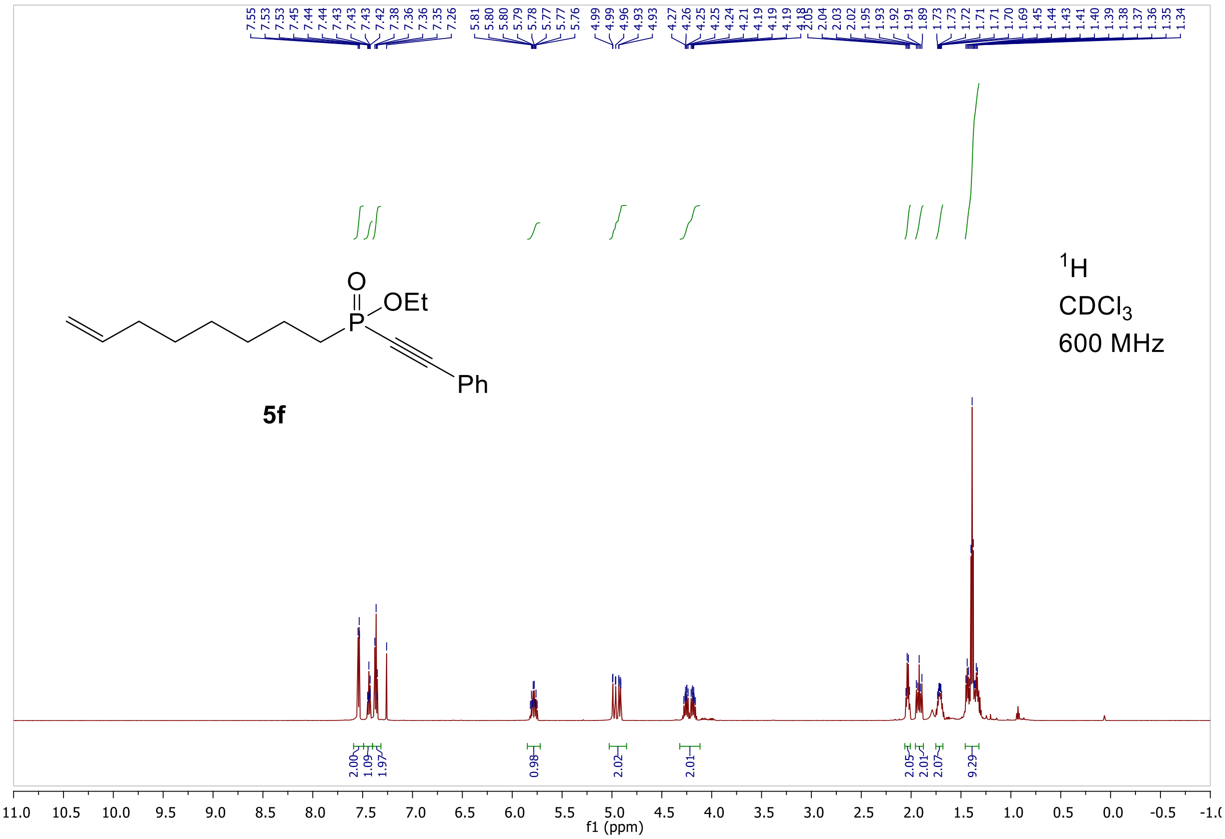

5f
OEt
Ph
^{1}H
CDCl$_3$
600 MHz
f1 (ppm)

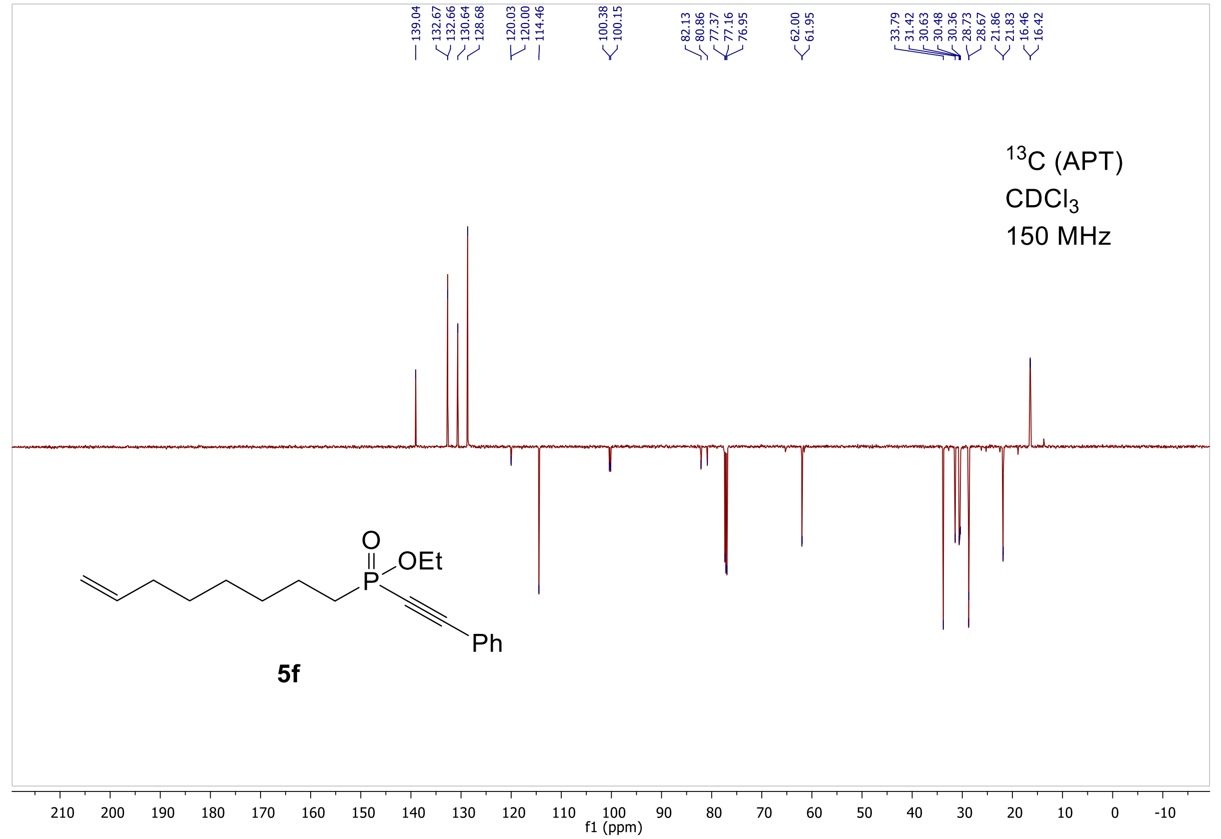

5f
OEt
Ph
^{13}C (APT)
CDCl$_3$
150 MHz
f1 (ppm)

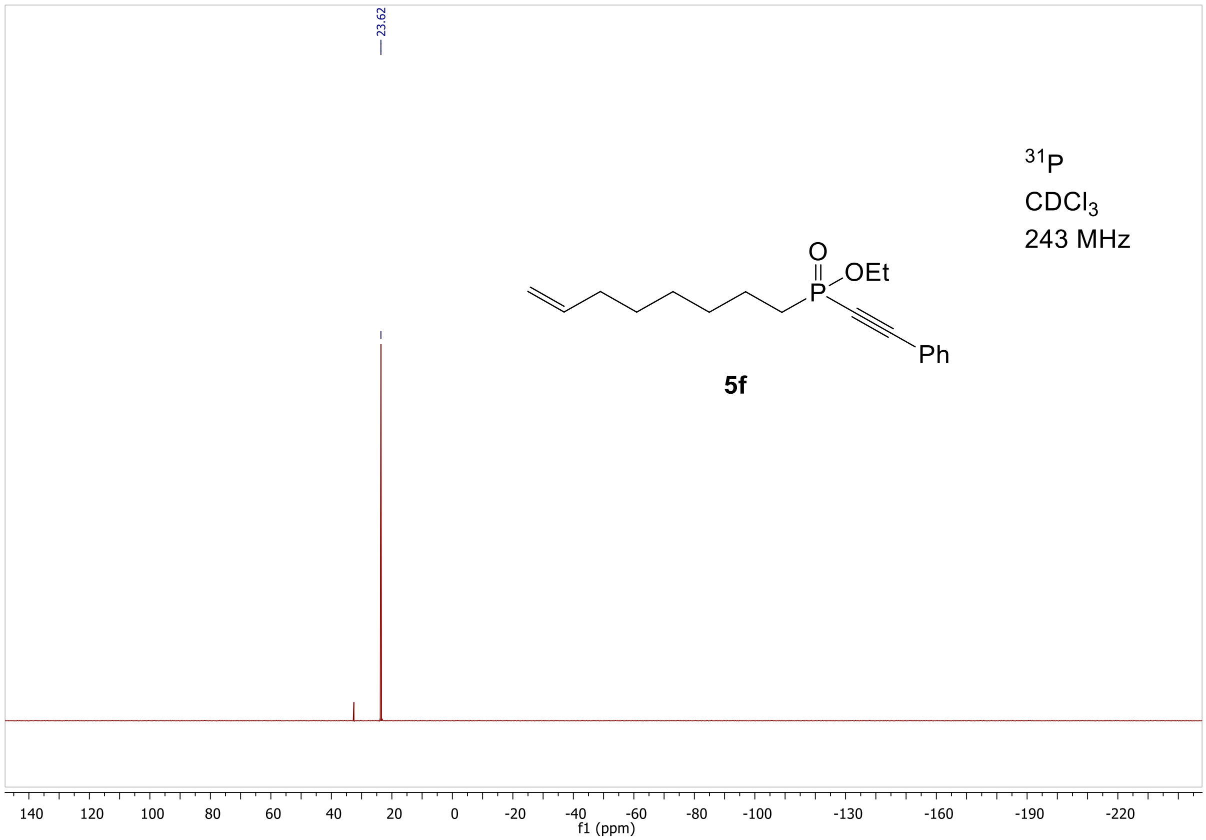

23.62
^{31}P
CDCl$_3$
243 MHz
O
OEt
P
Ph
5f
140 120 100 80 60 40 20 0 -20 -40 -60 -80 -100 -130 -160 -190 -220
f1 (ppm)

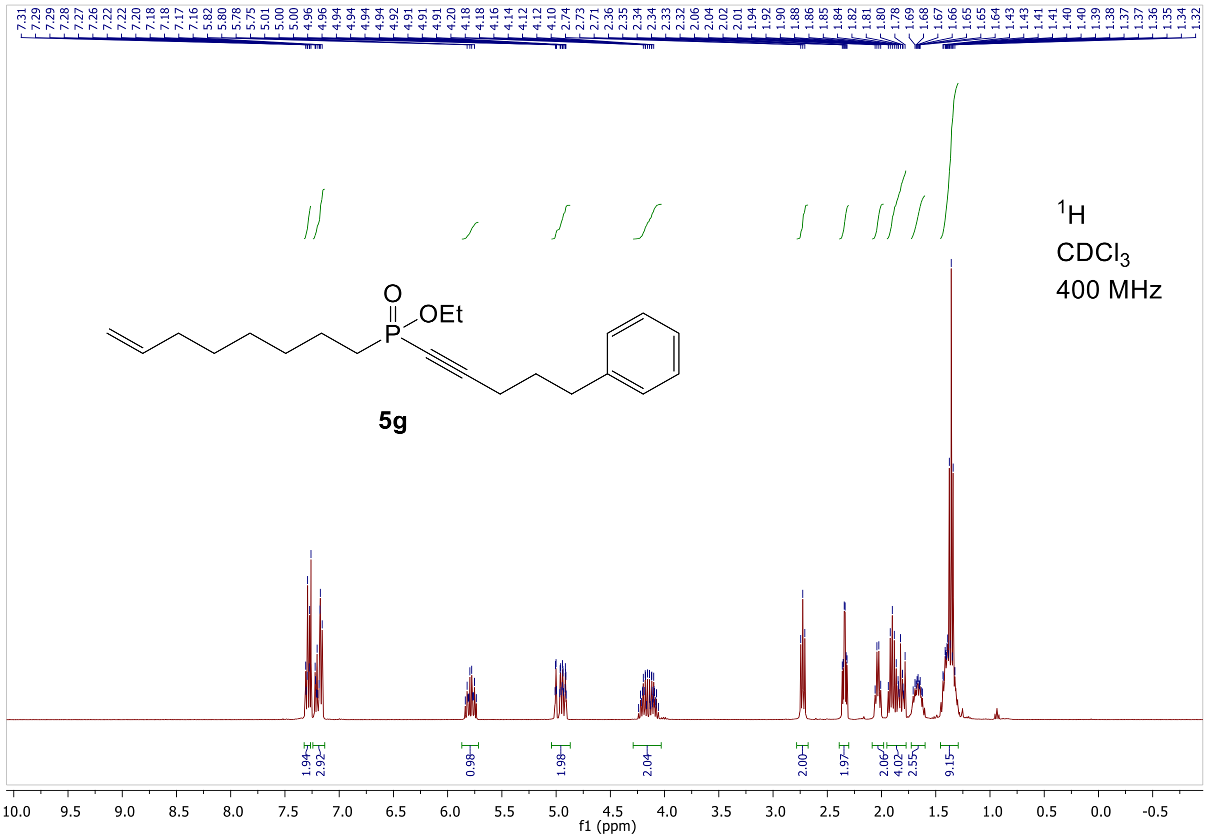

1H
CDCl3
400 MHz
O
P
OEt
5g
f1 (ppm)

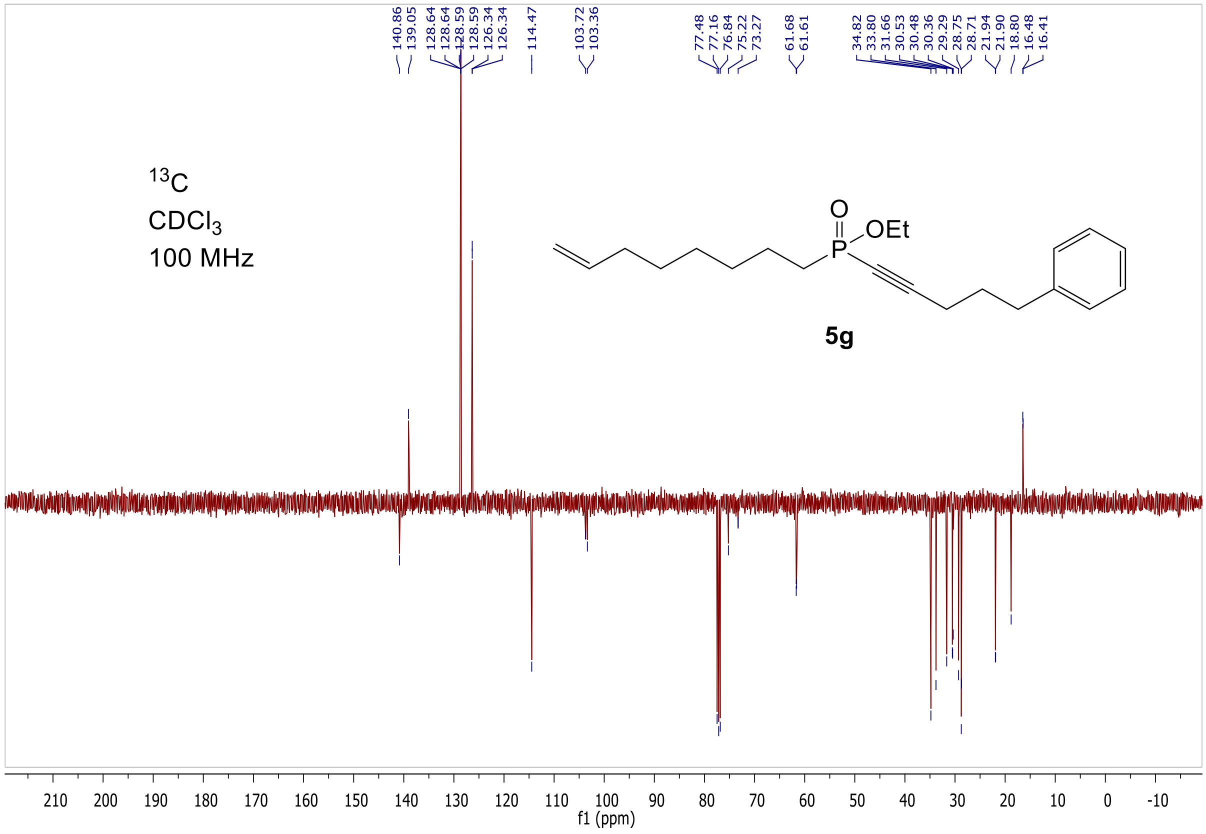

13C
CDCl3
100 MHz
O
P
OEt
5g
f1 (ppm)

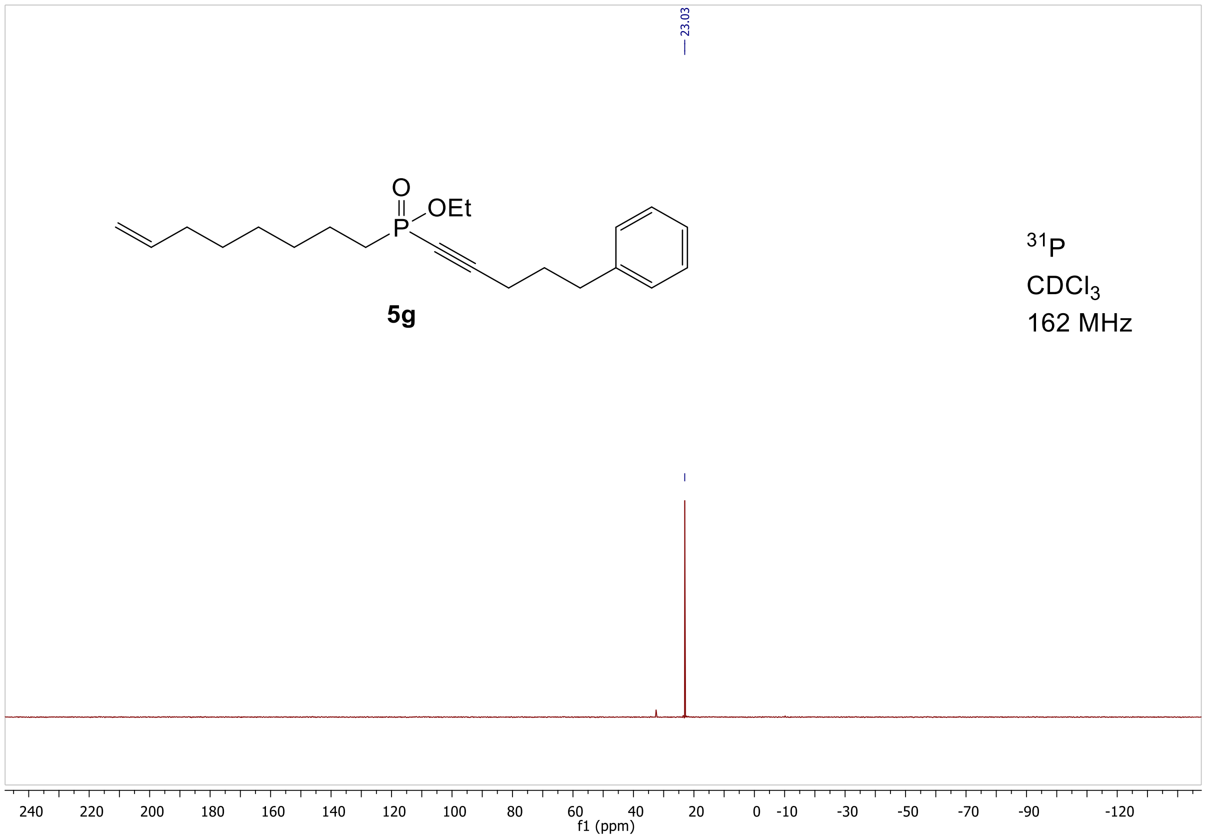
23.03
OEt
5g
^{31}P
$CDCl_3$
162 MHz
240 220 200 180 160 140 120 100 80 60 40 20 0 -10 -30 -50 -70 -90 -120
f1 (ppm)

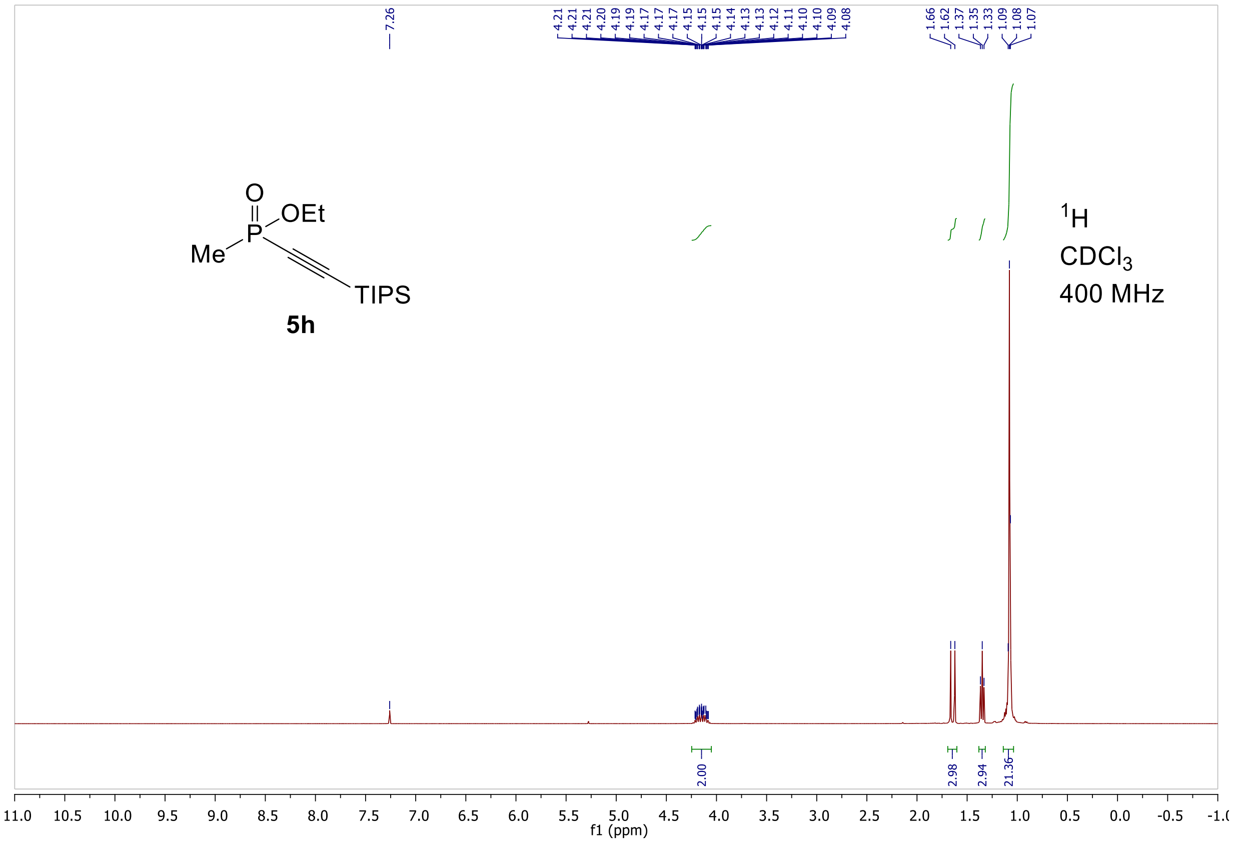
7.26
4.21
4.21
4.21
4.20
4.19
4.19
4.17
4.17
4.17
4.15
4.15
4.15
4.14
4.13
4.13
4.12
4.11
4.10
4.10
4.09
4.08
1.66
1.62
1.37
1.35
1.33
1.09
1.08
1.07
O
OEt
P
Me
TIPS
5h
1H
CDCl3
400 MHz
2.00
2.98
2.94
21.36
11.0
10.5
10.0
9.5
9.0
8.5
8.0
7.5
7.0
6.5
6.0
5.5
5.0
4.5
4.0
3.5
3.0
2.5
2.0
1.5
1.0
0.5
0.0
-0.5
f1 (ppm)

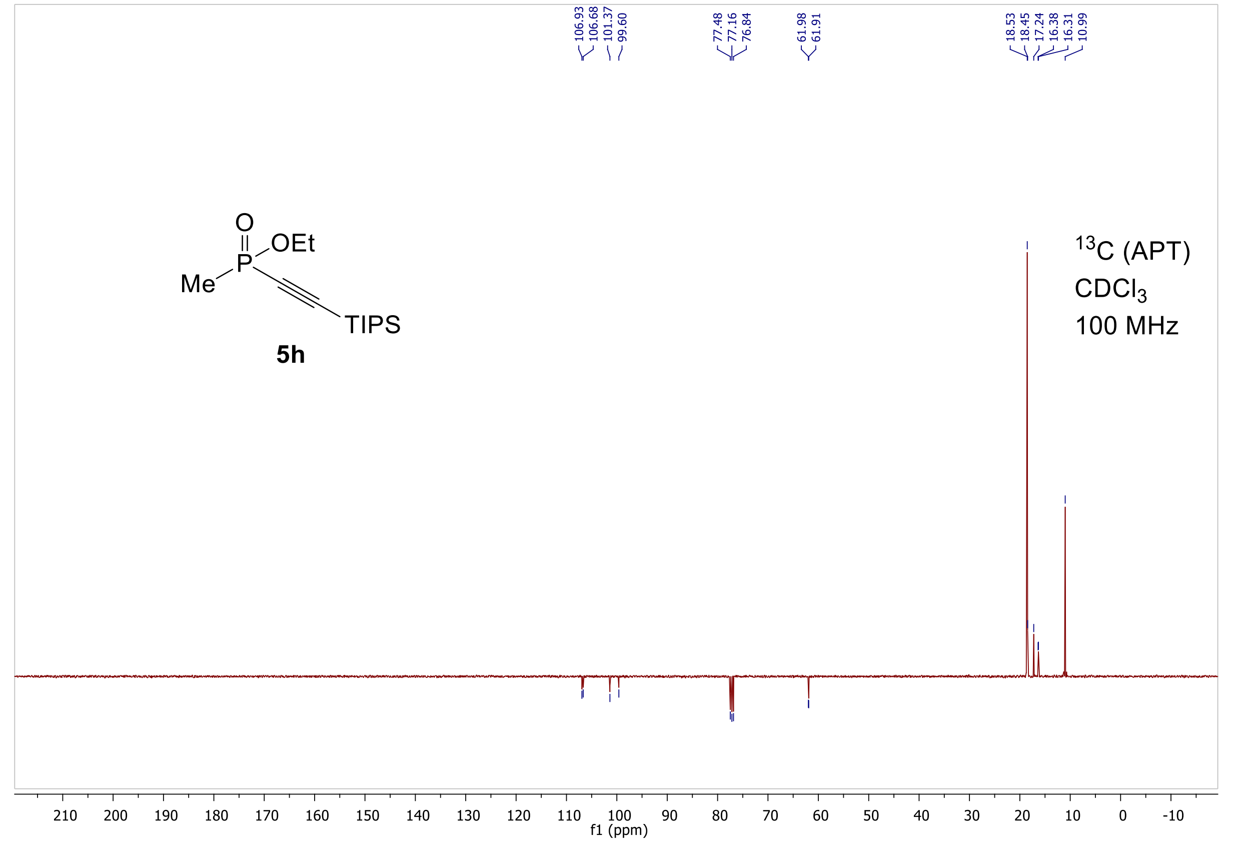
106.93
106.68
101.37
99.60
77.48
77.16
76.84
61.98
61.91
18.53
18.45
17.24
16.38
16.31
10.99
O
OEt
P
Me
TIPS
5h
13C (APT)
CDCl3
100 MHz
210
200
190
180
170
160
150
140
130
120
110
100
90
80
70
60
50
40
30
20
10
0
-10
f1 (ppm)

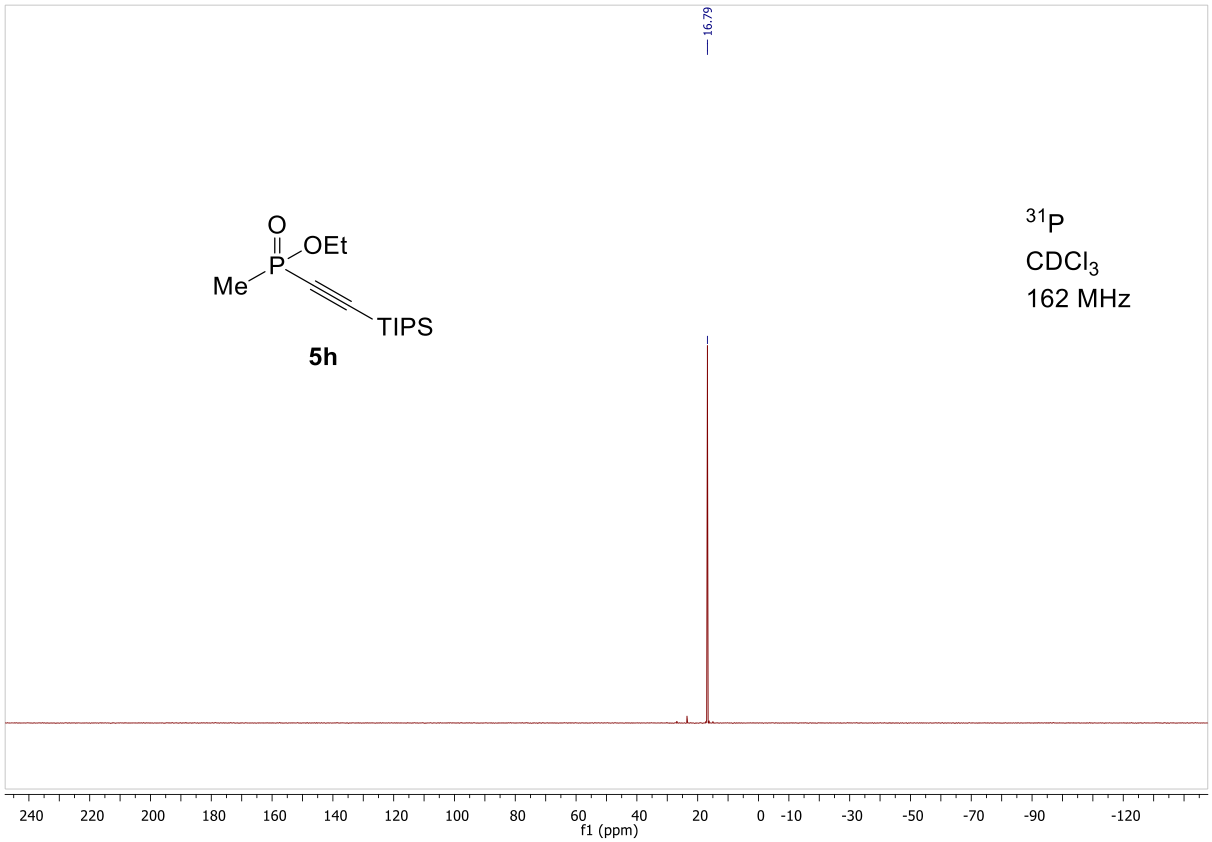

16.79
O
OEt
P
Me
TIPS
5h
31P
CDCl3
162 MHz
240 220 200 180 160 140 120 100 80 60 40 20 0 -10 -30 -50 -70 -90 -120
f1 (ppm)
